# Standards for practical intravenous rapid drug desensitization & delabeling: A WAO committee statement

**DOI:** 10.1016/j.waojou.2022.100640

**Published:** 2022-05-31

**Authors:** Emilio Alvarez-Cuesta, Ricardo Madrigal-Burgaleta, Ana D. Broyles, Javier Cuesta-Herranz, Maria Antonieta Guzman-Melendez, Michelle C. Maciag, Elizabeth J. Phillips, Jason A. Trubiano, Johnson T. Wong, Ignacio Ansotegui, Steering Committee Authors, F. Runa Ali, Denisse Angel-Pereira, Aleena Banerji, Maria Pilar Berges-Gimeno, Lorena Bernal-Rubio, Knut Brockow, Ricardo Cardona Villa, Mariana C. Castells, Jean-Christoph Caubet, Yoon-Seok Chang, Luis Felipe Ensina, Manana Chikhladze, Anca Mirela Chiriac, Weng-Hung Chung, Motohiro Ebisawa, Bryan Fernandes, Lene Heise Garvey, Maximiliano Gomez, Javier Gomez Vera, Sandra Gonzalez Diaz, David I. Hong, Juan Carlos Ivancevich, Hye-Ryun Kang, David A. Khan, Merin Kuruvilla, Jose Ignacio Larco Sousa, Patricia Latour-Staffeld, Anne Y. Liu, Eric Macy, Hans Jorgen Malling, Jorge Maspero, Sara M. May, Cristobalina Mayorga, Miguel A. Park, Jonathan Peter, Matthieu Picard, Tito Rodriguez-Bouza, Antonino Romano, Mario Sanchez-Borges, Luciana Kase Tanno, Maria Jose Torres, Alicia Ureña-Tavera, Rocco L. Valluzzi, Gerald W. Volcheck, Masao Yamaguchi, Review Panel Members

**Affiliations:** l Hospital Universitario de Canarias, Tenerife, Spain; m Allergy Division, Ramon y Cajal University Hospital, Madrid, Spain; n Department of Dermatology and Allergy Biederstein, School of Medicine, Technical University of Munich, Munich, Germany; o Universidad de Antioquia, Medellín, Colombia; p Division of Allergy and Clinical Immunology, Department of Medicine, Brigham & Women's Hospital, Boston, MA, USA; q Pediatric Allergy Unit, Geneva University Hospitals, Geneva, Switzerland; r Division of Allergy and Clinical Immunology, Department of Internal Medicine, Seoul National University Bundang Hospital, Seoul National University College of Medicine, Seoul, Republic of Korea; s Division of Allergy, Clinical Immunology and Rheumatology, Department of Pediatrics, Federal University of Sao Paulo, Brazil; t Medical Faculty at Akaki Tsereteli State University, KuTaisi, Tskaltubo, Georgia; u Division of Allergy, Department of Pulmonology, Hôpital Arnaud de Villeneuve, University Hospital of Montpellier, Montpellier, France; v Department of Dermatology, Chang Gung Memorial Hospital, Taipei, Linko and Keelung, Taiwan; w Clinical Research Center for Allergy and Rheumatology, National Hospital Organization Sagamihara National Hospital, Sagamihara, Kanagawa, Japan; x Allergy Clinic, Copenhagen University Hospital Gentofte, Copenhagen, Denmark; y School of Health Sciences, Catholic University of Salta, Argentina; z Institute of Security and Social Services of State Workers, López Mateos Regional Hospital, Mexico City, Mexico; aa Regional Center of Allergy and Clinical Immunology, University Hospital “Dr. José Eleuterio González”, Gonzalitos y Madero s/n Colonia Mitras Centro, Monterrey, Mexico; ab Servicio de Alergia e Immunologia, Clinica Santa Isabel, Buenos Aires, Argentina; ac Institute of Allergy and Clinical Immunology, Seoul National University Medical Research Center, Department of Internal Medicine, Seoul National University College of Medicine, Seoul, Republic of Korea; ad Department of Internal Medicine, Division of Allergy & Immunology, University of Texas Southwestern Medical Center, Dallas, TX, USA; ae Division of Pulmonary, Allergy, Critical Care & Sleep Medicine, Department of Medicine, Emory University School of Medicine, Atlanta, GA, USA; af Allergy Department, Clinica San Felipe, Lima, Peru; ag Centro Avanzado de Alergia y Asma de Santo Domingo, Santo Domingo, Dominican Republic; ah Stanford University School of Medicine, Palo Alto, CA, USA; ai Southern California Permanente Medical Group, Kaiser Permanente Southern California, San Diego Medical Center, San Diego, CA, USA; aj Danish Allergy Centre, University of Copenhagen, Copenhagen, Denmark; ak Allergy and Respiratory Research Unit, Fundación CIDEA, Buenos Aires, Argentina; al Division of Pulmonary, Critical Care, Sleep & Allergy, University of Nebraska Medical Center, Omaha, NE, USA; am Allergy Unit and Research Group, Hospital Regional Universitario de Málaga, UMA-IBIMA-BIONAND, ARADyAL, Málaga, Spain; an Division of Allergic Diseases, Mayo Clinic, Rochester, MN, USA; ao Division of Allergology and Clinical Immunology, Department of Medicine, University of Cape Town, Cape Town, South Africa; ap Department of Medicine, Division of Allergy and Immunology, Hôpital Maisonneuve-Rosemont, Université de Montréal, Montréal, Québec, Canada; aq Centro de Patología Alérgica, Hospital Quirón Palmaplanas, Palma, Spain; ar Oasi Research Institute – IRCCS, Troina, Italy; as Clinica Union Medica del Norte, Santiago, Dominican Republic; at Multifactorial and Systemic Diseases Research Area, Predictive and Preventive Medicine Research Unit, Division of Allergy, Bambino Gesù Children's Hospital IRCCS, Rome, Italy; au Division of Allergic Diseases, Mayo Clinic, Rochester, MN, USA; av Division of Respiratory Medicine, Third Department of Medicine, Teikyo University Chiba Medical Center, Anesaki, Ichihara, Chiba, Japan; aw Department of Clinical Medicine, University of Copenhagen, Copenhagen, Denmark; ax Allergy & Severe Asthma Service, St Bartholomew's Hospital, Barts Health NHS Trust, London, UK; ay Division of Rheumatology, Allergy and Immunology, Massachusetts General Hospital, Boston, MA, USA; az Allergy and Clinical Immunology Department, Centro Médico Docente La Trinidad, Caracas, Venezuela; ba Allergy and Clinical Immunology Department, Clínica El Avila, Caracas, Venezuela; a Ramon y Cajal University Hospital, Madrid, Spain; b Allergy & Severe Asthma Service, St Bartholomew's Hospital, Barts Health NHS Trust, London, UK; c Drug Desensitisation Centre, Catalan Institute of Oncology (ICO), Barcelona, Spain; d Division of Allergy & Immunology, Boston Children's Hospital, Boston, MA, USA; e Department of Allergy and Immunology, FIIS-Fundación Jiménez Díaz, UAM, Madrid, Spain; f RETIC ARADyAL, Instituto de Salud Carlos III, Spain; g Section of Immunology, HIV and Allergy, Department of Medicine, Clinical Hospital University of Chile, Chile; h Department of Medicine & Pathology, Microbiology and Immunology, Vanderbilt University Medical Center, Nashville, TN, USA; i Department of Infectious Diseases and Centre for Antibiotic Allergy and Research, Austin Health, Heidelberg, Australia; j Division of Rheumatology, Allergy and Immunology, Massachusetts General Hospital, Boston, MA, USA; k Hospital Quironsalud Bizkaia, Bilbao-Erandio, Spain

**Keywords:** Drug allergy, Drug desensitization, Drug challenge, Drug provocation test, Delabeling, Chemotherapy, Skin test, Risk stratification, Biological agents, Antibiotics, Penicillins, Betalactams, Antibiotic desensitization, Precision medicine, Personalized medicine

## Abstract

Drug hypersensitivity reactions (DHRs) to intravenous drugs can be severe and might leave patients and doctors in a difficult position where an essential treatment or intervention has to be suspended. Even if virtually any intravenous medication can potentially trigger a life-threatening DHR, chemotherapeutics, biologics, and antibiotics are amongst the intravenous drugs most frequently involved in these reactions. Admittedly, suspending such treatments may negatively impact the survival outcomes or the quality of life of affected patients.

Delabeling pathways and rapid drug desensitization (RDD) can help reactive patients stay on first-choice therapies instead of turning to less efficacious, less cost-effective, or more toxic alternatives. However, these are high-complexity and high-risk techniques, which usually need expert teams and allergy-specific techniques (skin testing, *in vitro* testing, drug provocation testing) to ensure safety, an accurate diagnosis, and personalized management. Unfortunately, there are significant inequalities within and among countries in access to allergy departments with the necessary expertise and resources to offer these techniques and tackle these DHRs optimally.

The main objective of this consensus document is to create a great benefit for patients worldwide by aiding allergists to expand the scope of their practice and support them with evidence, data, and experience from leading groups from around the globe.

This statement of the Drug Hypersensitivity Committee of the World Allergy Organization (WAO) aims to be a comprehensive practical guide on the technical aspects of implementing acute-onset intravenous hypersensitivity delabeling and RDD for a wide range of drugs. Thus, the manuscript does not only focus on clinical pathways. Instead, it also provides guidance on topics usually left unaddressed, namely, internal validation, continuous quality improvement, creating a healthy multidisciplinary environment, and redesigning care (including a specific supplemental section on a real-life example of how to design a dedicated space that can combine basic and complex diagnostic and therapeutic techniques in allergy).

## Introduction

### Motivation for this document

Rapid drug desensitization (RDD) is a technique used to temporarily modify a patient's immune response to a drug in a few hours. RDD is helpful in patients who have experienced confirmed drug hypersensitivity reactions (DHRs) that are amenable to desensitization, including anaphylaxis. When these patients have a potentially life-threatening chronic condition such as cancer, an inflammatory disease, or an acute infection, where the implicated drug is the most efficacious, cost-effective, or safest therapy, RDD may be life-saving. The fact that frequently there are no ideal treatment alternatives in these situations is presumably the most substantial incentive to desensitize patients.^1^

In the last twenty years, the use of RDD has increased exponentially, and so have the reports in the literature showing the feasibility and benefits of this technique.^1^, ^2^, ^3^, ^4^, ^5^ However, although there are examples of RDD for most existing drugs, a simple literature search would show the reader how DHRs to chemotherapy and biologics have been the leading cause of this increase in RDD. Of note, chemotherapy and biologics are usually intravenous drugs, which require a different approach than orally administered drugs.

The number of patients requiring intravenous procedures in many allergy departments has grown dramatically. To illustrate this, we will draw attention to a real-life example, the experience at Ramon y Cajal University Hospital (RCUH), Madrid, Spain. In the last decade, the number of RDD patients referred annually increased about 30%. In addition, the number of annual intravenous drug provocation testing (DPT) procedures (a diagnostic technique sometimes used before RDD to delabel patients, ie, to confirm or rule out hypersensitivity) increased over 85%.^2^^,^^3^

Allergists worldwide could potentially struggle to meet this increase in the demand for high-risk and high-complexity intravenous procedures. While allergists might find an abundance of valuable clinical data and reviews on RDD, there is a lack of general guidance on the logistics of implementing intravenous delabeling and RDD into the daily practice of an allergy department.

The allergy team at RCUH adapted previously published RDD protocols to meet specific local requirements and validated these RDD protocols for their population.^3^ Moreover, to keep the highest standards of care despite the increase in demand, they also validated diagnostic tools for their specific population.^4^ However, it is not easy to find guidance on the importance of internal validation or the best approach to achieve it for RDD protocols and their associated diagnostic pathways.

Over the years, the authors audited their diagnostic and therapeutic pathways.^2^, ^3^, ^4^ Combined with data from other groups, these audit findings led to a reconsideration of the approach to RDD and resulted in specific improvements.^5^^,^^6^ Unfortunately, reviews and clinical guidelines usually overlook the importance of auditing and continuous quality improvement.

The RCUH team advocated for a multidisciplinary approach since the inception of their “Desensitization Program”. Collaboration with non-allergists triggered thought-provoking questions that extended beyond the scope of Allergy as a speciality. For example, the authors studied whether reactive patients undergoing RDD had similar survival rates to non-reactive patients receiving standard chemotherapy (ie, without RDD).^7^ The resulting data helped to support the hypothesis that RDD does not affect the efficacy of chemotherapy. Only a healthy multidisciplinary environment could foster this kind of cooperation. Strikingly, articles in the field fail to offer support tools to create these environments.

Inevitably, the increasing demand for RDD had a direct effect on the size of the RCUH cohort. On the one hand, this helped the authors identify groups of patients that could benefit from a tailored approach.^8^, ^9^, ^10^, ^11^, ^12^ On the other hand, it created a managerial crisis that led to a complete structural redesign to cope with the needs of a twenty-first-century allergy department. Arguably, the notion that implementing RDD is a prohibitively resource-intensive endeavour and a major managerial challenge is probably one of the main reasons that put off many colleagues from giving service to patients in need of RDD. Unfortunately, the lack of published recommendations on how to tackle these issues does not help to improve this situation.

The RCUH is not an isolated example. Other groups have recorded similar processes in the literature. However, the most remarkable examples in RDD to chemotherapy and biologics are Brigham and Women's Hospital, Boston, MA, United States (BWH) and Massachusetts General Hospital, Boston, MA, United States (MGH).^5^

For these reasons, this consensus document aims to clarify complex concepts usually unaddressed in other publications. Based on various real-life models, the manuscript will go beyond clinical pathways to fill these voids of guidance, namely, internal validation, continuous quality improvement, creating a healthy multidisciplinary environment, and redesigning care. Following a diverse range of real-life experiences, this manuscript aims to be a comprehensive practical guide on the technical aspects of acute-onset intravenous delabeling and RDD (with occasional allusions to oral or subcutaneous drugs and other types of reactions). This article does not intend to be a clinical review of the latest publications on the topic. Instead, we have brought together various experts from the field to share their experiences on the practical aspects of using intravenous RDD.

The main objective of this document is to create a great benefit for patients worldwide by aiding allergists to expand the scope of their practice and support them with evidence, data, and experience.

### Guide to approaching this document

This manuscript will mainly explore the practicalities of implementing intravenous delabeling and desensitization to a wide range of drugs and using different approaches.

Section 1 will introduce several general concepts that will appear recurrently over the manuscript: classification systems in drug allergy, drug provocation testing or drug challenge, and rapid drug desensitization.

Section 2 will focus on the managerial and governance issues of implementing these techniques, which are aspects of clinical practice that most consensus documents frequently overlook. This section and the practical example on supplemental text 1 should offer insight into how to approach these issues successfully and efficiently for emerging and established groups alike.

Sections 3–5, 3–5, 3–5 will focus on delabeling, which is fundamental before considering RDD. The pathways for delabeling can significantly vary depending on the type of drug and the number of patients that potentially have a label of allergy. For example, delabeling penicillin allergy will need cooperation with other hospital teams and the community, whereas delabeling chemotherapy allergy will need a niche service offered in specialist centers. The information provided in these sections should empower allergy departments worldwide with tools to design their delabeling strategies.

Sections 6–8, 6–8, 6–8 will deal with RDD and will thoroughly approach the practicalities of using this tool. The reader will understand how RDD saves lives and justifies why hospitals need a solid hospital-based allergy department with specific resources and capacity for urgent assessment and management of the patients in need.

Section 9 is an essential complement to the previous sections, focusing on delabeling and RDD with drugs when evidence is lacking. The need to offer quick, safe, and effective solutions forces many allergists on painstaking journeys when receiving referrals for one of these patients. Thus, we envisaged this section as a practical guide for the allergist.

Section 10 will explicitly focus on pediatric patients. These patients have specific care needs that are different to those of adult patients.

Finally, Section 11 will focus on certain controversies and unmet needs. Bringing together experts in drug allergy with contrasting opinions on approaching various issues will inevitably generate inconsistencies in the document. There are many areas where evidence is lacking, and practice can be drastically different depending on patient populations and regional differences. For this reason, we collected the very pertinent comments of a review panel of expert allergists from all around the globe in this section.

The readers will notice that different leading groups in drug allergy have authored different sections, and these authors have provided an immense range of supplemental material. This invaluable material should be available through the online repository.

### Key practice points

- Skin testing (ST) concentrations may differ depending on whether we prioritize sensitivity or specificity, so cut-off points need internal validation.
- DPT (or drug challenge) is the diagnostic gold standard and the final step in the delabeling process. Therefore, authors should disclose data on DPT when publishing an article since the vast majority of patients where DPT is used are successfully delabeled (ie, articles not including DPT within their allergy workup might incur a selection bias of an overestimation of the amount of really hypersensitive patients).
- RDD is more than a mere protocol but needs complex multidisciplinary logistic support like other highly specialized and high-risk procedures involving high-complexity in medicine.
- RDD protocols are personalized. However, they must meet particular criteria.
- Allergy departments may benefit from access to a Technical Area for Diagnostic and Therapeutic Procedures in Allergy (throughout the manuscript, allergy technical area). The allergy technical area is an allergy-dedicated space used to diagnose and manage allergy patients and may receive different names in different centers (see supplemental text 1 for a practical, real-life example). This area will be divided into several “safety areas” depending on risk assessment. In addition, a unique “safety area” of the allergy technical area will be dedicated to intravenous delabeling and RDD.

## Section 1: General concepts

### Classification of drug hypersensitivity reactions

Despite the enormous efforts of many societies, poor documentation and mislabelling are strikingly frequent in drug allergy.^5^^,^^13^, ^14^, ^15^ As a result, virtually all the reliable data on well-characterized DHRs to intravenous medications such as chemotherapy, biologics, or antibiotics comes from a few expert centers. For instance, it is difficult to gather an accurate snapshot of DHRs to chemotherapy or biologics outside expert centers, probably due to the poor representation of drug allergy in the International Classification of Diseases (ICD), which has only recently been aligned with current allergy practice in the ICD-11, thanks to the efforts by Tanno LK et al.^16^^,^^17^ However challenging, adequate classification, diagnosis, and documentation are of the essence to further understand and manage DHRs, especially when intravenous drugs are involved.

DHRs are usually divided according to the timing of their onset into immediate (I-DHR) and non-immediate (NI-DHR).^18^ Severe immediate reactions to drugs and vaccines such as anaphylaxis typically occur within 1 hour of exposure; however, most classifications consider that I-DHRs can happen for some drugs as late as within 6 hours of exposure (or 4 hours for vaccines).^18^, ^19^, ^20^ In parallel, DHRs are further categorized using the Gell and Coombs classification, which explains the clinical presentation of the DHRs by their mechanisms.^19^, ^21^ As we understand more about mechanisms and risk of DHRs, classifications will be further refined. However, classifications such as Gell and Coombs' still provide a valuable framework to guide patient allergy workup and management pathways.^19^, ^21^

The type I Gell and Coombs category features immediate IgE-mediated DHRs leading to mast cell/basophil degranulation with symptoms from mild urticaria to anaphylaxis. Some authors argue that type I DHRs should also include non-IgE-mediated activation of mast cells/basophils.^19^ Although controversial, we have included non-IgE-mediated DHRs featuring mast cell/basophil release symptoms as type I Gell and Coombs reactions.

The type II Gell and Coombs category features cytotoxic IgM- or IgG-mediated reactions against a cell surface antigen, such as drug-induced hemolytic anemia.^19^ Type III DHRs feature immune complex deposition reactions with complement activation (eg, serum sickness-like DHRs). Finally, type IV DHRs feature delayed T-lymphocyte-mediated reactions and DHRs for which the mechanism involves cells other than (or in addition to) T cells. Examples of type IV DHRs range from the typically milder contact dermatitis or delayed maculopapular rashes to the more severe and even life-threatening drug reaction with eosinophilia and systemic symptoms (DRESS), Stevens-Johnson's Syndrome (SJS), or toxic epidermal necrolysis (TEN).^19^

However, some DHRs do not neatly fall into a clear category of this classification system or have yet unknown mechanisms. For example, some patients reacting to oxaliplatin or biologics can show mixed patterns of type I Gell and Coombs hypersensitivity and “cytokine-release-syndrome-like” reactions with fever/chills, generalized malaise, flushing, or hypotension, with elevations of both tryptase and IL-6.^21^, ^22^, ^23^

Moreover, we cannot forget that DHRs are only a subset of adverse drug reactions. More holistic classifications divide adverse drug reactions into on-target (referring to those that occur due to the predictable pharmacological action of the drug) and off-target reactions. Off-target reactions are further divided into cellular toxicity, immune receptor interaction (eg, non-IgE mediated reactions associated with MRGPRX2), and true immunologically mediated reactions that can be antibody-mediated or T-cell mediated.^24^

The range of DHRs that biologics can trigger has spurred the publication of several classifications.^25^ However, Isabwe et al^23^ recently published an article focusing on managing DHRs to biologics and RDD. The authors proposed a pragmatic classification based on the allergy workup, separating patients into different endophenotypes and comparing their RDD outcomes. The same group successfully applied these endophenotypes to oxaliplatin DHRs.^22^ It combines previous Gell & Coombs' types with recent findings to classify DHRs into type I (IgE- or non-IgE-dependent), type II, type III, type IV, cytokine release reactions, and mixed reactions. This novel classification seems promising for the practical assessment of DHRs to biologics and chemotherapy, especially as novel biomarkers emerge.^26^

In any case, despite the limited tools currently available, a systematic approach can be helpful. A detailed clinical history and assessment of all the available data, including serum biomarkers extracted during the reaction (eg, tryptase, IL6, which would normally involve liaison with other services, such as emergency departments or infusion centers, to ensure samples are collected properly), and a deep understanding of how all these classifications and endophenotypes of patients interact will be essential for successful categorization of a DHR.^5^^,^^21^^,^^27^

Grading the severity of DHRs can also be challenging, as the ideal severity classification is yet to be determined. Virtually every allergy society has published a different grading system, and these systems can often vary based upon the type of triggers, eg, allergen-specific immunotherapy vs contrast media. However, many authors call for simplification and a common classification.^28^ The World Allergy Organization (WAO) has recently published a guidance document on anaphylaxis with a more encompassing severity classification, which could be helpful in the future.^29^

However, these classifications usually focus on general allergy and are based on data from allergic reactions to any trigger. This can be limiting when grading DHRs to chemotherapy or biologics, which tend to feature symptoms not contemplated on standard grading systems. On the other hand, when assessing DHRs to chemotherapy or biologics, many authors find that non-allergy-focused classifications like the National Comprehensive Cancer Network (NCCN) guidelines, and the Common Terminology Criteria for Adverse Events (CTCAE) developed by the National Cancer Institute (NCI), are insufficient for the optimal assessment, classification, and management of these DHRs.^3^^,^^4^^,^^30^^,^^31^ A severity classification by Brown has been widely used as an alternative in grading the severity of these DHRs.^32^ However, given the unique characteristics of DHRs, some authors have proposed modified classifications for grading the severity of DHRs to chemotherapy and biologics.^2^^,^^30^ Finding an optimal and universal grading system for severity remains an unmet need.

### Drug provocation testing or drug challenge

DPT is a diagnostic technique that involves administering a drug to a patient who carries a label of an unconfirmed allergy to that drug, and it is the criterion standard to confirm or rule out an allergy.^33^ Indeed, the sensitivity and specificity of clinical history alone are usually unacceptably poor, whereas diagnostic tests such as ST or *in vitro* testing tend to be specific but not sensitive or may still need validation.^2^^,^^34^

DPT is, therefore, an essential tool for effectively delabeling patients. When assessing DHRs to intravenous drugs, this is helpful to avoid unnecessary RDDs,^2^^,^^6^^,^^35^, ^36^, ^37^, ^38^, ^39^ to study patients who received more than 1 drug simultaneously,^2^^,^^10^ and as the gold standard to validate other diagnostic tests.^4^^,^^6^^,^^34^ DPT is also a helpful tool to find possible drug alternatives in hypersensitive patients, for example, when there is a potential cross-reactivity or when the clinical history is unclear regarding culprit drugs.^33^

Despite these invaluable benefits, DPT is a high-risk technique, especially when dealing with highly sensitizing intravenous drugs such as chemotherapy or biologics.^5^^,^^6^ Therefore, careful patient selection and optimal risk-management plans are critical to ensure patient safety during intravenous DPT. DPT should be performed only by experts in drug allergy in highly specialized centers with access to allergy-dedicated spaces designed and prepared to carry out this technique.^5^^,^^6^

DPT is used principally with a diagnostic intention.^33^ Other terms like “drug challenge” or “rechallenge” are sometimes used interchangeably with DPT.^2^^,^^4^, ^5^, ^6^ Since a DPT intends to delabel a patient rather than provoke a drug hypersensitivity reaction, “drug challenge” is preferred in many countries as an alternative to the term DPT.^19^^,^^40^ Indeed, “provocation” could have a negative connotation for patients, and it might be a misleading term, as this test does not provoke a DHR most of the time. On the other hand, the term “challenge” could be a potentially less ominous word for patients. Unfortunately, allergy societies rarely involve patients or patient associations in nomenclature discussions, so we have no data on this. Nevertheless, in Europe, DPT remains the preferred term.^33^^,^^41^^,^^42^

In any case, especially regarding intravenous drugs, “challenge” or “rechallenge” are sometimes used to refer to other techniques with different purposes and motivations than protocolized diagnostic DPT. For example, in DHRs to intravenous drugs such as chemotherapy or biologics, these terms can be used for re-exposure using a modification of the infusion rate and additional premedications,^43^, ^44^, ^45^ or even “same-day rechallenge”.^46^^,^^47^ We will now explore the current evidence for using these techniques outside of the strict diagnostic intention of DPT.

When a patient reacts to an intravenous chemotherapeutic or biologic agent, there are clear disadvantages linked to a re-exposure using intensified premedication and reduced infusion rates. We must bear in mind that the manufacturer's instructions for biologics already include slow infusion rates that may be progressively increased depending on tolerance.^6^ Therefore, some authors consider re-exposure using additional premedication and modifications on the infusion rate not beneficial, let alone a missed opportunity for a reliable diagnosis and an adequate endophenotyping.^2^^,^^5^^,^^33^ Moreover, if such procedures are successful, it may be challenging to know whether these measures will be effective for the next administration or if they could have been avoided altogether (eg, because the true diagnosis was not a hypersensitivity reaction).^5^ Additionally, these techniques are sometimes performed by non-allergists and in suboptimal conditions, qualifying thus as an “uncontrolled challenge”. RCUH defined “uncontrolled challenge” as administering a culprit-drug to a reactive patient lacking allergy/risk assessment, in an inappropriate environment, by untrained or unaware personnel.^4^^,^^5^

An article by Levin et al described how the use of “same-day rechallenge” by non-allergists was reportedly helpful in mild reactions but not so much for moderate or severe reactions.^47^ However, “same-day rechallenge” in that article did not follow specific timings or homogeneous protocols, it was performed under heterogeneous circumstances (arguably qualifying as “uncontrolled challenge”, which is unsafe), and practitioners selected patients for “same-day rechallenge” following individual criteria. For these reasons, the efficacy of “same-day rechallenge” could likely be underestimated in this manuscript.^47^

Interestingly, a particular kind of “same-day rechallenge”, also known as “restart protocol”, was helpful, in the hands of expert centers with allergy-dedicated spaces, to successfully ensure tolerance to chemotherapy and biologics immediately after a positive DPT.^2^^,^^4^^,^^6^^,^^36^ This phenomenon of temporary tolerance to a culprit drug within minutes or hours after a DHR is known as post-anaphylaxis mast cell anergy or “empty mast cell syndrome”, attributed to the depletion of mast cell granules or temporary dominance of inhibitory signalling pathways.^48^ This phenomenon has been reported for hymenoptera venom, perioperative reactions, non-steroidal anti-inflammatory drugs (NSAIDs), biologics, and chemotherapy.^4^^,^^46^, ^47^, ^48^, ^49^

In an article by Madrigal-Burgaleta et al, after performing 341 DPTs with chemotherapy and biologics over 7 years, 112 DPTs were positive (ie, the patients experienced a reaction in a controlled environment).^2^ To ensure that patients received the medication prescribed by their referring physician despite experiencing a reaction during the DPT, these authors devised a same-day rechallenge, which they refer to as “restart protocol”. Patients with a positive DPT were given the option of initiating the “restart protocol” within minutes after controlling the reaction to the DPT.

In this article, the “restart protocol” was used in 13 patients who had a positive DPT with intravenous biologics, and it was successful in all patients regardless of the severity of their reactions during DPT (5 patients, grade 1; 7 patients, grade 2; 1 patient, grade 3; as per Brown's severity grading system).^2^ In that same study, this “restart protocol” was used in patients reacting to platinum drugs (27 patients, grade 1; 16 patients, grade 2; 7 patients, grade 3; as per Brown's severity grading system), taxanes (11 patients, grade 1; 14 patients, grade 2; 7 patients, grade 3; as per Brown's severity grading system), and miscellaneous chemotherapeutic agents (5 patients, grade 1; 10 patients, grade 2; 2 patients, grade 3; as per Brown's severity grading system).^2^ In summary, over these 7 years, the “restart protocol” was successful in all 112 patients reacting to DPT, except for 2 patients.^2^ These results give the “restart protocol” a success rate of 98%.^2^^,^^4^ Regarding these 2 patients who did not tolerate the “restart protocol”, 1 taxane-reactive patient experienced a grade 3 reaction (Brown's classification) during a DPT kept experiencing dyspnoea and erythema on “restart protocol”, and 1 oxaliplatin-reactive patient suffered fever-chills and back pain during the DPT kept experiencing these symptoms on “restart protocol”.^2^^,^.

However, it is of crucial importance to understand that the objective of these “same-day rechallenges”, or “restart protocol” after a positive DPT, is preventing the patient from missing treatment sessions, and by no means does this technique have a diagnostic value because tolerance to a “same-day rechallenge” does not ensure tolerance of the next cycle.^6^^,^^50^ Therefore, after a positive DPT, patient would need to be considered for RDD regardless of their tolerance to the “restart protocol”.^1^

### Rapid drug desensitization

The incidence of DHRs to intravenous drugs, such as chemotherapeutics, biologics, or antibiotics, increases as the therapeutic arsenal evolves and patient survival rates improve, allowing more patients to be at risk of allergic sensitization due to longer periods of exposure to these treatments.^2^^,^^51^, ^52^, ^53^, ^54^, ^55^, ^56^ DHRs can be severe, including anaphylaxis and even death. DHRs affect the prognosis and quality of life of many patients by preventing the use of first-choice therapies and forcing patients to change to a second-choice drug before they become refractory to treatment.^2^^,^^56^^,^^57^

Drug desensitization is the cornerstone of the therapeutic approach to DHRs.^1^^,^^5^ The term RDD is applied to drug desensitization processes designed to induce desensitization in a few hours.^19^^,^^56^ RDD usually refers to intravenous procedures; however, it has been used for other routes (eg, subcutaneous or oral).^19^

RDD is indicated in patients with a confirmed hypersensitivity.^58^ In situations where the drug hypersensitivity has not been confirmed and where the time and situation permits testing and likely delabeling, delabeling is the preferred strategy to desensitization. However, in situations where the drug hypersensitivity is not confirmed, and desensitization is done because of time pressure or necessity, the patient should be referred for allergy testing as soon as possible following completion of treatment.

RDD is usually considered only when there is no alternative drug, but it is widely accepted that it should also be considered when the culprit drug is more effective or is associated with fewer side effects.^21^^,^^56^^,^^1^, ^5^, ^41^, ^42^^,^^59^ For example, recent data show how RDD does not affect the efficacy of oxaliplatin; thus, colorectal cancer patients reacting to the first line of therapy with oxaliplatin should consider RDD to oxaliplatin, even if other lines of treatment are available (because alternative lines of treatment are usually less effective or more toxic).^7^ In addition, overall survival was better in patients with ovarian cancer when they could continue their therapy with platins instead of abandoning them after a reaction.^60^

The confirmation of an indication is vital and must be documented to avoid mistakes that might affect patient safety and to ensure empowered patient-centered decisions as well as a real multidisciplinary coordination. Madrigal-Burgaleta et al solved this by using 2 different informed consents for RDD: one form is signed by the allergists and the patient, and it explains the procedures and its risks; whereas the other form is signed by the referring physician and the patient, and it confirms that there is an indication for treatment with the culprit drug as a first-choice drug despite a DHRs, and that the patient will be referred to the allergy department for this.^2^

RDD is progressively becoming a standard of care. Recent data are extinguishing concerns over costs, managerial difficulties, and queries on drug efficacy under RDD (triggered by the fact that RDD administration patterns may differ from standard infusions). Indeed, RDD has shown to be a cost-effective technique that offers reactive patients comparable survival outcomes to those of non-allergic patients.^7^^,^^61^

Drug desensitization exploits a feature of mast cell physiology. Slow increments in the concentration of a ligand that binds to certain mast cell surface receptors fail to trigger mast cell activation and the systemic release of the mast cell mediators responsible for anaphylaxis.^56^^,^^62^ *In vitro* and *in vivo* models demonstrated that RDD inhibits several key processes of mast cell activation: extracellular calcium influx, degranulation and release of preformed mediators, newly generated lipid mediators, and cytokine and chemokine production.^62^

Based on these data, some authors argue that RDD should only be considered for mast cell-dependent I-DHRs and not for NI-DHRs where T cells may be involved. However, the mechanisms of RDD have not been completely elucidated, and preliminary data suggest that drug-specific T cell response seems to be affected by drug desensitization due to the expansion of T regulatory cells producing IL-10 and IL-35.^63^ Thus, various authors consider that some mild NI-DHRs could potentially benefit from RDD.^1^^,^^2^^,^^19^^,^^56^^,^^62^ However, some members of the Reviewing Panel of this manuscript advised caution, as mechanistic data from RDD on large series of patients with well-characterized NI-DHRs are lacking. We will discuss this controversial issue in Section 11. Nevertheless, in this manuscript, we will not limit the use of the term “desensitization” to I-DHRs for the sake of homogeneity with recent consensus documents.^19^^,^^64^

It is generally agreed that certain reaction types are unlikely to benefit from RDD or put the patient at an unacceptably high risk of a potentially irreversible life-threatening reaction. Therefore, these reaction types constitute a contraindication for RDD. These include immunocytotoxic reactions such as type II reactions or oxaliplatin immune-induced syndrome (OIIS), vasculitis, serum sickness-like (type III reactions), or SCARs (such as Stevens-Johnson syndrome, toxic epidermal necrolysis, acute generalized exanthematous pustulosis, drug-induced hypersensitivity syndrome or drug reaction -rash- with eosinophilia and systemic symptoms).^21^^,^^56^ Of note, OISS may clinically resemble CRR, so these syndromes need to be carefully differentiated.^65^ Unfortunately, these reactions will generally benefit from avoidance, as both DPT and RDD can trigger a potentially lethal DHR in those patients. Again, however, there are indeed grey areas due to a lack of good-quality data.

As discussed, RDD has been mainly studied for use on type I-DHRs (acute onset reactions involving the release of preformed mediators of mast cells and basophils). However, as we mentioned, controversy exists as to whether desensitization can be successfully and safely used for different DHRs, especially CRR and specific nonsevere type IV reactions.^1^^,^^19^^,^^23^^,^^56^^,^^64^ Guidelines usually contraindicate the use of desensitization in non-mild type IV reactions.^19^^,^^58^ Controversially, there is some literature on desensitization for unascertained type IV DHRs, including DRESS-like DHRs.^66^ Nevertheless, data are scarce, and desensitization outside of type I DHRs should be limited to highly specialized centers or research.

As we will explain in detail in Section 6, RDD protocols use doubling doses in a step-wise manner every 15–30 minutes to administer the agent after starting at a concentration under the threshold for reactivity.^1^^,^^56^^,^^62^^,^^67^^,^^68^ Exposing mast cells or basophils to the same ligand at a higher 10-fold concentration increase over the same time period may trigger mediator release and potentially another episode of anaphylaxis.^67^^,^^69^^,^^70^ Similarly, shortening the period between the steps will not help with reaching a state of desensitization.^71^

Over 20 years ago, Shalit et al demonstrated *in vitro* how the tolerance induced by RDD is antigen-specific and temporary.^72^^,^^73^ More recent studies using *in vitro* models for RDD have confirmed these findings.^62^^,^^68^

Regarding antigen-specificity, patients allergic to two different drugs in real-life need desensitization to each drug separately.^2^

As for the temporary nature of RDD, these findings are compatible with real-life experience, where chemotherapy-allergic patients receiving chemotherapy every 2–3 weeks need desensitization for every administration.^2^, ^298^ In contrast, antibiotic-allergic patients will usually receive continuous exposure to an antibiotic for a continuous course and, generally, will only need desensitization for the first dose of the course.^2^^,^^67^^,^^298^

The exact duration of the desensitized state is unknown and might be influenced by multiple factors. Shalit et al found that desensitized mast cells can be activated after 24 hours^72^^,^^73^ Sancho-Serra et al found that the desensitized state lasted at least 4 hours, but the authors could not test this beyond 4 hours due to technical difficulties.^68^ High-quality supporting data are lacking, but the consensus is that the desensitized state approximately lasts from 2 to 5 half-lives of the culprit drug.^19^^,^^67^

RDD has been successfully used for countless drugs, in patients of all ages with different conditions, in patients presenting with severe reactions like anaphylaxis, pregnant women and patients with underlying conditions such as mastocytosis.^2^^,^^8^^,^^10^^,^^12^^,^^19^^,^^55^^,^^56^^,^^67^ Nonetheless, as we will explore in Section 2, similarly to other highly specialized procedures in medicine, RDD is far more than using an administration protocol or a recipe that anyone can follow^298^. Indeed, RDD reaches its optimal efficacy and safety outcomes in combination with sophisticated risk management strategies and when in the hands of multidisciplinary teams led by expert allergists with access to dedicated spaces and the necessary resources.^5^

## Section 2: Well-grounded know-how: “Laying the foundations for the new silk road” (Alvarez-Cuesta & Madrigal-Burgaleta)

### “Excellence is not a destination, but a path.”

Barely a handful of groups led by experts in drug allergy have carried out the most significant studies on both intravenous RDD or intravenous delabeling with various drugs, mostly on chemotherapy and biologics.^2^^,^^5^^,^^61^^,^^74^, ^75^, ^76^, ^77^, ^78^, ^79^, ^80^ In addition, a limited number of groups have provided immense amounts of data on delabeling with other drugs when there is an acute need for them, mostly penicillins.^81^, ^82^, ^83^, ^84^, ^85^, ^86^ In this manuscript, a selection of these leading groups will share their different perspectives on delivering patient care based on their experience with all drug classes. We will find there is more than 1 road to excellence, and we will analyze the common tools that make these groups successful.

### The overall framework for action

While an individual RDD procedure may be straightforward for the expert, developing the infrastructure to perform more widescale RDD procedures is complex. Those teams willing to implement this technique more broadly and efficiently may need to significantly redesign the way they deliver care. Disorganization or system errors can cause harm to patients; thus, careful planning will reduce the risk for patients in this regard. Furthermore, being prepared for a potential managerial crisis is of the essence.^87^

These 3 questions are key: i) How do we identify the potential for innovation and improvement? ii) Which are the best decisions to strengthen the department and how to implement them? iii) How do we prepare ourselves for the recovery of a managerial crisis so that we can emerge from it as a leading competitive group and even as a benchmark standard?

In this section, we will provide tools that were effective in redesigning care at RCUH. We will start by establishing the framework for the team involved in the project, which should facilitate a successful and seamless process:

A) To define the fundamental parameters of the project (eg, priorities, red lines, or strategies).
B) To share the workload among the team by creating a handful of thematic working groups to advise on critical areas of expertise (eg, adult allergy, pediatric allergy, or translational laboratory, among others).
C) To define the rules by which the working groups will operate when redesigning care (eg, how many working groups, election or appointment process, or meetings). A clear leadership or arbitrating commission (eg, the clinical lead or a commission of working group leads). In our experience, precise regulation will make the process seamless and prevent friction and delays.
D) To understand that inaction will be harmful to the patient.

### Ramon y Cajal University Hospital's 7 fundamental pillars for redesigning care to implement rapid drug desensization

1) **Think globally, but act locally.**^88^ Finding inspiration on functioning international models is vital, but we need to listen to local ideas from the local working groups, as only they can provide local insight.
2) **Patient centeredness.** Continuous quality improvement, personalized medicine (eg, flexible, tailored RDD protocols), patient safety, patient comfort, patient education, and patient satisfaction must be at the heart of the redesigning project.
3) **To adapt the department to high-complexity and high-risk patients.** It will require specific resources for innovation (from hiring new expert staff to creating new areas or training) and the constant analysis of every decision to prevent guesswork or lack of planning.
4) **Team empowerment.** Multidisciplinary teams (MDTs) led by experts and focused on a similar objective are of organizational help. They can create solid, focused and motivated teams with a manageable workload (eg, one team for in-patient antibiotic allergy, another for chemotherapy allergy, and many others depending on local variation). Positive collaboration with other departments is of the essence.
5) **To create a dedicated Technical Area for Diagnostic and Therapeutic Procedures in Allergy (Technical Area).** This Technical Area should be the driving force of the Allergy Department as a whole, should be adapted to the local needs (which will be different for an allergy department focused on food allergy in children or one focused on asthma in adults) and should be flexible enough to be able to adapt to foreseeable future demands.^89^ In addition, we envisioned RCUH's Technical Area to comply with a “safety first” policy, which will involve devoting resources to risk management strategies and quality control.^2^^,^^89^ Supplementary text 1 expands on the design of the RCUH Technical Area.
6) **To establish measurable indicators of efficacy, efficiency, safety and quality for the Department.**
7) **To live up to a constant obligation towards excellence and continuous quality improvement.**

### The shared unique selling proposition among leading groups in rapid drug desensitization

In a nutshell, MDTs led by experts in drug allergy, with local strategies for risk-assessment and personalization, and access to dedicated spaces within the allergy department seem to be the shared unique selling proposition of the leading teams on RDD.^5^

The critical role of the allergist in providing access to optimal care for patients with drug allergies is incontestable.^27^^,^^90^ Offering high-quality care in drug allergy without involving an allergy department becomes cumbersome.^24^^,^^76^^,^^91^ However, the allergist is unlikely to work alone. The increasing complexity of care in drug allergy calls for MDTs.^2^^,^^24^^,^^61^^,^^78^ Consequently, leading groups in drug allergy encourage allergists to become proactive in their role as leaders within their organizations.^5^^,^^27^

These MDTs are the most effective when services have access to proper physical resources, including dedicated spaces^2^, ^92^. However, we would recommend avoiding different dedicated areas scattered around the hospital. For example, RCUH has one specific Technical Area for all diagnostic and therapeutic procedures in Allergy (see supplementary text 1).^2^^,^^89^A robust Technical Area integrated within the allergy department can efficiently centralize all the space, staff, and resources for all the clinical procedures of the department, including intravenous desensitization and delabeling. This more comprehensive and flexible central hub with large areas also allows for the other fundamental MDT needs (e.g, office space, hot-desk space for visiting specialists, room to conduct meetings and teaching, or even storage space for studies documentation).^2^^,^^93^^,^^94^

### Allergy departments should lead rapid drug desensitization teams

RDD shows better outcomes in the hands of experts in drug allergy.^5^ Therefore, our positioning is that all MDTs offering RDD and delabeling should be led by an experienced allergy department, and institutions should provide specific funding to aid the allergy department in this role. If this is impossible, the MDT should involve at least an expert allergist in all the decision-making processes of the MDT, from the more general issues regarding service provision to day-to-day patient management.^21^^,^^90^

We strongly discourage non-allergists or non-experts (with little understanding of DHRs and no experience in drug allergy) from managing patients in need of RDD, even when they used published RDD protocols. RDD protocols alone do not suffice for a successful RDD strategy.^2^^,^^4^^,^^5^^,^^56^^,^^298^ “*RDD is not only a protocol for drug administration, but it is a complex and multidisciplinary system*”.^2^ For example, this multidisciplinary approach to RDD should involve not only the right protocols, but also validated diagnostic pathways, dedicated spaces, trained and experienced staff, and even institutional preventative measures for early detection of reactive patients and expedited referral to the allergy department, thus preventing suboptimal management.^4^, ^5^, ^298^

The indiscriminate use of allergy-specific techniques (such as DPT and RDD) by non-allergists is frequently recommended against, as it can negatively impact patients in the form of misdiagnosis, unnecessary risks (including death), or surprisingly low rates of successful RDDs.^2^^,^^5^^,^^21^^,^^56^ For example, failure to refer patients to an allergy department after a reaction can incur false labels of allergy to the chemotherapeutic drug by overlooking other culprit drugs, as explained by Urena-Tavera et al.^10^

Studies from Massachusetts General Hospital (MGH) have observed how DPT (or drug challenges) to chemotherapy and biologics conducted by non-allergists expose patients to unnecessary risks. Additionally, it prevents them from continuing with their first-line therapy as very low percentages of patients were given the option of being referred to the allergy department to consider RDD (eg, only 4% of rituximab-reactive patients were referred to the allergist in their studies).^5^^,^^47^^,^^95^

Furthermore, the reader will find articles published by non-allergists where patients are blindly put at unnecessary risk of a reaction to platins on reexposure; for example, lacking the appropriate risk assessment (which should include ST) and failing to offer patients the option of RDD.^60^^,^^96^ More so, some of these reports even describe deaths both for delabeling attempts and for RDD attempts.^97^^,^^98^

The lack of understanding of the mechanisms of allergy and RDD, the lack of proficiency/experience in allergy-specific techniques, and the lack of specific resources and dedicated spaces can put patients at unnecessary risks, as we just discussed. Thus, allergy departments must always encourage close collaboration. There are remarkable but rare exceptions of commendable teams who have created standardized approaches to RDD without the leadership of an allergy department and yet have managed to help many patients succesfully.^30^^,^^98^^,^^99^ However, not involving the allergy department in these teams can potentially affect healthcare quality or perpetuate inequalities. For example, it can perpetuate the lack of access to allergy-specific techniques (eg, ST, specific IgE, or basophil activation test), to clinical expertise in overall drug allergy and risk-assessment (eg, adequate endophenotyping), or to additional therapeutic options (eg, omalizumab as a premedication), which would be widely available to allergy-led teams.^2^^,^^5^^,^^61^^,^^100^

Furthermore, allergy-led RDD teams with access to a more comprehensive dedicated Technical Area are arguably more cost-effective, as the same staff can offer RDD and delabeling to different departments of the hospital with the same resources and in the same space, and for a broader range of patients and drug reactions (including chemotherapy, biologics, antibiotics, antivirals, insulins, heparins, and many other drugs).^2^^,^^12^^,^^55^^,^^61^ In contrast, non-allergy-led teams would have a specific focus and would offer delabeling and RDD only to a subset of patients with drug reactions to only one type of drug.^30^^,^^98^^,^^99^ It would be arguably more reasonable to focus institutional resources on supporting the allergy Technical Area to provide service to all the different departments.

Safety should also be an essential part of the discussion because allergy-led Technical Areas will have access to comprehensive risk management strategies (see supplementary text 1). However, areas led by non-allergists can be potentially dangerous for patients, as resources may be insufficient, and staff might not have specific training or may even be unaware of the procedures taking place.^2^^,^^4^^,^^5^^,^^95^ In addition, non-allergists will find it virtually impossible to reach a critical number of patients to be able to consider themselves experts in drug allergy. Indeed, the expertise that the allergist can provide in diagnosing and treating anaphylaxis is essential for patient safety. Groups led by expert allergists have access to trained staff who feel confident with sophisticated strategies to manage any DHRs.^2^ On the other hand, our colleagues from MGH have identified how untrained non-allergist staff fails to identify and treat anaphylaxis adequately even when consciously subjecting patients to high-risk allergy-specific procedures like intravenous DPT,^47^^,^^95^ which is consistent with the published experience in anaphylaxis whether in the community or the hospital.^101^, ^102^, ^103^, ^104^, ^105^, ^106^

### Creating a multidisciplinary team

The MDT should be a central part of the drug allergy pathway of care, considering plenty of evidence on the benefits of MDTs and MDT Meetings as valuable mechanisms for coordinating care in patients with complex needs.^93^ Correspondingly, as clinical care becomes increasingly challenging, translational research has also progressively adopted multidisciplinary approaches, and collaborative work has shown to have a higher scientific impact.^107^

When successful, MDTs are a rapid single point of access to specialist expertise, enabling comprehensive and seamless care services to be delivered.^93^ Several common factors increase the chances of a thriving MDT, even if these MDTs are part of a unique organizational environment.^92^^,^^108^ Therefore, the allergist needs to carefully plan for creating the MDT, ensuring clear allergy leadership, a strong shared vision, good communication, team skills, and patient centeredness.^92^^,^^93^^,^^107^, ^108^, ^109^

The MDT should include the members of the allergy department that will be involved in patient care (eg, doctors, nurses, trainees, researchers, laboratory staff, allied health professions, or administrative staff). However, a meticulous strategy should include an initial outreach campaign beyond the allergy department (talks, meetings, or team-building activities, among others) focused on the potential prospective members of the MDT. Ideally, there should be one person of contact from each essential department involved in the MDT. The necessary members of the MDT may depend on the team (eg, a team for chemotherapy allergy or another for inpatient delabeling would involve different professionals), and it might vary locally. However, the MDT would likely include, for example, Pharmacy, Oncology (or other involved departments like Infectious Diseases, Haematology, or Cardiology, depending on the type of team that needs to be created), critical care (to coordinate high-risk patients and management of severe cases), and the appropriate high-level managers.

It will be fundamental to identify potential partners, to assert allergy leadership, and to plant the seeds for a shared vision; whereas, continuous training, individual rewards/opportunities, appropriate processes, flexibility, a solid institutional image as an MDT service, and monitoring of quality and outcomes of care will be some critical factors for consolidation and smooth continuous strengthening.^92^^,^^93^^,^^107^, ^108^, ^109^

### Creating a technical area for diagnostic and therapeutic procedures in allergy

We envisioned the allergy department as a whole, not as a department fragmented in different “units”.

MDTs may be organized in various "programs" specialized in different conditions. However, all MDTs will have access to the resources and installations of the flexible allergy Technical Area, with spaces organized by levels or risk stratification and not divided by units or conditions. This centralized Technical Area allows for efficient management of all diagnostic and therapeutic techniques of the allergy department, optimizing resources and staff.

Allergy Technical Areas for diagnostic and therapeutic procedures (eg, food challenges, drug challenges, skin testing, lung function testing, immunotherapy, or desensitization) should ideally be located in a hospital setting with easy access to critical care for safety reasons.^111^

Please, see supplementary text 1 for more information on the practical, real-life example of the RCUH's Technical Area for Diagnostic and Therapeutic Procedures in Allergy.

### Safety first, zero harm, patient-centeredness, and staff protection

Patient safety needs to be a core value of the care continuum. Breakdowns in patient safety are a leading cause of both death and incidents of nonfatal harm to patients.^110^ Although targeting “zero harm” is a controversial approach in drug allergy because both sides of the decision-making spectrum (avoiding life-saving drugs for fear of another reaction or attempting re-introduction) are potentially harmful.

The approach to risk in drug allergy has been remarkably reactive, focusing on the type of reaction, patient characteristics, and provision of resources.^111^ A shift from reactivity to proactivity is essential to all aspects of a total systems approach to safety.^110^ One of the main issues is a lack of definition for “zero harm”,^110^ and allergists have failed to explore this topic thoroughly.

Emotional, psychological, and socio-behavioral harm are as real, impactful, and preventable as physical harm.^110^ There is a clear potential for physical harm during the management of drug allergy, the most obvious one being the inherent non-zero risk of anaphylaxis during drug challenge or RDD.^2^^,^^61^ On the other hand, there are inherent risks linked to abandoning the first-choice therapy for fear of inducing a reaction in a patient with an unstudied label of allergy.^7^^,^^80^ The different types of harm in drug allergy have been surprisingly understudied.

There are data to say that patients suffer from psychological harm after anaphylaxis triggered by a drug allergy,^112^ but beyond that, our experience is limited to validating a specific quality-of-life questionnaire for drug allergy in different populations.^113^, ^114^, ^115^, ^116^ Data show that a drug challenge can positively impact the quality of life of the patient.^116^ On the other hand, some authors wonder whether a drug challenge could have a negative psychological impact.^50^ There is virtually no mention of other factors. For instance, whether patients who have not been delabeled and are travelling far away for repeated unnecessary RDDs might be put indirectly at risk of traffic accidents, financial risk by expensive travel fares, or even exposure to infection risks such as COVID-19. Another example is the lack of data on whether patients reacting to chemotherapy are more comfortable under the care of their outpatient oncology setting or the specialized care of the allergist.

Even if previous experiences in RDD have addressed the need for patient-centredness in complex care, the field of drug allergy is unacceptably behind in this.^2^^,^^5^ However, interestingly, patient-centeredness is one of the key features for quality in healthcare.^117^ Gandhi et al analyze the 4 elements that make a systems-focused approach to safety robust. One of these elements is engaging patients in the codesign, improvement, and delivery of care with a shared decision-making process.^110^

The other 3 interdependent elements in the study by Gandhi et al were, namely: effectively managing change by tending to the psychology of change; creating and sustaining a just culture of safety; and developing and leveraging an optimal learning system (continuous learning leveraged for continuous improvement, using processes such as Root Cause Analysis and Actions).^110^ These areas are underdeveloped in the field of drug allergy, and some future efforts need to be directed towards filling this gap of knowledge.

Staff safety should also be paramount. Even if this should be an essential comment on articles focused on hazardous drug handling, there are only brief mentions on staff safety in drug allergy.^2^^,^^3^, ^300^ It should include ensuring respectful team dynamics where any member of staff (not only the leading medical staff) can express their opinion on patient management.^110^

### Think global, act local: Risk-assessment, continuous quality improvement, and lifelong learning

One of the critical features that account for much of the success of the different leading groups in intravenous RDD is their approach to risk.^5^ Risk assessment may vary locally, but it is cemented on 3 fundamental pillars, namely: (i) Access to appropriate facilities and specific resources (eg, the allergy Technical Area); (ii) Access to expert personnel capable of appropriate patient selection and management (eg, MDTs); and, (iii) Locally-designed risk-management strategies, which are open to tailored plans based on precision medicine (eg, personalized approach, phenotyping and endotyping).^5^, ^41^, ^42^, ^298^

Even if the general approach remains very similar, different groups might have locally unique approaches to drug allergy management, including risk assessment. Berwick et al, Institute of Health Improvement, explained how “*a properly redesigned care system requires detailed local, social, and technical adaptations to leverage contextual differences. Innovations that work well in one setting often do not work in another without substantial modification*”.^118^ Local variation is the main reason why leading groups on intravenous RDD constantly recommend other groups to validate their procedures for their local system.^5^

Despite differences in local approaches, risk-management strategies should be widely comprehensive. Risk assessment in drug allergy has not been thoroughly addressed in the literature, and current risk management strategies only focus on patient characteristics and providing classical institutional safety nets such as acute medicine support.^111^ However, there is much we can learn from risk management in the world of finances. An article by Kaplan et al presented a helpful classification of different types of risks and how to tackle them, namely, preventable risks (arising from within the organization), external risks (uncontrollable and to some extent unpredictable), and strategy risks (those taken consciously and for positive reasons).^119^ The mainstream risk assessment strategies for clinicians focus primarily on trying to minimize the impact of external risks, which can come, for example, in the form of patient comorbidities or drug toxicity.

However, other types of risks are usually left unmentioned. For example, preventable risks can be tackled by quality improvement, rules, standard operating procedures (SOPs), or constant monitoring. Preventable risks should be eliminated because they do not offer any benefit and can cause harm. These can be as simple as a member of the team not turning up to MDT meetings. Strategic risks are consciously accepted because the MDT believes taking the chance will bring significant benefits (eg, recent drug allergy delabeling strategies). Strategic risks do not respond to a classical approach to risk (such as checklists or SOPs). More complex techniques are needed (such as worst-case scenario planning, tail-risk stress tests, or wargaming, which involve analysis or simulation designed to determine the ability of a process to deal with a crisis —from a severe reaction to a chemotherapy leak, or a pandemic), all of which require support from a strong MDT with access to constant learning.^119^

The concept of continuous quality improvement is yet to reach drug allergy. Nevertheless, this discipline has shown how beneficial it is for all parties involved in healthcare (from the patient to the funding organization) to have a clear framework to continuously improve value by reducing cost and improving quality.^120^ There are many ways to approach quality improvement, and indeed, training in this field will be an essential part of the MDT. In fact, as an example, a recent article by Mate et al^120^ applied Lean management tools (an approach to eliminating waste and optimizing workflows) at an inpatient respiratory ward in a mid-sized hospital in NHS Scotland. Their continuous value management helped reduce costs and improve quality by standardizing their care model, using map processing, understanding variation, and redesigning processes. Indeed, they optimized efficiency by tracking the expenses, reducing waste, and improving performance. Interestingly, except for one article that explored this issue on carboplatin-reactive patients,^61^ there are virtually no studies on cost-utility, and certainly none on quality improvement in intravenous RDD. But, as we can conclude from Mate et al, only by measuring outcomes and checking continuously can we further improve patient care quality.^120^

In conclusion, allergists thinking about starting a safe, effective, and reliable MDT and Technical Area will need specific training on quality and patient safety. Engaging in continuous quality improvement training will inevitably require devoting resources and creating and supporting lifelong learning for all the staff.^121^

## Section 3: Delabeling allergy to chemotherapy in adult patients (Alvarez-Cuesta & Madrigal-Burgaleta)

### Diagnostic *in vivo* risk-biomarkers with chemotherapy

Immediate readings for skin prick testing (SPT) and intradermal testing (IDT) are the 2 types of ST most widely used for the study of I-DHRs to chemotherapy, and these have a vital role in risk-assessment.^6^ Even if some groups find a low sensitivity for ST,^2^^,^^6^ they are still a fundamental tool because, when they are positive, they can accurately identify an IgE-mediated endotype.^6^

NI-DHRs could potentially benefit from using other techniques, such as the delayed reading of IDT, patch testing, or photo patch testing, but there are virtually no data on using these tests with chemotherapy agents.^6^

ST for drug allergy is usually a common and invaluable technique in allergy departments. However, the experience of ST with chemotherapy is rather heterogeneous, as different groups use a different methodology, different interpretation criteria, and their own set of non-irritant concentrations (trying to find a difficult balance between prioritizing sensitivity or specificity).^6^ In addition, it may be difficult for some departments to obtain material for testing, often due to special requirements for handling chemotherapy, which is one of the main reasons why allergy departments need to be well-prepared before managing these patients. Section 2 and supplementary text 1 offer further insights on this topic.

Moreover, many factors make the interpretation of ST with chemotherapy difficult, even for highly specialized staff. For instance, the presence of different endophenotypes of reactions might affect ST reliability: patients with a cytokine release syndrome endophenotype can have negative ST,^22^ and some patients with a history of an “old DHR” with platins (described as >6 months) can show negative STs (and even negative DPT) only to become “positive converters” on the next administration.^2^^,^^4^^,^^100^^,^^122^^,^^123^ In consequence, there is a clear need for studies on the optimal approach to ST with chemotherapy.^6^

### Diagnostic *in vitro* risk-biomarkers with chemotherapy

Widely available tests such as tryptase determination during the acute phase and comparison with baseline levels can help better endophenotype type I hypersensitivity reactions.^4^^,^^6^^,^^22^^,^^124^, ^125^, ^126^ Tryptase has been used to identify patients at risk of mast cell clonality and systemic mastocytosis, especially when used in combination with the helpful score validated by REMA (an acronym for the Spanish Mastocytosis Network).^8^^,^^127^ Inflammatory biomarkers such as IL-6 is a novel tool to identify cytokine release syndrome-like reactions,^22^^,^^26^ and a full blood count might aid in identifying type II hypersensitivity reactions (especially with oxaliplatin).^6^^,^^22^ Total IgE is not usually a reliable biomarker of allergy. However, it was found to be a modest predictor of a final positive diagnosis of allergy in platin-reactive patients, with an RRR of 1.46 (95% confidence interval, 1.00–2.40).^2^ In that population, patients with a confirmed diagnosis of allergy to platins had a mean total IgE of 421 UI/L, whereas patients with a negative DPT (ie, an allergy was ruled out) had a mean total IgE of 117 UI/L (unpublished data from the same article).^2^ Interestingly, another group recently found that a total IgE greater than 100 UI/L was an independent risk factor for breakthrough reactions with platins.^128^ The use of these “more general” biomarkers seems promising but needs to be further studied in different populations.

More specific tests such as the determination of specific IgE are only available for a handful of drugs, still limited to research, and not commercially available. Taxane-specific IgE has been successfully used at least in one case report,^129^ but virtually all published data on chemotherapy-specific IgE is focused on platins.^3^^,^^4^^,^^130^^,^^131^ These studies showed how specific IgE helps to identify an IgE-mediated endotype and may be of value when studying cross-reactivity. But, they also found unresolved issues, such as finding false positives, determining optimal cut-off points, and using adequate criterion standards for validation.^6^ In addition, most of these studies involved small series of patients. However, one group attempted to validate chemotherapy-specific IgE in a prospective cohort study with a large population of 74 oxaliplatin-reactive patients who followed a diagnostic protocol including systematic DPT.^4^ As a methodological strength, DPT was performed blindly before having access to the result of the oxaliplatin-specific IgE.^4^ The authors found that oxaliplatin-specific IgE showed reasonable specificity and poor sensitivity, and concluded that a negative result will still require DPT to reach a diagnosis.^6^^,^^34^ This is not an uncommon scenario with specific IgE for other drugs.^34^ However, the poor sensitivity for oxaliplatin-specific IgE in this study could potentially be explained by the presence of non-IgE-dependant cytokine release syndrome-like reactions in the analysis, an endophenotype that has only been recently described.^4^,^22^

The basophil activation test (BAT) is still challenging to implement in clinical practice, and standardization/validation is an unmet need. However, there have been promising results using BAT as a diagnostic tool and a risk marker for the severity of reactions during RDD.^6^^,^^11^^,^^132^, ^133^, ^134^

Novel tools such as nanoallergen platforms have also shown promising results but need further studies.^135^ In addition, discoveries on key cytokines involved in anaphylaxis, neutrophils as potential cellular actors in certain types of anaphylaxis, platelet activation factor as a critical mediator of anaphylaxis, and further insight on the mechanisms of no-IgE-dependent anaphylaxis will hopefully provide us with a broader range of biomarkers in the future.^29^^,^^136^, ^137^, ^138^

### Drug challenge or drug provocation testing with chemotherapy

Section 1 dealt with the general concepts of DPT. However, we must bear in mind specific considerations when using DPT in chemotherapy-reactive patients. For instance, it must be designed for the patient not to miss a treatment session. For this reason, specific strategies should be put into place to ensure that even patients with a positive DPT receive all their medication; as explained in Section 1, RCUH has described this as the “restart protocol”.^4^^,^^6^

The financial and staffing expenditure linked to the high-risk technique of DPT with chemotherapy can explain why real-life data are rather scarce.^5^ RCUH published the first reported data on systematic DPT with chemotherapy agents.^3^ Two original articles by this same group followed this initial experience, and they featured the largest reported series of systematic DPTs with chemotherapy.^2^^,^^4^ In one of these studies, Madrigal-Burgaleta et al^2^ reported data from over 300 DPTs in patients with an unequivocal clinical history of a reaction with chemotherapy and biologics. Strikingly, 67% (229/341) of all performed DPTs were negative, and only 33% (112/341) were positive. Regarding safety, only 5% (17/341) of all patients undergoing DPT experienced a severe reaction as per Brown's classification. However, this comprised 15% of all positive DPTs (17/112) who suffered a severe reaction over 7 years, with 4 patients presenting with hemodynamic changes (grade IV anaphylactic shock reaction according to the criteria of RCUH).^2^^,^^5^ Further results from these studies are shown in Table 1.

**Table 1:** Data on drug provocation testing (DPT) with chemotherapy agents from the large cohort of patients of the RCUH (Ramon y Cajal University Hospital), Madrid, Spain

Alvarez-Cuesta E, et al. Allergy. 2015				Madrigal-Burgaleta R, et al. JACI: In Practice. 2019			
**Results from all chemotherapy-reactive patients referred over three years (n = 156)**				**Results from all chemotherapy-reactive referred patients in seven years (n = 515)**			

**DPT Result**	**Taxanes** **n = 43**	**Platins** **n = 95**	**Other** **n = 18**	**DPT Result**	**Taxanes** **n = 135**	**Platins** **n = 188**	**Other** **n = 97**

Negative DPT 58/156 (37%)	17/43 (40%)	31/95 (33%)	10/18 (56%)	Negative DPT 229/515 (45%)	70/135 (52%)	43/188 (23%)	69/97 (71%)
Positive DPT 33/156 (21%)	9/43 (21%)	20/95 (21%)	4/18 (22%)	Positive DPT 112/515 (22%)	32/135 (24%)	50/188 (27%)	17/97 (18%)
DPT not undergone 65/156 (42%)	17/43 (40%)	44/95 (46%	4/18 (22%)	DPT not undergone 174/515 (34%)	33/135 (24%)	95/188 (51%)	11/97 (12%)
**Results only from the patients who underwent DPT with chemotherapy (n = 91)**				**Results only from the patients undergoing DPT with chemotherapy (n = 341)**			
**DPT Results**	**Taxanes** **n = 26**	**Platins** **n = 51**	**Other** **n = 14**	**DPT Result**	**Taxanes** **n = 102**	**Platins** **n = 93**	**Other** **n = 86**
Negative DPT 58/91 (64%)	17/26 (65%)	31/51 (61%)	10/14 (71%)	Negative DPT 229/341 (67%)	70/102 (69%)	43/93 (46%)	69/86 (80%)
Positive DPT 33/91 (36%)	9/26 (35%)	20/51 (39%	4/14 (29%)	Positive DPT 112/341 (33%)	32/102 (31%)	50/93 (54%)	17/86 (20%)
**Safety results from the patients with a positive DPT to chemotherapy**				**Safety results from the patients with a positive DPT with chemotherapy (n = 112)**			
**Severity Brown classification**	**Taxanes** **n = 9**	**Platins** **n = 20**	**Other** **n = 4**	**Severity Brown classification**	**Taxanes** **n = 32 (%)**	**Platins** **n = 50 (%)**	**Other** **n = 17 (%)**
Grade 1 16/33 (48%)	5/9 (56%)	10/20 (50%)	1/4 (25%)	Grade 1 48/112 (43%)	11/32 (34%)	27/50 (54%)	5/17 (29%)
Grade 2 13/33 (39%)	2/9 (22%)	9/20 (45%)	2/4 (50%)	Grade 2 47/112 (42%)	14/32 (44%)	16/50 (32%)	10/17 (59%)
Grade 3 4/33 (12%)	2/9 (22%)	1/20 (5%)	1/4 (25%)	Grade 3 17/112 (15%)	7/32 (22%)	7/50 (14%)	2/17 (12%)
**Alvarez-Cuesta E, et al. Allergy. 2015**				**Madrigal-Burgaleta R, et al. JACI: In Practice. 2019**			

n, number of patients; DPT, drug provocation test; DHR, drug hypersensitivity reaction.

This table has been modified from the data in the studies by Alvarez-Cuesta et al and Madrigal-Burgaleta et al to show only drug provocation test results from chemotherapy-reactive patients.

Recently, Vazquez-Revuelta et al,. Catalan Institute of Oncology (ICO)/Bellvitge Drug Desensitization Center (DDC) in Barcelona, Spain,- published data on implementing the systematic use of DPT before RDD with chemotherapy within their diagnostic pathways on a different population than that of the RCUH, with promising results, such as a remarkable reduction in unnecessary resource-intensive RDDs.^35^^,^^36^

MGH has also contributed with invaluable data on the use of DPT. However, this group does not use DPT as a diagnostic test systematically before RDD but instead includes DPT at different stages within drug-specific assessment pathways based on skin testing and risk stratification.^5^^,^^38^^,^^47^^,^^139^

These data from different groups strongly support the vital importance of DPT as a critical tool within the management pathways for reactions to chemotherapy. Moreover, these different approaches to DPT are not mutually exclusive, and a recent review article by Hong et al^5^ proposed a management pathway combining them, with the possibility of DPT set at different stages. Regardless, most groups include this procedure (referred to as DPT, “challenge”, or “rechallenge”) within their diagnostic pathways.^39^^,^^50^^,^^95^

DPT is the final criterion standard for a diagnosis of drug allergy. However, not all patients are ideal candidates for DPT, and so patient selection is vital for a safe approach to this technique.^5^^,^^6^ Fortunately, there is a great deal of *in vivo* and *in vitro* diagnostic tools that can be considered risk biomarkers to aid us in deciding whether a patient should be regarded as a candidate for DPT.^1^^,^^2^^,^^6^^,^^22^^,^^126^ These diagnostic risk biomarkers can help in accurate endophenotyping and provide likelihood ratios for a positive or a negative DPT^4^. Thus, they can support a precise risk assessment for delabeling in these patients.

### Delabeling pathways in reactions to chemotherapy

In a recent article, the leading research groups that deal with DHRs to chemotherapy discussed the different challenges and successes of the different delabeling pathways for these drugs.^5^ This article explained how some authors had focused mainly on RDD,^61^ and others had prioritized delabeling with DPT before RDD provided the risk assessment was favorable.^2^^,^^35^ In contrast, other authors had designed complex pathways with risk assessment based on repeated skin testing, progressively shorter RDDs when possible, and eventually delabeling by DPT.^37^^,^^38^^,^^100^^,^^122^ The invaluable amount of data produced in the last decade by these groups and others has made it possible to start considering effective, safe and evidence-based pathways for delabeling, which can be flexibly adapted locally.^1^^,^^5^

Hong et al^5^ identified areas of agreement, controversies and recognized critical unmet needs. However, the fundamental pillars of the pathways for these patients are clear: MDTs led by experts in drug allergy who can help to endophenotype (ie, to classify the signs and symptoms into phenotypes and endotypes by using specific biomarkers), to carry out a personalized risk assessment, to delabel all nonallergic patients (wherever possible, if the risk assessment is favorable), and finally, to provide an appropriate treatment option (ie, to recommend avoidance or desensitization if possible and indicated).^5^^,^^6^^,^^27^

Another crucial factor the authors mention is access to allergy-led dedicated spaces, including specific modifications for manipulating chemotherapy.^300^ See Section 2 and supplementary text 1 for more information on these spaces and handling hazardous drugs like chemotherapy at your allergy department^300^.

**Fig. 1:**
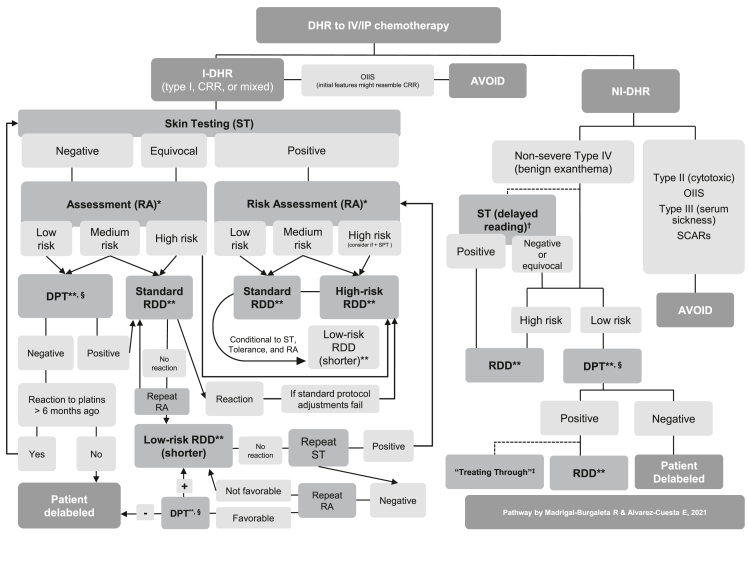

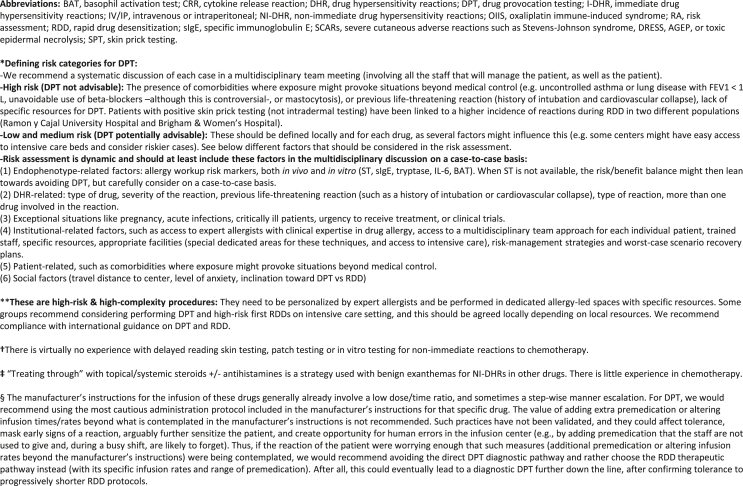
Pathway for the assessment and management of drug hypersensitivity reactions to chemotherapeutic agents.

Table 2 shows a practical example of a DPT to paclitaxel as per RCUH recommendations, and Fig. 1 shows a proposed assessment pathway for reactions to chemotherapy.

**Table 2:** Example of a drug provocation test (DPT) or drug challenge with paclitaxel as per Ramon y Cajal University Hospital (RCUH) standard and cautious protocols for a total dose of 200 mg of paclitaxel that was intended to be administered in 250 ml over approximately 3 h

The standard approach to drug provocation test						
Total dose	200 mg		Solution concentration		Drug	
Solution A	250 ml		0.8 mg/ml		Paclitaxel	
Step	Solution	Rate (ml/h)	Administered volume (ml)	Time (min)	Administered dose (mg)	Cumulative dose infused (mg)
1	A	80	250	187.5	200	200

The cautious approach to drug provocation test^a^:						
Total dose	200 mg		Solution concentration		Drug	
Solution A	250 ml		0.8 mg/ml		Paclitaxel	
Step	Solution	Rate (ml/h)	Administered volume (ml)	Time (min)	Administered dose (mg)	Cumulative dose infused (mg)
1	A	40	20	30	24	24
2	A	80	230	172.5	176	200

Considerations:
(i) Safety is of the essence. DPT with intravenous chemotherapy should be limited to well-selected patients with a favorable risk assessment. It should be done ideally in an intensive care setting or equivalent (a well-equipped Allergy-dedicated Technical Area with complete crash cart, and rapid access to intensive care <1 min), 1:1 patient:nurse ratio, expert nursing staff (well trained in chemotherapy, allergy, and emergency treatment), constant monitoring, constant supervision with nurse and allergist at the bedside (including someone ready to stop the infusion rapidly), emergency anaphylaxis treatment at the ready (including pre-prepared intramuscular adrenaline). Observation after DPT should be at least 1 h after finishing the infusion (or longer, conditional to product information, local guidance, and type of reaction).
(ii) The recommended concentrations and rates for paclitaxel are rather wide and might depend on the condition of the patient or local standard operating procedures. Concentrations and infusion times need to be discussed according to local guidelines (including small details such as whether the line is flushed with the drug or with the solvent saline), and the protocol will need to be altered accordingly. Always check product information leaflets and local protocols for specific administration recommendations in specific populations.
(iii) DPT with chemotherapy agents should be done using standard posology and premedication recommendations as per product information. In the specific case of paclitaxel, the product information states that patients need to be premedicated with corticosteroids, H1 antihistamines, and H2 antihistamines. It is recommended to check local guidance, as there might be variations. This practice differs from guidelines on DPT with other drugs, which usually recommend against the use of premedication (especially with antihistamines or steroids), as it can alter tolerance and hide warning symptoms. However, Madrigal-Burgaleta et al.^b^ have shown data on the usefulness of DPT with paclitaxel whilst still using the standard premedication with corticosteroids, H1 antihistamines, and H2 antihistamines for the DPT.
(iv) Product information leaflets should be available for all products from their manufacturers and must be compliant with the recommendations of the relevant regulatory body, such as www.ema.europa.eu, products.mhra.gov.uk, or https://www.fda.gov/.
(v) Follow recently published guidance on DPT with chemotherapy agents^c^

DPT, drug provocation testing; RCUH, Ramon y Cajal University Hospital; RDD, rapid drug desensitization.

**Note:** The standard approach to DPT is as close as possible to a standard infusion as per product information to avoid the risk of unnecessarily affecting efficacy and to ensure that we confirm tolerance under normal conditions so that nothing alters tolerance to the drug. These drugs are meant to be infused over long periods, so the dose/minute ratio is already low on a standard infusion. In our experience, the key to safety is not necessarily on the infusion rate but on carrying out these techniques in a high-risk area and having experienced and trained staff at the bedside ready to stop the infusion and administrate medication at the first sign of a reaction.

**Note on increasing premedication beyond routine premedication:** The value of adding extra premedication or altering infusion times/rates beyond what is contemplated in the manufacturer's instructions is not recommended. Such practices have not been validated, and they could affect tolerance, mask early signs of a reaction, arguably further sensitize the patient, and create opportunity for human errors in the infusion center (e.g., by adding premedication that the staff are not used to give and, during a busy shift, are likely to forget). Thus, if the reaction of the patient were worrying enough that such measures (additional premedication or altering infusion rates beyond the manufacturer's instructions) were being contemplated, we would recommend avoiding the direct DPT diagnostic pathway and rather choose the RDD therapeutic pathway instead (with its specific infusion rates and range of premedication). After all, this could eventually lead to a diagnostic DPT further down the line, after confirming tolerance to progressively shorter RDD protocols.

a The cautious approach could potentially induce tolerance, and thus, a negative DPT using the cautious approach might benefit from later performing a standard DPT on the next scheduled chemotherapy session.

b Madrigal-Burgaleta R, Bernal-Rubio L, Berges-Gimeno MP, Carpio-Escalona LV, Gehlhaar P, Alvarez-Cuesta E. A Large Single-Hospital Experience Using Drug Provocation Testing and Rapid Drug Desensitization in Hypersensitivity to Antineoplastic and Biological Agents. J Allergy Clin Immunol Pract. 2019;7(2):618–632. DOI:10.1016/j.jaip.2018.07.031.

c Pagani M, Bavbek S, Alvarez-Cuesta E, Dursun AB, Bonadonna P, Castells M, Cernadas J, Chiriac A, Sahar H, Madrigal-Burgaleta R, Sanchez Sanchez S. Hypersensitivity Reactions to Chemotherapy: an EAACI Position Paper. Allergy. 2021; in press. DOI: 10.1111/all.15113

## Section 4: Delabeling allergy to biologics in adult patients (Alvarez-Cuesta & Madrigal-Burgaleta)

### Diagnostic *in vivo* and *in vitro* risk-biomarkers with biologics

As described in Section 3 for chemotherapy agents, the diagnostic *in vivo* and *in vitro* risk-biomarkers are essential to correctly endophenotype patients and thus assess their adequacy for delabeling.^5^^,^^44^

Similar challenges as those found for ST with chemotherapy agents apply to ST with biologics. Even if different groups have found that ST is essential to identify an IgE-mediated endotype, there are still questions regarding non-irritating concentrations and the ideal methodology for performance and interpretation.^5^^,^^6^^,^^23^^,^^44^^,^^140^ Nevertheless, several authors have reported safe and helpful ST with nonirritant concentrations to most biologics that have been in the market long enough to start causing DHRs and needing an assessment by an allergist (when the biologic is easily replaced, physicians tend to use an alternative and avoid referral to an allergy department).^42^ Generally, the only apparent limitation, compared with ST to other drugs, seems to be the elevated price of some biologics.^2^^,^^5^^,^^21^^,^^23^^,^^25^^,^^35^^,^^42^, ^43^, ^44^^,^^140^, ^141^, ^142^, ^143^, ^144^ However, liaison with Pharmacy can help to minimize costs, for example, by booking patients for ST on a day in which the Pharmacy can use remanents from other vials to make the ST dilutions, or by booking patients for ST on the same day of their DPT or RDD, so that Pharmacy can use a small amount of that material for ST.^2^^,^^36^

There is a great deal of experience with determining specific IgE (sIgE) to biologics, and the literature shows a good correlation with ST. Unfortunately, their use is limited by the difficulty of access to commercial platforms.^21^^,^^124^^,^^140^^,^^145^, ^146^, ^147^ Interestingly, cetuximab-reactive patients can be sensitized (even before exposure) to the allergen galactose-alpha-1,3-galactose as part of the “alpha-gal syndrome”, and antibodies against this allergen (which can induce DHRs to cetuximab) can be detected by commercial platforms.^143^^,^^148^^,^^149^

There are limited case reports on using the basophil activation test (BAT) to assess reactions to biologics.^150^, ^151^, ^152^ Other biomarkers, such as tryptase (for a type I hypersensitivity phenotype) or IL6 (for a cytokine release syndrome), are helpful for endophenotyping.^5^^,^^23^^,^^44^

### Drug challenge or drug provocation testing with biologics

Re-exposure to biological agents using standard infusion in patients with mild initial reactions and negative skin testing was already considered by Brennan et al in 2009.^43^ However, most of the experience comes from one center, as specific data on DPT to biologics from large prospective studies have only been published by RCUH.^2^^,^^4^ Nevertheless, Vazquez-Revuelta et al, ICO/Bellvitge DDC, recently published new data on the use of DPT in a different population, which supports the successful use of DPT as a tool for delabeling allergy to biologics.^35^^,^^36^ These promising data can explain why recent comprehensive reviews with proposed algorithms for managing reactions to biologics include the possibility of DPT in the assessment pathways.^5^^,^^6^^,^^25^^,^^42^^,^^44^^,^^153^

As explored in Section 1 of this manuscript, DPT is the Gold Standard for delabeling.^18^^,^^33^^,^^154^ Delabeling prevents non-hypersensitive patients from unnecessary RDD procedures.^2^^,^^4^, ^5^, ^6^ For example, it is remarkable how in the RCUH population, up to 49% of all referred patients with an unequivocal clinical history of a hypersensitivity reaction to biologics had a negative DPT and therefore could avoid RDD.^2^ However, 13 DPTs were positive in a 7-year-long study, and even while most reactions were mild, there was at least one severe reaction as per Brown's classification.^2^ Thus, even if there were no reactions with hemodynamic compromise (grade IV reaction as per RCUH classification), it is undeniable that DPT with biologics is a high-risk technique and must be performed only by experts in drug allergy.^2^^,^^5^ More data on the use of DPT with biologics on Table 3.

**Table 3:** Data on drug provocation testing (DPT) with biologics from the large cohort of patients of the RCUH (Ramon y Cajal University Hospital), Madrid, Spain

Alvarez-Cuesta E, et al. Allergy. 2015		Madrigal-Burgaleta R, et al. JACI: In Practice. 2019	
**Results from all patients referred over three years after a DHR to biologics (n = 30)**		**Results from all referred patients over seven years after a DHR to biologics (n = 95)**	

**DPT Result**	**Biologics** **n = 30**	**DPT Result**	**Biologics** **n = 95**

Negative DPT	9/30 (30%)	Negative DPT	47/95 (49%)
Positive DPT	4/30 (13%)	Positive DPT	13/95 (14%)
DPT not undergone	17/30 (57%)	DPT not undergone	35/95 (37%)
**Results only from the patients who underwent DPT with biologics (n = 13)**		**Results only from the patients undergoing DPT with biologics (n = 60)**	
**DPT Results**	**Biologics** **n = 13**	**DPT Result**	**Biologics** **n = 60**
Negative DPT	9/13 (69%)	Negative DPT	47/60 (78%)
Positive DPT	4/13 (31%)	Positive DPT	13/60 (22%)
**Safety results from the patients with a positive DPT to biologics (n = 4)**		**Safety results from the patients with a positive DPT to biologics (n = 13)**	
**Severity Brown classification**	**Positive DPTs to biologics** **n = 4**	**Severity Brown classification**	**Positive DPTs to biologics** **n = 13**
Grade 1	3/4	Grade 1	5/13 (38%)
Grade 2	2/4	Grade 2	7/13 (54%)
Grade 3	0/4	Grade 3	1/13 (8%)
**Alvarez-Cuesta E, et al. Allergy. 2015**		**Madrigal-Burgaleta R, et al. JACI: In Practice. 2019**	


n, number of patients; DPT, drug provocation test; DHR, drug hypersensitivity reaction.

This table has been modified from the data in the studies by Alvarez-Cuesta et al. and Madrigal-Burgaleta et al. to show only drug provocation test results from patients reacting to biologics.

**Fig. 2:**
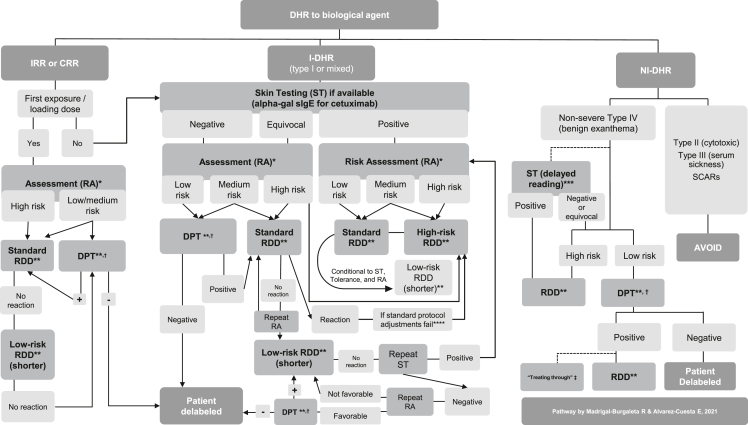

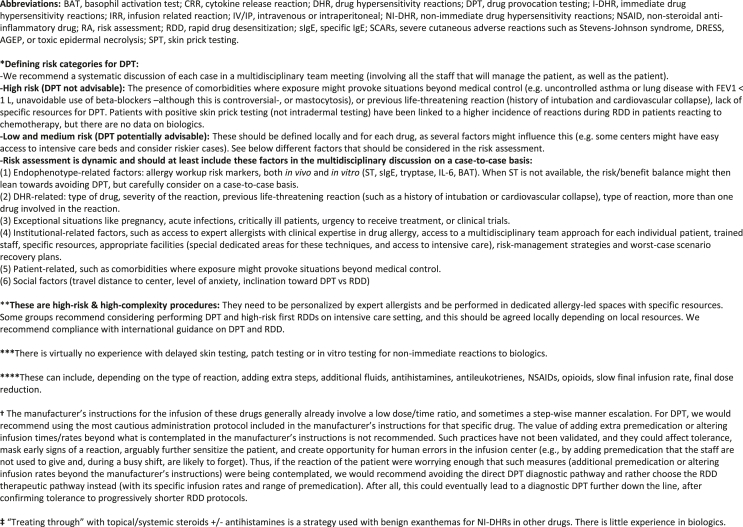
Pathway for the assessment and management of drug hypersensitivity reactions to biological agents.

There is an open discussion about the ideal way to implement delabeling within the diagnostic pathways and the perfect candidates for DPT with biologics.^5^^,^^6^^,^^25^^,^^42^ In any case, given the currently available data, reactive patients should always benefit from assessment by experts in drug allergy, and DPT should be an available option provided the risk assessment is favorable.^5^^,^^42^ Therefore, multidisciplinary decisions are encouraged when considering DPT to ensure a thorough evaluation of the benefits and risks for the patient. Table 4 shows a practical example of a DPT to cetuximab as per RCUH recommendations. Fig. 2 shows a proposed assessment pathway for reactions to biologics.

**Table 4:** Example of a drug provocation test (DPT) or drug challenge with cetuximab as per Ramon y Cajal University Hospital (RCUH) standard and cautious protocols for a total dose of 800 mg of cetuximab that was intended to be administered in 160 ml

The standard approach to drug provocation test:						
Total dose	800 mg		Solution concentration		Drug	
Solution A	160 ml		5 mg/ml		cetuximab	
Step	Solution	Rate (ml/h)	Administered volume (ml)	Time (min)	Administered dose (mg)	Cumulative dose infused (mg)
1	A	60	160	160	800	800

The cautious approach to drug provocation test^a^:						
Total dose	800 mg		Solution concentration		Drug	
Solution A	160 ml		5 mg/ml		cetuximab	
Step	Solution	Rate (ml/h)	Administered volume (ml)	Time (min)	Administered dose (mg)	Cumulative dose infused (mg)
1	A	30	15	30	15	75
2	A	60	145	145	725	800

Considerations:
(i) Safety is of the essence. DPT with intravenous biologics should be limited to well-selected patients with a favorable risk assessment. It should be done ideally in an intensive care setting or equivalent (a well-equipped Allergy-dedicated Technical Area with complete crash cart, and rapid access to intensive care <1 min), 1:1 patient:nurse ratio, expert nursing staff (well trained in the infusion of these drugs, allergy, and emergency treatment), constant monitoring, constant supervision with nurse and allergist at the bedside (including someone ready to rapidly stop the infusion), emergency anaphylaxis treatment at the ready (including pre-prepared intramuscular adrenaline). Observation after challenge should be at least 1 h after finishing the infusion (or longer, conditional to product information, local guidance, and type of reaction).
(ii) The recommended concentrations and rates for cetuximab recommend not exceeding 5 mg/min on the first infusion, and that is the chosen target speed for the DPT. Recommendations might depend on local standard operating procedures. Concentrations and infusion times need to be discussed according to local guidelines (including small details such as whether the line is flushed with the drug or with the solvent saline), and the protocol will need to be altered accordingly. Always check product information leaflets and local protocols for specific administration recommendations in specific populations.
(iii) In the specific case of cetuximab, the product information states that patients need to be premedicated with corticosteroids and antihistamines at least one hour before the infusion. It is recommended to check local guidance, as there might be variations. This practice differs from guidelines on DPT with other drugs, which usually recommend against the use of premedication (especially with antihistamines or steroids), as it can alter tolerance and hide warning symptoms. However, Madrigal-Burgaleta et al.^b^ have shown data on the usefulness of DPT with cetuximab whilst still using the standard premedication with corticosteroids and antihistamines for the DPT.
(iv) Product information leaflets should be available for all products from their manufacturers and must be compliant with the recommendations of the relevant regulatory body, such as www.ema.europa.eu, products.mhra.gov.uk, or https://www.fda.gov/.
(v) Follow recently published guidance^c^

DPT, drug provocation testing; RCUH, Ramon y Cajal University Hospital.

**Note:** The standard approach to DPT is as close as possible to a standard infusion as per product information to avoid the risk of unnecessarily affecting efficacy and to ensure that we confirm tolerance under normal conditions so that nothing alters tolerance to the drug. These drugs are meant to be infused over long periods, so the dose/minute ratio is already low on a standard infusion. In our experience, the key to safety is not necessarily on the infusion rate but on carrying out these techniques in a high-risk area and having experienced and trained staff at the bedside ready to stop the infusion and administrate medication at the first sign of a reaction.

**Note on increasing premedication beyond routine premedication:** The value of adding extra premedication or altering infusion times/rates beyond what is contemplated in the manufacturer's instructions is not recommended. Such practices have not been validated, and they could affect tolerance, mask early signs of a reaction, arguably further sensitize the patient, and create opportunity for human errors in the infusion center (e.g., by adding premedication that the staff are not used to give and, during a busy shift, are likely to forget). Thus, if the reaction of the patient were worrying enough that such measures (additional premedication or altering infusion rates beyond the manufacturer's instructions) were being contemplated, we would recommend avoiding the direct DPT diagnostic pathway and rather choose the RDD therapeutic pathway instead (with its specific infusion rates and range of premedication). After all, this could eventually lead to a diagnostic DPT further down the line, after confirming tolerance to progressively shorter RDD protocols.

a The cautious approach could potentially induce tolerance, and thus, a negative DPT using the cautious approach might benefit from later performing a standard DPT on the next scheduled infusion session.

b Madrigal-Burgaleta R, Bernal-Rubio L, Berges-Gimeno MP, Carpio-Escalona LV, Gehlhaar P, Alvarez-Cuesta E. A Large Single-Hospital Experience Using Drug Provocation Testing and Rapid Drug Desensitization in Hypersensitivity to Antineoplastic and Biological Agents. J Allergy Clin Immunol Pract. 2019;7(2):618–632. DOI: 10.1016/j.jaip.2018.07.031.

c Bavbek S, Pagani M, Alvarez-Cuesta E, Castells M, Dursun AB, Hamadi S, Madrigal-Burgaleta R, Sanchez-Sanchez S, Vultaggio A. Hypersensitivity Reactions to biologicals: an EAACI Position Paper. Allergy. 2021; in press. DOI: 10.1111/all.14984

## Section 5: Delabeling antibiotic allergy in adult patients (Phillips & Trubiano)

### Stewardship imperative for antibiotic delabeling

Patient-reported antibiotic allergies (so-called antibiotic allergy labels [AALs]) are associated with the generation of antimicrobial resistance (AMR) and inferior antimicrobial stewardship (AMS) outcomes, inappropriate antibiotic usage, use of broad-spectrum and restricted antimicrobials, methicillin-resistant *Staphylococcus aureus* (MRSA) colonization, *Clostridium difficile* infection, post-operative infections, prolonged admissions, and delay to appropriate antibiotic therapies.^24^^,^^155^, ^156^, ^157^, ^158^, ^159^, ^160^, ^161^, ^162^ Antibiotic allergy delabeling programs, in both immunocompetent and immunocompromised hosts, have safely been implemented into antimicrobial stewardship programs (ASP) globally, improving patient and hospital outcomes.^163^, ^164^, ^165^, ^166^, ^167^, ^168^, ^169^ There has been a call to incorporate antibiotic allergy delabeling that includes not only delabeling penicillin but other antibiotics, and incorporating delabeling strategies for multiantibiotic labels into ASPs is supported by the recent Infectious Diseases Society of America (IDSA) guidelines.^170^, ^171^, ^172^ The recent development of penicillin allergy risk assessment tools and protocols for direct oral penicillin DPT have enabled widespread implementation of these practices to improve medication safety and ASP endpoints.

### Penicillin allergy – Risk stratification and assessment tools

Previously, a range of antibiotic allergy assessment tools that include ST with or without oral DPT, direct oral DPT and decision support have been published.^167^^,^^173^ Shenoy and colleagues presented an expert opinion algorithm for extrapolating antibiotic allergy assessment into risk-stratification (Table 5).^174^ Devchand et al produced an assessment tool that a non-allergist can utilize to enable an allergy history and subsequent risk stratification, with a resultant sensitivity of 91.8% and specificity of 97.5% (Table 5).^175^^,^^176^ A range of assessment tools and grading systems are now available to aid clinicians in asserting penicillin allergy phenotype correctly.

**Table 5:** Examples of antibiotic allergy assessment criterion

Author (year)	Clinically validated or expert opinion	Risk assessment criteria		
		Low risk	Moderate risk	High risk
Shenoy et al. (2019)^21^	Expert opinion	1. Isolated reactions that are unlikely allergic (eg, GI, headaches) 2. Pruritus without rash 3. Remote (>10 y) unknown reactions without features of IgE 4. Family history of penicillin allergy	1. Urticaria or other pruritic rashes 2. Reactions with features of IgE but not anaphylaxis	1. Anaphylactic symptoms. 2. Positive skin testing 3. Recurrent reactions 4. Reactions to multiple beta-lactam antibiotics
Devchand et al. (2019)^22^	Clinically validated	1. Childhood exanthema unspecified 2. Diffuse rash (>24 h post) > 10 years ago 3. Unknown reaction >10 years ago or family history 4. Isolated reactions are unlikely to be allergic (e.g. GI, headache	1. Urticaria 2. Angioedema 3. Swelling 4. Immediate onset rash (<2 h post dose) 5. Laryngeal or respiratory involvement	1. Anaphylaxis 2. Blistering, pustular or desquamating rash 3. Rash and mucosal ulceration 4. Severe renal, liver or haematological abnormalities 5. Unknown reaction <10 years ago

Clinical decision rules have also been recently employed to allow further point-of-care risk-stratification utilizing minimally available clinical information. A penicillin allergy rule (PEN-FAST) was derived from prospective data from 622 patients that underwent penicillin allergy testing in Melbourne (Australia) and subsequently externally validated in cohorts from Sydney, Perth, and Nashville (United States) (n = 945 patients).^177^ The 4 features associated with a positive penicillin allergy test result on multivariate analysis were subsequently summarized in the mnemonic PEN-FAST: penicillin allergy, 5 or fewer years ago, anaphylaxis/angioedema, severe cutaneous adverse reaction (SCAR), and treatment required for allergy episode. The major criteria included an allergy event occurring 5 or fewer years ago (2 points) and anaphylaxis/angioedema or SCAR (2 points); the minor criterion (1 point), treatment required for an allergy episode. A cutoff of fewer than 3 points for PEN-FAST was chosen to classify a low risk of penicillin allergy, for which only 17 of 460 patients (3.7%) had positive results of allergy testing (Area under the curve (AUC), 0.805; Negative predictive value (NPV) 96.3% (95% CI, 94.1%–97.8%)). There have been other successful attempts at clinical decision rule development. Stevenson et al, from a retrospective cohort of 447 Australian patients with a penicillin allergy, identified benign rash more than 1 year previous as a low-risk criterion (sensitivity, 80.6%; specificity, 60.8%).^178^ Siew et al used a retrospective cohort from the United Kingdom (n = 1092) to identify predictors of true antibiotic allergy and generated low-risk criteria consisting of (1) no anaphylaxis, (2) reaction more than 1 year ago, and (3) no recall of index drug (NPV of 98.4%).^179^ Chiriac et al from a French retrospective β-lactam allergy cohort generated a clinical decision rule that was unable to predict allergy (AUC, 0.67; sensitivity, 51%; NPV, 83%).^180^ Stone et al applied a risk assessment rule to effectively delabel patients of penicillin allergy in the medical intensive care unit.^181^ A range of penicillin allergy clinical results are available. PEN-FAST appears to offer advantages over alternative models due to international validation, high NPV and use of prospective data for internal derivation and validation.

### Penicillin allergy – evidence for direct oral drug provocation testing or drug challenge

There is increasing evidence for the use of direct oral penicillin DPT without ST on benign NI-DHRs.^182^ A recent article by Rose et al reviewed the recent literature up to May 2020 for direct oral DPT in 1912 accumulative low-risk penicillin allergy patients, with adverse event rates of 0–4.2% and 0–8.8% for outpatient and inpatients noted, respectively. Heterogeneity was present in selected phenotypes, DPT steps and drug dosing.^183^ Chua et al, in the most recent study, integrated direct oral penicillin DPT into a whole-of-hospital ASP, demonstrating significant benefits to appropriate prescribing (2-fold increase) and narrow-spectrum penicillin (10-fold increase) usage.^81^ Direct oral DPT is the most cost-effective delabeling strategy,^81^ and penicillin delabeling overall consistently proves to be cost-saving.^184^^,^^185^ A summary table of direct oral DPT studies is shown in Table 6.

**Table 6:** Direct oral penicillin challenges in the inpatient and outpatient setting of primarily adults^a^ – Adapted from Rose et al.^134^ (2020)

Author/Region	Year	N	Setting	Assessor	Prospective	Patients	Low-Risk Definition	Follow-up	Oral Challenge (full dose)	ADR
Trubiano et al. (AUS)^152^	2018	58	Inpatient Outpatient	Infectious Diseases	Yes	Adults	**Any one of:** Type A reaction (non-immune mediated)/Unknown reaction >10 years prior/Benign childhood rash/Non-urticarial rash/Maculopapular exanthem >10 years prior	5 Days	1-step penicillin/amoxicillin (250 mg)	0%
Blumenthal et al. (USA)^116^	2019	76(6)^b^	Inpatient	Allergist/Non-specialist medical/Physician Assistant/Nurse	No	Adults	**Any one of:** Minor rash (not hives)/Maculopapular rash/Recorded allergy which patient denies	–	2-step amoxicillin	3.9% (immediate)
Du Plessis et al. (NZ)^153^	2019	34	Inpatient	Pharmacist	Yes	Adults	Delayed onset Rash **and** > 5 years ago	1 month + 1 year	5-step amoxicillin (500 mg)	8.8% (immediate)
Li et al. (AUS)^154^	2019	56	Inpatient	Allergist/Immunologist	Yes	Adults	**Any one of**: Type A reaction (non-immune mediated)/Immune mediated reaction **Excluding:** Anaphylaxis (<10 years)/IgE mediated <1 year/Hemolytic anemia/Serum Sickness/Severe cutaneous reactions	3 Days	3-step amoxicillin (250 mg) then 3days	3.6% (delayed)
Ramsey et al. (USA)^155^	2020	48	Inpatient	Allergist/Immunologist	Yes	Adults	**Any of one:** Rash/Hives/Itching/Unknown **and** >20 years ago **and** Nil emergency medical attention	2 weeks	3-step amoxicillin (500 or 875 mg)	2% (immediate) 4% (delayed)
Stone et al. (USA)^132^	2020	54	Inpatient (ICU)	Allergist/Immunologist	Yes	Adults	**Any one of:** Urticaria only >5 years distant/Self-limited rash/Gastrointestinal symptoms only/Remote childhood reaction with limited details/Family history of penicillin allergy alone/Avoidant due to fear of allergy/Known tolerance of penicillin post-reaction/Other symptoms-non-allergy	Chart review at 7 months	1-step amoxicillin (250 mg)	0%
Chua et al. (AUS)^135^	2020	200	Inpatient	Infectious Diseases	Yes	Adults	**Any one of:** Type A reaction (non-immune mediated)-where direct de-labelling not accepted by patient/Unknown reaction >10 years prior/Benign childhood rash/Injection site reaction/Maculopapular exanthem >10 years prior	90 Days	1-step penicillin/amoxicillin (250 mg)	1.5%^c^ (immediate) 1.5% (delayed)
Livirya et al. (NZ)^156^	2020	41	Inpatients	Allergy	Yes	Adults	Limited cutaneous reaction (including rash and hives), unknown > 6 months ago and >1 h post drug	6-months	1-step amoxicillin (250 mg)	0%
Tucker et al. (USA)^157^	2017	328	Outpatient	Allergist/Immunologist	No	Adults	All reactions **Excluding:** Severe cutaneous reactions/Hepatitis/Hemolytic anemia/Nephritis	–	1-step amoxicillin (250 mg)	1.5%^b^ (immediate)
Banks et al. (USA)^c^^,^^133^	2019	283	Outpatient	Allergist/Immunologist	No	Adults/Children	**Any one of:** Benign rash/GI Symptoms/Headache/Benign somatic symptoms/Unknown **and** >1 year ago	–	1-step amoxicillin (250 mg)	0%
	2018	380	Outpatient	Allergist/Immunologist	No	Adults	All reactions **Excluding:** Severe cutaneous reactions/Hepatitis/Hemolytic anemia/Nephritis	–	1-step amoxicillin (250 mg)	1.1% (immediate)
Iammatteo et al. (USA)^158^	2019	155	Outpatient	Allergist/Immunologist	Yes	Adults	All reactions **Excluding:** Bronchospasm or laryngeal edema requiring intubation/Anaphylactic shock/Severe non-IgE-mediated reactions (SJS, TEN, Interstitial nephritis, Hepatitis, Hemolytic anemia, DRESS, Cutaneous/mucosal blisters, Hypersensitivity vasculitis, Pneumonitis, Pulmonary fibrosis and Serum sickness)	1 Month	2-step amoxicillin (500 mg)	2.6% (immediate) 4.2% (delayed)
Kuruvilla et al. (USA)^159^	2019	20	Outpatient	Allergist/Immunologist	No	Adults	**Any one of:** Non-specific benign rash/Remote unknown reaction/Benign somatic symptoms **and** >1 year ago	–	1-step amoxicillin (500 mg)	0%
Stevenson et al. (AUS)^129^	2019	167	Outpatient	Allergist/Immunologist	No	Adults	“Low-risk” defined by center-specific protocols	3–7 Days	1 or 2-step amoxicillin or implicated penicillin	3.6% (immediate)
Mustafa et al. (USA)^143^	2019	79	Outpatient	Allergist/Immunologist	Yes (RCT)	Adults + paeds (mean age 35.3)	**Any one of:** Skin rash/Hives/Itching/Unknown **and** >10 years ago **and** Nil emergency medical attention	–	2-step amoxicillin (400 mg)	3.8% (immediate)
Savic et al. (UK)^160^	2019	56	Outpatient (Pre-Operative)	Nurse	Yes	Adults	**Any one of:** Nausea/Vomiting/Diarrhea/Non-itchy rash/No hospital admission/Thrush/Unknown **and** >15 years ago	5–7 Days	3-step amoxicillin then 3 days (500 mg)	2% (Immediate)

a Paediatric patients were included in some of the studies. Adults were considered >16 years of age.

b Received IM adrenaline to prevent reaction progression (nil systemic features identified).

c Data presented in review article and not published previously.

Similar studies have demonstrated the efficacy of oral DPT in pediatric patients.^186^^,^^187^ In the outpatient setting, the efficacy of direct amoxicillin DPT without ST has been demonstrated in children.^188^ Mill et al studied 818 children in the outpatient allergy setting and demonstrated that graded direct oral amoxicillin DPT was tolerated in 94.1%, even when including potentially moderate- and high-risk allergies (eg, anaphylaxis).^189^ Abahams and Ben-Shosan recently reviewed the literature,^190^ and in conjunction with recommendations from British Society of Allergy and Clinical Immunology (BSACI) and American Academy of Allergy Asthma and Immunology (AAAAI)/World Allergy Organization (WAO), proposed a recommendation for direct oral DPT in NI-DHRs, and potentially I-DHRs, to amoxicillin.^77^^,^^191^

The available literature is now overwhelming for the support of direct oral DPT and, in particular, the integration into ASP. Previous small cohort studies are now supported by large prospective multicentre inpatient studies^81^ and randomized control data.^192^ The use of direct oral penicillin DPT should be employed in the inpatient setting under specialist observation and routinely in the outpatient allergy setting. Future studies are required to standardize the testing parameters, in particular: single vs multi-step DPTs (1-step vs multi-step), final accumulated dose and duration of DPT (single dose vs multi dose).

However, many members of the reviewing panel of this document have recommended caution and avoiding overenthusiastic approaches to direct oral DPT. Please, see Section 11 for some comments from other colleagues on this approach.

### Cephalosporin and other beta-lactam allergy

Anaphylaxis occurs in less than 0.001% of parenteral exposures of cephalosporins.^24^ As documented for penicillin allergy, immediate hypersensitivity reactions to cephalosporins can wane over time.^193^ There is less information about the validity of cephalosporin ST. One study suggested that almost 70% of patients had lost ST sensitivity to cephalosporins after 5 years.^194^^,^^195^ However, cross-reactivity between cephalosporins appears to be largely driven by their shared R1 side chains.^193^ In particular, in Europe and Australia, there may be cross-reactivity between aminopenicillins and amino cephalosporins based on the shared R1 side-chain.^195^, ^196^, ^197^ Evidence exists to suggest that cephalosporin testing is not necessary in patients with a penicillin allergy label with unknown cephalosporin tolerance.^195^ For low-risk aminocephalosporin allergy, where drugs are only available orally and there is no ST strategy, direct oral DPT is a reasonable approach to delabeling. Cephalosporin ST is useful for diagnosis of an I-DHR to the suspected drug and also to identify cross-reactivity patterns related to typically shared R1 but occasionally shared R2 side-chains. However, accumulating evidence suggests that such patients will tolerate all other penicillins and cephalosporins with distinct side chains. Cefazolin has distinct R1 and R2 side-chains.^77^^,^^198^ Cefazolin is currently the most common cause of perioperative anaphylaxis in some countries. Ceftriaxone is also a common cause of anaphylaxis and shared R1 side-chains with cefepime, cefotaxime and other cephalosporins. Current evidence indicates that cross-reactivity between penicillins and other beta-lactams such as carbapenems, which do not share either a class-specific ring or an R1 or R2 side-chain, is <1%.^24^

### Sulfonamide antibiotics

It is well established that there is no cross-reactivity between sulfonamide antibiotics and non-antibiotic sulfonamides.^199^ Currently, it is estimated that sulfa antibiotics are the most common antibiotic label following penicillin.^200^ The approach to sulfonamide DHRs might vary depending on the type of reaction and the risk assessment; however, the usefulness of skin testing seems to be limited to specific cases and DPT seems to be the main delabeling tool.^198^ Previous guidelines supported multidose DPT approaches to sulfa antibiotics. However, the evidence base that this was needed is weak, and there is concern that these approaches could represent desensitization rather than delabeling.^201^ More recent studies reinforce that direct sulfa antibiotic DPT accomplished by single-dose DPT in those with remote low-risk reactions or multi-step DPT in those with a history of immediate reactions can be conducted safely with excellent efficacy and effectiveness.^202^ In one recent study of oral DPT in adults with sulfa antibiotic allergy labels, 98.9% of those with an unspecified sulfa allergy could safely receive a single or 2-dose direct trimethoprim-sulfamethoxazole DPT.^202^

### Fluoroquinolone allergies

Recent animal and human studies support that many reactions previously thought to be IgE-mediated allergies associated with fluoroquinolones may be non-IgE reactions mediated through the interaction between fluoroquinolones and the MRGPX2 mas-related G-protein coupled receptor on mast cells.^203^ This has been supported by tolerance of lower dose fluoroquinolones in the vast majority of patients with allergy labels to fluoroquinolones.^204^ The apparent lack of cross-reactivity between fluoroquinolones may also be supported by the different affinity of fluoroquinolones for this receptor.^203^ In addition, true anaphylaxis has been reported to be more common with moxifloxacin which has been estimated to cause approximately 54% of fluoroquinolone associated anaphylaxis.^205^^,^^206^

However, the Reviewing Panel has considered it advisable to discuss data from a recent study in which hypersensitivity to quinolones was diagnosed in 128 of the 170 patients assessed.^206^ This study detected a certain degree of cross-reactivity among fluoroquinolones. Remarkably, DPTs with alternative quinolones were positive in 13 of 24 subjects with a well-demonstrated quinolone hypersensitivity. In particular, DPTs with levofloxacin were positive in 3 of the 5 subjects with ciprofloxacin hypersensitivity, while DPTs with ciprofloxacin were positive in 5 of the 8 subjects with hypersensitivity to moxifloxacin.

ST with fluoroquinolones has been problematic secondary to their innate ability to cause concentration-dependent non-IgE mediated mast cell activation. However, recent studies suggest that oral DPT may be a valid mechanism to delabel fluoroquinolone IgE mediated reactions and that ST may potentially be used to differentiate IgE and non-IgE mediated mast cell activation.^204^^,^^206^ These studies suggest that up to 75% or more of those with immediate reactions to fluoroquinolones will tolerate the implicated fluoroquinolone when undergoing diagnostic drug challenge. It is quite likely in these cases that the original reaction may have been more likely related to Mas-related G protein-coupled receptor-X2 (MRGPRX2) activation. In patients with a non-anaphylactic immediate reaction to fluoroquinolones it seems reasonable to conduct a single or graded observed diagnostic drug challenge to the implicated fluoroquinolone. In patients with anaphylactic reactions, it could be argued that the time from the index reaction to the study could be relevant, as patients with positive ST results seem to have a more recent history of a reaction, and thus suggest that sensitization wanes over time.^204^ This is in line with findings from other groups.^205^^,^^206^ However, whether this means clinical allergy wanes over time is unclear, and studies have struggled to find statistical significance when comparing outcomes in patients with more recent vs more distant DHRs.^205^^,^^206^ SPT and IDT with the highest non-irritating concentrations can be helpful in the diagnosis of a true IgE mediated reaction and to help characterize cross-reactivity.^204^

## Section 6: Intravenous desensitization to chemotherapy in adult patients (Madrigal Burgaleta & Alvarez-Cuesta)

### General considerations

DHRs to chemotherapy have increased in recent years, and these DHRs can jeopardize treatment completion, thus affecting survival outcomes and quality of life for reactive patients. Fortunately, as we explored in Section 1, RDD is a cost-effective procedure that allows hypersensitive patients to receive their treatment safely and maintain the same survival outcomes as their non-hypersensitive counterparts.^7^^,^^61^

In this section, we will assume that the patient has already undergone an allergy workup. As per guidelines, patients should have a confirmed hypersensitivity before considering RDD.^58^ As explained in Section 3, *in vivo* and *in vitro* techniques will help us reach that diagnosis. However, some patients will inevitably have an unconfirmed diagnosis, and this usually happens in patients with negative biomarkers who cannot undergo confirmatory DPT for whatever reason (eg. unfavorable risk assessment).^5^

The detailed clinical history and the results obtained from the *in vivo* and *in vitro* biomarkers will help us endophenotyping these patients. Most chemotherapy-reactive patients will present with a type I hypersensitivity (as per Gell & Coombs) endophenotype (whether IgE-dependent or non-IgE-dependent). However, as discussed in Section 3, some chemotherapeutics, such as oxaliplatin, can also present with mast-cell-independent cytokine release reactions (CRRs) or even with reactions that include mixed symptoms resembling both type I and CRR symptoms. Bearing this in mind is essential, as it can impact the ideal premedication, the odds of a breakthrough reaction, the choice of protocol, the management of breakthrough reactions, and the later adjustments to the protocols of reactive patients.^22^,^298^ Personalized risk assessment is the key to safety during RDD, and there are different non-mutually exclusive approaches to it.^5^

In Section 1, we discussed the general considerations of RDD, including its indications and contraindications. However, this section will delve into the development of RDD protocols for chemotherapy and the specific considerations we must bear in mind.

### Rapid drug desensitization protocols

Only the use of validated RDD protocols is recommended.^58^ Three existing groups (BWH, MGH, RCUH) have validated their flexible standard protocols in a large series of patients for different types of reactions and a wide range of drugs.^2^^,^^3^^,^^61^^,^^122^^,^^125^^,^^207^^,^^208^

Desensitization was first used in the mid-twentieth century in patients experiencing type I reactions to penicillins.^5^ In 1994, Wong et al^208^ published an RDD protocol for vancomycin that served as a template for some of the MGH current RDD protocols. According to Wong et al,^122^ MGH RDD protocols for oxaliplatin “*were adopted standardized protocols for Partners Institutions (Massachusetts General Hospital, Brigham and Women's Hospital, and Dana Farber Cancer Institute)*. *They were similar to our published protocols used for carboplatin and cisplatin, which were modifications of our previously published vancomycin desensitization protocol*.”^122^

Other protocols like BWH or RCUH were inspired by previous clinical experiences but incorporated the data obtained from *in vitro* models, such as one published by Sancho-Serra et al.^68^ According to Castells:^56^ “*the protocols used for in vitro desensitization have been adapted in vivo and further adaptations have produced safe protocols for human use with similar dose increments and interval times.*” Indeed, the adaption of *in vitro* data into *in vivo* real-life practice and continuous adaptation to produce safer and more personalized protocols has been at the heart of BWH and RCUH protocols.^2^^,^^5^^,^^56^, ^298^ Again, according to Castells:^56^ “these human protocols have now been used in thousands of cases with remarkable safety since the inhibitory mechanisms of desensitization protect against anaphylaxis.” Indeed, both BWH and RCUH have each published data of over 1000 RDD procedures for a wide range of drugs. Their impressive efficacy and safety profiles guarantee the success of this technique in real life, even in 2 remarkably different populations, in the hands of expert allergists with access to the necessary resources.^2^^,^^61^

The RCUH protocol published by Madrigal-Burgaleta et al^3^ was designed to last for approximately four hours, thus allowing for single-morning RDDs. It was also designed so that each solution bag had an individual infusion line previously primed with the dilutor substance, a local requirement for handling hazardous drugs in some hospitals.^3^^,^^209^ Table 7 features a practical example of an RDD protocol for a high-risk patient as per RCUH, and Fig. 1 shows a management pathway for RDD in DHRs to chemotherapy.

**Table 7:** Example of a high-risk version of the standard flexible RCUH RDD protocol for a total dose of 200 mg of oxaliplatin meant to be administered in a volume of 250 ml over two hours

Total dose	200 mg	Solution concentration	Total dose in each solution (mg)	Drug
Solution A′	250 ml	0.0016 mg/ml	0.4	Oxaliplatin
Solution A	250 ml	0.016 mg/ml	4	Oxaliplatin
Solution B	250 ml	0.16 mg/ml	40	Oxaliplatin
Solution C	250 ml	0.8 mg/ml	200	Oxaliplatin

Step	Solution	Rate (ml/h)	Administered volume (ml)	Time (min)	Administered dose (mg)	Fold increase per step (mg/min)	Approximative cumulative dose infused (mg)
1	A′	88	22	15	0.0	NA	0.0
2	A′	100	25	15	0.04	NA	0.04
3	A′	200	50	15	0.08	×2	0.12
4	A′	400	100	15	0.16	×2	0.28
5	A	88	22	15	0.0	NA	0.28
6	A	100	25	15	0.4	×2.5	0.68
7	A	200	50	15	0.8	×2	1.48
8	A	400	100	15	1.6	×2	3.08
9	B	88	22	15	0.0	NA	3.08
10	B	100	25	15	4.0	×2.5	7.08
11	B	200	50	15	8.0	×2	15.08
12	B	400	100	15	16.0	×2	31.08
13	C	88	22	15	0.0	NA	31.08
14	C	123	211.15	102	168.92	×1.6	200.0
Total infusion time: 297 min (4 h and 57 min)							
**Premedication:** Ideally, they should comply with the manufacturer's instructions and institutional protocols for standard oxaliplatin infusion. We do not recommend systematic additional premedication (e.g. with steroids or antihistamines) as a measure to prevent breakthrough reactions, however, tailored premedication may be added depending on a personalized case-to-case approach.							
**Total dose calculation and discarded volume:** Not all the volume in solutions A′, A, B, or C is infused. The protocol starts with one solution A′ containing a 1:500 dilution. Solution A contains a 1:50 dilution, solution B a 1:5 dilution, and the final solution C contains the full concentration (1:1) of the culprit drug. The total dose to be infused during solution C is calculated by subtracting the cumulative dose administered in steps 1–13 from the total desired dose.							
**Adjustments to the volume of the bags:** The standard volume in the solution bags for the RCUH RDD protocol is 250 ml. In some cases, bag volume might need adjustment depending on the manufacturer's instructions and/or product information.							
**Additional bags for high-risk patients:** The standard RCUH flexible protocol uses 3 bags to be administered over 10 steps, starting on bag A (1:50 dilution). This is a version of the protocol for high-risk patients in which an additional solution A’ (1:500 dilution) has been added be added before solution A. This modification provides a lower starting dose. In patients with positive ST, the starting dose can potentially be determined based on an endpoint titration according to local protocols.							
**Flushing steps:** Each solution uses an individual infusion line previously primed with 22 ml of the solvent serum (be aware that platins need glucose 5% serum, not saline). Steps 1, 5, 9, and 13 are considered “line flushing steps” (in which 22 ml of the solvent serum are administered). Line flushing steps must be adapted to local circumstances. For example, in this case, RCUH used 22 ml infusion systems for the infusion pumps (Alaris® SE I pump smartsite infusion set), hence the 22 ml flushing steps.							
**Adjustments to final infusion rate:** Step 14 may be adapted to the desired final infusion rate according to the standard regimes indicated by the referring oncologist (additional steps may be added to reach higher infusion rates while maintaining a maximum dose increasing by 2-fold to 2.5-fold with each step).							
**Avoiding human errors:** Infusion pumps with automatic multi-step infusion options (e.g., RCUH used Alaris® SE double channel for this protocol) should be used to avoid human errors associated with manually changing infusion rates every 15 min.							

RCUH, Ramon y Cajal University Hospital; NA, not applicable; RDD, rapid drug desensitization; ST, skin testing.

The BWH protocol published by Castells MC et al^207^ lasts approximately 6 hours, similar to that by MGH. It was designed so that the individual infusion lines for each solution bag are primed with the chemotherapeutic, thus allowing for minimal volumes to be infused during the initial steps.

RCUH and BWH protocols are based on a three-bag standard flexible protocol that can be personalized for the individual patient. For instance, the starting dose can be modified for a more cautious protocol for selected high-risk patients by including more solutions (typically a four-bag protocol), or more steps can be added in patients who develop breakthrough reactions during RDD. In addition, they have been validated for several different types of drugs and reactions (IgE-dependent and non-IgE-dependent).^2^^,^^61^ RCUH, as per European guidelines, includes the possibility of deciding the initial dose based on an ST endpoint titration for patients with positive ST.^2^^,^^3^^,^^58^ Determining the initial dose this way is more typical of food allergy, but it has also been recommended in drug allergy, for instance, in antibiotic RDD.^67^

As discussed in the Introduction and Section 2, RDD protocols need to be validated in the local population. MGH features one of the most ambitious internal validation efforts in large series of patients, not only of their flexible protocol for RDD but also of the delabeling diagnostic pathways (based on risk-stratification and ST).^37^^,^^38^^,^^47^^,^^59^^,^^100^^,^^122^^,^^125^^,^^210^^,^^211^ Indeed, MGH's Banerji et al^95^ have been trying to understand for many years how other doctors manage reactions to chemotherapeutics at their institution and how they can most efficiently help their colleagues and their patients, and this has influenced their sophisticated assessment pathways. MHG's pathways offer flexibility using clinical history in conjunction with ST to guide RDD protocols tailored to each patient and specific drug, intending to avoid unnecessary RDDs and optimize resources.^100^^,^^122^^,^^125^

Most centers use variations of these standard protocols depending on risk assessment and other factors, such as longer protocols for high-risk patients and progressively shorter protocols in lower-risk individuals with negative ST results who have tolerated RDD without breakthrough reactions.^2^^,^^22^^,^^139^ In any case, irrespective of the choice of protocol depending on local characteristics, ideally, internal validation of the protocol on a series of patients should be pursued as soon as possible, as countless factors might incur on local variations that can impact the correct functioning of RDD, from drug preparation to human errors, different administration protocols, or even automated infusion machines.^2^ Thus, identifying local strengths, hazards, risks and establishing error prevention strategies is vital for successfully improving patient outcomes.^2^ Recent efforts for internal validation include those applying these existing protocols to their specific hospitals.^35^^,^^36^^,^^212^

Some groups have made modifications to the protocols to serve local needs and benefit from tailored improvements.^213^, ^214^, ^215^, ^216^ Remarkably, there have been recent efforts to design protocols based on the use of one bag alone, which could reduce complexity in preparing the drug, avoid using low concentrations that might affect drug stability, and reduce human errors.^213^, ^214^, ^215^, ^216^, ^217^, ^218^ However, there are still some methodological limitations to one-bag protocols that need to be addressed. Firstly, there is a lack of large series of well-characterized patients (including DPT to minimize false-positive diagnosis of allergy) using one-bag protocols and including enough numbers of severe reactors. To date, only one study by Sala-Cunill et al. has actively attempted to meet these criteria and, remarkably so, compared the results of using three-bag protocols versus one-bag protocols in their population, finding no differences between them.^218^ Hopefully, further future publications will cast some light on this. Nevertheless, the current evidence suggests that one-solution protocols are certainly a valuable tool in low/medium-risk patients.

The choice of protocol is a complex issue, as many factors will be involved: local hazardous drugs handling guidelines, workload (both in the allergy department and the pharmacy department), drug stability, patient risk assessment, error prevention policies, or type of drug. However, protocols published by different groups may co-exist in one center^298^. For instance, hazardous drugs handling policies may motivate one center to use RCUH's protocols for chemotherapy and, instead, BHW's protocols for non-hazardous drugs.^2^^,^^10^^,^^12^, ^298^ Similarly, one center may use three-bags protocols for one type of drug and one-bag protocol for a different one.^2^,^298^ And, hypothetically, a given center usually using one-bag protocols may decide to use the 16-step BWH's protocol for the occasional especially high-risk patients, as this protocol reaches remarkably low initial doses.^61^

Irrespective of the choice of protocol for RDD, these need to adhere to two basic principles based on the current *in vitro* and *in vivo* experience:^2^^,^^56^^,^^58^^,^^62^^,^^198^ (i) The initial dose should be around a 1000–10,000th of the target dose (100th of the initial dose could be considered for the shorter transition protocols in low-risk patients with good tolerance) and be subthreshold for anaphylaxis. (ii) There should be around 10–16 steps of approximately 2-fold to 2.5-fold incremental doses of the drug antigen at fixed 15–30 minute time intervals.

Furthermore, protocols alone are no guarantee for success.^298^ Therefore, for optimal outcomes, it is agreed that these protocols are to be used by expert allergists, within an institutional MDT, and in dedicated spaces with the adequate resources.^2^^,^^5^^,^^25^^,^^36^^,^^219^, ^220^, ^221^ We strongly discourage RDD being performed without the direct supervision of an allergy team with experience in drug allergy and specific training in chemotherapy RDD. As explained in Section 2, unstructured management by isolated teams without the input of allergists can lead to patients unnecessarily stopping their first-line therapies or being exposed to unnecessary risks.^47^

### Premedication for rapid drug desensitization

There are no evidence-based guidelines for premedication in RDD, as data from systematic studies are scarce.^1^, ^41^

Medical practitioners typically believe that adding systematic “anti-allergic” premedication will prevent breakthrough reactions and make RDD easier. However, counterintuitively, some centers support minimizing premedication and only using the minimum recommended by the manufacturer's instructions for the first RDD procedure.^2^^,^^25^^,^^36^ These groups only add additional premedication to subsequent RDDs if necessary, ie, after breakthrough reactions during the first RDD attempts. The results seem similar to other groups, with most RDDs being uneventful after the third attempt.^2^^,^^22^ It is of note that manufacturer's instructions already use considerable premedication, and additional drugs might not improve the effectiveness of RDD, yet they could potentially hide early warning breakthrough reactions.^2^^,^^25^^,^^36^ ^9^On the other hand, other centers use systematic premedications.^25^^,^^61^^,^^141^^,^^142^

In any case, all these groups consider that the decision should be personalized to the individual patient, the type of reaction, and the specific drug, as particular cases might need different management.^2^^,^^23^^,^^25^^,^^61^ For example, antihistamines can be used for H1 and H2 blockage, montelukast or zileuton to prevent respiratory symptoms, acetylsalicylic acid can prevent symptoms caused by prostaglandins, other NSAIDs, opioids and steroids can be used to avoid CRR symptoms such as fever/chills or pain, even benzodiazepines can have a role to relieve the anxiety caused by the procedure.^2^^,^^19^^,^^25^^,^^61^^,^^222^ Interestingly, adding fluids as a type of premedication has shown promising results for specific kinds of reactions, especially in biologics.^23^

Recent evidence shows that routine premedication with antihistamines and steroids in RDD to paclitaxel might be unnecessary, could be associated with unwanted effects, and could potentially mask the onset of breakthrough reactions whilst they are mild.^9^

Routinely adding corticosteroids does not seem beneficial for RDD unless recommended by the manufacturer's instructions, for other purposes (eg, antiemetic), or in patients with specific endophenotypes.^22^^,^^23^^,^^222^^,^^224^

Interestingly, avoiding routine premedication with antihistamines in RDD with platins allowed to produce *in vivo* data on mast cell involvement in the RDD process by confirming the negativization of STs after RDD.^3^

### Breakthrough reactions during rapid drug desensitization

Most RDD procedures (74–88%) are entirely uneventful,^2^^,^^61^ and breakthrough reactions during RDD procedures are mostly mild (58–73%);^2^^,^^61^ however, up to 39–42% of the patients undergoing RDD can potentially suffer a breakthrough reaction.^2^^,^^61^ Some authors have found that a positive skin prick test (SPT) result or a total IgE over 100 UI/l could be predictors of breakthrough reactions during RDD with platins.^2^^,^^128^ Nevertheless, further studies are needed in this regard.

Interestingly, breakthrough reactions usually occur during the first 3 procedures, which could be explained by the careful personalizations to the patient's protocol by the expert allergist.^2^^,^^22^ However, they can happen at any point, even after several uneventful RDD procedures.^2^^,^^22^^,^^61^

The treatment of these breakthrough reactions should follow local guidelines, but RCUH devised a specific management tool, which can be useful in RDD to chemotherapy.^2^ After a reaction is controlled, RDD should continue where it was stopped.^2^, ^61^

There is always a non-zero risk of anaphylaxis during RDD, and we must bear in mind that those anaphylactic reactions can be fatal even after many successful RDD procedures.^98^ Furthermore, potentially fatal reactions of a nature different to anaphylaxis, such as oxaliplatin immune-induced syndrome (OIIS), can happen at any time also need monitoring.^65^ For these reasons, patients under RDD should remain under the direct care and supervision of the allergy department.^2^^,^^61^^,^^1^, ^5^, ^41^, ^298^

After a breakthrough reaction, most authors recommend tailored adjustments to the following RDD procedure, and these could come in the form of customized premedication (either upfront or right before reactive steps), decelerating dose escalation with intermediate steps, more prolonged RDD procedures starting at a lower dose and concentration, or temporary final dose reduction.^2^^,^^61^ Omalizumab has been successfully used as premedication in difficult cases.^225^, ^226^, ^227^, ^228^, ^229^, ^230^, ^231^, ^232^ It is helpful to keep in mind that other drugs such as premedication or concomitant antineoplastics/biologics can be possibly involved as the cause of breakthrough reactions during RDD.^2^^,^^10^

### Specific phenotypes of patients and considerations

The description of endophenotypes in drug allergy to chemotherapy has been a landmark in the diagnosis and tailored management of these patients.^22^^,^^27^ In addition to the endophenotypes we discussed in Section 3, an article by Madrigal-Burgaleta et al^2^ described a series of “phenotypic patterns”:

(1) “First RDD”, Most reactive patients, regardless of the culprit drug, react during their first RDD procedure. Thus, they recommend specific safety measures during the first procedure.^2^ These authors found that once reactive patients achieve an uneventful RDD (usually by the third attempt), it is uncommon for patients to suffer more reactions, and they can be transferred to lower risk areas, which is compatible with the experience of other authors.^2^^,^^21^^,^^22^
(2) However, some patients can suffer “breakthrough reactions after several uneventful RDDs”, and thus all patients need to remain under the care of the allergy department.^2^^,^^21^^,^^22^
(3) Some patients might present with “fever/chills” either from the initial reaction or after several uneventful procedures. Fever/chills could be compatible with a cytokine release syndrome (which will need specific premedication) or an OIIS (when oxaliplatin is involved and specific alterations are identified in the blood tests, and which would be a reason to stop administering oxaliplatin altogether).^2^^,^^21^^,^^22^^,^^65^
(4) Some authors have found that a “positive skin prick test (SPT)” during the allergy workup after a DHRs can be associated with a higher risk of a breakthrough reaction during RDD, and so these patients should be managed carefully.^2^^,^^61^
(5) Patients with an old history (> 6 months) of a reaction with platins show a tendency to have negative ST and even DPT, but then become “positive converters”^2^, ^4^.These patients might need tailored approaches, especially during their assessment, as discussed in Section 3.
(6) Chemotherapy-reactive patients are surprisingly likely to suffer a DHR to more than one “concomitant drugs”, and DPT becomes essential to study these patients^2^, ^10^.
(7) When those patients are confirmed as hypersensitive to two different drugs that happen to be involved in the same chemotherapy scheme, these patients might require “double RDD” (i.e. RDD to two different drugs at the same time because RDD is antigen-specific^62^). The most common combinations of double RDD are leucovorin/oxaliplatin, leucovorin/irinotecan, carboplatin/paclitaxel, cyclophosphamide/mesna, cisplatin/mannitol, cyclophosphamide/docetaxel.^2^^,^^4^^,^^10^:

## Section 7: Intravenous desensitization to biologics in adult patients (Madrigal-Burgaleta & Alvarez-Cuesta)

### General considerations

The use of biologics has increased remarkably in recent years, and so have DHRs to these therapies.^23^ Unfortunately, DHRs to biologics not only can be severe but can also compromise the use of first-line therapies for fear of inducing further reactions.^23^^,^^47^ Despite the available data on the success of therapeutic techniques like RDD for the management of DHRs to biologics, referrals to the allergy department can be as low as 4% (or even inexistent in many centers), and many patients are unnecessarily changed to alternative therapies or are unnecessarily put at risk of further reactions without an appropriate allergy workup.^47^

This section will assume that patients have already followed the delabeling pathway, including a full allergy workup and risk assessment, before considering RDD, as explained in Section 4. In Section 1, we discussed the general considerations of RDD, including its indications and contraindications. However, this section will focus on how this applies to the specific characteristics of biologics. In addition, Supplementary text 2 will briefly review some specific practicalities of drug desensitization in subcutaneous biologics.

### Rapid drug desensitization protocols

Recent reviews show how there is an array of different local empirical protocols, which are either originally designed or are variations of previous protocols, generally showing single cases or data from small populations.^25^^,^^220^ However, there are mainly three groups using RDD protocols that are compliant with the findings of *in vitro* models for RDD and, importantly, validated *in vivo* on series of well-studied patients, namely, those used by BWH, MGH, and RCUH.^5^^,^^25^^,^^1^, ^2^, ^23^, ^44^, ^62^, ^74^ Section 6 discussed the origins, similarities and differences of these different protocols.

The RCUH standard flexible RDD protocol for biologics was validated on 30 patients undergoing 178 RDD procedures with different biologics (rituximab, infliximab, natalizumab, and trastuzumab).^2^ Even if most of the RDD experience with cetuximab RDD has involved one-bag protocols, a separate group successfully used this 3-bag protocol for cetuximab in a reactive patient who was sensitized to alpha-gal.^143^ Interestingly, this patient showed decreased alpha-gal sIgE levels after completing 10 RDD procedures, which could inform future research on the behaviour of biomarkers overtime during RDD.^143^ Table 8 features a practical example of the RCUH RDD protocol, and Fig. 2 shows the management pathways for RDD in DHRs to biologics.

**Table 8:** Example of the standard RCUH RDD protocol for a total dose of 500 mg of infliximab meant to be administered in a volume of 250 ml over two hours

Total dose	500 mg	Solution concentration	Total dose in each solution (mg)	Drug
Solution A	250 ml	0.04 mg/ml	10	Infliximab
Solution B	250 ml	0.4 mg/ml	100	Infliximab
Solution C	250 ml	2 mg/ml	500	Infliximab

Step	Solution	Rate (ml/h)	Administered volume (ml)	Time (min)	Administered dose (mg)	Fold increase per step (mg/min)	Approximative cumulative dose infused (mg)
1	A	88	22	15	0.0	NA	0.0
2	A	100	25	15	1	NA	1.0
3	A	200	50	15	2	×2	3.0
4	A	400	100	15	4	×2	7.0
5	B	88	22	15	0.0	NA	7.0
6	B	100	25	15	10	×2.5	17.0
7	B	200	50	15	20	×2	37.0
8	B	400	100	15	40	×2	77.0
9	C	88	22	15	0.0	NA	77.0
10	C	125	212.5	101.5	425	×1.6	500.0
Total infusion time: 236.5 min (3 h, 57 min, 30 s)							
**Premedication:** Ideally, they should comply with the manufacturer's instructions and institutional protocols for standard infliximab infusion. We do not recommend systematic additional premedication (e.g. with steroids or antihistamines) as a measure to prevent breakthrough reactions, however, tailored premedication may be added depending on a personalized case-to-case approach.							
**Total dose calculation and discarded volume:** Not all the volume in solutions A, B, or C is infused. The protocol starts with one solution A, which contains a 1:50 dilution. Solution B contains a 1:5 dilution, and the final solution C contains the full concentration (1:1) of the culprit drug. The total dose to be infused during solution C is calculated by subtracting the cumulative dose administered in steps 1–9 from the total desired dose.							
**Adjustments to the volume of the bags:** The standard volume in the solution bags for the RCUH RDD protocol is 250 ml. In some cases, bag volume might need adjustment depending on the manufacturer's instructions and/or product information.							
**Additional bags for high-risk patients:** The standard RCUH flexible protocol uses 3 bags to be administered over 10 steps. This can be flexibly adapted for high-risk patients by adding a solution A’ (1:500 dilution) with additional 4 steps before solution A. This modification provides a lower starting dose. In patients with positive ST, the starting dose can potentially be determined based on an endpoint titration according to local protocols.							
**Flushing steps:** Each solution uses an individual infusion line previously primed with 22 ml of the solvent serum (be aware that infliximab needs saline serum as solvent). Steps 1, 5, and 9 are considered “line flushing steps” (in which 22 ml of the solvent serum are administered). Line flushing steps must be adapted to local circumstances. For example, in this case, RCUH used 22 ml infusion systems for the infusion pumps (Alaris® SE I pump smartsite infusion set), hence the 22 ml flushing steps.							
**Adjustments to final infusion rate:** Step 10 may be adapted to the desired final infusion rate according to the standard regimes indicated by the referring physician (additional steps may be added to reach higher infusion rates while maintaining a maximum dose increasing by 2-fold to 2.5-fold with each step).							
**Avoiding human errors:** Infusion pumps with automatic multi-step infusion options (e.g., RCUH used Alaris® SE double channel for this protocol) should be used to avoid human errors associated with manually changing infusion rates every 15 min.							

RCUH, Ramon y Cajal University Hospital; NA, not applicable; RDD, rapid drug desensitization; ST, skin testing.

The BWH standard flexible RDD protocol was validated on 23 patients undergoing 105 RDD procedures with different biologics (rituximab, infliximab, trastuzumab).^43^ Recently, the BWH group has presented further data on 104 patients undergoing 526 RDD procedures with a wide range of biologics, including data also on subcutaneous procedures.^23^ Moreover, the BWH protocol has been widely used for an array of biologics in different populations.^35^^,^^36^^,^^141^^,^^142^

MGH has successfully used their RDD protocols, similar to their previously published protocols for chemotherapeutics, in combination with risk stratification with the goal of decreasing unnecessary RDDs.^47^^,^^74^ This group recently published data using their protocols for 25 rituximab-reactive patients who underwent 170 RDD procedures using three related protocols.^74^

An unmet need is how to make these RDD protocols easier to prepare for the pharmacy department to save time and minimize the risk of errors (e.g. one-bag RDD protocols), and in this sense, both the BWH and the RCUH groups have successfully used the same one-bag RDD protocol for cetuximab,^2^^,^^144^ with promising data by Madrigal-Burgaleta et al on 6 patients undergoing 77 one-bag RDD procedures.^2^ Interestingly, another group shared data successfully using a one-bag protocol on patients reacting to rituximab, cetuximab, and natalizumab.^214^ And recently, another group published one-bag RDD procedures for cetuximab, obinutuzumab, and trastuzumab.^218^

Most centers use variations of their standard protocols depending on risk assessment and other factors, such as more prolonged protocols for high-risk patients and progressively shorter protocols in good responders.^2^^,^^23^^,^^74^ Understandably, some centers might need further local adaptations to the standardized published protocols, so internal validation of the RDD protocols is essential, as countless factors could affect the RDD outcomes.^2^^,^^74^

Irrespective of the choice of protocol, as explained in more detail in Section 6, we encourage allergy-led MDTs to use protocols validated in a large series of patients and compliant with the current *in vitro* knowledge on the mechanisms of RDD.^58^^,^^62^

### Premedication for RDD

There are no evidence-based guidelines for premedication in RDD to biologics. Please see Section 6 for a more detailed discussion.

### Breakthrough reactions during RDD

Most RDD procedures with biologics (72–87% of all RDD procedures) are completely uneventful.^2^^,^^23^^,^^61^^,^^142^ However, up to 40–58% of the patients undergoing RDD to biologics can potentially suffer a breakthrough reaction.^2^^,^^142^ Interestingly, a meaningful percentage of breakthrough reactions during RDD seems to be caused by CRRs.^23^ This could explain why RDD to biologics can feature patients who tend to encompass multiple breakthrough reactions, which can be progressively more severe in some cases.^2^^,^^23^ In any case, severe breakthrough reactions during RDD procedures with biologics are still rare (accounting for 2–17% of the reactive RDD procedures),^2^^,^^23^^,^^61^^,^^142^ and breakthrough reactions during RDD are significantly milder than the initial reaction to the drug.^23^

The treatment of these reactions should follow local guidelines, but RCUH devised a useful tool for RDD in biologics.^2^ After a reaction is controlled, RDD should proceed where it was stopped.^2^, ^23^, ^61^

Interestingly, as discussed in Section 6 for chemotherapy, breakthrough reactions usually occur during the first three procedures and then become rare. This is easily explained by the personalized adjustments made by expert allergists to the protocols of reactive patients.^2^ However, they can happen at any point, even after several uneventful RDD procedures.^2^^,^^61^ For this reason, even if the specific location for RDD or the protocols can be adjusted after several uneventful procedures, patients under RDD should remain under the care and direct supervision of the allergy department.^2^^,^^61^

After a breakthrough reaction, most authors recommend tailored adjustments to the following RDD procedure, and these could come in the form of customized premedication (either upfront or right before reactive steps), decelerating dose escalation with intermediate steps, more prolonged RDD procedures starting at a lower dose and concentration, or temporary final dose reduction.^2^^,^^61^, ^298^ Recent data by Isabwe et al^23^ suggest that dose reduction and fluids as premedication could be helpful in patients with multiple recalcitrant reactions. It is beneficial to keep in mind that other drugs such as premedication or concomitant antineoplastics/biologics can be possibly involved.^2^^,^^10^

The BWH seems to have reduced the number of reactive RDD procedures to biologics in the last years when comparing the data from Sloane et al^61^ and Isabwe et al.^23^ Describing different endophenotypes to better guiding the selection of the optimal RDD protocol and premedication (including fluids) might constitute the landmark that could potentially explain this remarkable improvement. However, more data are needed in different populations.

## Section 8: Intravenous desensitization to antibiotics in adult patients (Wong)

Investigations for antibiotic allergy, including DPT, are usually standard procedures for an allergy department.^19^ However, the need for RDD to intravenous antibiotics is not as frequent. Some allergy departments based in larger hospitals may have to provide care for inpatients. Some inpatients may have a label of allergy or may present with a DHR during their admission. An antibiotic from a different class can be used in most cases, and investigations can be postponed until the patient is stable. However, there may not be an equally effective alternative for some of these patients in some situations. Thus, an allergy workup might be needed on site.

In some patients, the allergy workup may confirm an allergy to the required drug. However, in other cases, the risk assessment may not be favorable for the patient to undergo confirmatory testing, leaving the allergist with a patient in need of a specific antibiotic to which there is an unconfirmed allergy label and with no option for confirmation in the acute setting. In both of these situations, RDD may be a life-saving option for these patients.^19^

In this section, we will explore the section authorgroup's standard practical approach to intravenous RDD to antibiotics. In addition, Supplementary text 3 describes how to approach these patients before considering whether they are appropriate candidates for RDD.

### General approach

#### Intravenous (IV) rapid drug desensitization

##### Informed consent

Informed consent should be obtained from the patient, responsible family member, or caretaker if available. As explained in Section 1, an additional informed consent should be requested from the referring physician confirming that there is an indication for the patient to be treated with the given drug despite DHRs. This ensures the indication is documented and discussed with the patient, that there is a multidisciplinary discussion, and that the drug of choice is clearly specified by the referring physician to avoid mistakes.

##### The site of desensitization may depend on the resource available

If available, RDD should take place in the inpatient setting, and intensive care unit (ICU) if the patient is high risk. A monitored inpatient unit may be used if the risk is low to moderate or it is deemed that the benefit outweighs the risk for the particular patient when an ICU bed is not available.

A monitored outpatient unit with emergency medications and personnel may be used if the risk is low to moderate when an inpatient unit is unavailable.

In any case, there should be an expert allergist at the bedside. And option is for a mobile allergy team (eg, allergist and nurse) to visit the patient in the ICU or the inpatient setting. An ideal option is to move the patient to the allergy-dedicated Technical Area were high-risk allergy procedures are usually performed. This would involve a discussion with the patient's team to ensure that the patient can be safely transported there. See supplementary text 1 for an example of a Technical Area.

We strongly discourage allergists providing non-allergist inpatient teams with RDD protocols for them to carry out RDD procedures at the ward without the specific supervision of an allergist, as if this was standard practice. This could be potentially risky for the patient, as the space might not be suitable for these high-risk and high-complexity procedures, and the staff in the ward might not be specifically trained in RDD and anaphylaxis, might lack experience in drug allergy, might not be able to constantly monitor and supervise the patient, or – in worst-case scenarios – might not even be aware that an RDD is being performed or what it entails.

##### Prior to the desensitization

Obtain baseline vital signs including temperature, blood pressure (BP), pulse rate, and O2 sat %.

Ensure that emergency medications (epinephrine, H1 antihistamines, H2 antihistamines, IV fluid, and bronchodilators/nebulizers) are available.

When possible, try to minimize cofactor(s) that can interfere with the desensitization, such as concurrent narcotics or other agents with direct mast cell degranulating (DMCD) properties.

##### The procedure

As discussed in Section 1, the general principle is to start with a minute quantity that the body can tolerate safely. This is generally 1/10,000–1/1000 of the final dose but may range from 1/10^6^ to 1/1 starting at a very slow speed. The choice is dependent on the particular antibiotic and the severity of the previous reaction. If the suspicion for an anxiety reaction is high, normal saline may be administered as the first step (as a single-blind placebo). If a hypersensitivity reaction occurs at these low doses, the physician may step back and start lower. However, it is important to do so only for reaction(s) likely reflecting a true hypersensitivity reaction and not due to anxiety.

Increase the infusion rate over time, usually at intervals of 15–30 min. The ideal protocol should increase the rate at small to moderate multiples but sufficiently fast to allow completion of the RDD in a reasonable period of time.

As a specific feature of this approach, the protocol that we have developed often alternates between 3× and 3.3 times every other step at the early to mid-portion of the RDD.^208^ These increments were chosen to be an approximation of the square root of 10 (3.14). The net result is that there will be a 10-fold increase in the RDD infusion rate after every 2 steps. As discussed in Section 1, based on *in vitro* data, near-doubling doses every 15–30 minutes theoretically is safer than going up by 10-fold every 30 minutes. We typically use the time interval between steps starting at 15 minutes, but it may be adjusted to every 10–30 minutes. At 15 minutes intervals between steps, the early to the middle part of the RDD protocol will increase 3.0- to 3.3-fold every 15min, equivalent to a 10-fold increase every 30min. For the typical patient that starts at 1/1000 dilution for the initial step, the first 100 fold increase can be achieved by 60 minutes, and the target full-strength infusion rate can be reached 90 minutes into the RDD. This can be accelerated by using a 10-minute interval, achieving the 3.0–3.3 fold increase by 10 minutes and the 10-fold increase by 20 minutes. In situations where the risk for reactions is higher, a longer interval may be pursued. For 30 minutes intervals, the time for 3.0–3.3 fold increase slows to 30 minutes and for 10-fold increase slows to every 60 minutes, respectively.

Once the desensitization reaches the full strength dilution, we advise slowing the advancement to a 2-fold increase or less as our experience indicates that more patients are likely to develop a reaction at this point of the desensitization.

In general, there are no evidence-based guidelines on the use of antihistamines, steroids, or other pretreatments for antibiotic RDD. There is one exception of note, vancomycin, a well known direct mast cell degranulator (DMCD), may sometimes benefit from premedication and not necessarily need RDD, but this should be assessed by expert allergists (see detailed information on specific antibiotic classes below). In addition, if the patient had required antihistamines for cutaneous reactions during previous desensitization, antihistamines may also be used prophylactically.

##### If a reaction occurs during any stage of the desensitization

If the reaction is mild (eg, grade 1 as per Brown's classification), the physician may try treating through the reaction by adding H1 antihistamines, +/− H2 antihistamines, and a temporary continuation at the same rate until the reaction subsides.

If the reaction is moderate (eg, grade 2 as per Brown's classification), the physician may hold the infusion, add H1 antihistamines, +/− H2 antihistamines, and bronchodilators/respiratory medications if necessary. Epinephrine should be ready and observe if symptoms subside. If improved, may restart at 25–50% of the previously tolerated infusion rate and resume incremental desensitization at the rate(s) that is tolerated by the patient.

If the reaction is severe (life-threatening reactions like grade 3 reactions as per Brown's classification), administer epinephrine 0.15 mg (0.15 ml of 1 mg/ml for lighter children) to 0.3 mg (0.3 ml of 1 mg/ml for bigger children and adults) or 0.5 mg (0.5 ml of 1 mg/ml for adults weighing over 50 kg) IM in the thigh immediately. The antibiotic infusion should be paused. IV fluid should be administered if available. Additional measures may be pursued depending on the type of reaction, available resources, local guidelines, specific circumstances and response to treatment. The RDD may be resumed at 10–20% of the last infusion rate if the patient has recovered completely and it is deemed that the benefit outweighs the risk to further pursue RDD.

If the reaction appeared to have a threshold beyond which reactions continue to recur despite the desensitization process, administration of the remaining dose might be capped and administered at the highest tolerated drug infusion rate.

As additional considerations, repeat measurement of vital signs at each step, take photos of any cutaneous reaction, draw serum tryptase level within 1–2 hours of the reactions if laboratory measurement is available. If the patient developed severe back pain, fever, and/or darkened urine, draw complete blood count, differential, platelet count, urinalysis, and compare with baseline. Detail documentation of the complete desensitization process to serve for future reference.

##### Oral desensitization (rapid and slow protocols)

For antibiotics and other drugs that are only available in oral forms, oral desensitization is the protocol of choice. The need for this may arise in reactive patients in different scenarios, from using metronidazole for trichomoniasis to the urgent need for treatment with antituberculosis drugs.^198^ This exceeds the objectives of this manuscript, which is focused on intravenous RDD, but we will mention some general considerations.

The principle is the same as for the intravenous desensitization protocols with the following modifications:

If the drug is soluble in water, dissolve the drug in a defined volume of water (filtered, previously boiled, bottled, or distilled water may be used). Preparation should start with the amount of water that will dilute the drug to an easily defined concentration such as 1 or 10 mg per ml starting concentration. For example, for an antibiotic vial that contains 250 mg of lyophilized powder, add sufficient water to bring the final volume to 25 ml to make the full concentration of 10 mg/ml. Label the concentration.

If the drug is not soluble in water, crushed the drug by mortar or open the capsule and suspend the drug in a defined volume of water (filtered, previously boiled, bottled, or distilled water may be used) in an empty small medical bottle. Preparation should start with the amount of water that will dilute the drug to an easily defined concentration such as 1 or 10 mg per ml starting concentration as above. Ideally, we should have support from the pharmacy department.

Again, in liaison with the pharmacy department, the next steps should be to make serial 10-fold dilutions by taking a defined volume of the solution or suspension with the highest concentration (such as 3 ml) and adding sufficient water to make a 10-fold less concentrated solution/suspension (27 ml). For suspension, be sure to shake the medication container just prior to removing the defined volume to be diluted to avoid settling of the suspension. A convenient container will be a small empty clean medication bottle. A small beaker or paper cup may also be used. Label each dilution.

The remaining steps will be similar to the intravenous desensitization protocol with oral substituted for the intravenous. However, specific practical considerations will need to be considered, for example, to shake the container that will be used for each step and remove the desired amount with a small measuring spoon or pipette and give to the patient to take orally. Then proceed in similar steps as the intravenous desensitization protocol, bearing in mind similar principles (an initial dose that is subthreshold for anaphylaxis and lower than 1/1000 of the target dose, near-doubling dose increases in a step-wise manner every 15–30 minutes in at least 10 steps).^62^ The oral desensitization protocols have been adopted to provide successful desensitization for many other drug classes; however, wherever possible published protocols tested in large series of well-characterized patients should be used. Liaison with pharmacy is essential to ensure quality control of the preparations. Ideally, all these drug preparations should be prepared and checked by pharmacy and delivered timely and safely to the area where desensitization will take place.

### Detailed information on specific antibiotic classes

Whilst this section described the general practical approach towards desensitization to antibiotics, specific antibiotic classes might have distinctive features that will influence the practical approach to the individual patient. Please see supplementary text 4 for beta-lactams, supplementary text 5 for fluoroquinolones, supplementary text 6 for vancomycin, supplementary text 7 for sulfonamides, and supplementary text 8 for macrolides.

### Supplementary information on non-IV drugs

The section's author group has a longstanding experience in delabeling and desensitization in drug allergy. We thought it would be useful for the reader to have access to further information on drug desensitization (supplementary text 9) for other drugs (namely, aspirin and other NSAIDs, balsalazide, clopidogrel, ethacrynic acid, hydroxychloroquine, lamotrigine, methadone, metronidazole, sulfadiazine, simvastatin).

## Section 9: Delabeling & intravenous desensitization to miscellaneous drugs (Cuesta-Herranz & Guzman-Melendez)

### Introduction

Whilst there is extensive experience with desensitization protocols for some drugs, there is a body of miscellaneous drugs for which only case reports or series of cases have been reported. Patients in need of treatment with these drugs and lacking alternatives will surely benefit from assessment by an expert allergist if they are to receive safely the treatment they have reacted to previously. For these patients, allergists might find themselves in the situation of having to use desensitization protocols with little documented experience. In this section, we will review desensitization with intravenous drugs with scarcely documented experience in the literature. Hopefully, this can aid allergists in the decision-making process, knowing that, even if the general principles of desensitization apply, the recommendations described in this section might be supported by low-quality evidence and will need a great deal of experience on the part of the allergist to successfully and safely manage these patients.

### Specific drug groups

See supplementary text 10 for corticosteroids, supplementary text 11 for intravenous iron, supplementary text 12 for heparins, supplementary text 13 for radiocontrast media, supplementary text 14 for insulin, supplementary text 15 for fluorescein, supplementary text 16 for vitamins, and supplementary text 17 for diuretics. In addition, medications used for rare diseases can be irreplaceable. There are commendable examples in the literature of allergy departments liaising with teams managing rare diseases to ensure that all patients received their optimal first-line treatment despite DHRs. For instance, Aranda et al. published their remarkable experience with enzyme replacement therapy desensitization.^233^

## Section 10: De-labeling & intravenous desensitization in children - chemotherapy, biologics, antibiotics - (Broyles & Maciag)

### Introduction

DHRs are relatively common in pediatric patients, occurring in up to 9.5% of hospitalized patients and 1–8% of outpatient visits.^234^, ^235^, ^236^ Medication-induced anaphylaxis represents the most common cause of fatal anaphylaxis among pediatric patients in the United States.^237^ These reactions can be unpredictable and dose-dependent, making them difficult to diagnose and manage.^18^^,^^238^

Although a significant number of reactions can be serious, unfortunately, many pediatric patients are inappropriately labeled as medication-allergic.^239^ Many of the symptoms attributable to DHRs in children may be virus-induced or related to a drug-virus interaction. These may not represent a persistent, drug-specific hypersensitivity reaction. These confounders complicate the diagnosis of pediatric drug allergy.^240^

An article by Dioun et al explored how the approach to the diagnosis and management of DHRs applies to children as well as adults, with several caveats.^234^ If possible, ST to the offending agent should be completed in the outpatient setting, preferably >4 weeks after the reaction to reduce false-negative results.^18^^,^^201^^,^^241^ If there is a history of mild reaction, particularly if ST is negative, DPT as a diagnostic procedure may be performed in a monitored setting to observe if the medication in question provokes a reaction. Unlike RDD, DPT does not modify the child's immunologic response to a drug.^242^ See Section 1 for a review of these general concepts.

In cases with confirmed immune-mediated DHRs, particularly in cases with moderate to severe reactions, RDD may be considered if the medication is considered first-line treatment or alternatives are not available. The decision to desensitize a child should be made carefully as severe breakthrough reactions are possible.^241^, ^242^, ^243^, ^244^ Informed consent must be obtained with every patient and/or their parents/guardians, with a careful discussion of the risks, benefits, and alternatives to the procedure.^244^ For very young pediatric patients, or those with developmental disabilities, RDD in an intensive care setting may be advantageous, as they may require more careful monitoring.^61^ At Boston Children's Hospital, pediatric patients with mild-moderate reactions undergo initial RDD in the step-down unit of the intensive care, while those with severe reactions are desensitized in the intensive care unit.^244^ After a well-tolerated RDD, repeat procedures may be performed in a carefully-monitored outpatient unit with appropriately-trained staff.^61^ Pediatric RDD have been completed successfully to various antibiotics,^245^, ^246^, ^247^ monoclonal antibodies,^45^^,^^75^^,^^246^^,^^248^ and chemotherapeutics as well as other drugs.^249^, ^250^, ^251^

### Pretreatment

As discussed in Section 6, there are no evidence-based guidelines on premedication for RDD. However, in the experience of the authors of this section, pretreatment regimens for children are aimed at preventing or minimizing the severity of breakthrough reactions. Regimens for children may include diphenhydramine (1 mg/kg up to 50 mg), and/or ranitidine (1.5 mg/kg up to 150 mg), administered 20 minutes prior to the initiation of the protocol. Second or third generation antihistamines, such as cetirizine, are also used and may mitigate reactions towards the end of a protocol. Montelukast may be used, especially when RDD was previously unsuccessful.^222^ In children, CRRs during RDD for monoclonal antibodies or chemotherapeutic agents may be ameliorated by the administration of acetaminophen (15 mg/kg, maximum 650 mg) with histamine blockers.^252^

### Antibiotics

The ramifications of being labeled with a pediatric antibiotic allergy are serious and potentially long-lasting. In fact, 75% of children diagnosed with penicillin allergy were labeled before age 3. As discussed in Section 5, the label of penicillin allergy is frequently perpetuated into adulthood, precipitating the use of less effective, more expensive and broader-spectrum antibiotics, with potentially more adverse effects.^253^^,^^254^ In reality, after the indicated evaluation, >90% may be able to tolerate the penicillin in question.^255^, ^256^, ^257^ Many cephalosporins may be inappropriately restricted in children labeled as penicillin-allergic. The rate of cephalosporin allergy in those with penicillin allergy is now known to be 2%, significantly less than the previously reported 8%.^174^ Careful evaluation of the molecular structure and side-chain configuration is helpful in understanding the risk of reaction to other related beta-lactam antibiotics.^240^ Remarkably, some older articles suggest that cefuroxime is non-cross-reactive with other cephalosporins. However, data from different studies indicate a significant cross-reactivity rate with ceftriaxone, cefotaxime and cefepime given a shared methoxy-imino group on the R1 side chain.^258^

Aside from penicillins, RDD procedures to other antibiotics have been successfully completed in children. For example, successful desensitizations to doxycycline, minocycline, tigecycline^259^ and clindamycin^245^ have been reported in pediatric patients.

### Biologics

RDD to monoclonal antibodies (mAbs) is essential. Many children who have experienced DHRs to these drugs may not have alternative therapeutic options. Treatment with humanized and murine mAbs has caused systemic DHRs in children, which limit their use.^244^^,^^250^ Reactions to mAbs may be IgE-mediated, cytokine release-related, or IgG antibody-mediated.^23^^,^^45^^,^^248^

Various RDD protocols have been implemented for an array of mAbs, including infliximab and trastuzumab, and allow continued first-line therapy despite DHRs.^2^^,^^144^^,^^207^ Other RDD protocols for biologic agents, such as rituximab (Table 9),^75^ and tocilizumab^260^ have been used successfully in children. In our experience, younger patients undergoing RDD to mAbs benefit from longer procedures with slower infusion rates.^75^ As pediatric RDD procedures become more common, we are finding that carefully planned protocols are successful in children and allow continued administration of first-line therapy.^261^^,^^262^

**Table 9:** Successful Pediatric Rituximab Desensitization Protocol. Total time 648 min infusion; final infusion rate: 2.0 mg/kg/hour. Each Solution was 250 ml total volume

Rituximab Desensitization Protocol for Pediatric Patients						
Solution			Rituximab/bag (mg)		Concentration (mg/ml)	
1			2.06		0.008	
2			20.6		0.082	
3			205.189		0.821	
**Step**	**Solution**	**Rate (ml/h)**	**Rate (mg/kg/h)**	**Time (min)**	**Dose/Step (mg)**	**Total Dose (mg)**
1	1	1	0.0006	15	0.0021	0.0021
2	1	2.5	0.002	15	0.0052	0.0072
3	1	5	0.003	15	0.0103	0.0175
4	1	10	0.006	15	0.0206	0.0381
5	2	2.5	0.02	15	0.0515	0.0896
6	2	5	0.03	15	0.103	0.1926
7	2	10	0.07	15	0.206	0.3986
8	2	20	0.1	15	0.412	0.8106
9	3	5	0.3	15	1.0259	1.8366
10	3	10	0.7	15	2.0519	3.8885
11	3	20	1.3	15	4.1038	7.9922
12	3	30	2	482.5	198.0078	206

Reprinted with permission from Dilley MA, Lee JP, Platt CD, Broyles AD. Rituximab Desensitization in Pediatric Patients: Results of a Case Series. Pediatr Allergy Immunol Pulmonol 2016; 29:91–4.

### Chemotherapy

Due to advances in modern medicine, childhood cancer survivors are living longer and often require repeated courses of chemotherapeutic agents.^263^ A variety of immunological and non-immunological mechanisms may mediate hypersensitivity to chemotherapy.^264^ Chemotherapy is often combined with anti-emetics, analgesics and antimicrobials, which can complicate the diagnosis of drug allergy. RDD to chemotherapeutics has been successfully completed and reported in children, including RDD to pegasparaginase,^251^ methotrexate,^249^ brentuximab,^265^ and etoposide.^250^ Some authors consider that pre-treatment with corticosteroids which can be included as part of the chemotherapy regimen, such as dexamethasone (10 mg/m2), may be helpful.^263^ However, as discussed in Section 6, this is controversial. Success in RDD procedures may be malignancy-specific as well as protocol-dependent, as has been shown for carboplatin.^266^^,^^267^

### Conclusions

DHRs represent a significant problem for children. Many children may inappropriately be labeled with specific drug allergies when the allergy is incorrectly diagnosed initially, or has since been outgrown, emphasizing the importance of delabeling. ST regimens and DPT are diagnostic tools that can be used in appropriate children to evaluate drug hypersensitivity. RDD procedures to a variety of medications including antibiotics, biologics and chemotherapeutic agents have been successfully completed in pediatric patients when no alternate medications are available or, continued administration of first-line therapy is indicated.

## Section 11: Limitations, unmet needs, and comments from Review panel Members

### Controversies around IgE: The correct use of the terms “allergy” and “desensitization”

The Reviewing Panel recommended mentioning that some authors reserve the word “allergy” to describe IgE-mediated acute-onset hypersensitivity.^67^ These authors believe that we should not speak of “allergy” when describing events that may be acute-onset hypersensitivity or delayed-onset hypersensitivity with no IgE involvement. On the other hand, some authors believe that the term “drug allergy” can be applied to any immunologically mediated response in a sensitized person, including non-IgE mediated and non-immediate hypersensitivity.^201^

Some authors consider that type I Gell & Coombs DHRs should only include IgE-mediated DHRs.^18^ In contrast, other authors believe that non-IgE-mediated DHRs should also be considered as type I.^19^

As we briefly discussed in Section 1, some authors consider that desensitization is only possible for IgE-mediated acute-onset hypersensitivity or another acute-onset mast-cell mediated hypersensitivity.^67^ These authors believe that desensitization is not possible when referring to T-cell-mediated delayed-onset hypersensitivity.^67^ Some reviewers have suggested that T-cell mediated drug hypersensitivity reactions become clinically apparent over a period of days after exposure and that there is no known mechanism to reduce the severity of these reactions by slowly increasing the cumulative exposure over a period of hours or days. They consider that most evidence is based on case reports or small series of cases compatible with either eventful or uneventful challenges when individuals with a history of potential delayed-onset hypersensitivity are re-exposed to the implicated agents.

On the other hand, other authors agree with the low quality of the current evidence but cannot ignore the growing body of *in vitro* data on the involvement of T-cells in desensitization and *in vivo* experiences that support that desensitization can be helpful in CRRs and specific NI-DHRs.^2^^,^^19^^,^^22^^,^^23^^,^^63^^,^^64^^,^^66^ Further studies are needed to cast some light on this controversial issue.

### Controversies on the feasibility of a universally applicable model of an allergy department

The reviewing panel is concerned about geographical and cultural differences regarding the structure of allergy departments and the effective management of high-complexity drug allergy patients. For example, some reviewers remarked how some countries such as the United States or Spain have a long tradition of using rapid drug desensitization in drug allergy, whereas some allergists from Northern Europe barely receive referrals for desensitization. This reality entails that allergists in different countries will have different degrees of experience. Thus, allergists from some countries will be used to dealing with high-complexity drug allergy patients, where allergists from other countries may have no experience. Starting networks (funding for multicentre studies, mentoring programs in partnership with centers of excellence, external advisory services, or fellowships) and promoting collaboration may help reduce these differences in practice.

Some reviewers argue that, depending on the country and the demand for the procedure, only a single center (in small countries), or relatively few, should be established to ensure sufficient patients to maintain routine and optimal quality assurance, including research. Moreover, they believe that investigating patients with suspected drug allergies not only involves allergists but exceptionally skilful and educated allergists. In other words, establishing a center for drug allergy and desensitization should not be a standard procedure for any given allergist. They find that drug challenge (DPT) and drug desensitization should be only for the few excellent and dedicated allergists in multidisciplinary cooperation. However, this would be impracticable in larger countries where drug allergy referrals represent an important percentage of the day-to-day clinic. Moreover, it could potentially affect access to desensitization for many patients who would not be able to commute to a distant center regularly.

Other members of the Review Panel consider that all allergists should actively educate themselves in the management of drug allergy (including high-complexity drug allergy), not only as part of the training curriculum but also as continuous professional development. In addition, the allergist should be a driving force making sure that patients are being referred, or otherwise we may not be offering essential services to patients in need. But, of course, simply reading this consensus would not substitute the need for adequately certifying knowledge and expertise and practical training at an expert center.

Acknowledging the previously mentioned differences, we believe there are 2 distinguishable successful organizational models for dealing with these high-risk and high-complexity procedures:

On the one hand, the RCHU's Technical Area (supplementary text 1) considers the allergy department as a whole and does not segregate it into isolated “units” or “centers” dedicated to specific conditions. Consequently, it has access to a larger staff pool and flexible (can be adapted locally) multipurpose spaces with varying degrees of supervision and resources for different patients and procedures, depending on risk and complexity.

On the other hand, the Danish Anaesthesia Allergy Centre, a highly specialized center focused on dealing with one specific high-risk and high-complexity condition (perioperative anaphylaxis) at a national level.^268^ A similar case is the Catalan Institute of Oncology (ICO)/Bellvitge University Hospital Drug Desensitization Center in Barcelona,^35^^,^^36^ a highly specialized service fully integrated into a dedicated oncology center that receives patients from the ICO's referral network. This center is fully staffed with expert allergy doctors and nurses but is separated from the main allergy department (in another hospital). In this case, the reason to have an isolated focused unit is that it is a satellite branch of the main allergy department based on another hospital.

It seems sensible that highly populated countries with larger hospitals and more referrals may benefit from the RCUH approach, potentially more cost-effective in terms of staff and spaces under such circumstances. Indeed, the RCUH model is flexible enough to allow for different allergy departments within a given country with an allergy technical area adapted to their diverse needs (ie, one department may be specialized in mastocytosis, whereas another one may focus on occupational asthma, perioperative anaphylaxis or food desensitization). In contrast, smaller countries with fewer referrals, or satellite centers, may benefit from the Danish Anaesthesia Allergy Centre or the ICO/Bellvitge DDC model. In any case, we would recommend a case-to-case approach, as so many factors could be involved in the decision-making process (Section 2).

### *In vitro* testing in beta-lactam allergy

The reviewing panel for this manuscript recommended adding a summary of the current knowledge on *in vitro* testing for beta-lactam allergy. The main *in vitro* tests for evaluating immediate reactions to beta-lactams (BL) are the sIgE and the BAT.^124^^,^^269^^,^^270^

Regarding BLs-sIgE, the fluorescent-enzyme-immunoassay (ImmunoCAP®, Thermo-Fisher, Uppsala, Sweden) is the commercial method most widely used, although it is available only for a limited number of BLs (benzylpenicillin [penicillin G], penicillin V, amoxicillin, ampicillin, and cefaclor). However, its sensitivity is low (as low as 0%–23% in some studies) and variable depending on clinical manifestations.^271^ To improve sensitivity, some authors have lowered the cutoff point from 0.35 to 0.1 kU/L; however, this can reduce specificity, particularly in subjects with a total IgE > 200 kU/L, so a sIgE/total IgE ratio ≥0.002 has been proposed and confirmed as a way to increase specificity.^147^^,^^272^^,^^273^ Moreover, false-positive test results with ImmunoCAP® have been reported particularly for penicillin V.^274^^,^^275^

BAT is helpful in research, and can be used to assess immediate reactions to BL.^276^, ^277^, ^278^, ^279^, ^280^ BAT is particularly useful for those BLs with no other diagnostic methods available, such as clavulanic acid,^281^, ^282^, ^283^, ^284^ or cefazolin.^285^ BAT sensitivity usually ranges from 22% to 55% for penicillins, and up to 55% for clavulanic acid,^282^ whereas specificity ranges from 79% to 96%.^34^^,^^124^^,^^286^

It is crucial to perform *in vitro* testing relatively soon after the index reaction to avoid the decrease of sensitivity of both sIgE and BAT over time.^124^^,^^287^

*In vitro* testing has shown to be helpful in combination with ST,^288^^,^^289^ especially to improve the sensitivity of the allergy workup, reducing the use of DPT in patients with specific profiles. For instance, DPT could be avoided in those patients who show negative results to ST, but positive results to sIgE or BAT, after experiencing a clear-cut history of immediate hypersensitivity reactions to BLs such as penicillins, and cephalosporins, and clavulanic acid.^282^^,^^290^, ^291^, ^292^, ^293^ Moreover, recommending these tests in subjects with a history of severe anaphylaxis may reduce the need for ST.^294^^,^^295^ Additionally, *in vitro* testing for beta-lactams can be used both in research and in the clinical assessment of non-immediate reactions, but this exceeds the purpose of this document.^34^^,^^296^^,^^297^

### Warnings regarding direct oral drug provocation test (or challenge) with beta-lactams

Several Review Panel members recommend caution with direct oral DPT because it has been studied in selected populations of reactors sharing specific characteristics. Future studies may be needed to standardize the role of direct oral DPT in patients with other profiles, for example, in patients with a history of immediate hypersensitivity reactions, who may benefit from using techniques like ST or *in vitro* testing.^34^^,^^77^ In addition, there is no unified definition of low risk, and many define low risk by clinical history alone, which has its limitations when patients cannot recall necessary data or when the pharmacy department cannot check records for different reasons.

In this regard, the Reviewing Panel of this document has recommended mentioning a recent Position Paper by the European Academy of Allergy and Clinical Immunology on the diagnosis of DHRs to beta-lactams, which classified index reactions as immediate and nonimmediate and patients as low- and high-risk.^295^ Of note, patients who experienced immediate urticarial reactions to beta-lactams, especially within the first hour after exposure, were classified as high-risk, while those who had delayed-appearing urticarial eruptions were classified as low-risk.

To date, there is no consensus on the risk stratification of patients reporting urticarial eruptions associated with beta-lactam therapy.^24^^,^^161^ Some authors have classified patients who experienced only cutaneous symptoms as medium risk.^24^ However, referring to penicillin immediate reactions, they have considered “extensive” urticaria a severe reaction and “isolated” urticaria a non-severe presentation. Other authors have defined urticaria and delayed maculopapular exanthema as benign cutaneous reactions and classified subjects with such skin eruptions as low-to-medium-risk.^161^

Nevertheless, the chronological criterion (ie, immediate or nonimmediate) is relevant for appropriate risk stratification of patients reporting urticarial eruptions associated with beta-lactam therapy, especially considering the widespread implementation of delabeling strategies based on direct oral penicillin DPT. In subjects reporting immediate urticaria, direct DPT without ST with suspected beta-lactams could be potentially very harmful. Therefore, in these subjects, immediate-reading skin testing should be performed first, while the DPTs should only be considered in the event of a negative skin test result.

### Concerns with the quality of articles being published in the field

Members of the Reviewing Panel were concerned about the lack of confirmatory testing in many published articles on intravenous drug allergy, potentially leading to false assumptions. Journals and reviewers are responsible for maintaining high publication standards and should request data on confirmatory DPT in submitted papers (or an appropriate explanation of why the authors did not perform DPT).

Of course, the reviewers and the authors acknowledge that not all centers may have access to the resources needed for systematic DPT (provided risk assessment is favorable) and that real-life clinical practice may differ from a research project. However, when this is the case, said centers should then reconsider whether they should be managing these patients and publishing their data or whether they should be referring them to a more specialized center.

## Conclusions

In this document, some of the leading groups in drug allergy have generously shared their experience and local approaches to the topic of intravenous desensitization and delabeling. Some points of view may be controversial and even slightly clash with one another. However, the reader will appreciate the main learning points from this. Namely, delabeling and desensitization are invaluable techniques in drug allergy. Evidence suggests that these techniques are cost-effective, improve the quality of life of many patients, and even help cancer patients who experience hypersensitivity reactions to their treatments achieve the same survival rates as non-reactive patients. However, delabeling and desensitization are high-risk and high-complexity allergy-specific techniques. As such, they need specific resources and specific spaces led and managed by experts in drug allergy. Local variation is inevitable, and we should learn to embrace this and celebrate it, provided procedures, pathways, and risk assessment are adequately validated and based on current evidence.

## Abbreviations

AAAAI, American Academy of Allergy, Asthma, & Immunology; AAL, antibiotic allergy label; AMR, antimicrobial resistance; AMS, antimicrobial stewardship; ASP, antimicrobial steweardship program; AUC, area under the curve; BAT, basophil activation test; BL, beta-lactam; BSACI, British Society for Allergy and Clinical Immunology; BWH, Brigham & Women's Hospital; CI, confidence interval; CRR, cytokine release reaction; DHR, drug hypersensitivity reactions; DDC, drug desensitization center; DMCD, direct mast cell degranulation; DPT, drug provocation testing or drug challenge; DRESS, drug reaction with eosinophilia and systemic symptoms (DRESS); EAACI, European Academy of Allergy and Clinical Immunology; e.g., for example; ICD, International Classification of Diseases; ICO, Catalan Institute of Oncology; ICU, intensive care unit; I-DHR, immediate drug hypersensitivity reactions; IDSA, Infectious Diseases Society of America; IDT, intradermal testing; i.e., in other words; IgE, immunoglobulin E; IM, intramuscular; IP, intraperitoneal; IV, intravenous; MDM, multidisciplinary team meeting; MDT, multidisciplinary team; MGH, Massachusetts General Hospital ; Mg, milligram; Ml, milliliter; MRSA, methicillin-resistant *Staphylococcus aureus*; NI-DHR, non-immediate drug hypersensitivity reactions; NPV, negative predictive value; NSAID, non-steroidal anti-inflammatory drug; OIIS, oxaliplatin immune-induced syndrome; RA, risk assessment; RDD, rapid drug desensitization; RCUH, Ramon y Cajal University Hospital; SCARs, severe cutaneous adverse reactions; sIgE, specific immunoglobulin E; SJS, Stevens-Johnson's Syndrome; SPT, skin prick testing; ST, skin testing; TEN, toxic epidermal necrolysis; USD, United States Dollars; WAO, World Allergy Organization

## Author contributions

Alvarez-Cuesta (Chair of the Task Force), Ansotegui (WAO Past President), and Madrigal-Burgaleta (Secretary of the Task Force) contributed to conceiving, designing, editing, and revising the manuscript. Steering Committee authors Alvarez-Cuesta, Madrigal-Burgaleta, Broyles, Cuesta-Herranz, Guzman-Melendez, Maciag, Phillips, Trubiano, and Wong performed the literature research and drafted their specific sections.

Alvarez-Cuesta & Madrigal-Burgaleta co-authored Sections 2, 3, 4, 6, 7, 2, 3, 4, 6, 7, 2, 3, 4, 6, 7, 2, 3, 4, 6, 7, 2, 3, 4, 6, 7. Phillips & Trubiano co-authored Section 5. Wong authored Section 8. Cuesta-Herranz & Guzman-Melendez co-authored Section 9. Broyles & Maciag co-authored Section 10. All the Steering Committee authors accepted the final version of the manuscript. Any supplementary material was kindly provided individually by the authors of each section.

A Review Panel of expert allergists from all around the globe critically reviewed the manuscript and accepted the final version of the manuscript. All the review panel members sent a review with modifications to the manuscript that were duly implemented and equally contributed to the discussions collected in Section 11.

## Ethics approval

Ethics approval not applicable.

## Consent for publication

All authors gave their consent to publish this work.

## Availability of data and material

Not applicable.

## Funding

The World Allergy Organization provided funding for the open access publication of this article. Otherwise, there was no other source of funding for this article.

## Declaration of competing interest

The authors report no competing interests with regards to this document.
