# Supplementary material for: Standards for practical intravenous rapid drug desensitization & delabeling: A WAO committee statement
Source: World Allergy Organ J. 2022 May 31;15(6):100640. doi: 10.1016/j.waojou.2022.100640 (PMC9163606; doi:10.1016/j.waojou.2022.100640)
Supplement: Multimedia component 1 [file mmc1.pdf]

SUPPLEMENTARY TEXT 1

Technical Area for Diagnostic and Therapeutic Procedures in an Allergy Department

Emilio Alvarez-Cuesta MD, PhD

Former Head, Allergy Division, Ramon y Cajal University Hospital, Madrid, Spain

Ricardo Madrigal-Burgaleta MD, PhD

Allergy & Severe Asthma Service, St Bartholomew's Hospital, Barts Health NHS Trust, London, UK

INTRODUCTION

The main manuscript of this consensus paper includes some notions on the blueprint -the "DNA"- of an allergy department at a university hospital level. It involves its "culture" (politics, experience, and practice) and the distinctive traits of its added value, unique selling propositions, and other aspects. For instance, some may prioritize multidisciplinary and transversal teamwork when tackling high complexity and high-risk conditions and a personalized approach to patient care (individual diagnosis, monitoring, and treatment).

This supplementary text 1 will focus on the "foundation stones" of an allergy department: the Technical Area for Diagnostic and Therapeutic Procedures in Allergy (Technical Area) and the Clinical Research Unit[1]. Strikingly, it is virtually impossible to find any published documentation on this. Hence, we deemed it appropriate to dedicate a whole supplement to explaining how to develop these areas based on a practical, real-life model, that of the Technical Area of the Allergy Division at the Ramon y Cajal University Hospital (RCUH), Madrid, Spain[1]. Furthermore, over the years, we have received countless requests for information on the specifics of the design of our Technical Area, which motivated us to share our experience, which we will develop further below.

We structured this supplement in three main sections, namely, (i) the "fundamental pillars" of a Technical Area; (ii) the "cornerstones" of a Technical Area; and (iii) the "mainstays" of a Technical Area. Finally, we will conclude with some final touches on the practicalities of the day-to-day management of the Technical Area and the Clinical Research Unit. A figure with a schematic design of the area will help the readers visualize the spaces.

FIGURE: A SCHEMATIC DESIGN INSPIRED BY RAMON Y CAJAL UNIVERSITY HOSPITAL'S (RCUH) ALLERGY DEPARTMENT'S TECHNICAL AREA FOR DIAGNOSTIC AND THERAPEUTIC PROCEDURES.

A) Allergy Technical Area (TA)

B) Adjacent Spaces to the South of the TA

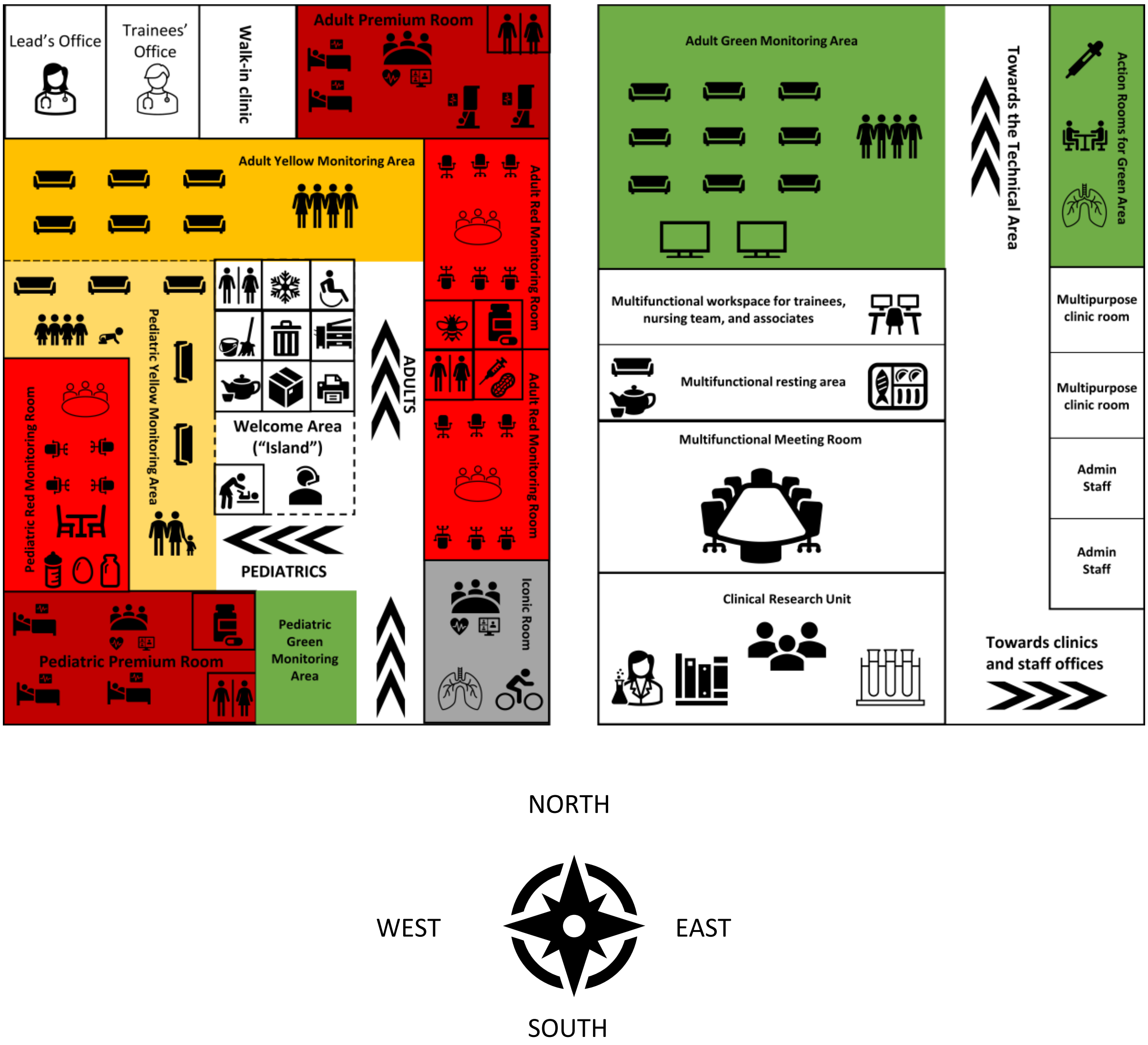

LEGEND: Part A) shows the blueprint for the Technical Area. Part B) show the immediately adjacent areas. See the text for further explanations. Please, be aware that this schema is only a design inspiration for the reader. This figure is not trying to reliably reproduce the RCUH's allergy Technical Area blueprint. For instance, the dimensions of the rooms are not accurate and have been modified for the sake of clarity. In addition, some rooms or their location might be still a design idea not yet realized.

THE FUNDAMENTAL PILLARS OF A TECHNICAL AREA

We understand the Technical Area as the "powerhouse" or "focal point" of an allergy department, based on its crucial role in clinical services, medical education, research, prevention/outreach, and disseminating the generated knowledge.

Inevitably, the Technical Area must be patient-centered. Therefore, patient satisfaction, patient education, and the patient's comprehensive care must be staples of the Technical Area, focusing on high-risk and high-complexity patients. In other words, the patient must receive from us the best we can offer, both clinically (e.g., world-class diagnostic and therapeutic procedures) and personally (e.g., offering easy access to our care by opening 12 hours a day, six days a week, and offering personalized fast-track services for patients with selected conditions).

The Technical Area should be the "motor," the "image," and the "flagship" of an allergy department, and it should be a true reflection of its solidarity and empathy with patients, of its medical care, of its collaborators and partners, of its community, and even of the efforts of the department in continuous quality improvement and innovation.

In a way, the Technical Area should be the "standard-bearer" of the quality indicators, namely, (i) safety (both patient and staff); (ii) effectiveness; (iii) patient satisfaction; (iv) efficiency; (v) accessibility; and, (vi) equity.

Last but not least, it would be optimal to integrate the Clinical Research Unit within the Technical Area or, if impossible, in an adjacent area.

The Allergy Division at RCUH, Madrid, Spain, redesigned its Technical Area around ten years ago. All these principles were considered then, even before World Allergy Organization (WAO) consensus documents on risk and safety requirements were published[1,2].

Structural resources, location, design, and organization

We will focus on a real-life example, the RCUH Technical Area, one possible model that could surely admit improvements and adjustments depending on local circumstances. The hospital assigned a suitable sector of the building for the Technical Area that would favor spaciousness, accessibility, and natural lighting. Necessarily, we divided this space into two well-defined sections: adult allergy patients and allergy pediatric patients. The calculations for the total area of the Technical Area and each section considered a series of parameters, including the number of patients, the needs of the local community patients, the services provided, the different specific programs awaiting further development (e.g., drug desensitization program, mastocytosis program, or immunotherapy program, amongst others), and the future provisions for the department.

As mentioned above, spaciousness, functionality, and natural lighting were prioritized factors in the design. The ground plan was a single rectangle (**see the figure for a schematic visual guide**). The section for adult patients takes up a long East-facing arm located on the right-hand side when entering the Technical Area. The short arm facing North at the back of the rectangle harbors the high-risk areas for adult patients and offices for staff and trainees. The Southern short arm of the rectangle accommodates the entrance to the Technical area and some rooms for the pediatric section. The long West-facing arm, located on the left-hand side when entering the Technical Area, accommodates most of the pediatric section.

In the center of the rectangle, an "island" that incorporates the welcome area, space for clerical personnel, storage space, the clean utility room, the dirty utility room, toilets (different toilest for staff, adult patients, and pediatric patients), as well as an area for fridges and freezers.

The design of the Technical Area is based on specific fundamental parameters, namely, patient safety ("safety first") and adequate programming to render it functional and efficient. The adult patients' and pediatric patients' modules will each comprise different rooms: waiting areas, action areas, monitoring/observation/recovery areas, "premium rooms," and "iconic rooms."

These monitoring/observation areas are classified into three safety levels (or risk levels), green, yellow, and red. Accordingly, we will have a given area with a specific safety level at our disposal, depending on the complexity of the procedure at hand and the patient's risk assessment. As a result, we will adjust the resources and staffing assigned to every procedure. We will now delve into the characteristics of the areas with some unique features.

The "premium rooms," both for adult and pediatric patients, are multipurpose action areas where monitoring and observation are intense and strict. They constitute an upgrade in safety when compared with red areas. We can assign patients stratified to the highest level of risk or patients undergoing high-complexity or very specialized techniques to these areas. Or, beyond clinical practice, these areas can be used as the epicenter of the Clinical Research Unit (Phase I, II, and III studies).

For instance, in the premium rooms of the Technical Area at Ramon y Cajal University Hospital, adult patients mostly undergo high-risk and high-complexity drug challenges (e.g., intravenous drugs or inpatients) and drug desensitizations. In contrast, pediatric patients mostly undergo high-risk food challenges and desensitization to foodstuff. Another example of very high-risk or 'special risk' patients that may need the premium room are beekeepers with mastocytosis and bee venom allergy in

need of subcutaneous specific immunotherapy. Premium rooms are bedded areas, and even if all these procedures are booked as outpatient slots, patients can occupy their beds for up to 14 hours.

The premium rooms at Ramon y Cajal University Hospital Technical Area resemble a semi-intensive care unit and are certainly fully equipped to treat anaphylaxis. The adult module could fit up to 4-6 beds, whereas the pediatric module could fit 2-3. These modules resemble open wards (with no wall partitions), so these rooms can be constantly supervised (the nurse:patient ratio should be 1:1), with the leading nurse or a senior allergist-in-training operating the central monitoring center, with screens displaying the vital signs for each patient. These monitors continuously and non-invasively record heart rate, respiration rate, oxygen saturation, and blood pressure. Each module (both pediatric and adult) should have a crash cart, adequately sealed and daily revised to monitor and document all the material. The beds include a headboard with outlets for oxygen and vacuum. Specifically for drug desensitization, there are programmable multi-step infusion pumps and, for chemotherapy, specific drug disposal bins and equipment to handle potential leakage of hazardous drugs.

These Premium areas were placed on the angles of the rectangle to facilitate their multipurpose nature. The premium rooms should have a toilet of their own (this is important for the safety of high-risk patients and, in the case of chemotherapy, for the adequate disposal of detritus). They should also have a changing room for the patients, with individual lockers. The working area for nurses and doctors should be functional and include the necessary tools (telephone, printer, computer, space for drug/food preparation). In our experience, it is beneficial to grant patients access to television screens and WiFi for further comfort and quiet entertainment during the long hours of treatment.

As mentioned before, the monitoring/observation areas are subdivided into green rooms (for low-risk patients), yellow (medium-risk patients), and red (high-risk patients). In addition, the different monitoring areas have access to specific resources and staffing. Namely:

The "red monitoring rooms" are reserved for high-risk patients, and, in the case of the Technical Area at Ramon y Cajal University Hospital, they acted as both action and monitoring rooms. The specific provision for these rooms includes comfortable and broad recliner chairs for patients (which can go flat in case of a severe reaction), vital signs monitors, a licensed resuscitation stretcher, outlets for oxygen and vacuum, and other devices (tensiometers, pulse oximeters). Furthermore, these provisions should allow for constant supervision by a highly specialized nurse. These rooms should be in immediate proximity

to the areas where the allergists are physically supervising the Technical Area. A nurse:patient ratio of 1:1 is still advisable for these high-risk procedures, although patients can potentially be staggered, and nurse:patient ratio decreased to 1:2, depending on factors like the risk assessment and the complexity of the techniques. Commonly, patients remain under observation on finalizing the active part of their procedures, and nurses could then redeploy to other sections of the Technical Area as required. However, there needs to be constant supervision by at least a highly specialized nurse at all times (ideally accompanied by an allergist-in-training or a lower rank nurse, to be able to quickly act and call for help in case of a reaction). In addition, the area needs to be ready for patients needing particular isolation protocols (e.g., patients with cystic fibrosis or hematological patients). The nursing team should have a specific workspace and a staff toilet.

The "yellow monitoring areas" are reserved for medium-risk patients and located near the "red monitoring rooms," the "premium rooms," and the public toilets (which need to be within sight of the staff, to ensure constant monitoring of patients and to prevent patients from wandering around the hospital in search of a toilet). These multipurpose observation areas, ideally furnished with comfortable chairs, should be monitored by staff at all times. These patients are actioned in a specific room and, once actioned, are assigned to the yellow monitoring area for observation. An example of patients who could have to wait in these areas are patients receiving specific immunotherapy with a medium-risk assessment (e.g., previous reactions, cluster induction phases, or patients with risk factors like asthma). As another example, we could find patients undergoing a lower-risk drug challenge (drug challenges, even the lower-risk ones, are considered to be, at least, a medium-risk procedure and are assigned to, at least, "yellow monitoring areas").

The "green monitoring areas" are similar to general waiting areas and don't need to be located within the Technical Area but should be immediately adjacent. These areas can hold patients with a low risk of reaction (such as patients undergoing standard skin testing with inhalants, basic lung function testing, and other low-risk procedures).

An example of an "iconic room" would be a room for allergen-specific bronchial provocation, featuring different chambers depending on the condition under study and with a specific focus on occupational asthma. These can potentially include chambers to study occupational asthma or exposure chambers to inhalant allergens for clinical research. A focus on conditions such as occupational asthma would entail that the area would need to be equipped accordingly: spirometry (including all the necessary tools such as calibration, mouthpieces, or nose clips), stadiometer, scale, tensiometer,

stethoscope, pulse oximeter, timers, fractional exhaled nitric oxide (FeNO) test, electrocardiogram (ECG), rhinoscope, inspection penlight torch, head-mounted lights, rhinomanometer, nasal peak inspiratory flow meter, nasal nitric oxide measurement, impulse oscillometry system, stationary bicycle or treadmill, toilet, and a work station with computers, printers, telephone. These areas need unique design and maintenance. Some Iconic rooms may require a fixed ventilation system providing around 12-14 changes of air per hour (which will probably need a roof outlet) or, depending on needs, negative pressure rooms. These features need careful planning alongside the hospital's building team, including details like the electricity requirements.

The different sections of the Technical Area will need rooms for cleaning and disinfecting instruments and surfaces. These rooms need a wide sink, sufficient ventilation, and air extraction (especially when using strong disinfectants). Likewise, it needs a cleanroom to store linen, equipment, and anything that needs storage.

We also recommend having clinic rooms available in the space to facilitate seeing patients with adequate resources and privacy, supporting the Technical Area and the Clinical Research Unit. It is necessary to consider the need for offices for staff, allergists-in-training, a multi-functional rest area for the nursing team, and the required space for clinical trials volunteers (for resting, eating, meeting) or other outreach activities, such as patient education activities.

The ideal location for the Technical Area is as close as possible to the Intensive Care Units (both adult and pediatric), adjacent to adult and pediatric allergy clinics (to make patient processes more efficient and minimize duplication of staff). In addition, the space adjacent to the Technical Area should include a room for educational meetings, clinic rooms for the nursing team, and a spacious multipurpose workroom.

We must emphasize that the infrastructure of the Ramon y Cajal University Hospital Technical Area was designed so it could be used by the allergy department's high-risk and high-complexity patient care unit, as well as the allergy department's Clinical Research Unit. This Clinical Research Unit aims to support the researcher in developing and carrying out research projects. If we want to make this feasible, the unit should give access to the researchers to a workspace prepared for their needs, allowing them to carry on with their activities efficiently and safely. In other words, the Clinical Research Unit should focus on the researcher-clinician. Funding for the Clinical Research Unit may come from research grants and other research funding avenues.

Near the Technical Area, there should be an allergy-dedicated translational research laboratory.

Hospitals providing care to only medium or low complexity patients may not have access to all these features. However, it is desirable that any allergy department designs and provides their Technical Area to the best of their capabilities and the highest standards, including an area for clinical research adapted to their local singularities.

Human Capital

Given the Technical Area's importance within the allergy department, it must be managed by a specific "Technical Area Lead", a senior allergist in a permanent position affiliated to the allergy department with full-time specific dedication to the Technical Area. The motto of the Technical Area Lead could be: "my work is my commitment; I force myself to improve the quality of life of patients, not to merely report and document". The rubric could read: "we don't operate through enforcing, but we co-operate through shared knowledge and participation".

The said Technical Area Lead, or another supporting allergist in a permanent position affiliated to the allergy department, will directly supervise the diagnostic procedures, the interpretation of results, and the therapeutic interventions. The said lead, or direct collaborators, will be physically available within the Technical Area during the 12 hours the Technical Area remains open.

When selecting the ideal candidate for the post of Technical Area Lead, we will consider different characteristics: human qualities, specific knowledge (highly qualified), relevant skills set (clinical, technical, and managerial). In addition, we will define what level of dedication the Technical Area will need, and we will consider whether the prospective candidates to the lead role will be able to rise to the challenge, discussing what contribution they will make during this specific moment in their lives.

When assessing prospective leads, we need to consider which personal form of leadership the figure of the Technical Area lead should foster. In the first quarter of the 21st century, investing in a trusted and functioning leadership with a proud sense of belonging and good rapport with the institution seems fundamental. We are talking about an approachable leader, open to communicating clearly with others, prioritizing a solid relationship with patients and collaborators, and always showing the utmost respect and care for them.

A trustworthy leadership will transpire through specific actions executed with the necessary mental clarity to make the right decisions and an abundance of energy to resolve the derived proposals successfully, but constantly communicating, listening, and safeguarding. Importantly, communicating why and how the leader chose and resolved each proposal in a relatable, straightforward, and transparent manner. Furthermore, safeguarding patients and their families and being empathic with their different realities and backgrounds. And

above all, listening to what patients, families, and coadjutors have to say.

Another relevant figure will be the Technical Area Lead Nurse, who will need the same virtues, values as the above described Technical Area Lead.

The Technical Area lead allergist will be constantly supported and assisted by at least one fully trained allergist (who should be very well trained and demonstrate outstanding knowledge), an allergist-in-training, and highly specialized nurses. The number of nurses, nurse's aides, and other staff will depend on the complexity and risk of the procedures and patients serviced in a specific allergy Technical Area, the department's services portfolio, and the daily workload. Because the allergy Technical Area remains open for 12 hours, at least two teams are necessary to cover the area (the AM and the PM teams).

Nurses have an essential role in the allergy Technical Area. nurses require specific training, including advanced life support, with competencies in allergy completed through the appropriate professional bodies and engagement in continuous professional development. Therefore, specific training sessions on each of the procedures offered in the Technical Area are essential, and there should be time and resources dedicated to periodic training and educational sessions led by the Technical Area nurses. Nurses also have a fundamental role in outreach to the community, including patient and health education. Thus, dedicated space, time, and resources should be provisioned for these activities.

We thoroughly explained in the main manuscript (section 2: well-grounded know-how: "laying the foundations for the new silk road") our position on the vital role of multidisciplinary teams and how to manage them, as well as how helpful it is to assign one specific fixed team for each different work and research program (e.g., one team specifically for the mastocytosis program, and another team for the desensitization program).

Technical equipment and resources

A multidisciplinary team, led by the allergy Technical Area lead allergist, will list the specific needs, depending on the services provided and other local considerations. At the Ramon y Cajal University Hospital Technical Area, we had a minimalist approach and prioritized equipment and resources to maintain the highest standards, intending to diagnose and manage high-complexity and high-risk, guarantee safety (patients and staff), and meet all the requisites to remain functional at all the required levels.

For example, focusing on the topic of the main manuscript (i.e., intravenous drug provocation testing and drug desensitization), we needed programmable multi-step infusion pumps and, for chemotherapy, specific drug disposal bins and equipment to handle potential leakage of

hazardous drugs. We also considered necessary a central monitoring center with screens displaying the vital signs coming from monitors assigned to each patient (fundamental in the premium areas and desirable in the red monitoring rooms), and equipment for advanced life support (e.g., crash cart, bag valve mask manual resuscitator, tools for tracheal intubation, defibrillator, portable spirometry, ECG and transportation with ECG monitoring, transport ventilator, material for arterial access). Other devices, appliances, or items may include latex-free gloves, disinfecting material, gowns, masks, or protective plastic googles.

An allergy Technical Area with these characteristics cannot lack other more standard needs for allergy testing, to name a few: allergen extracts for skin testing (e.g., airborne or food allergens), lancets for skin prick testing, dermographic pen, disposable razors (e.g., for patch testing in hairy individuals), measuring rulers, stethoscopes, timers for each patient, bibulous paper, adequate syringes for each procedure according to guidelines, or an error-proof system to identify patient samples.

Pharmacological resources

In practice, we would also recommend assigning this task to a multidisciplinary team led by the Technical Area aead allergist. The core of the list of medications needed should be based on the World Health Organization recommendations on essential medicines[3,4]. In addition, the team should add other drugs specific to the Technical Area, such as medications required in the treatment of anaphylaxis[5–7], namely, adrenaline 1:1,000 (1 mg/ml), bronchodilators, glucose 5% and 10% fluids, 4.2% sodium bicarbonate, volume expanders (e.g., depending on the area of the world, saline solution, sodium lactate solution, colloids, albumin), systemic corticosteroids, systemic antihistamines, or glucagon.

Close collaboration with the pharmacy department is essential, and some departments work with a highly-specialized pharmacist with interest in allergy, which will be helpful in the multidisciplinary team, as pharmacist will also need to take care of preparing dilutions for skin testing, or preparing dilutions for drug provocations testing and drug desensitization.

THE "CORNERSTONES" OF A TECHNICAL AREA

1.- To institute a culture of safety and prevention of anaphylaxis. Safety is a crucial feature and measure of the quality of care. Therefore, we opted for personalized risk stratification for each patient before any procedure, a risk management plan for the Technical Area, and a periodic (at least twice a year) update of our anaphylaxis management plans. In addition, this update included a practical approach to staying skilled in managing anaphylaxis and its complications (e.g., simulation of real-life scenarios) and a revision in a specific Morbidity & Mortality meeting of all the cases of anaphylaxis since the previous update meeting, encouraging a critical approach to identify areas for improvement as a team.

2.- To establish a culture of quality, fostering continuous quality improvement. We recommend promoting the essential aspects of healthcare quality: safety, effectiveness, equity, patient satisfaction (anonymized surveys), accessibility, and efficiency. In addition, we recommend carrying out at least basic quality control for all procedures. These basic controls should address the procedure's performance, morbidity & mortality, and we will need to identify the optimal quality, activity, and performance measures and indicators. However, more complex and external quality controls are, of course, desirable.

3.- To instil a culture of knowledge and teamwork (favored by communication, transparency, commitment, making decisions based on general agreement, coordination).

4.- To introduce permanent innovation as the driving force for change and the guarantee of the future.

THE "MAINSTAYS" OF A TECHNICAL AREA

We won't be too extensive on this, but we would like to invite the reader to consider some issues regarding the work philosophy[8] and the management of the Technical Area[1].

The work philosophy of the Technical Area is: "To think globally, to be knowledgeable like a generalist, to act like a super-specialist, and to always bear in mind local singularities."[1] We would recommend prioritizing one-stop care and minimizing waiting times, as well as standardizing all the clinical processes to reduce variation, and establishing a sensible system of red flags.

The Technical Area should be **an example of efficient management**, based on current evidence, auditing (measuring accurate and adequate indicators), and objectives. Efficient management will be the guarantee of the present. We shouldn't see auditing as a corrective tool but, quite the opposite, as an opportunity for improvement. We recommend implementing a management model focused on measuring quality (e.g., quality control of every process or instrumental control).

Standardization: operating rules, daily checklists, register of special procedures, clinical documentation including informed consents, local protocols (should be clear, practical, and straightforward to minimize variation), standard operating procedures, clinical pathways, clinical guidelines, organizational pathways, manual of procedures.

The portfolio of services should be clearly defined, adequate benchmarking should be set, and gold standards should be defined for each program. We fostered Personalized and Precision Medicine (signature procedures and convictions).

Waste disposal should follow specific guidelines. For example, there should be a record of cleaning, disinfection, and sterilization of rooms and materials.

To add other examples of internal protocols: establishing clear code names like blue (for medical emergencies), red (for fire, explosions, or natural catastrophes), or fuchsia (for safety concerns regarding aggression or violence against staff).

We recommend detailed **signposting** of the allergy department and, specifically, of the allergy Technical Area. It is "a must-do"!

Staff should have been assessed and cleared by Occupational Health and have not only all the vaccines included in the national vaccination schedule, but also for other conditions that could potentially put patients or staff at risk when managing patients in this area (such as the flu, hepatitis B, or COVID-19). Liaison with Occupational Health is also essential for issues such as screening and monitoring staff working with hazardous drugs.

FURTHER NOTES ON PRACTICAL MANAGEMENT

1.- Practical Management of the Technical Area (minor final touches)

Patients should arrive into the Technical Area from the (adult or pediatric) allergy clinics with a personalized request specifying what test the patient needs. The request should be inexcusably accompanied by a personalized risk assessment of the patient (depending on the patient's allergy profile, intrinsic and extrinsic factors, or co-factors) and an allocation to the specific room where the patient will undergo the procedure or treatment, and the area where monitoring/observation should be completed. Moreover, the patient will have an information leaflet with warnings and specific recommendations for the procedure. On arrival, the nursing team will double-check that the patient has signed the adequate informed consent.

The practical management of the patient within the Technical Area will be adapted to the personalized protocol suitable for the specific procedure. For instance, allergen-specific immunotherapy[9], drug desensitization[10], or drug provocation testing[11]. In addition, practical management should also be adapted to the specifics of the previous paragraph.

We achieved a wide availability of service supply by a precise organization of all the employees staffing the allergy department. The allergy department should be organized to cover 12-14 continuous working hours. Thus, staff shifts should be coordinated to cover morning and afternoon shifts accordingly. In those 12-14 hours, all the staff should be present at some point to ensure the adequate passing of information (allergists, nurses, and others) from the morning shift to the afternoon shift. In addition, multidisciplinary meetings, teaching sessions, clinical governance meetings, morbidity and mortality meetings, and other departmental meetings should involve all the staff.

Patients should be discharged with personalized recommendations (including, for instance, whether the patient needs to take any medications) and be provided with clear contact details to solve any queries if these should arise.

How will we keep walking in practice towards excellence? In our opinion[1,8,10], creating monographic clinical programs anchored firmly to the Technical Area will help us keep walking in practice towards excellence. These specific programs should be led by a senior allergist in a permanent position within the allergy department. The lead should be supported by a senior allergist in training, a junior allergist in training, and a recently incorporated allergist in training. When the senior allergists in training leave the

department, the junior allergists in training take their place within the program until they have to leave the department, and successively so, making sure that these programs are always adequately staffed. These programs are the foundations for future high-quality scientific publications. As a very successful example, we have repeatedly mentioned and cited in this manuscript the "Drug Desensitization & Delabeling Program" at Ramon y Cajal University Hospital[8,10–18].

As a take-home message, if you wish your project to succeed: choose an attractive plan, ensure efficient management (and you will guarantee the present); foster continuous innovation, high-complexity disease, high-risk patients (and you will secure the future); and, don't forget to persevere, to be resilient, and to conduct a broad, constant, and convincing dissemination of the project.

2.- Practical Management of the Clinical Research Unit

In the interest of this self-sufficient unit's optimal functioning, the administrative and nursing teams for this unit should have everything ready. When the doctor enters the unit, the data logbook should be prepared at the desk in front of the patient. The doctor should be able to listen to the patient, supervise the project, and avoid unnecessary administrative tasks (which should be managed by the specific staff of the unit).

ACKNOWLEDGEMENTS:

The authors would like to express their gratitude and praise Dr Pilar Berges-Gimeno, for her extensive efforts to make this landmark become a reality, and Dr Daniel Alvarez-Cabo, Medical Director of Ramon y Cajal University Hospital at the time, for his unwavering support.

REFERENCES

1. Alvarez-Cuesta E, Berges-Gimeno M, Cuesta-Herranz J. Área de procedimientos alergológicos, diagnósticos y terapéuticos en el siglo XXI: propuesta de un modelo estándar . In: Guzman M, ed. *Alergia e Intolerancia Alimentaria*. Santiago de Chile: Editorial Mediterraneo; 2015:413-423. https://mediterraneo.cl/busqueda?controller=search&order=product.position.desc&s=978-956-220-371-5. Accessed February 11, 2021.

2. Kowalski ML, Ansotegui I, Aberer W, et al. Risk and safety requirements for diagnostic and therapeutic procedures in allergology: World Allergy Organization Statement. *World Allergy Organ J*. 2016;9(1):1-42. doi:10.1186/s40413-016-0122-3

3. The 2019 Expert Committee on the Selection and Use of Essential Medicines. WHO Model Lists of Essential Medicines. World Health Organization. https://www.who.int/groups/expert-committee-on-selection-and-use-of-essential-medicines/essential-medicines-lists. Published 2019. Accessed June 11, 2021.

4. *World Health Organization Model List of Essential Medicines, 21st List, 2019*. Geneva; 2019. https://apps.who.int/iris/bitstream/handle/10665/325771/WHO-MVP-EMP-IAU-2019.06-eng.pdf. Accessed June 11, 2021.

5. Cardona V, Ansotegui IJ, Ebisawa M, et al. World allergy organization anaphylaxis guidance 2020. *World Allergy Organ J*. 2020;13(10):100472. doi:10.1016/j.waojou.2020.100472

6. Dhami S, Panesar S, Roberts G, et al. Management of anaphylaxis: a systematic review. *Allergy*. 2014;69:168-175.

7. Shaker MS, Wallace D V., Golden DBK, et al. Anaphylaxis—a 2020 practice parameter update, systematic review, and Grading of Recommendations, Assessment, Development and Evaluation (GRADE) analysis. *J Allergy Clin Immunol*. 2020;145(4):1082-1123. doi:10.1016/j.jaci.2020.01.017

8. Alvarez-Cuesta E, Madrigal-Burgaleta R, Berges-Gimeno MPP, Angel-Pereira D. Reply. *Allergy Eur J Allergy Clin Immunol*. 2013;68(11):1483-1484. doi:10.1111/all.12228

9. Alvarez-Cuesta E, Bousquet J, Canonica GW, Durham SR, H-j M, Valovirta E. Standards for practical allergen-specific immunotherapy. *Allergy Eur J Allergy Clin Immunol*. 2006;61(suppl. 82):1-20. doi:10.1111/j.1398-9995.2007.01302.x

10. Madrigal-Burgaleta R, Bernal-Rubio L, Berges-Gimeno MP, Carpio-Escalona LV, Gehlhaar P,

Alvarez-Cuesta E. A Large Single-Hospital Experience Using Drug Provocation Testing and Rapid Drug Desensitization in Hypersensitivity to Antineoplastic and Biological Agents. *J Allergy Clin Immunol Pract*. 2019;7(2):618-632. doi:10.1016/j.jaip.2018.07.031

11. Alvarez-Cuesta E, Madrigal-Burgaleta R, Angel-Pereira D, et al. Delving into cornerstones of hypersensitivity to antineoplastic and biological agents: Value of diagnostic tools prior to desensitization. *Allergy Eur J Allergy Clin Immunol*. 2015;70(7). doi:10.1111/all.12620

12. Lopez-Gonzalez P, Madrigal-Burgaleta R, Carpio-Escalona LV, et al. Assessment of Antihistamines and Corticosteroids as Premedication in Rapid Drug Desensitization to Paclitaxel: Outcomes in 155 Procedures. *J Allergy Clin Immunol Pract*. 2018;6(4). doi:10.1016/j.jaip.2017.11.013

13. Ureña-Tavera A, Zamora-Verduga M, Madrigal-Burgaleta R, Angel-Pereira D, Berges-Gimeno MPMPMP, Alvarez-Cuesta E. Hypersensitivity reactions to racemic calcium folinate (leucovorin) during FOLFOX and FOLFIRI chemotherapy administrations: To the editor. *J Allergy Clin Immunol*. 2015;135(4):1066-1067. doi:10.1016/j.jaci.2014.09.045

14. Madrigal-Burgaleta R, Berges-Gimeno MP, Angel-Pereira D, et al. Desensitizing oxaliplatin-induced fever: A case report. *J Investig Allergol Clin Immunol*. 2013;23(6):435-436. http://europepmc.org/abstract/med/24459821.

15. Solano-Solares E, Madrigal-Burgaleta R, Carpio-Escalona LV, Bernal-Rubio L, Berges-Gimeno MP, Alvarez-Cuesta E. Chemotherapy in mastocytosis: Administration issues, hypersensitivity, and rapid drug desensitization. *J Investig Allergol Clin Immunol*. 2017;27(5). doi:10.18176/jiaci.0171

16. Angel Pereira D, Madrigal-Burgaleta R, Berges P, Urena Tavera MA, Rodriguez X, Alvarez-Cuesta E. Desensitization to Methylprednisolone Succinate in a Patient with Multiple Sclerosis. *J Allergy Clin Immunol*. 2013. doi:10.1016/J.JACI.2012.12.1248

17. Berges-Gimeno M, Carpio-Escalona L, Longo-Muñoz F, et al. Does rapid drug desensitization to chemotherapy affect survival outcomes. *J Investig Allergol Clin Immunol*. 2020;30(4):254-263. doi:10.18176/jiaci.0425

18. Madrigal-Burgaleta R, Berges-Gimeno MP, Angel-Pereira D, et al. Hypersensitivity and desensitization to antineoplastic agents: Outcomes of 189 procedures with a new short protocol and novel diagnostic tools assessment. *Allergy Eur J Allergy Clin Immunol*. 2013;68(7). doi:10.1111/all.12105
