# Supplementary material for: Standards for practical intravenous rapid drug desensitization & delabeling: A WAO committee statement
Source: World Allergy Organ J. 2022 May 31;15(6):100640. doi: 10.1016/j.waojou.2022.100640 (PMC9163606; doi:10.1016/j.waojou.2022.100640)
Supplement: Multimedia component 2 [file mmc2.pdf]

SUPPLEMENTARY TEXT 2

Reactions To Subcutaneous Biologics

Javier Cuesta-Herranz MD, PhD
Fundación IIS-Fundación Jiménez Díaz, Retic ARADyAL (RD16/0006/0013), Madrid (Spain).
Dr María Antonieta Guzmán Meléndez
Servicio de Inmunología y Alergias, Hospital Clínico Universidad de Chile, Santiago (Chile).

Etanercept (Enbrel) y Adalimumab (Humira)

Etanercept and adalimumab were the two first TNF-α antagonists used in the treatment of patients with inflammatory and autoimmune disorders. Tumor necrosis factor-alpha (TNF-α) is a cytokine that stimulates the acute phase of inflammation being one of the central mediators of it. Besides etanercept and adalimumab, other TNF-α antagonists have been introduced as drugs (ie, infliximab, certolizumab or golimumab).

Although they are well tolerated in most cases, there are sporadic reports of hypersensitivity reactions in the literature. In these cases, the simplest and most practical approach is to change therapy to an alternative anti–TNF-α drug. Nonetheless, in cases in which there is no suitable alternative, desensitization may be a good option.

The first successful desensitization to adalimumab was described by Rodriguez-Jiménez et al. in 2009 (1). The protocol started with an initial subcutaneous dose of 0.5 mg (1/100) that was gradually increased until a cumulative dose of 44.25 mg was reached. The intervals between doses were 60 minutes and the complete process took 6 hours. After the second and third doses (1.25 and 2.5 mg, respectively), the patient presented an injection site reaction, consisting of skin eruption, edema, and itching. She required no treatment and was able to continue the regimen until the total dose was reached. Quercia et al. (2) described a slower protocol with premedication. A 3-day protocol that consisted of 6 injections on each day spaced by 30 to 90 minutes starting at 1/100 dilution of the final dose and doubling the dose with each step.

Bavbek et al. reported the first successful desensitization to Etanercept in 2011 (3). It was a four-day protocol with premedication. The schedule began with an initial subcutaneous dose of 0.25 mg (dilution 1:100) and the dose interval was 30-60 mn until the final dose of 25 mg was reached. Other reports have documented the successful desensitization to Etanercept (4-5) .

Some years later, Bavbek et al, reported the largest series of patients on desensitization to both adalimumab and etanercept (6). Based on their experience, they proposed standardized protocols for subcutaneous administration in patients sensitized to etanercept (a 3-day protocol) and adalimumab (a 1-day protocol) (7). Desensitization protocols for 50 mg of weekly etanercept and 40

mg of adalimumab were generated as shown in Tables 1 and 2. With this schedule, all patients were able to continue with etanercept, with minor local erythema resolving within 1 to 2 hours. Similarly, all patients were successfully desensitized to adalimumab and maintained on weekly adalimumab for 3 months with premedication. Adalimumab injections were then spaced to every other week without further problems.

Recently, a shorter protocol to Etanercept was reported by de la Varga Martínez et al. (8). They described a protocol that consisted of subcutaneous administration of 8 doses every 15 minutes until reaching a cumulative therapeutic dose of 50 mg.

Table 1
Subcutaneous desensitization protocol with etanercept (for 50 mg/mL, weekly injection)

Time (min)	Dose (mg)	Dilution (Concentration)	Volume Administered (mL)
Days 1, 2 (Monday, Wednesday)			
0	0.50	1:100, (0.5 mg/mL)	1
30	1.0	1:10, (5 mg/mL)	0.2
60	2	1:10, (5 mg/mL)	0.4
90	4	1:10,(5 mg/mL)	0.8
120	8	1:1, (50 mg/mL)	0.16
150	9	1:1, (50 mg/mL)	0.18
Total dose	24.5		
Day 3 (Monday)			
0	0.50	1:100, (0.5 mg/mL)	1.0
30	1.00	1:10, (5 mg/mL)	0.2
60	2.00	1:10, (5 mg/mL)	0.4
90	4.00	1:10, (5 mg/mL)	0.8
120	8.00	1:1, (50 mg/mL)	0.16
150	16.00	1:1, (50 mg/mL)	0.32
180	18.5	1:1, (50 mg/mL)	0.37
Total dose	50		

Table 1 reproduced with copyright permission from Bavbek et al. (7)

Table 2
Subcutaneous desensitization protocol with adalimumab (for 50 mg/mL, weekly injection)

Time (min)	Dilution Concentration (50 mg/mL)	Volume (mL)	Dose (mg)	Cumulative Dose (mg)
0	1:100 (0.5 mg/mL)	1.00	0.50	0.50
30	1:10 (5 mg/mL)	0.15	0.75	1.25
60	1:10 (5 mg/mL)	0.25	1.25	2.50
90	1:1 (5 mg/mL)	0.50	2.50	5.00
120	1:1 (50 mg/mL)	0.10	5.00	10.00
150	1:1 (50 mg/mL)	0.20	10.00	20.00
180	1:1 (50 mg/mL)	0.40	20.00	40.00

Table 2 reproduced with copyright permission from Bavbek et al. (7)

OMALIZUMAB

Omalizumab is a humanized murine monoclonal antibody that prevents binding of human IgE to its high-affinity receptor. It has been used to treat allergic rhinitis, eczema and chronic urticaria; but also to achieve success in difficult cases of Hymenoptera venom immunotherapy or desensitization protocols to drugs and foods (9). Paradoxically, hypersensitivity adverse reactions to omalizumab have been reported in some patients and desensitization protocols to omalizumab have been reported.

The first desensitization to omalizumab was reported by Dreyfus et al. (10). The protocol started with a dose of 7.5 mg, doubling every 30 minutes for a total goal dose of 150 mg. The patient experienced continued pruritus and erythema after desensitization which resolved with pretreatment with ibuprofen. Unfortunately, she developed a serum sickness-like reaction after the seventh dose post-desensitization and therapy was discontinued.

Later, Owens et al. (11) reported successful desensitization to omalizumab in three patients using a 10 step protocol. The starting dose was 0.0625 mg, doubling every 30 minutes to a total cumulative dose of 113 to 190 mg. All three patients had mild to moderate reactions during desensitization but were able to complete the desensitization procedure and continue omalizumab therapy without adverse reactions for 12 months after desensitization. The dose of 300 mg monthly was split in half with a plan for biweekly administration of 150mg to prevent the loss of desensitized state.

Paranjpe et al (12) reported a case of failed desensitization to omalizumab resulting in anaphylaxis in a patient with severe asthma.

Recently, Bernaola et al. (13) reported successful administration of omalizumab by desensitization protocol after systemic reactions in 12 patients. These 12 patients received 97 treatments with omalizumab by desensitization protocol at Brigham and Women's Hospital. All treatments received premedication 30 minutes before desensitization, including cetirizine 10 mg orally, famotidine 20 mg orally, and montelukast 10 mg orally. When indicated, patients also received premedication with aspirin 325 mg orally (for history of flushing, which may be mediated by prostaglandins), zileuton 1200 mg orally (for refractory respiratory problems), ibuprofen 600 mg orally (for pain associated with reactions), and 0.9% normal saline continuously infused throughout desensitization. Ninety-three treatments were completed without any reactions or limited skin symptoms, and only four of the 97 treatments were complicated by systemic symptoms: 2 patients had grade 3 reactions and 2 patients had grade 2 reactions. In this report is described the desensitization protocol and pathway for return to standard injection.

REFERENCES:

1.- Rodríguez-Jiménez B, Domínguez-Ortega J, González-Herrada C, et al. Successful adalimumab desensitization after generalized urticaria and rhinitis. J Investig Allergol Clin Immunol. 2009;19:246-7.

2.- Quercia O, Emiliani F, Foschi FG, Stefanini GF. Adalimumab desensitization after anaphylactic reaction. Ann Allergy Asthma Immunol 2011; 106: 547-8.

3.- Bavbek S, Aydin O, Ataman S, et al. Injection-site reaction to etanercept: role of skin test in the diagnosis of such reaction and successful desensitization. Allergy. 2011; 66: 1256-7.

4.- Hall J, Findeisen J. Successful etanercept desensitization in a patient with severe injection site reactions. J Clin Rheumatol 2013; 19: 407-8.

5.- Fellner MJ, Yohe N. Etanercept urticaria in a patient with psoriasis desensitized using a new method. J Drugs Dermatol 2013; 12: 1168-1169.

6.- Bavbek S, Ataman Ş, Akinci A, Castells M. Rapid subcutaneous desensitization for the management of local and systemic hypersensitivity reactions to etanercept and adalimumab in 12 patients. J Allergy Clin Immunol Pract 2015; 3: 629-632.

7.- Bavbek S, Lee MJ. Subcutaneous Injectable Drugs Hypersensitivity and Desensitization: Insulin and Monoclonal Antibodies. Immunol Allergy Clin North Am. 2017;37:761-71.

8.- de la Varga Martínez R, Gutiérrez Fernández D, Foncubierta Fernández A, et al. Rapid subcutaneous desensitization for treatment of hypersensitivity reactions to etanercept in two patients with positive basophil activation test. Allergol Int 2017; 66: 357-9.

9.- Yong PF, Malik R, Arif S, et al. Rituximab and omalizumab in severe, refractory insulin allergy. N Engl J Med. 2009;360:1045.

10.- Dreyfus DH, Randolph CC. Characterization of an anaphylactoid reaction to omalizumab. Ann Allergy Asthma Immunol. 2006; 96; 624-7.

11.- Owens G, Petrov A. Successful Desensitization of Three Patients with Hypersensitivity Reactions to Omalizumab. Drug Safety 2011, 6: 339-42.

12.- Paranjpe P, Hilton K, Khan DA. Failure of omalizumab desensitization resulting in anaphylaxis in a patient with severe asthma. Ann Allergy Asthma Immunol 2009; 103(5):A124.

13.- Bernaola M, Sahar A, Hamadi BS, et al. Successful administration of omalizumab by desensitization protocol following systemic reactions in 12 patients. J Allergy Clin Immunol Pract 2021 (in press).
