# Supplementary material for: Standards for practical intravenous rapid drug desensitization & delabeling: A WAO committee statement
Source: World Allergy Organ J. 2022 May 31;15(6):100640. doi: 10.1016/j.waojou.2022.100640 (PMC9163606; doi:10.1016/j.waojou.2022.100640)
Supplement: Multimedia component 3 [file mmc3.pdf]

SUPPLEMENTARY TEXT 3

General Approach Prior To Desensitization in Antibiotic Hypersensitivity Reactions

Johnson T. Wong, M.D.

Division of Rheumatology, Allergy and Immunology, Massachusetts General Hospital, Boston, Mass, USA.

As a note, please bear in mind that this supplement shows some practical tools based on the author's experience. This is not intended to act as a prescriptive guideline for drug challenge or desensitization protocols. Local guidelines and guidelines of the corresponding national Allergy Societies should always be adhered to, and protocols should be adapted to the local population, local requirements, and local resources.

A history of hypersensitivity reaction to a particular class or classes of antibiotic(s) presents a challenge to the administration of similar antibiotic(s). The ability to administer the desired antibiotic under those circumstances at the very least will broaden the choice of antibiotics. It may allow administration of the optimal antibiotic based on effectiveness, least side effect profile, availability, and cost.

The general approach under that circumstance should include the following:

Detailed history of the initial reaction.

Description of the reaction, time of onset, severity, duration, and treatment, if available, permits assessment of how likely the reaction was a true hypersensitivity reaction and how likely it is to recur if administered currently or in the near future. Rapid onset supports that the reaction might be an IgE-mediated (Type I) or "pseudoallergic" (non-IgE-mediated) hypersensitivity[1–4]. The author defines rapid onset as within 1-2 doses in the first 24 hours, as my experience is that urticaria, angioedema, bronchospasm, abdominal and back pain may be delayed for several hours. Onset after several days into the course or later are suggestive of delayed hypersensitivity and may include antibody-mediated cell destruction (Type II)[1–4], immune complex/complement-mediated (Type III)[1–4], the various subtypes of cell/cytokine-mediated (Type IV subtypes)[1–4] including the common maculopapular/morbilliform rash, the less common but more serious drug reaction with eosinophilia and systemic symptoms (DRESS), the rare but severe Stevens-Johnson Syndrome (SJS), toxic epidermal necrolysis (TEN), and other undefined clinical pictures. The provider should be cognizant that multiple mechanisms may occur in the same patient either concurrently or at different times. Not all

hypersensitivity reactions have been defined. The type of reaction may dictate the risk stratification approach, such as testing, challenge, desensitization, or avoidance of the desired antibiotic. The severity, duration, and treatment initially applied may predict the severity of the reaction that may occur with a challenge or upon desensitization.

The setting of the initial reaction, if available, may help assess whether the underlying disease/infection such as viral infection was the main or a cofactor in causing the reaction.

The date of the initial reaction, generally available, may permit an estimate of how likely the sensitivity may have resolved. In my experience, the rate progressively lessens over a 4-10 year period for IgE-mediated hypersensitivities for most drugs but a small group continues to maintain their sensitivity.

History of similar antibiotic usage in the interim.

Successful tolerance of interim usage of other members of the same or related family of antibiotic supports that the hypersensitivity either has resolved or cross-sensitivity to other members of the same or related family is low. Documentation and trustworthiness of the interim usage are important and may permit the challenge approach.

It is pertinent to characterize the current need for antibiotics to treat ongoing infection, prophylaxis against infection, or anticipated need for antibiotic in the near and medium-term. This characterization may establish the timeline for risk stratification that may include ST, DPT, and/or desensitization. It also helps in selecting the particular antibiotic. The type of infection, the severity of the infection, the susceptibility of the likely infectious organism, the patient's general medical conditions, and the available resources will determine which antibiotic should be chosen.

Approach to the administration of the antibiotic.

Once the decision is to proceed with a particular antibiotic, several options are available. In most cases, the physician should minimize cofactor(s) that can potentially aggravate reactions such as concurrent administration of narcotics or radiocontrast medium that can lead to direct mast cell degranulation.

Challenge.

A challenge protocol may be appropriate if one or more of the following is true: the original reaction(s) was minor, likely due to the original underlying infection rather than the medication, distant history, had tolerated similar antibiotic(s) in the interim, or the infection is so severe, that any delay in treatment is detrimental.

Challenge may proceed with: (1) Full dose under the usual protocol if low risk. A slower infusion rate may be used for the first dose as long as the dose is finished prior to the next dose. (2) Test dose

(usually 1/10 the full dose) followed by the remainder of the full dose 30-60 minutes later if the test dose is tolerated. This may be pursued if there is adequate time for treatment.

Seek alternative antibiotic if the initial reaction was suggestive of a potentially life-threatening reaction but was IgE-mediated or non-IgE-mediated.

(1) Absolute avoidance. This is particularly the case if the patient had one of the following: TEN, severe SJS, or severe end-organ damage.

(2) Relative contraindication. Proceed only if the situation fits one or more of the following: if there is no reasonable alternative, if the underlying infection is life-threatening/severely damaging, and if resources are available to mitigate the reaction. This may include a reaction history suggestive of serum sickness and mild/modest end-organ damage.

(3) Hematologic changes such as drug-induced hemolytic anemia (DIHA), drug-induced thrombocytopenia (DITP), drug-induced neutropenia (DINP) represent strong relative contraindications. A workaround may be pursued using other members of the same family if the risk is judged to be worthwhile. They may be administered with substantial steroid pretreatment with modulation in the rapidity and severity of the reaction but the risk is rarely outweighed by the benefit in case of infections, though may be helpful for chemotherapy.

(4) Drug fever, morbilliform rash are generally manageable with a modification of the desensitization protocol.

REFERENCES:

1. Weiss ME, Bernstein DI, Blessing-moore J, et al. Drug allergy: An updated practice parameter. *Ann Allergy, Asthma Immunol*. 2010;105(4):259-273.e78. doi:10.1016/j.anai.2010.08.002

2. Broyles AD, Banerji A, Barmettler S, et al. Practical Guidance for the Evaluation and Management of Drug Hypersensitivity: Specific Drugs. *J Allergy Clin Immunol Pract*. 2020;8(9):S16-S116. doi:10.1016/j.jaip.2020.08.006

3. WEISS ME, ADKINSON NF. Immediate hypersensitivity reactions to penicillin and related antibiotics. *Clin Exp Allergy*. 1988;18(6):515-540. doi:10.1111/j.1365-2222.1988.tb02904.x

4. Pichler WWJ. Drug hypersensitivity: Classification and clinical features. UpToDate. https://www.uptodate.com/contents/drug-hypersensitivity-classification-and-clinical-features. Published 2019. Accessed March 11, 2021.
