# Supplementary material for: Standards for practical intravenous rapid drug desensitization & delabeling: A WAO committee statement
Source: World Allergy Organ J. 2022 May 31;15(6):100640. doi: 10.1016/j.waojou.2022.100640 (PMC9163606; doi:10.1016/j.waojou.2022.100640)
Supplement: Multimedia component 4 [file mmc4.pdf]

SUPPLEMENTARY TEXT 4

Beta-lactam Hypersensitivity Reactions

Johnson T. Wong, M.D.

Division of Rheumatology, Allergy and Immunology, Massachusetts General Hospital, Boston, Mass, USA.

This document is not intended to act as a prescriptive guideline for drug challenge or desensitization protocols. The objective of this supplementary text is not to review current evidence but to share personal experience. Local guidelines and guidelines of the corresponding national Allergy Societies should always be adhered to, and protocols should be adapted to the local population, local requirements, and local resources.

Beta-lactam antibiotics are historically the first and most widely used families of antibiotics in the modern medical era. Beta-lactam antibiotics are currently comprised of families of penicillins, cephalosporins, carbapenems, monobactams, and beta-lactamase inhibitors[1]. They work principally by interfering with bacterial cell wall synthesis by inhibiting various penicillin-binding proteins (PBP)[2,3]. A history of sensitivity to either the penicillin and/or cephalosporin families of antibiotics represent the most commonly reported drug reactions and present a major hurdle in treating various infections. Adverse side effects, particularly gastrointestinal (GI), are common. True immune-mediated hypersensitivity reactions of various forms are also common. After 5 or more years, many patients with a history of Type I and the Type IV subtype with late-onset maculopapular/morbilliform rash either become skin test negative and/or tolerate a drug challenge with the involved beta-lactam[4–6]. This suggests that one or more of the following factors may apply: Many patients will lose their sensitivity over time, the initial history may be incorrect, the reaction(s) might not have been true hypersensitivity to the beta-lactam antibiotics, and/or the underlying infection might have been a major contributor to the reaction. However, a small percentage of patients remain hypersensitive even after decades, as evidenced by maintaining a positive skin test to penicillin/metabolites and/or develop a hypersensitivity reaction upon taking a course of beta-lactam.

However, some authors argue that well-characterized Type IV hypersensitivity seems to be a long-lasting condition [7–11], which does not appear to be influenced by the time interval between the last adverse reaction and allergy workup[12]. In patients with a T-cell-mediated allergy to beta-lactams, the long persistence of memory despite strict drug avoidance has actually been found both in vivo and in

vitro[8–11]. Specifically, in a study by Rozieres et al.[11], amoxicillin-specific T cells were still detectable both by ELISPOT and LTT assays for as long as 20 years after the occurrence of maculopapular exanthem. Moreover, some studies demonstrated that very few patients with a T-cell-mediated hypersensitivity to penicillins lose their sensitivity and become skin- or patch-test negative within 5 years[13,14]. In another study[15], all 49 patients with a T-cell-mediated hypersensitivity to penicillins, who were retested from 1 year to more than 10 years after the first allergy examination, continued to be positive.

For an explanation on the different types of hypersensitivity that will be explored now, please see the "general concepts" section of the main manuscript.

Type I (IgE-mediated) hypersensitivity reactions may include any combination of urticaria, angioedema, flushing, bronchospasm, hypotension, abdominal, and/or back pain[16,17]. The symptoms may start within minutes but some may be delayed for several hours. The severity may range from mild, short-lasting and self-resolved to severe anaphylaxis needing emergency room treatment. Type I hypersensitivity reactions for beta-lactam antibiotics, even severe cases, are the most likely to subside over time and most amenable to desensitization[4–6,18]. Skin testing, if available, will be useful in assessing the current hypersensitivity status and dictate whether challenge, avoidance, or desensitization should be pursued[18,19]. Cross-hypersensitivity among different beta-lactam antibiotic families is mainly related to similarities or identities of beta-lactam side-chain structures[20–23]. Cross-hypersensitivity with cephalosporins not sharing a common side-chain is thought to be very low in penicillin-allergic patients (type I hypersensitivity) (<3%)[19,20,22]. The reverse situation, where the primary sensitivity is to a particular cephalosporin, is less clear. Cross-hypersensivitiy appears to be low to other betalactam antibiotics. However, in addition to the index cephalosporin, many tolerated challenge to other cephalosporins (20/24) but 4 reacted to other cephalosporins (2/24 with similar side chains and 2/24 with disparate side chains)[24]. The cross-hypersensitivity between the penicillin and carbapenems was demonstrated to be even lower (<1%)[25–28]. In fact, prospective studies[26,27,29–31], each performed on more than 100 subjects with a well-demonstrated IgE-mediated penicillin allergy, found a rate of cross-reactivity between penicillins and carbapenems lower than 1% by performing skin tests with different compounds (i.e., imipenem/cilastatin, meropenem, and ertapenem) and, in case of negative results, challenges with the related carbapenems.

Type II (Antibody-mediated)[16,17]. Type II hypersensitivity reactions predominantly present as drug-induced hemolytic anemia (DIHA)[32], drug-induced thrombocytopenia (DITP)[33], and/or drug-induced neutropenia (DINP)[34]. In several series, cephalosporins (especially ceftriaxone) and penicillins (especially piperacillin) were among the most common causes of DIHA (with cephalosporin accounting

for up to 50% of one DIHA series)[35–38]. The beta-lactam antibiotics: penicillins (especially piperacillin) and cephalosporins (especially ceftriaxone) were again reported in causing DITP but accounted for a much lower percentage of DITP than DIHA[33,39]. We have known of two cases of cephalosporin-associated DINP and the literature also had case reports to small series induced by penicillin and cephalosporin families[40–43]. Beta-lactam induced neutropenia needed to be distinguished from the majority of cases where the cephalosporins were used to treat neutropenic patients where neutropenia is the preexisting condition. The proposed mechanisms for the Type II reactions include antibodies directed against the drug or drug-associated immune complexes adsorbed on the cell surface (overlap Type III). How long does Type II hypersensitivity to beta-lactam antibiotics persists is unknown. In limited experience, we had encounter patients who developed the reactions years later when the drug was reintroduced when the pattern was not initially recognized. For patients who developed Type II reactions, it is generally recommended to avoid using the same or similar beta-lactam antibiotics. However, in the situation where the infection is severe and an appropriate non-beta-lactam antibiotic is not available, a provider may try using another member of the same or different beta-lactam family. For DINP, Vial, et al. shown that neutropenia recurred in 2 out 21 patients but 19 tolerated the alternative betalactam antibiotics[40]. In our experience, we have only identified one patient. However, in the literature, the cases reported experiencing these reactions seem to be drug-specific, and patients seemingly tolerate alternative cephalosporin. Careful monitoring must be done under challenge or desensitization protocols.

Type III (antigen-antibody immune complex/complement-mediated)[16,17]. Serum sickness (SS) and serum sickness-like reactions (SSLR) are the prototypical Type III hypersensitivity reaction where the antigen-antibody immune complex of the right size deposit into various tissues, often fixing complements and leads to urticaria/non-urticarial rashes, arthralgia, adenopathy, fever, and other symptoms. All beta-lactam groups have been reported to cause SS or SSLR[44–48]. For chemotherapeutic agents that have caused SS or SSLR, we had used an IV desensitization protocol with steroid/antihistamine pretreatment which generally allowed the patients to continue the chemotherapy for a small number of courses with modulation of the symptoms. This is generally not worth doing in cases of antibiotics. Drug-induced hypersensitivity vasculitis may present as palpable purpura, petechia, fever, arthralgia, and/or GI symptoms. It differs from SS in that the small blood vessels showed vasculitis features. It can be seen in a small percentage of patients who received beta-lactam antibiotics[49,50]. There has been no reported attempt at desensitization for drug-induced vasculitis, however, administration of other members of the same family may work. Arthus type hypersensitivity has not been reported for beta-lactam antibiotics.

Type IV (T cells, non-T immune cells, cytokine-mediated) subtypes[17]. Beta-lactam antibiotics have been associated with all four subtypes, including overlaps. The most common and least damaging Type IV subtype is the late-onset exanthematous maculopapular/morbilliform rash and the least damaging. It may be accompanied by superficial desquamation, fever, +/- eosinophilia, and +/- mild end-organ damage. The mechanism may represent an overlap of Type IVb and IVc). Typically the rash starts centrally and spread peripherally, at times clearing centrally even as the peripheral involvement increases (personal observations). The reactions generally resolve over several days to weeks without permanent damage. This maculopapular/morbilliform sensitivity to penicillin and cephalosporin tends to wane and is often lost for patients over time (however, as explained before, some authors argue that well-characterized Type IV hypersensitivity seems to be a long-lasting condition). Skin test generally is negative (5-10% may have concurrent Type I hypersensitivity and be skin test positive) and most patients tolerate a challenge years later. A small percentage remains sensitive.

Cross-hypersensitivity among different beta-lactam antibiotic families for the maculopapular/morbilliform rash is unclear. In our experience, it appears to be higher than for Type I reactions and in the range from 5-15%. However, some data in the literature indicate that even in this type of hypersensitivity the cross-reactivity among beta-lactams is mainly related to side chain similarity or identity[20,23]. In a study of 214 consecutive adults with a well-demonstrated T-cell-mediated hypersensitivity to penicillins, mostly aminopenicillins[51], all participants were negative to skin tests with cefuroxime, ceftriaxone, and aztreonam – which do not share side chain structures with penicillins – and tolerated challenges with them, while 40 subjects (18.7%) were positive to skin tests with aminocephalosporins (i.e., cephalexin, cefadroxil, and cefaclor), which share similar or identical side chains with aminopenicillins. Of the 174 participants negative to aminocephalosporin skin tests, 170 accepted challenges; one reacted to cefaclor. In another study concerning 204 patients with a delayed allergy to penicillins, all participants were skin test negative to carbapenems (i.e., imipenem/cilastatin, meropenem, and ertapenem) and tolerated challenges with them[52].

Less common but potentially serious is the reaction known as ‘drug reaction with eosinophilia and systemic symptoms’ (DRESS)/ ‘drug-induced hypersensitivity syndrome’ (DiHS). This shares many of the characteristics of other type IV reactions but more severe and potentially damaging. The eosinophilia is generally moderately to markedly elevated and the end-organ damage(s) moderate to severe. DRESS reactions have progressed when the causative drug was not initially recognized and readministered within a few months. Stevens-Johnson Syndrome (SJS) with mucosal involvement and exfoliation of deeper layers of skin range from moderate to severe. In toxic epidermal necrolysis (TEN), a relatively rare reaction, there can be excessive shedding of the deep layers of the skin which can be disfiguring, and potentially life-threatening.

The cross-hypersensitivity rate for DRESS, SJS, and TEN among different members of the same or different beta-lactam antibiotic families is poorly known. However, there are studies that assessed subjects with severe cutaneous adverse reactions and positive patch tests and/or skin tests to the responsible beta-lactams by performing allergy tests with alternative ones[51,53,54]. In some of these studies[51,53], subjects displaying negative responses to allergy tests with alternative beta-lactams, underwent challenges with the related compounds and tolerated them. Specifically, in an aforementioned study[51], 5 participants with TEN and 2 with acute generalized exanthematous pustulosis (AGEP) to penicillins, tolerated cefuroxime, ceftriaxone, and aztreonam found negative in skin testing. In a study by Trubiano et al[53], 3 subjects with DRESS and 3 with AGEP associated with penicillins were positive to skin tests with penicillins and negative to those with cefazolin and ceftriaxone; 5 of them underwent oral challenges with cephalosporins (3 with cephalexin and 2 with cefuroxime) and tolerated them.

How long does the hypersensitivity of DRESS, SJS, and TEN persist is also uncertain as the general approach has been the avoidance of the drugs. AGEP is a rare member of the type IV subgroup characterized by neutrophilic pustules that are attributed to T cell inducing neutrophils and eosinophils. In fixed drug eruption (FDE), the rahs reoccurs in the same location and is often associated with post-inflammatory hyperpigmentation. FDE is generally attributed to CD8+ T cells. Amoxicillin and Ceftriaxone have been reported to cause FDE in case reports[55–59].

<u>Other mechanisms and/or combinations.</u> Not all cases of beta-lactam antibiotic sensitivity can be nicely divided into one of the above categories. During intravenous desensitizations to cephalosporins, we have encountered occasional cases where there appeared to be a threshold drug infusion rate. Above this infusion rate the patient repeatedly develop reaction but would subside once we lower the infusion rate below the threshold. This suggests that under certain circumstances, cephalosporin may have direct mast cell degranulation (DMCD) property similar to vancomycin antibiotic. Drug fever may accompany types II, III, and IV. In some cases, patients may develop hypersensitivity that involves several different pathways (Type I and Type IV, Type I and Type III, etc).

<u>Approach to patients with a history of hypersensitivity to beta-lactam antibiotics</u>

- Start with a detailed history of the initial reaction.
    - If the reaction was delayed onset with moderate to severe end-organ damage (TEN, SJD, DRESS, vasculitis), strictly avoid usage of beta-lactam antibiotics in the same

family. If possible, also avoid using beta-lactam antibiotics from a different family, at least among penicillins, cephalosporins, and carbapenems.

- If the reaction was delayed onset with moderate to severe SS or SSLR, avoid usage of the same antibiotic. May consider the usage of an antibiotic from a different beta-lactam antibiotic family (or the same family), if there is no suitable non-beta-lactam antibiotic available and treatment of the infection is deemed crucial. Must monitor closely.
- If the reaction was delayed onset with moderate to severe AIHD, AITP, or AINP, avoid usage of the same antibiotic. May consider the usage of an antibiotic from a different beta-lactam antibiotic family (or the same family), if there is no suitable non-beta-lactam antibiotic available and treatment of the infection is deemed crucial. Must monitor closely.
- If the initial reaction was delayed onset exanthematous maculopapular/ morbilliform rash +/- superficial desquamation, fever, +/- eosinophilia, and +/- mild end-organ damage, skin test with penicillin and the desired beta-lactam antibiotics, if available, to rule out concurrent IgE-mediated sensitivity. Skin test generally is negative (5-7% may have concurrent Type I and be skin test positive). If the skin test is negative or if skin testing is not available, may proceed with the challenge protocol if there has been at least 1-2 years elapsed since the initial reaction. In our experience, patients who tolerated a challenge with 250-500mg of the beta-lactam antibiotic generally will tolerate subsequent courses of a beta-lactam antibiotic in the same family. A small percentage, however, may develop the same or similar reaction upon taking a full course even when they tolerated the initial challenge. Cross-hypersensitivity among different beta-lactam antibiotic families for the maculopapular/morbilliform rash is unclear. In our experience, it appeared to be higher than for Type I reactions and in the range from 5-15%, although some data in the literature indicate that even in this type of hypersensitivity the cross-reactivity among beta-lactams is mainly related to side chain similarity or identity.
- If it’s highly likely that the patient has tolerated the same or a different member of the same family of beta-lactam antibiotics, one may proceed with the challenge or test dose protocol of the tolerated beta-lactam antibiotic.

- If the initial reaction had a rapid onset and was compatible with a type I reaction with any combination of urticaria, angioedema, flushing, bronchospasm, hypotension, abdominal, and/or back pain, and if the patient has not taken a member of the same family of beta-lactam antibiotic family since the initial reaction, then perform the penicillin skin test if available. Even for initial type I IgE-mediated hypersensitivity, skin tests frequently become negative if there has been a five or more year gap. A small percentage will remain skin test positive. If the skin tests are negative, one may then may proceed with a challenge or test dose protocol with the desired beta-lactam antibiotic with low risk. If the skin test is positive, consider alternative antibiotics, including challenge or test dose of a different beta-lactam antibiotic family. If an alternative antibiotic is not suitable or available, then one may proceed with the desired member of the same beta-lactam antibiotic family under the IV or oral desensitization protocol depending on the desired formulation of the antibiotic to be used.
- If the initial reaction was vague or not available, and if there is no interval tolerance of beta-lactam antibiotics, the recommended protocol is the following:
    - Penicillin skin tests should be performed if available.
        - If the skin test is negative, may proceed with the challenge or test dose protocols.
        - If the skin test is positive, then should consider alternative antibiotics, including challenge or test dose of a different beta-lactam antibiotic family. If an alternative antibiotic is not suitable or available, then one may proceed with the desired member of the same beta-lactam antibiotic family under the IV or oral desensitization protocol depending on the desired formulation of the antibiotic to be used.
    - If penicillin skin tests are not available
        - History remote (>5 years). May proceed with a challenge or test dose protocol with a different beta-lactam antibiotic family and one that does not share a common side chain.
        - If the reaction is recent (<2 years). If a suitable non-beta-lactam antibiotic is not available, and no evidence of scarring from severe SJD, TEN, or end-organ damage from DRESS, may proceed with

desensitization protocol, preferable with a different beta-lactam antibiotic family and one that does not share a common side chain.

Penicillin and other beta-lactam antibiotics skin testing

Prick: Penicillin G (10,000U/ml), major determinant: Penicilloyl-polylysine (PPL, Prepen), Ampicillin (25mg/ml). Optional: beta-lactam antibiotic of choice for patient if soluble (25mg/ml). Minor determinants: Penilloate, Penicillioate are not commercially available in the USA.

Intradermal testing: Penicillin G (1,000U & 10,000U), major determinant: Penicilloyl-polylysine (PPL, Prepen), Ampicillin (0.25mg/ml, 2.5mg & 25mg/ml), Optional: beta-lactam antibiotic of choice for patient if soluble (0.25mg/ml, 2.5mg & 25mg/ml). Minor determinants: Penilloate, Penicillioate are not commercially available in USA. However, benzylpenicillin reagents (i.e., benzylpenicilloyl-octa-L-lysine and penilloate) are marketed outside the United States and currently used in clinical practice[60–64].

Beta-lactam antibiotics outpatient challenge protocol:

- Challenge. A challenge protocol in the outpatient setting may be appropriate if one or more of the following is true: the original reaction(s) was minor, likely due to the original underlying infection rather than the medication, distant history, and/or had tolerated similar antibiotic(s) in the interim. Bear in mind that the initial dose may be smaller in certain guidelines and especially depending on risk assessment.
    - Administer beta-lactam antibiotic A (e.g. Amoxicillin) 250mg at time 0 (start)
    - If tolerated, administered 2nd dose of antibiotic A (e.g. Amoxicillin) 250mg at 30min.
    - Alternatively, if one wishes to clear 2 classes of beta-lactam antibiotic families, administer the 2nd beta-lactam antibiotic family antibiotic (e.g. cephalexin) 250 mg in place of the 2nd dose of antibiotic A. But, in case of a reaction, the culprit agent will need further testing for precise identification.
    - Continue to observe for at least 60 min after the 2nd dose at the office and monitor for next several days at home. Patient instructed to take pictures and call for any significant delayed reaction.

    - If the history stated that the initial reaction was delayed for many doses and days, consider continuing a 3-day challenge of 250-500mg once to twice a day for 3 days and monitor for the whole week.
    - If the patient needs the antibiotic to treat an infection, then extend the course to complete the treatment.

<u>Beta-lactam antibiotics inpatient challenge/test dose protocol:</u>

- Challenge. A challenge protocol in the inpatient setting may be appropriate if one or more of the following is true: Patient is already inpatient, the original reaction(s) was minor, likely due to the original underlying infection rather than the medication, distant history, had tolerated similar antibiotic(s) in the interim, and/or the infection is so severe, that any delay in treatment is detrimental. Challenge may proceed with either of the two pathways:
    - Full dose under the usual protocol if time is of the essence. A slower infusion rate may be used for the first dose as long as the dose is finished before the next dose.
    - Test dose (usually 1/10 the full dose) followed by the remainder of the full dose 30-60min later if the test dose is tolerated. This may be pursued if there is adequate time for treatment.

TABLES AND FIGURES

1.- Beta-lactam antibiotic rapid intravenous desensitization protocol[1]

Time (hr:min)	Beta-lactam antibiotic concentration (mg/ml)	Fluid infusion rate (ml/hr)	Beta-lactam antibiotic infusion rate (mg/hr)	Cumulative dose (mg)
0:00	0.0001[2]	60.0	0.0060	0.0015
0:15	0.001	20.0	0.020	0.0065
0:30	0.001[3]	60.0	0.060	0.022
0:45	0.01	20.0	0.20	0.072
1:00	0.01	60.0	0.60	0.22
1:15	0.1	20.0	2.0	0.77
1:30	0.1	60.0	6.0	2.2
1:45	1.0	20.0	20	7.7
2:00	1.0	60.0	60	22
2:15	10	12.5	125	54
2:30	10	25.0	250	117
2:45[4]	10	50.0	500	242
3:00[5, 6, 7]	10	50.0	500	242

[1]Adapted from our original vancomycin protocol[65]

[2]Typical starting concentration for patients with severe systemic reactions to previous beta-lactam antibiotic infusions.

[3]Typical starting concentration for patients with moderate systemic reactions to previous beta-lactam antibiotic infusions. This will save 2 steps and 30min.

[4]Optional step for highly sensitive patients.

[5]Continue at this infusion rate for the remainder of the dosage.

[6]Minimize concurrent narcotic and other direct mast degranulators if possible

[7]May need to stay just below a threshold beta-lactam antibiotic infusion rate the first day and advance as tolerated (infrequently seen for cephalosporin).

1.- Beta-lactam antibiotic rapid oral desensitization protocol[1]

Dose#	Time (hr:min)	Beta-lactam antibiotic concentration (mg/ml)	Volume of suspension or # of tablet or capsule	Cumulative Dose (mg)	Reaction
1(optional)	0:00	Water	10ml	0	
2	0:15	0.1	1 ml	0.1	
3	0:30	0.1	3 ml	0.4	
4	0:45	0.1	10 ml	1.4	
5	1:00	1.0	3 ml	4.4	
6	1:15	1.0	10 ml	14.4	
7	1:30	1.0	30 ml	44.4	
8	1:45	10	6.0 ml	104	
9	2:00	10	12.5 ml	229	
10	2:15	250mg	1 cap or tab	479	
11	6-24hr depending on dosing frequency	250mg	1 cap or tab if dose is 250mg<br>2 if dose is 500 mg		
Thereafter give the desired dose at the desired frequency					

[1]Unpublished protocol

- Open capsule or crush beta-lactam antibiotic tablet 250mg and suspend in 25ml of water to make 10.0mg/ml solution (suspension).
- 10ml of beta-lactam antibiotic 10mg/ml solution add to 90ml of water to made the 1.0mg/ml solution
- 10ml of beta-lactam antibiotic 1.0mg/ml solution add to 90ml of water to made the 0.1mg/ml solution
- Obtain informed consent.
- Examine vital signs, oral mucosa, skin, and chest before the start.
- Shake up each solution well before taking out the appropriate amount with the appropriate size syringe for taking
- Monitor blood pressure, skin, GI side effect, or other adverse symptoms.
- If adverse reaction develops, then repeat that dose or cut down to the previous dose. Lengthen protocol accordingly.
- Stay with the patient until 60 min after the first 250mg tablet or capsule is given.

REFERENCES

1. Letourneau A. Beta-lactam antibiotics: Mechanisms of action and resistance and adverse effects. UpToDate. https://www.uptodate.com/contents/beta-lactam-antibiotics-mechanisms-of-action-and-resistance-and-adverse-effects. Published July 1, 2019. Accessed June 24, 2021.

2. Spratt BG, Cromie KD. Penicillin-binding proteins of gram-negative bacteria. *Clin Infect Dis*. 1988;10(4):699-711. doi:10.1093/clinids/10.4.699

3. Novak R, Charpentier E, Braun JS, Tuomanen E. Signal transduction by a death signal peptide: Uncovering the mechanism of bacterial killing by penicillin. *Mol Cell*. 2000;5(1):49-57. doi:10.1016/S1097-2765(00)80402-5

4. Sullivan TJ, Wedner HJ, Shatz GS, Yecies LD, Parker CW. Skin testing to detect penicillin allergy. *J Allergy Clin Immunol*. 1981;68(3):171-180. doi:10.1016/0091-6749(81)90180-9

5. Romano A, Gaeta F, Valluzzi RL, Zaffiro A, Caruso C, Quaratino D. Natural evolution of skin-test sensitivity in patients with IgE-mediated hypersensitivity to cephalosporins. *Allergy Eur J Allergy Clin Immunol*. 2014;69(6):806-809. doi:10.1111/all.12390

6. Picard M, Paradis L, Bégin P, Paradis J, Des Roches A. Skin testing only with penicillin G in children with a history of penicillin allergy. *Ann Allergy, Asthma Immunol*. 2014;113(1):75-81. doi:10.1016/j.anai.2014.04.017

7. Schnyder B, Helbling A, Kappeler A, Pichler WJ. Drug-induced papulovesicular exanthema. *Allergy Eur J Allergy Clin Immunol*. 1998;53(8):817-818. doi:10.1111/j.1398-9995.1998.tb03985.x

8. Luque I, Leyva L, José Torres M, et al. In vitro T-cell responses to β-lactam drugs in immediate and nonimmediate allergic reactions. *Allergy Eur J Allergy Clin Immunol*. 2001;56(7):611-618. doi:10.1034/j.1398-9995.2001.000115.x

9. Pichler WJ, Tilch J. The lymphocyte transformation test in the diagnosis of drug hypersensitivity. *Allergy Eur J Allergy Clin Immunol*. 2004;59(8):809-820. doi:10.1111/j.1398-9995.2004.00547.x

10. Beeler A, Engler O, Gerber BO, Pichler WJ. Long-lasting reactivity and high frequency of drug-specific T cells after severe systemic drug hypersensitivity reactions. *J Allergy Clin Immunol*. 2006;117(2):455-462. doi:10.1016/j.jaci.2005.10.030

11. Rozieres A, Hennino A, Rodet K, et al. Detection and quantification of drug-specific T cells in penicillin allergy. *Allergy Eur J Allergy Clin Immunol*. 2009;64(4):534-542. doi:10.1111/j.1398-9995.2008.01674.x

12. Romano A, Quaratino D, Di Fonso M, Papa G, Venuti A, Gasbarrini G. A diagnostic protocol for evaluating nonimmediate reactions to aminopenicillins. *J Allergy Clin Immunol*. 1999;103(6):1186-1190. doi:10.1016/S0091-6749(99)70197-1

13. Torres MJ, Sánchez-Sabaté E, Álvarez J, et al. Skin test evaluation in nonimmediate allergic reactions to penicillins. *Allergy Eur J Allergy Clin Immunol*. 2004;59(2):219-224. doi:10.1046/j.1398-9995.2003.00308.x

14. Pinho A, Marta A, Coutinho I, Gonçalo M. Long-term reproducibility of positive patch test reactions in patients with non-immediate cutaneous adverse drug reactions to antibiotics. *Contact Dermatitis*. 2017;76(4):204-209. doi:10.1111/cod.12720

15. Romano A, Viola M, Bousquet PJ, et al. A comparison of the performance of two penicillin

reagent kits in the diagnosis of β-lactam hypersensitivity. *Allergy Eur J Allergy Clin Immunol*. 2007;62(1):53-58. doi:10.1111/j.1398-9995.2006.01272.x

16. WEISS ME, ADKINSON NF. Immediate hypersensitivity reactions to penicillin and related antibiotics. *Clin Exp Allergy*. 1988;18(6):515-540. doi:10.1111/j.1365-2222.1988.tb02904.x

17. Pichler WWJ. Drug hypersensitivity: Classification and clinical features. UpToDate. https://www.uptodate.com/contents/drug-hypersensitivity-classification-and-clinical-features. Published 2019. Accessed March 11, 2021.

18. Solensky R. Penicillin allergy: Immediate reactions. UpToDate. https://www.uptodate.com/contents/penicillin-allergy-immediate-reactions. Published April 19, 2021. Accessed June 24, 2021.

19. Blumenthal K, Solensky R. Allergy evaluation for immediate penicillin allergy: Skin test-based diagnostic strategies and cross-reactivity with other beta-lactam antibiotics. UpToDate. https://www.uptodate.com/contents/allergy-evaluation-for-immediate-penicillin-allergy-skin-test-based-diagnostic-strategies-and-cross-reactivity-with-other-beta-lactam-antibiotics. Published September 26, 2017. Accessed June 24, 2021.

20. Romano A, Gaeta F, Poves MFA, Valluzzi RL. Cross-Reactivity among Beta-Lactams. *Curr Allergy Asthma Rep*. 2016;16(3):1-12. doi:10.1007/s11882-016-0594-9

21. Zagursky RJ, Pichichero ME. Cross-reactivity in β-Lactam Allergy. *J Allergy Clin Immunol Pract*. 2018;6(1):72-81.e1. doi:10.1016/j.jaip.2017.08.027

22. Picard M, Robitaille G, Karam F, et al. Cross-Reactivity to Cephalosporins and Carbapenems in Penicillin-Allergic Patients: Two Systematic Reviews and Meta-Analyses. *J Allergy Clin Immunol Pract*. 2019;7(8):2738. doi:10.1016/j.jaip.2019.05.038

23. Caruso C, Valluzzi RL, Colantuono S, Gaeta F, Romano A. β-Lactam Allergy and Cross-Reactivity: A Clinician's Guide to Selecting an Alternative Antibiotic. *J Asthma Allergy*. 2021;14:31-46. doi:10.2147/JAA.S242061

24. Yuson C, Kumar K, Le A, et al. Immediate cephalosporin allergy. *Intern Med J*. 2019;49(8):985-993. doi:10.1111/imj.14229

25. Kula B, Djordjevic G, Robinson JL. A systematic review: Can one prescribe carbapenems to patients with IgE-mediated allergy to penicillins or cephalosporins? *Clin Infect Dis*. 2014;59(8):1113-1122. doi:10.1093/cid/ciu587

26. Atanasković-Marković M, Gaeta F, Medjo B, Viola M, Nestorović B, Romano A. Tolerability of meropenem in children with IgE-mediated hypersensitivity to penicillins. *Allergy Eur J Allergy Clin Immunol*. 2008;63(2):237-240. doi:10.1111/j.1398-9995.2007.01532.x

27. Gaeta F, Valluzzi RL, Alonzi C, Maggioletti M, Caruso C, Romano A. Tolerability of aztreonam and carbapenems in patients with IgE-mediated hypersensitivity to penicillins. *J Allergy Clin Immunol*. 2015;135(4):972-976. doi:10.1016/j.jaci.2014.10.011

28. Blumenthal KG, Peter JG, Trubiano JA, Phillips EJ. Antibiotic allergy. *Lancet*. 2019;393(10167):183-198. doi:10.1016/S0140-6736(18)32218-9

29. Romano A, Viola M, Guéant-Rodriguez R-M, Gaeta F, Pettinato R, Guéant J-L. Imipenem in Patients with Immediate Hypersensitivity to Penicillins. *N Engl J Med*. 2006;354(26):2835-2837. doi:10.1056/nejmc053529

30. Romano A, Viola M, Guéant-Rodriguez RM, Gaeta F, Valluzzi R, Guéant JL. Brief communication: Tolerability of meropenem in patients with IgE-mediated hypersensitivity to penicillins. *Ann*

Intern Med. 2007;146(4):266-269. doi:10.7326/0003-4819-146-4-200702200-00005

31. Atanasković-Marković M, Gaeta F, Gavrović-Jankulović M, Veličković TČ, Valluzzi RL, Romano A. Tolerability of imipenem in children with IgE-mediated hypersensitivity to penicillins. *J Allergy Clin Immunol*. 2009;124(1):167-169. doi:10.1016/j.jaci.2009.02.031

32. Brodsky R. Drug-induced hemolytic anemia. UpToDate. https://www.uptodate.com/contents/drug-induced-hemolytic-anemia. Published May 15, 2020. Accessed June 24, 2021.

33. Arnold D, Cuker A. Drug-induced immune thrombocytopenia. UpToDate. https://www.uptodate.com/contents/drug-induced-immune-thrombocytopenia. Published September 9, 2019. Accessed June 24, 2021.

34. Coates T. Drug-induced neutropenia and agranulocytosis. UpToDate. https://www.uptodate.com/contents/drug-induced-neutropenia-and-agranulocytosis. Published January 4, 2021. Accessed June 24, 2021.

35. Johnson ST, Fueger JT, Gottschall JL. One center's experience: The serology and drugs associated with drug-induced immune hemolytic anemia - A new paradigm. *Transfusion*. 2007;47(4):697-702. doi:10.1111/j.1537-2995.2007.01173.x

36. Garbe E, Andersohn F, Bronder E, et al. Drug induced immune haemolytic anaemia in the Berlin Case-Control Surveillance Study. *Br J Haematol*. 2011;154(5):644-653. doi:10.1111/j.1365-2141.2011.08784.x

37. Mayer B, Bartolmäs T, Yürek S, Salama A. Variability of findings in drug-induced immune haemolytic anaemia: Experience over 20 years in a single centre. *Transfus Med Hemotherapy*. 2015;42(5):333-339. doi:10.1159/000440673

38. Garratty G. Immune hemolytic anemia associated with drug therapy. *Blood Rev*. 2010;24(4-5):143-150. doi:10.1016/j.blre.2010.06.004

39. Mitta A, Curtis BR, Reese JA, George JN. Drug-induced thrombocytopenia: 2019 Update of clinical and laboratory data. *Am J Hematol*. 2019;94(3):E76-E78. doi:10.1002/ajh.25379

40. Vial T, Bailly H, Perault-Pochat MC, et al. Beta-lactam-induced severe neutropaenia: a descriptive study. *Fundam Clin Pharmacol*. 2019;33(2):225-231. doi:10.1111/fcp.12419

41. Neftel KA, Hauser SP, Muller MR. Inhibition of granulopoiesis in vivo and in vitro by β-lactam antibiotics. *J Infect Dis*. 1985;152(1):90-98. doi:10.1093/infdis/152.1.90

42. Olaison L, Belin L, Hogevik H, Alestig K. Incidence of β-lactam-induced delayed hypersensitivity and neutropenia during treatment of infective endocarditis. *Arch Intern Med*. 1999;159(6):607-615. doi:10.1001/archinte.159.6.607

43. Sharpe A, Mourad BM, Hardwick CJ, Reilly T, Dweck E, Bondarsky E. Oxacillin-induced drug reaction with eosinophilia and systemic symptoms (DRESS). *Am J Case Rep*. 2019;20:345-348. doi:10.12659/AJCR.913748

44. Tatum AJ, Ditto AM, Patterson R. Severe serum sickness-like reaction to oral penicillin drugs: Three case reports. *Ann Allergy, Asthma Immunol*. 2001;86(3):330-334. doi:10.1016/S1081-1206(10)63308-X

45. Wener M. Serum sickness and serum sickness-like reactions. UpToDate. https://www.uptodate.com/contents/serum-sickness-and-serum-sickness-like-reactions. Published February 5, 2021. Accessed June 24, 2021.

46. Khan DA, Banerji A, Bernstein JA, et al. Cephalosporin Allergy: Current Understanding and Future Challenges. *J Allergy Clin Immunol Pract*. 2019;7(7):2105-2114. doi:10.1016/j.jaip.2019.06.001

47. Ponvert C, Perrin Y, Bados-Albiero A, et al. Allergy to betalactam antibiotics in children: Results of a 20-year study based on clinical history, skin and challenge tests. *Pediatr Allergy Immunol*. 2011;22(4):411-418. doi:10.1111/j.1399-3038.2011.01169.x

48. Borish L, Tamir R, Rosenwasser LJ. Intravenous desensitization to beta-lactam antibiotics. *J Allergy Clin Immunol*. 1987;80(3 PART 1):314-319. doi:10.1016/0091-6749(87)90037-6

49. Ortiz-Sanjuán F, Blanco R, Hernández JL, et al. Drug-associated cutaneous vasculitis: Study of 239 patients from a single referral center. *J Rheumatol*. 2014;41(11):2201-2207. doi:10.3899/jrheum.140390

50. Mericliler M, Shnawa A, Al-Qaysi D, Fleisher J, Moraco A. Oxacillin-induced leukocytoclastic vasculitis. *IDCases*. 2019;17. doi:10.1016/j.idcr.2019.e00539

51. Romano A, Gaeta F, Valluzzi RL, Maggioletti M, Caruso C, Quaratino D. Cross-reactivity and tolerability of aztreonam and cephalosporins in subjects with a T cell–mediated hypersensitivity to penicillins. *J Allergy Clin Immunol*. 2016;138(1):179-186. doi:10.1016/j.jaci.2016.01.025

52. Romano A, Gaeta F, Valluzzi RL, et al. Absence of cross-reactivity to carbapenems in patients with delayed hypersensitivity to penicillins. *Allergy Eur J Allergy Clin Immunol*. 2013;68(12):1618-1621. doi:10.1111/all.12299

53. Trubiano JA, Chua KYL, Holmes NE, et al. Safety of cephalosporins in penicillin class severe delayed hypersensitivity reactions. *J Allergy Clin Immunol Pract*. 2020;8(3):1142-1146.e4. doi:10.1016/j.jaip.2019.10.005

54. Bérot V, Gener G, Ingen-Housz-Oro S, et al. Cross-reactivity in beta-lactams after a non-immediate cutaneous adverse reaction: experience of a reference centre for toxic bullous diseases and severe cutaneous adverse reactions. *J Eur Acad Dermatology Venereol*. 2020;34(4):787-794. doi:10.1111/jdv.15986

55. Mizukawa Y, Shiohara T. Fixed drug eruption: A prototypic disorder mediated by effector memory T cells. *Curr Allergy Asthma Rep*. 2009;9(1):71-77. doi:10.1007/s11882-009-0011-8

56. Romano A, Valluzzi RL, Caruso C, Maggioletti M, Gaeta F. Non-immediate Cutaneous Reactions to Beta-Lactams: Approach to Diagnosis. *Curr Allergy Asthma Rep*. 2017;17(4). doi:10.1007/s11882-017-0691-4

57. Jimenez I, Anton E, Picans I, Sanchez I, Quinones M, Jerez J. Fixed drug eruption from amoxycillin. *Allergol Immunopathol (Madr)*. 1997;25(5). https://pubmed.ncbi.nlm.nih.gov/9395009/. Accessed June 24, 2021.

58. Özkaya E, Mirzoyeva L, Jhaish MSH. Ceftriaxone-induced fixed drug eruption: First report. *Am J Clin Dermatol*. 2008;9(5):345-347. doi:10.2165/00128071-200809050-00011

59. Arias J, Fernandez-Rivas M, Panadero P. Selective fixed drug eruption to amoxycillin. *Clin Exp Dermatol*. 1995;20(4):339-340. doi:10.1111/j.1365-2230.1995.tb01338.x

60. Mirakian R, Leech SC, Krishna MT, et al. Management of allergy to penicillins and other beta-lactams. *Clin Exp Allergy*. 2015;45(2):300-327. doi:10.1111/cea.12468

61. Romano A, Atanaskovic-Markovic M, Barbaud A, et al. Towards a more precise diagnosis of hypersensitivity to beta-lactams — an EAACI position paper. *Allergy Eur J Allergy Clin Immunol*.

2020;75(6):1300-1315. doi:10.1111/all.14122

62. Brockow K, Garvey LH, Aberer W, et al. Skin test concentrations for systemically administered drugs - An ENDA/EAACI Drug Allergy Interest Group position paper. *Allergy Eur J Allergy Clin Immunol*. 2013;68(6):702-712. doi:10.1111/all.12142

63. Torres MJ, Blanca M, Fernandez J, et al. Diagnosis of immediate allergic reactions to beta-lactam antibiotics. *Allergy Eur J Allergy Clin Immunol*. 2003;58(10):961-972. doi:10.1034/j.1398-9995.2003.00280.x

64. Moreno E, Laffond E, Muñoz-Bellido F, et al. Performance in real life of the European Network on Drug Allergy algorithm in immediate reactions to beta-lactam antibiotics. *Allergy*. 2016;71(12):1787-1790. doi:10.1111/all.13032

65. Wong JT, Ripple RE, MacLean JA, Marks DR, Bloch KJ. Vancomycin hypersensitivity: Synergism with narcotics and “desensitization” by a rapid continuous intravenous protocol. *J Allergy Clin Immunol*. 1994;94(2):189-194. doi:10.1016/0091-6749(94)90039-6
