# Supplementary material for: Standards for practical intravenous rapid drug desensitization & delabeling: A WAO committee statement
Source: World Allergy Organ J. 2022 May 31;15(6):100640. doi: 10.1016/j.waojou.2022.100640 (PMC9163606; doi:10.1016/j.waojou.2022.100640)
Supplement: Multimedia component 5 [file mmc5.pdf]

SUPPLEMENTARY TEXT 5

Fluoroquinolone Hypersensitivity Reactions

Johnson T. Wong, M.D.

Division of Rheumatology, Allergy and Immunology, Massachusetts General Hospital, Boston, Mass, USA.

This document is not intended to act as a prescriptive guideline for drug challenge or desensitization protocols. The objective of this supplementary text is not to review current evidence but to share personal experience. Local guidelines and guidelines of the corresponding national Allergy Societies should always be adhered to, and protocols should be adapted to the local population, local requirements, and local resources.

Fluoroquinolone is a powerful class of antibiotics that works by inhibiting DNA gyrase and DNA topoisomerase IV, thereby causing bacterial DNA strand breaks and inhibiting DNA synthesis[1–3]. Quinolones can be classified according to their generation: e.g., first (cinoxacin and nalidixic acid), second (ofloxacin, norfloxacin, ciprofloxacin, and enoxacin), third (levofloxacin), and fourth (gemifloxacin and moxifloxacin). They are well absorbed orally but also available in intravenous form. They have excellent bactericidal activity against aerobic gram-negative bacilli, various gram-positive cocci, many respiratory pathogens including both tuberculosis and non-tuberculosis mycobacteria, and special organisms such as anthrax and typhoid. Due to Fluoroquinolones' propensity for tendon injury/rupture, neurologic side effects, higher incidence of C. difficile, wide drug interactions, and the gradual emergence of bacterial resistance, their usage is advocated to be reserved for those situations where the conventional antibiotics are ineffective or unable to be used[3]. The latter may occur due to hypersensitivity reactions (HSRs) or side effects of the other antibiotics, or unavailability. The major fluoroquinolones currently used in the USA include ciprofloxacin, levofloxacin, and moxifloxacin[3]. Ofloxacin is used topically in ophthalmic infections. Delafloxacin and gemifloxacin are newer members.

Whereas adverse effects are common, true HSRs to fluoroquinolones were less common than HSRs to beta-lactam antibiotics and sulfonamide antibiotics in our experience. In a retrospective study of our patients, we were able to identify 29 patients with HSRs to fluoroquinolones with additional patients whose history were too poorly characterized to be included. Of these 29 patients, immediate hypersensitivity was the most common, with delayed onset maculopapular/morbilliform rash the

second largest group. Patients with features of Type III hypersensitivity occurred in 3 patients. We had no patients with Type II hematologic hypersensitivity to fluoroquinolones.

See the "General Concepts" section in the main manuscript for more information on the types of HSRs.

Immediate hypersensitivity reactions may include any combination of urticaria, angioedema, flushing, bronchospasm, hypotension, abdominal, and/or back pain[4,5]. The symptoms may start within minutes but some may be delayed for several hours. The severity may be mild, short-lasting and self resolve to severe anaphylaxis. The attempts to measure fluoroquinolone-binding IgE were described in a few studies using sepharose RIA and inhibition, reporting 30-55% elevated results in patients with immediate HSRs to fluoroquinolones[6,7]. These measurements need to be confirmed. In addition to the potential IgE-mediated mechanism, there were other reports of direct mast cell degranulation effect (DMCD) effect through the MRGPRX2 receptor[8,9]. In our series of 32 patients with fluoroquinolone HSR, immediate HSR was the most common group with 16 patients (50%). Though several patients had combined oral, pulmonary, and/or cutaneous reactions, we did not encounter anyone with severe anaphylaxis but such cases have been reported. Immediate hypersensitivity reactions for fluoroquinolones may subside over time. Limited measurements of IgE by one group found higher levels and greater frequency in patients whose HSR occurred <9 months vs those with longer elapsed period[7]. Based on our experience with other drugs, immediate HSR should be amenable to desensitization. In contrast to beta-lactam antibiotics, skin testing is not well established. The few reports on fluoroquinolone skin testing showed low sensitivity (about 50%)[10]. We have not been performing skin testing to fluoroquinolones. In our own experience, only 3 patients with immediate HSRs to fluoroquinolones were challenged with the same drug. One tolerated it without a problem, one had a similar HSR to the original, and one had a new HSR. Literature data suggest that cross-reactivity between second, third, and fourth generation quinolones is low[11]. In particular, a low degree of cross-reactivity between ciprofloxacin and levofloxacin has been found[12]. We had 5 patients who underwent a challenge with a different fluoroquinolone with 4 tolerated without a problem.

Type II (Antibody-mediated)[4,5]. Type II hypersensitivity reactions predominantly present as drug-induced hemolytic anemia (DIHA), drug-induced thrombocytopenia (DITP), and/or drug-induced neutropenia (DINP). Unlike beta-lactam antibiotics, there were only 1-2 case reports of DIHA[13,14], DITP[15], and DINP[16,17], respectively, associated with fluoroquinolones. Measurement of fluoroquinolone-binding antibodies was not done in the case reports. There were no patients in our 32 patients with fluoroquinolone HSR associated with Type II hypersensitivity.

Type III (antigen-antibody immune complex/complement-mediated)[4,5]. Serum sickness (SS) and serum sickness-like reactions (SSLR) are the prototypical type III hypersensitivity reactions where antigen-

antibody immune complexes of the right size deposit into various tissues, often fixing complements and leads to urticaria/non-urticarial rashes, arthralgia, adenopathy, fever, and other symptoms. Three out of our 32 fluoroquinolone HSR patients have features consistent with type III hypersensitivity with late-onset arthralgia, fever, and rashes. There were several case reports of SSLR associated with fluoroquinolones[18,19]. We have used an IV desensitization protocol with steroid/antihistamine pretreatment for chemotherapeutic agents, which generally allows the patients to continue the chemotherapy for a small number of courses with modulation of the symptoms. This is generally not worth doing in cases of antibiotics. At least for some patients, it appears that using a different member of the same or different beta-lactam family may be tolerated without inducing SS or SSLR[20]. This has not been investigated with fluoroquinolone hypersensitivities.

Type IV (T cells, non-T immune cells, cytokine-mediated) subtypes.[4,5] The most common Type 4 subtype is the late-onset exanthematous maculopapular/morbilliform rash and the least damaging. Five out of our 32 patients with fluoroquinolone HSR showed late-onset morbilliform rash. How often and how fast this subtype to fluoroquinolones wane over time and the degree of cross hypersensitivity among different fluoroquinolones are uncertain. Challenge data from beta-lactam antibiotics and sulfonamide antibiotics showed the majority of this subtype would wane over time. None of our series of 32 patients with fluoroquinolone HSRs had the more serious DRESS, SJS, or TEN. There were case reports in the literature of fluoroquinolone HSR associated with DRESS, SJS, or TEN[21–26]. Acute generalized exanthematous pustulosis (AGEP) is a rare member of the type IV subgroup characterized by neutrophilic pustules that are attributed to T cell inducing neutrophils and eosinophils. There were case reports of AGEP with fluoroquinolones[27–30]. In fixed drug eruption (FDE) there is a recurrence occurs of the rash in the same location and this is often associated with post-inflammatory hyperpigmentation. FDE is generally attributed to CD8+ T cells. Again there were only case reports of FDE and fluoroquinolone HSR[31,32].

Other mechanisms and/or combinations. Not all cases of fluoroquinolone HSRs can be nicely divided into one of the above categories. We have one patient who has both immediate and late-onset symptoms. Concurrent pathways have also been observed in HSRs to beta-lactam antibiotics and sulfonamide antibiotics. There were several patients whose history was not clear enough to decide the classification.

Fluoroquinolone Skin testing

Skin test on several reports appeared to have low sensitivity but high specificity, although other reports show low specificity. In any case, from our point of view, for patients with severe immediate/rapid onset hypersensitivity suggestive of anaphylaxis, it will be reasonable to perform skin testing with fluoroquinolone at non-irritating concentrations (prick levofloxacin 5mg/ml, Moxifloxacin 1.6mg/ml)[10,33]. The authors of the report cannot find a non-irritating concentration for ciprofloxacin. Intradermal testing was not recommended as the authors cannot identify a nonirritating concentration for the fluoroquinolones. A negative skin test would then allow a patient with a severe anaphylaxis history to be challenged. A positive skin test suggested that the patient proceed to be desensitized to a fluoroquinolone if there is no suitable alternative. We have not previously performed skin testing with the fluoroquinolone.

Interestingly, in a study by Krantz et al.[34], of 163 patients with hypersensitivity reactions to fluoroquinolones, mostly immediate, the authors selected a set of criteria for fluoroquinolone skin test positivity that allowed them to safely identify patients eligible for challenges, many of whom otherwise would have been excluded by current criteria for skin test positivity.

<u>Fluoroquinolones outpatient challenge protocol:</u>

- Challenge. A challenge protocol in the outpatient setting may be appropriate if one or more of the following is true: the original reaction(s) was minor, likely due to the original underlying infection rather than the medication, distant history, and/or had tolerated similar antibiotic(s) in the interim.
    - Administer fluoroquinolone 250mg at time 0 (start)
    - If tolerated, administered 2nd dose of the fluoroquinolone 250mg at 30min.
    - Continue to observe for at least 60min after the 2nd dose at the office and monitor for the next several days at home. The patient instructed to take pictures and call for any significant delayed reaction.
    - If the history stated that the initial reaction was delayed for many doses and days, consider continuing a 3-day challenge of 250-500mg once (levofloxacin or moxifloxacin) to twice (ciprofloxacin) a day for 3 days and monitor for the whole week.
    - If the patient needs the antibiotic to treat an infection, then extend the course to complete the treatment.

<u>Fluoroquinolones inpatient challenge/test dose protocol:</u>

- Challenge. A challenge protocol in the inpatient setting may be appropriate if one or more of the following is true: Patient is already inpatient, the original reaction(s) was minor, likely due to the original underlying infection rather than the medication, distant history, had tolerated similar antibiotic(s) in the interim, and/or the infection is so severe, that any delay in treatment is detrimental. Challenge may proceed with one of the following pathways:
    - Full dose under the usual protocol if time is of the essence. A slower infusion rate may be used for the first dose as long as the dose is finished prior to the next dose.
    - Test dose (usually 1/10 the full dose) followed by the remainder of the full dose 30-60min later if the test dose is tolerated. This may be pursued if there is adequate time for treatment.

TABLES

1.- Fluoroquinolone rapid intravenous desensitization protocol[1]

Time (hr:min)	Fluoroquinolone concentration (mg/ml)	Fluid infusion rate (ml/hr)	Fluoroquinolone infusion rate (mg/hr)	Cumulative dose (mg)
0:00	0.0001[2]	60.0	0.0060	0.0015
0:15	0.001	20.0	0.020	0.0065
0:30	0.001[3]	60.0	0.060	0.022
0:45	0.01	20.0	0.20	0.072
1:00	0.01	60.0	0.60	0.22
1:15	0.1	20.0	2.0	0.77
1:30	0.1	60.0	6.0	2.2
1:45	1.0	20.0	20	7.7
2:00	1.0	60.0	60	22
2:15	10	12.5	125	54
2:30	10	25.0	250	117
2:45[4]	10	50.0	500	242
3:00[5, 6, 7]	10	50.0	500	242

[1]Adapted from our original vancomycin protocol and beta-lactam antibiotic protocol[35]. This protocol has not been tested in fluoroquinolone HSR.

[2]Typical starting concentration for patients with severe systemic reactions to previous fluoroquinolone infusions.

[3]Typical starting concentration for patients with moderate systemic reactions to previous fluoroquinolone infusions. This will save 2 steps and 30min.

[4]Optional step for highly sensitive patients.

[5]Continue at this infusion rate for the remainder of the dosage.

[6]Minimize concurrent narcotic and other direct mast degranulators if possible

[7]May need to stay just below a threshold Fluoroquinolone infusion rate the first day and advance as tolerated (infrequently seen for cephalosporin).

2.- Fluoroquinolone rapid oral desensitization protocol[1]

Dose#	Time (hr:min)	Fluoroquinolone concentration (mg/ml)	Volume of suspension or # of tablet or capsule	Cumulative Dose (mg)	Reaction
1(optional)	0:00	Water	10ml	0	
2	0:15	0.1	1 ml	0.1	
3	0:30	0.1	3 ml	0.4	
4	0:45	0.1	10 ml	1.4	
5	1:00	1.0	3 ml	4.4	
6	1:15	1.0	10 ml	14.4	
7	1:30	1.0	30 ml	44.4	
8	1:45	10	6.0 ml	104	
9	2:00	10	12.5 ml	229	
10	2:15	250mg	1 cap or tab	479	
11	6-24hr depending on dosing frequency	250mg	1 cap or tab if dose is 250mg<br>2 if dose is 500 mg		
Thereafter give desired dose at desired frequency					

[1]Unpublished protocol. This protocol has not been tested in fluoroquinolone HSR.

- Open capsule or crush fluoroquinolone tablet 250mg and suspend in 25ml of water to make 10.0mg/ml solution (suspension).
- 10ml of fluoroquinolone 10mg/ml solution add to 90ml of water to made the 1.0mg/ml solution
- 10ml of fluoroquinolone 1.0mg/ml solution add to 90ml of water to made the 0.1mg/ml solution
- Obtain informed consent.
- Examine vital signs, oral mucosa, skin, and chest prior to start
- Shake up each solution well prior to taking out the appropriate amount with the appropriate size syringe for taking
- Monitor BP, skin, GI side effect, or other adverse symptoms.
- If adverse reactions develop, then repeat that dose or cut down to the previous dose. Lengthen protocol accordingly.
- Stay with the patient until 60 min after the first 250mg tablet or capsule is given.

REFERENCES

1. Drlica K, Zhao X. DNA gyrase, topoisomerase IV, and the 4-quinolones. *Microbiol Mol Biol Rev*. 1997;61(3):377-392. doi:10.1128/mmbr.61.3.377-392.1997

2. Correia S, Poeta P, Hébraud M, Capelo JL, Igrejas G. Mechanisms of quinolone action and resistance: where do we stand? *J Med Microbiol*. 2017;66(5):551-559. doi:10.1099/jmm.0.000475

3. Hooper D. Fluoroquinolones. UpToDate. https://www.uptodate.com/contents/fluoroquinolones. Published May 2021. Accessed June 24, 2021.

4. WEISS ME, ADKINSON NF. Immediate hypersensitivity reactions to penicillin and related antibiotics. *Clin Exp Allergy*. 1988;18(6):515-540. doi:10.1111/j.1365-2222.1988.tb02904.x

5. Pichler WWJ. Drug hypersensitivity: Classification and clinical features. UpToDate. https://www.uptodate.com/contents/drug-hypersensitivity-classification-and-clinical-features. Published 2019. Accessed March 11, 2021.

6. Aranda A, Mayorga C, Ariza A, et al. In vitro evaluation of IgE-mediated hypersensitivity reactions to quinolones. *Allergy Eur J Allergy Clin Immunol*. 2011;66(2):247-254. doi:10.1111/j.1398-9995.2010.02460.x

7. Manfredi M, Severino M, Testi S, et al. Detection of specific IgE to quinolones. *J Allergy Clin Immunol*. 2004;113(1):155-160. doi:10.1016/j.jaci.2003.09.035

8. Liu R, Hu S, Zhang Y, et al. Mast cell-mediated hypersensitivity to fluoroquinolone is MRGPRX2 dependent. *Int Immunopharmacol*. 2019;70:417-427. doi:10.1016/j.intimp.2019.02.001

9. Porebski G, Kwiecien K, Pawica M, Kwitniewski M. Mas-Related G Protein-Coupled Receptor-X2 (MRGPRX2) in Drug Hypersensitivity Reactions. *Front Immunol*. 2018;9:3027. doi:10.3389/fimmu.2018.03027

10. Seitz CS, Bröcker EB, Trautmann A. Diagnostic testing in suspected fluoroquinolone hypersensitivity. *Clin Exp Allergy*. 2009;39(11):1738-1745. doi:10.1111/j.1365-2222.2009.03338.x

11. Doña I, Moreno E, Pérez-Sánchez N, Andreu I, Hernández Fernandez de Rojas D, Torres MJ. Update on Quinolone Allergy. *Curr Allergy Asthma Rep*. 2017;17(8). doi:10.1007/s11882-017-0725-y

12. Lobera T, Audicana M, Alarcon E, Longo N, Navarro B, Munoz D. Allergy to quinolones: low cross-reactivity to levofloxacin. *J Investig Allergol Clin Immunol*. 2010;20(7):607-611. https://pubmed.ncbi.nlm.nih.gov/21314003/. Accessed June 24, 2021.

13. Oh YR, Carr-Lopez SM, Probasco JM, Crawley PG. Levofloxacin-induced autoimmune hemolytic anemia. *Ann Pharmacother*. 2003;37(7-8):1010-1013. doi:10.1345/aph.1C525

14. Sheikh-Taha M, Frenn P. Autoimmune Hemolytic Anemia Induced by Levofloxacin. *Case Rep Infect Dis*. 2014;2014:1-2. doi:10.1155/2014/201015

15. Shih AW, Lam AS, Warkentin TE. Levofloxacin-Induced Acute Immune-Mediated Thrombocytopenia of Rapid-Onset. *J Pharm Pract*. 2018;31(2):234-237. doi:10.1177/0897190017702306

16. Berk V, Demiraslan H, Berk E, et al. Moxifloxacin-associated neutropenia. *Scand J Infect Dis*. 2013;45(5):415-416. doi:10.3109/00365548.2012.738937

17. Chang CM, Lee NY, Lee HC, et al. Moxifloxacin-associated neutropenia in a cirrhotic elderly woman with lower extremity cellulitis. *Ann Pharmacother*. 2008;42(4):580-583. doi:10.1345/aph.1K596

18. Guharoy S. Serum sickness secondary to ciprofloxacin use. *Vet Hum Toxicol*. 1994;36(6):540-541. https://pubmed.ncbi.nlm.nih.gov/7900274/. Accessed June 24, 2021.

19. Slama TG. Serum sickness-like illness associated with ciprofloxacin. *Antimicrob Agents Chemother*. 1990;34(5):904-905. doi:10.1128/AAC.34.5.904

20. Blumenthal KG, Youngster I, Shenoy ES, Banerji A, Nelson SB. Tolerability of cefazolin after immune-mediated hypersensitivity reactions to nafcillin in the outpatient setting. *Antimicrob Agents Chemother*. 2014;58(6):3137-3143. doi:10.1128/AAC.02504-13

21. Charfi O, Lakhoua G, Sahnoun R, et al. DRESS Syndrome Following Levofloxacin Exposure with Positive Patch-test. *Therapie*. 2015;70(6):547-549. doi:10.2515/therapie/2015046

22. Christie MJ, Wong K, Ting RH, Tam PY, Sikaneta TG. Generalized seizure and toxic epidermal necrolysis following levofloxacin exposure. *Ann Pharmacother*. 2005;39(5):953-955. doi:10.1345/aph.1E587

23. Melde SL. Ofloxacin: A probable cause of toxic epidermal necrolysis. *Ann Pharmacother*. 2001;35(11):1388-1390. doi:10.1345/aph.1Z433

24. Moshfeghi M, Mandler HD. Ciprofloxacin-induced toxic epidermal necrolysis. *Ann Pharmacother*. 1993;27(12):1467-1469. doi:10.1177/106002809302701212

25. Uzun R, Yalcin AD, Celik B, Bulut T, Yalcin AN. Levofloxacin induced toxic epidermal necrolysis: Successful therapy with omalizumab (anti-IgE) and pulse prednisolone. *Am J Case Rep*. 2016;17:666-671. doi:10.12659/AJCR.899823

26. Vaghela JJ, Nimbark VN, Chavda BC, Mehta HH, Purohit BM. A Rare Case Report of Toxic Epidermal Necrolysis Due to Ofloxacin. *Curr Drug Saf*. 2018;13(2):137-140. doi:10.2174/1574886313666180302124012

27. Tajmir-Riahi A, Wörl P, Harrer T, Schliep S, Schuler G, Simon M. Life-Threatening Atypical Case of Acute Generalized Exanthematous Pustulosis. *Int Arch Allergy Immunol*. 2017;174(2):108-111. doi:10.1159/000480700

28. Kim H, Bang ES, Lim SK, Lee JM. DRESS syndrome and acute generalized exanthematous pustulosis induced by antituberculosis medications and moxifloxacin: Case report. *Int J Clin Pharmacol Ther*. 2016;54(10):808-815. doi:10.5414/CP202595

29. Foti C, Romita P, Zanframundo G, et al. Ciprofloxacin induced acute generallised exanthematous pustulosis. *Indian J Pharmacol*. 2017;49(1):119-120. doi:10.4103/0253-7613.201014

30. Häusermann P, Scherer K, Weber M, Bircher AJ. Ciprofloxacin-induced acute generalized exanthematous pustulosis mimicking bullous drug eruption confirmed by a positive patch test. *Dermatology*. 2005;211(3):277-280. doi:10.1159/000087024

31. Dhar S, Sharma V. Fixed drug eruption due to ciprofloxacin. *Br J Dermatol*. 1996;134(1):156-158. https://pubmed.ncbi.nlm.nih.gov/8745905/. Accessed June 24, 2021.

32. Iliyas M, Reddy MRS, Devi U. Ciprofloxacin-induced generalised non-bullous fixed drug eruption. *BMJ Case Rep*. 2018;2018. doi:10.1136/bcr-2018-224858

33. Solensky R. Hypersensitivity reactions to fluoroquinolones. UpToDate.

https://www.uptodate.com/contents/hypersensitivity-reactions-to-fluoroquinolones. Published March 23, 2021. Accessed June 24, 2021.

34. Krantz MS, Stone CA, Yu R, Adams SN, Phillips EJ. Criteria for Intradermal Skin Testing and Oral Challenge in Patients Labeled as Fluoroquinolone Allergic. *J Allergy Clin Immunol Pract*. September 2020. doi:10.1016/j.jaip.2020.09.017

35. Wong JT, Ripple RE, MacLean JA, Marks DR, Bloch KJ. Vancomycin hypersensitivity: Synergism with narcotics and "desensitization" by a rapid continuous intravenous protocol. *J Allergy Clin Immunol*. 1994;94(2):189-194. doi:10.1016/0091-6749(94)90039-6
