# Supplementary material for: Standards for practical intravenous rapid drug desensitization & delabeling: A WAO committee statement
Source: World Allergy Organ J. 2022 May 31;15(6):100640. doi: 10.1016/j.waojou.2022.100640 (PMC9163606; doi:10.1016/j.waojou.2022.100640)
Supplement: Multimedia component 6 [file mmc6.pdf]

SUPPLEMENTARY TEXT 6

Vancomycin Reactions Relevant to the Allergist (including Vancomycin infusion and Hypersensitivity reactions)

Johnson T. Wong, M.D.

Division of Rheumatology, Allergy and Immunology, Massachusetts General Hospital, Boston, Mass, USA.

This document is not intended to act as a prescriptive guideline for drug challenge or desensitization protocols. The objective of this supplementary text is not to review current evidence but to share personal experience. Local guidelines and guidelines of the corresponding national allergy societies should always be adhered to, and protocols should be adapted to the local population, local requirements, and local resources.

Vancomycin is a tricyclic glycopeptide antibiotic that is used intravenously (IV) to treat a variety of gram-positive cocci (GPC) bacterial infections including MRSA, enterococcus, GPC infections among patients with beta-lactam hypersensitivity, and GPC infections in patients with renal failure. Oral vancomycin is used to treat C. difficile. The spectrum of HSR includes:

I. Vancomycin infusion reactions (VIR) refers to flushing and pruritus that are commonly induced by the rate-dependent direct mast cell degranulation effect (DMCD) of vancomycin. In severe cases, hypotension, bronchospasm, and urticaria may also be present. At a rate of ≥ 1gm/hr, 10-80% of subjects developed RMS, whereas a much lower incidence occurred when the rate was reduced to 1gm over 2 hr[1]. Histamine is often elevated early on whereas tryptase was not elevated at 10min. Treatment of choice of mild to moderate RMS is to slow the infusion rate to ½ the previous infusion rate or ≤ 1gm/2hr, + antihistamine pretreatment. These reactions were formally referred to as "red man syndrome" and the nomenclature has recently been adjusted to more appropriate represent gender and racial inclusion and this has been endorsed by several professional societies[2,3].

II. <u>Anaphylactoid reactions/anaphylaxis/and/or severe refractory VIR (DMCD/Pseudoallergic and much less likely, IgE-mediated Type I):</u> may occur in a small subgroup of patients. Although IgE-mediated mechanism is possible, the majority of these cases appeared to be a severe form of DMCD. Vancomycin has been shown to be a small molecule ligand for MRGPRX2 which is currently the touted mechanism for the DMCD[4]. Vancomycin-specific IgE measurement has not been reported in the literature. Skin testing has been proposed both as a surrogate for sensitivity for the DMCD and possible IgE-mediated sensitivity (see Skin Test below)[5–7]. This highly sensitive group can be generally managed

by a rapid continuous IV desensitization protocol[8] and modified in various forms by our colleagues and us for desensitization of other drugs[9–11]. The advantage of this protocol is to provide continuous small increments that avoided periodic big increases in the rate of drug delivery. In several cases, one can only reach a threshold level on the first day and need to gradually increase over several days. When possible, stopping concurrent narcotics or other agents that also have DMCD property may often allow a difficult desensitization to be successful. These patients may benefit from stopping all other unnecessary drugs that are small molecule ligands for MRGPRX2 including fluoroquinolones, opioids, radiocontrast, and some practitiones recommend aggressive use of antihistamines.

III. Drug-induced thrombocytopenia (DITP), Drug-induced neutropenia (DINP) (type II): may occur in a small number of patients receiving vancomycin. At least for DITP, vancomycin-dependant platelet binding IgG and/or IgM were detectable in 34 patients by Von Drygalski, et al. DINP has also been reported (2-12% of extended infusion) with neutrophil binding antibodies demonstrated in some of the patients. These findings are consistent with Type II hypersensitivity (antibody-mediated). In general, stopping the Vancomycin infusion and avoidance of future usage is the preferred method. Assays for anti-drug platelet antibodies including vancomycin are available in some laboratories but have not undergone widespread validation.

IV. Drug Reaction with Eosinophilia and systemic symptoms/drug-induced hypersensitivity reaction: Delayed exanthem without other systemic features outside of that seen with VIR is uncommonly associated with vancomycin. Similarly drug fever without other systemic features associated with vancomycin has been described but is uncommon[12]. Vancomycin is now one of the most common causes of DRESS globally[13]. DRESS is a systemic disease that presents with facial edema, exanthem covering > 50% body surface area, lymphadenopathy, hematological changes such as eosinophilia and atypical lymphocytosis and internal organ involvement such as interstitial nephritis (more common with vancomycin) and hepatitis, however, any organ can be involved[13]. Circulating IFN-gamma vancomycin-specific T cells may be isolated in such patients[14]. A recent paper has shown a strong association between vancomycin DRESS and HLA-A*32:01. In this paper which primarily including European Americans, 83% of cases carried HLA-A*32:01 compared to 0% of controls. Using a time-to-event analysis this paper showed that approximately 20% of those carrying HLA-A*32:01 went onto develop vancomycin DRESS if exposed to > 2 weeks of vancomycin treatment. In a prospective component of this paper, IFN-gamma vancomycin ELISpot was a sensitive mechanism to detect those with vancomycin DRESS on multiple antibiotics. A combination of HLA-A*32:01 and functional testing may be useful diagnostically[15].

V. Linear IgA bullous diseases (LABD): Vancomycin is the most common reported cause of the drug-induced LABD and may present like TEN-like reactions[16]. Histologic findings, however, differs from TEN with IgA deposited in linear fashion in the basement membrane zone on the skin. Occasional IgM, IgG, and C3 deposit may be seen. These findings are supportive of Type II antibody-mediated hypersensitivity +/- a component of Type III immune complex-mediated hypersensitivity. The treatment is the cessation of vancomycin infusion.

VI. Stevens-Johnson Syndrome (SJD) and toxic epidermal necrolysis (TEN) (Type IV subtype):. SJS and TEN have also been reported (6/71 Vancomycin induced HSR) in one review and potentially life-threatening. Overall vancomycin associated SJS/TEN and reports in the literature may have been associated with mis-phenotyping (ie linear IgA bullous disease or severe DRESS/DIHS). Management is drug withdrawal and supportive treatment. These reactions serve as an absolute contraindication for re-administration or desensitization.

Teicoplanin and other glycopeptides and lipoglycopeptides as alternative treatments. Teicoplanin is a similar glycopeptide antibiotic that is only available outside the United States. Teicoplanin is not associated with DMCD or infusion reactions. One major retrospective study and one prospective study from the same institution over different time periods showed 12/117 (10%) and 14/24 (58%) patients, respectively, who had HSR to vancomycin also developed HSR to Teicoplanin[14,17,18]. There was a high recurrence rate for neutropenia, leukopenia, and thrombocytopenia (10/14). One study looked at cross-reactivity between vancomycin, teicoplanin, telavancin and dalbavancin using gamma-interferon ELISpot assay and showed that approximately 16% cross-reactivity between vancomycin, teicoplanin and telavancin in HLA-A*32:01 positive vancomycin DRESS patients[19].

Skin testing and implications

Skin testing has been proposed both as a surrogate for sensitivity for the DMCD and possible IgE-mediated sensitivity. The best study is reported by Polk, et al, where they performed titration of vancomycin skin test reactivity as a function of vancomycin concentration in a group of 12 healthy male volunteers[5]. At concentrations $\geq$10mcg/ml (0.02ml ID), all volunteers showed detectable wheal and flare. The area of the flare, but not the wheal size, increased with higher concentration up to 40-100mcg/ml (0.02ml ID) before plateauing. Since these are healthy volunteers without previous vancomycin exposure, the wheal and flare were due to DMCD and not IgE-mediated mechanism at doses $\geq$10mcg/ml (0.02ml ID). The size of the flare at 25mcg/ml (0.02ml intradermal testing)

correlated poorly with the area of flushing when the subjects were challenged with vancomycin infusion. Case reports of patients with “anaphylactoid/anaphylaxis/ pruritus” who developed wheal and flare at vancomycin concentration from 0.1mcg/ml to 5mcg/ml (0.02ml ID) on skin testing were interpreted by the authors to represent IgE-mediated sensitivity[6,7]. However, the cutaneous reactivity, even at the 0.1mcg/ml, may represent increased propensity for DMCD or increased sensitivity to the mast cell release products (induced by concurrent narcotic or due to intrinsic sensitivity of the patient)[8]. Hence vancomycin-specific IgE may need to be actually measured to be certain of IgE-mediated sensitivity, however, this is not commerically avaialble.

Considering that in the allergy workup, skin prick tests are usually performed before intradermal tests, the reader may find interesting a study by Otani et al.[20] of a case of perioperative anaphylaxis, in which skin prick testing with vancomycin at the concentration of 50 mg/mL proved to be nonirritating in 10 control subjects with no history of vancomycin reaction or exposure, while produced a clearly positive response in the patient, allowing to diagnose an IgE-mediated hypersensitivity to vancomycin. In addition, a recent study by Alvarez-Arango et al.[21] evaluated the effect of diluents (i.e., normal saline, human serum albumin-based sterile saline, lactated ringer's solution, and sterile water) on vancomycin skin test responses in 11 vancomycin-naïve subjects and, interestingly, human serum albumine in sterile saline proved to be the best tolerated diluent.

Vancomycin skin testing may be useful in the diagnosis of delayed hypersensitivity reactions such as DRESS. Skin testing should be deferred until 6 months following the acute presentation or when the patient has been off steroids for at least 1 month. Skin testing concentrations used for delayed testing are significantly higher than those used for immediate testing. In one paper concentrations of 0.05, 0.5, 5 and 50 mg/ml showed positive delayed repsonses only at the three highest concentrations[15].

TABLE 1: Vancomycin rapid intravenous desensitization protocol[1]

Time (hr:min)	Vancomycin concentration (mg/ml)	Fluid infusion rate (ml/hr)	Vancomycin infusion rate (mg/hr)	Cumulative dose (mg)
0:00	0.0001[3]	60.0	0.0060	0.0015
0:15	0.001	20.0	0.020	0.0065
0:30	0.001[4]	60.0	0.060	0.022
0:45	0.01	20.0	0.20	0.072
1:00	0.01	60.0	0.60	0.22
1:15	0.1	20.0	2.0	0.77
1:30	0.1	60.0	6.0	2.2
1:45	1.0	20.0	20	7.7
2:00	1.0	60.0	60	22
2:15	10	12.5	125	54
2:30[5, 6, 7]	10	25.0	250	117

[1]Adapted from our original protocol[8]

[2]H_1 antihistamine pretreatment.

[3]Typical starting concentration for patients with severe systemic reactions to previous vancomycin infusions.

[4]Typical starting concentration for patients with moderate systemic reactions to previous vancomycin infusions.

[5]Continue at this infusion rate for the remainder of the dosage.

[6]Minimize concurrent narcotic and other direct mast degranulators if possible

[7]May need to stay just below a threshold Vancomycin infusion rate the first day and advance as tolerated.

TABLE 2: For a patient who developed a hypersensitivity reaction to oral vancomycin, we modified the IV protocol for oral desensitization. For simplicity, we made up only two concentrations (1mg/ml and 0.1mg/ml). We administered 0.1mg over the second 15min, which represent 1/1250 of the final dose (125mg) by starting with 1ml of the 0.1mg/ml solution.

Vancomycin rapid oral desensitization protocol[1]

Dose#	Time (hr:min)	Vancomycin Concentration (mg/ml)	Volume or # of Pulvule	Cumulative Dose (mg)	Reaction
1	0:00	Water	10ml	0	
2	0:15	0.1	1 ml	0.1	
3	0:30	0.1	3 ml	0.4	
4	0:45	0.1	10 ml	1.4	
5	1:00	1.0	3 ml	4.4	
6	1:15	1.0	10 ml	14.4	
7	1:30	1.0	30 ml	44.4	
8	1:45	1.0	60 ml	104	
9	2:00	125mg pulvule	1	229	
10	6-8:00	125mg pulvule	2	479	
11	10-12:00	250mg pulvule	2	979	
Thereafter give desired dose					

[1]Unpublished protocol

- Open or crush Vancomycin HCL125 mg pulvule and suspend in 125 ml of water to make 1.0 mg/ml solution (suspension)
- 10ml of Vancomycin HCL 1.0 mg/ml solution add to 90ml of water to made the 0.1 mg/ml solution
- Obtain informed consent.
- Examine vital signs, oral mucosa, skin, and chest prior to start
- Shake up each solution well prior to taking out appropriate amount with appropriate size syringe for taking
- Monitor temperature, skin, GI side effect, or other adverse symptoms.
- If GI side effect significant, then repeat that dose or cut down to previous dose. Lengthen protocol accordingly.
- Stay with patient until 60 min after the first 125 mg pulvule given.

REFERENCES FOR SUPPLEMENTARY TEXT 4:

1. Polk RE, Healy DP, Schwartz LB, et al. Vancomycin and the red-man syndrome: Pharmacodynamics of histamine release. *J Infect Dis*. 1988;157(3):502-507. doi:10.1093/infdis/157.3.502

2. Alvarez-Arango S, Ogunwole SM, Sequist TD, Burk CM, Blumenthal KG. Vancomycin Infusion Reaction — Moving beyond "Red Man Syndrome." *https://doi.org/101056/NEJMp2031891*. 2021;384(14):1283-1286. doi:10.1056/NEJMP2031891

3. Austin JP, Empey A, Foster BA. Position on Terminology for Vancomycin Flushing Reactions. 2021. doi:10.1056/NEJMp2031891

4. E A, VB R, EA L. Brief communication: MRGPRX2, atopic dermatitis and red man syndrome. *Itch (Philadelphia, Pa)*. 2017;2(1):e5-e5. doi:10.1097/ITX.0000000000000005

5. Polk RE, Israel D, Wang J, Venitz J, Miller J, Stotka J. Vancomycin skin tests and prediction of "red man syndrome" in healthy volunteers. *Antimicrob Agents Chemother*. 1993;37(10):2139-2143. doi:10.1128/AAC.37.10.2139

6. Anne S, Middleton EJ, Reisman R. Vancomycin anaphylaxis and successful desensitization. *Ann Allergy*. 1994;73(5). https://pubmed.ncbi.nlm.nih.gov/7978531/. Accessed June 24, 2021.

7. Hwang MJ, Do JY, Choi EW, et al. Immunoglobulin E-mediated hypersensitivity reaction after intraperitoneal administration of vancomycin. *Kidney Res Clin Pract*. 2015;34(1):57-59. doi:10.1016/j.krcp.2014.09.005

8. Wong JT, Ripple RE, MacLean JA, Marks DR, Bloch KJ. Vancomycin hypersensitivity: Synergism with narcotics and "desensitization" by a rapid continuous intravenous protocol. *J Allergy Clin Immunol*. 1994;94(2):189-194. doi:10.1016/0091-6749(94)90039-6

9. Wong JT, Nagy CS, Krinzman SJ, MacLean JA, Bloch KJ. Rapid oral challenge-desensitization for patients with aspirin-related urticaria-angioedema. *J Allergy Clin Immunol*. 2000;105(5):997-1001. doi:10.1067/mai.2000.104571

10. Wong JT, Ling M, Patil S, Banerji A, Long A. Oxaliplatin hypersensitivity: Evaluation, implications of skin testing, and desensitization. *J Allergy Clin Immunol Pract*. 2014;2(1):40-45. doi:10.1016/j.jaip.2013.08.011

11. Hsu Blatman KS, Castells MC. Desensitizations for chemotherapy and monoclonal antibodies: Indications and outcomes. *Curr Allergy Asthma Rep*. 2014;14(8):1-8. doi:10.1007/s11882-014-0453-5

12. Clayman MD, Capaldo RA. Vancomycin Allergy Presenting as Fever of Unknown Origin. *Arch Intern Med*. 1989;149(6):1425-1426. doi:10.1001/ARCHINTE.1989.00390060137031

13. Blumenthal KG, Patil SU, Long AA. The importance of vancomycin in drug rash with eosinophilia and systemic symptoms (dress) syndrome. *Allergy Asthma Proc*. 2012;33(2):165-171. doi:10.2500/aap.2012.33.3498

14. Elera JD, Boteanu C, Blanco MAJ, et al. P164 Vancomycin-specific T cell responses and teicoplanin cross-reactivity / 7th Drug hypersensitivity meeting: part two. *Clin Transl Allergy*. 2016;6(S3). doi:10.1186/s13601-016-0122-y

15. Konvinse KC, Trubiano JA, Pavlos R, et al. HLA-A*32:01 is strongly associated with vancomycin-induced drug reaction with eosinophilia and systemic symptoms. *J Allergy Clin Immunol*. 2019;144(1):183. doi:10.1016/J.JACI.2019.01.045

16. Waldman MA, Black DR, Callen JP. Vancomycin-induced linear IgA bullous disease presenting as toxic epidermal necrolysis. *Clin Exp Dermatol*. 2004;29(6):633-636. doi:10.1111/j.1365-2230.2004.01649.x

17. Hsiao SH, Chou CH, Lin WL, et al. High risk of cross-reactivity between vancomycin and sequential teicoplanin therapy. *J Clin Pharm Ther*. 2012;37(3):296-300. doi:10.1111/j.1365-2710.2011.01291.x

18. Hung YP, Lee NY, Chang CM, et al. Tolerability of teicoplanin in 117 hospitalized adults with previous vancomycin-induced fever, rash, or neutropenia: A retrospective chart review. *Clin Ther*. 2009;31(9):1977-1986. doi:10.1016/j.clinthera.2009.09.010

19. Nakkam N, Gibson A, Mouhtouris E, et al. Cross-reactivity between vancomycin, teicoplanin, and telavancin in patients with HLA-A∗32:01–positive vancomycin-induced DRESS sharing an HLA class II haplotype. *J Allergy Clin Immunol*. 2021;147(1):403-405. doi:10.1016/J.JACI.2020.04.056

20. Otani IM, Kuhlen JL, Blumenthal KG, Guyer A, Banerji A. A role for vancomycin epicutaneous skin testing in the evaluation of perioperative anaphylaxis. *J Allergy Clin Immunol Pract*. 2015;3(6):984-985. doi:10.1016/j.jaip.2015.06.017

21. Alvarez-Arango S, Oliver E, Tang O, et al. Vancomycin immediate skin responses in vancomycin-naïve subjects. *Clin Exp Allergy*. 2021. doi:10.1111/cea.13850
