# Supplementary material for: Standards for practical intravenous rapid drug desensitization & delabeling: A WAO committee statement
Source: World Allergy Organ J. 2022 May 31;15(6):100640. doi: 10.1016/j.waojou.2022.100640 (PMC9163606; doi:10.1016/j.waojou.2022.100640)
Supplement: Multimedia component 7 [file mmc7.pdf]

SUPPLEMENTARY TEXT 7

Sulfonamide Antibiotic Hypersensitivity Reactions

Johnson T. Wong, M.D.
Division of Rheumatology, Allergy and Immunology, Massachusetts General Hospital, Boston, Mass, USA.

This document is not intended to act as a prescriptive guideline for drug challenge or desensitization protocols. The objective of this supplementary text is not to review current evidence but to share personal experience. Local guidelines and guidelines of the corresponding national Allergy Societies should always be adhered to, and protocols should be adapted to the local population, local requirements, and local resources.

Sulfonamide antibiotic hypersensitivity is the 2nd most commonly reported antibiotic hypersensitivity, behind only the reported rate for beta-lactam antibiotics[1]. The combination of trimethoprim (TMP)-sulfamethoxazole (SMX) is the most common sulfonamide antibiotic used. The combination works by sequentially inhibiting microbial synthesis of tetrahydrofolic acid (THF)[2–4]. THF is necessary for thymidine production and interference therefore inhibits susceptible microbe DNA synthesis. TMP-SMX is effective in treating a variety of aerobic gram-positive and gram-negative bacteria, pneumocystis jirovecii, and certain protozoan[2,3,5–7]. It has proven useful in treating certain methicillin-resistant Staph. aureus (MRSA)[8–10]. Due to its effectiveness against pneumocystis and other respiratory infections in immunocompromised hosts, it is a useful prophylactic agent for immunologists, pulmonologists, and infectious disease specialists. It is concentrated in the urine and therefore use extensively for UTI. Both adverse side effects and true hypersensitivity reactions are common. Though pharmacologically unrelated, hypersensitivity reactions to sulfonamide antibiotics are higher in patients with penicillin family hypersensitivities and vice versa (each about 15%)[11,12]. True hypersensitivities have been described for sulfonamide antibiotic for all 4 major classes and subclasses of hypersensitivity reactions[13].

For further information on types of hypersensitivity reactions, please see the “General Concepts” section in the main manuscript.

<u>Immediate/rapid onset hypersensitivity reactions (HSR)</u> -may include any combination of urticaria, angioedema, flushing, bronchospasm, hypotension, abdominal, and/or back pain. The symptoms may start within minutes but some may be delayed for several hrs. The severity may be mild, shortlasting and self resolve to severe anaphylaxis needing emergency room treatment. Immediate/rapid onset

HSR has generally been attributed to Type I HSR (IgE-mediated). However, measurement of IgE via RAST has been reported only on 3 patients by Gruchalla and Sullivan, using a non-commercially available sulfamethoxazole poly-L-tyrosine conjugate[14]. Skin test with the same reagent reportedly was positive only on 29% of patients with a positive immediate reactive history. Skin test using a 1:100 of the commercially available IV formulation of TMP-SMX (1/100 16/80mg/ml) was found to be non-irritating but there was no characterization of the sensitivity or specificity[15]. Patch test might be helpful for fixed drug eruptions (FDE) but not for other forms of sulfonamide antibiotic HSR and with variable results[16,17]. These tests were not judged reliable enough to be used for most practices. Immediate/rapid onset hypersensitivity occurs much less frequently than late onset maculopapular/morbilliform rash (see Type IV) for sulfonamide antibiotic[1,13]. Full dose challenge study by Krantz, et al suggested that patients with immediate/rapid onset hypersensitivity to TMP-SMX may wane over time as 20 of 23 patients with a history of immediate reaction tolerated a single dose challenge (followed for only 24hr)[18]. Challenge remained the most accurate way to determine the current hypersensitivity status and the data from several series suggested challenge may be tried first if the immediate Type I hypersensitivity was non-anaphylactic. If the challenge is positive or if the patient has a history of anaphylaxis to sulfonamide antibiotic then desensitization is the recommended approach for patients with Type I hypersensitivity to sulfonamide antibiotics if an alternative treatment is not available, an IV protocol may be used if patient needs the IV formulation. We have devised several oral protocols that varied over how fast the desensitization takes place (4 hours to 4 days). Of course, other groups may have different approaches[19], but we will focus on the MGH approach.

<u>Type II (Antibody-mediated)</u>. Type II hypersensitivity reactions predominantly present as drug-induced hemolytic anemia (DIHA), drug-induced thrombocytopenia (DITP), and/or drug-induced neutropenia (DINP). In several series with DIHA, DITP, and DINP, sulfonamide antibiotics were shown to be important. In addition, sulfonamide antibiotics also can cause hemolytic anemia in patients with G6PD deficiency. How long Type II hypersensitivities to sulfonamide antibiotics persist is unknown. For patients who developed Type II reactions, it is generally recommended to avoid using the same or similar sulfonamide antibiotic.

<u>Type III (antigen-antibody immune complex/complement-mediated)</u>. Serum sickness (SS) and serum sickness like reactions (SSLR) are the prototypical Type III hypersensitivity reaction where antigen-antibody immune complexes of the right size deposit into various tissues, often fixing complements and leads to urticaria/non-urticarial rashes, arthralgia, adenopathy, fever, and other symptoms.

Sulfonamide antibiotics have been rarely reported to cause SS or SSLR. For chemotherapeutic agents that have caused SS or SSLR, we have used IV desensitization protocol with steroid/antihistamine pretreatment which generally allows the patients to continue the chemotherapy for a small number of courses with modulation of the symptoms. However, this is generally not worth doing in cases of antibiotics.

Type IV (T cells, non-T cells, cytokine-mediated) subtypes. Sulfonamide antibiotics have been associated with all Type IV subtypes. The most common Type IV subtype is the late onset exanthematous maculopapular/morbilliform rash and this subtype is the least damaging. In the literature, it is also the most common form of hypersensitivity for sulfonamide antibiotics. It may be accompanied by superficial desquamation, fever, +/- eosinophilia, and +/- mild end organ damage. Typically the rash starts centrally and spread peripherally, at times clearing centrally even as the peripheral involvement increases (author's clinical observation). The reactions generally resolve over several days to weeks without causing permanent damage. Similar to beta-lactam antibiotics, this maculopapular/morbilliform sensitivity to sulfonamide antibiotics also appears to wane over time. Challenge data from several series suggested that around 90% of patients with delayed or unknown history tolerated a full dose challenge with TMP-SMX (a median 20 years in tolerated patients vs a median of only 3 years in failed patients)[18]. As with Type I hypersensitivity, the current data suggest challenge under the right circumstance is a reasonable first step if the delayed hypersensitivity did not involve damaging end organs or severe scarring. If the challenge is positive, then desensitization is the recommended approach for patients with this type of reactivity if an alternative treatment is not available. Less common but potentially serious is drug reaction with eosinophilia and systemic symptoms (DRESS)/ drug-induced hypersensitivity syndrome (DiHS). This shares many of the characteristics but more severe and potentially damaging. The eosinophilia are generally moderately to markedly elevated and the end organ damage(s) moderate to severe. DRESS reactions have progressed when the causative drug was not initially recognized and readministered within a few months. Stevens Johnson Syndrome (SJS) with mucosal involvement and exfoliation of deeper layers range from moderate to severe. Toxic epidermal necrolysis (TEN) where extensive deep layers of the skin shed are relatively rare, disfiguring, and potentially life threatening. How long the hypersensitivity of DRESS, SJS, and TEN persist in patients with these severe late phase reactions are uncertain as the general approach has been avoidance of the drugs[13]. Acute generalized exanthematous pustulosis (AGEP) is a rare member of Type IV subgroup characterized by neutrophilic pustules that was attributed to T cell inducing neutrophils and eosinophils. Fixed drug eruption (FDE) where recurrence of the rash occurs in the same location and is often associated with post-inflammatory

hyperpigmentation. It is generally attributed to CD8+ T cells. Sulfonamide antibiotic is a common cause of FDE[1,13].

Special considerations:

Hypersensitivity reaction (HSR) to penicillin family antibiotics among patients with HSR to sulfonamide antibiotics

Though pharmacologically unrelated, HSR to penicillin family antibiotics are substantially higher among patients with HSR to sulfonamide antibiotics and vice versa (each around 15%)[11,20]. The reason for this higher rate of HSR than among the general population is unclear but attributed to how patients may metabolize and the propensity to trigger HSR to drugs[11,13]. It should be noted however, that this increased HSR does not apply to non-beta-lactam antibiotics families of antibiotics except for fluoroquinolones. Hence patients with HSR to sulfonamide antibiotics do not appear to have a general increase propensity for HSR for all drugs.

Cross-hypersensitivity among sulfonamide antibiotics and sulfonamide non-antibiotics

Strom, et al, published a retrospective study in the UK showing that among patients who had a history of sulfonamide antibiotic hypersensitivity, 9.9% subsequently had an HSR to sulfonamide non-antibiotics[11]. Since this is lower than the 15% HSR to penicillin family of antibiotic, Strom et al and subsequently many other investigators interpreted this to mean there is no cross-hypersensitivity among sulfonamide antibiotics and sulfonamide non-antibiotics. However, Strom, et al did show that the 9.9% is several times higher than the 1.6% HSR to sulfonamide non-antibiotics among patients without a history of HSR to sulfonamide antibiotic. Based on this, we conclude that it is incorrect to state that there is no increased HSR to sulfonamide non-antibiotics among patients with HSR to sulfonamide antibiotic. The rate of HSR is approximately 10% and should be presented to patients and referring physicians as such. Whether this 10% HSR rate is due to immunological crossreactivity (deemed unlikely as the non-antibiotic sulfonamide lacks the N4 aromatic amine[13]) or due to other mechanism is unclear at present. The 10% rate is an acceptably low risk to take sulfonamide non-antibiotics but providers should discuss this with the patients. Patients should be informed that they should hold the drug and discuss their situation should they develop a reaction.

Sulfonamide antibiotic HSR among HIV-infected patients

Several series reported much higher incidence of adverse reactions, which include HSR, to sulfonamide antibiotics among HIV-infected patients, ranging from 30-70%. Hypothesis for this increased incidence include greater usage of sulfonamide antibiotics for pneumocystitis

treatment/prophylaxis, altered T cell immunity, and possibly glutathione deficiency[13,21–23]. When not associated with DRESS, SJS, or TEN, the incidence of HSR to sulfonamide antibiotics among HIV-infected patients also did appeared to diminish over time. It might differ between early (15 out of 26, 58% recurrence) and recent (2 out of 9, 22% recurrence) trials though the subject numbers were small[18]. As effectiveness of HIV treatment has improved over time, the need for PCP prophylaxis/treatment has also gone down. Given the recent trial, it is reasonable to start with an oral challenge in those HIV-infected subjects with a history of HSR to sulfonamide antibiotics provided there was no previous history of DRESS, SJS, TEN, or anaphylaxis. If HSR occur during challenge and there is no suitable alternative, then the patients may be a candidate for desensitization.

Sulfonamide antibiotics challenge protocol (modified from Krantz, et al[18]):

- Administer sulfonamide antibiotic TMP-SMX SS 80-400 mg tablet or suspension at time 0 (start)
- If tolerated, administered 2nd dose of antibiotic TMP-SMX SS 80-400 mg tablet or suspension at 60 min.
- Continue to observe for at least 60 min after the 2nd dose at the office and monitor for next several days at home. Patient instructed to take pictures and call for any significant delayed reaction.
- If the history stated that the initial reaction was delayed for many doses and days, consider continuing a 3 day challenge of TMP-SMX DS once to twice a day for 3 more days and monitor for the whole week.
- If patient needs the antibiotic to treat an infection, then extend the course to complete the treatment.

Sulfonamide antibiotic rapid intravenous desensitization protocol[1]

Time (hr:min)	Sulfonamide antibiotic concentration TMP/SMX(mg/ml)	Fluid infusion rate (ml/hr)	Volume infuse for step (ml)	Cumulative dose TMP/SMX (mg)
0:00	0.016/0.080	20	5.0	0.08/0.40
0:15	0.016/0.080	60	15.0	0.32/1.6
0:30	0.16/0.80	20	5.0	1.1/5.6
0:45	0.16/0.80	60	15.0	3.5/17.5
1:00	1.6/8.0	12.5	3.12	8.8/44
1:15	1.6/8.0	25	6.25	19/94
1:30	1.6/8.0	50	12.5	39/194
1:45-2:26[2]	1.6/8.0	100	68.2	148/741
12:00-13:30	16/80(full strength from manufacturer	6.7	10	

[1]Adapted from our original vancomycin protocol[24]. IV protocol may be used if patient needs IV formulation.

[2]Continue at this infusion rate for the remainder of the dosage.

- To make the TMP-SMX 1.6/8.0 mg/ml concentration, take the full 10ml of 16/80 stock solution from the manufacture and dilute into 90 ml of 5% dextrose in water.
- To make the TMP-SMX 0.16/0.80 mg/ml concentration, take the 10 ml of 1.6/8.0 mg/ml solution and dilute into 90ml of 5% dextrose in water.
- To make the TMP-SMX 0.016/0.080 mg/ml concentration, take the 10 ml 0.16/0.80 mg/ml solution and dilute into 90 ml of 5% dextrose in water.
- Adjust dose for renal failure.

Sulfonamide antibiotic (TMP-SMX) Rapid Oral Desensitization Protocol (over 3 hr for dose escalating portion)

Day	Time hr:min	TMP-SMX Concentration mg/ml (dilution)	Volume	Reaction
1[2]	0:00	0.08/0.4 (1/100)	1 ml	
	0:20	0.08/0.4 (1/100)	3 ml	
	0:40	0.08/0.4 (1/100)	10 ml	
	1:00	0.8/4.0 (1/10)	3 ml	
	1:20	0.8/4.0 (1/10)	10 ml	
	1:40	0.8/4.0 (1/10)	30 ml	
	2:00	8.0/40 full strength	5 ml	
	2:20	8.0/40 full strength	10 ml	
	8:00 or12:00	8.0/40 full strength	20ml	
2[3]	AM	DS tablet	1 tab	
	PM	DS tablet	1 tab	

[1]Adapted from our original vancomycin protocol[24].
[2]Day 1 may be performed in ICU setting if the previous history was severe. May be performed in monitored non-ICU setting if the previous history was not severe.
[3]Day 2 may be performed in non-ICU setting

Procedure:

- Dilute 1 ml of Sulfonamide antibiotic (TMP-SMX) suspension from pharmacy to 99 ml (water) to make 1/100 suspension
- 10ml of Sulfonamide antibiotic (TMP-SMX) suspension add to 90ml of water to made the 1/10 suspension
- Obtain informed consent.
- Examine vital signs, oral mucosa, skin, and chest prior to start
- Shake up each suspension well prior to taking out appropriate amount with appropriate size syringe for taking
- Monitor temperature, skin, GI side effect, or other adverse symptoms.
- If GI side effect occurs, then repeat that dose or cut down to previous dose. Lengthen protocol accordingly.
- Stay with patient until 60 min after the first SS 80-400 mg of tablet or suspension given.
- Adjust dose for renal failure.

Sulfonamide antibiotic (TMP-SMX) Intermediate Oral Desensitization Protocol (over 4.5 days for dose escalating portion)

Day	Time	TMP-SMX Concentration (mg/cc)	Volume	Reaction
1	AM	0.080/0.40 mg/ml (1/100)	1 cc	
	PM	0.080/0.40 mg/ml (1/100)	3 cc	
2	AM	0.080/0.40 mg/ml (1/100)	10 cc	
	PM	0.80/4.0 mg/ml (1/10)	3 cc	
3	AM	0.80/4.0 mg/ml (1/10)	10 cc	
	PM	0.80/4.0 mg/ml (1/10)	30 cc	
4	AM	8.0/40 mg/ml (full strength)	5 cc	
	PM	8.0/40 mg/ml (full strength)	10 cc	
5	AM	DS tablet	1	
	PM	DS tablet	1	

[1]Adapted from our original vancomycin protocol[24].
[2]Day 1 may be performed in ICU setting if the previous history was severe. May be performed in monitored non-ICU setting if the previous history was not severe.
[3]Day 2-5 may be performed in non-ICU setting

Procedure:

- Dilute 1 ml of Sulfonamide antibiotic (TMP-SMX) suspension from pharmacy to 100ml (water) to make 1/100 suspension
- 10 ml of Sulfonamide antibiotic (TMP-SMX) suspension add to 90ml of water to made the 1/10 suspension
- Obtain informed consent.
- Examine vital signs, oral mucosa, skin, and chest prior to start
- Shake up each suspension well prior to taking out appropriate amount with appropriate size syringe for taking
- Monitor temperature, skin, GI side effect, or other adverse symptoms.
- If GI side effect occurs, then repeat that dose or cut down to previous dose. Lengthen protocol accordingly.
- Stay with patient until 60 min after the first SS 80-400 mg of tablet or suspension given.
- Adjust dose for renal failure.
- May repeat any of the dose escalating steps to lengthen the protocol as desired.
- May lengthen the protocol to a slow 9 days by repeating the morning dose for each day and move the evening dose to the next day. This will effectively converts to a slow protocol.

REFERENCES

1. Bigby M. Drug-induced cutaneous reactions. A report from the Boston Collaborative Drug Surveillance Program on 15,438 consecutive inpatients, 1975 to 1982. *JAMA J Am Med Assoc*. 1986;256(24):3358-3363. doi:10.1001/jama.256.24.3358

2. Kalkut G. Sulfonamides and trimethoprim. *Cancer Invest*. 1998;16(8):612-615. doi:10.3109/07357909809032892

3. Gleckman R, Blagg N, Joubert DW. Trimethoprim: Mechanisms of Action, Antimicrobial Activity, Bacterial Resistance, Pharmacokinetics, Adverse Reactions, and Therapeutic Indications. *Pharmacother J Hum Pharmacol Drug Ther*. 1981;1(1):14-19. doi:10.1002/j.1875-9114.1981.tb03548.x

4. Hong YL, Hossler PA, Calhoun DH, Meshnick SR. Inhibition of recombinant Pneumocystis carinii dihydropteroate synthetase by sulfa drugs. *Antimicrob Agents Chemother*. 1995;39(8):1756-1763. doi:10.1128/AAC.39.8.1756

5. Smilack JD. Trimethoprim-sulfamethoxazole. In: *Mayo Clinic Proceedings*. Vol 74. Elsevier Ltd; 1999:730-734. doi:10.4065/74.7.730

6. Gleckman R, Gantz NM, Joubert DW. Intravenous Sulfamethoxazole-Trimethoprim: Pharmacokinetics, Therapeutic Indications, and Adverse Reactions. *Pharmacother J Hum Pharmacol Drug Ther*. 1981;1(3):206-211. doi:10.1002/j.1875-9114.1981.tb02542.x

7. May DB. Trimethoprim-sulfamethoxazole: An overview. UpToDate. https://www.uptodate.com/contents/trimethoprim-sulfamethoxazole-an-overview. Published January 9, 2020. Accessed June 25, 2021.

8. Khan A, Wilson B, Gould IM. Current and future treatment options for community-associated MRSA infection. *Expert Opin Pharmacother*. 2018;19(5):457-470. doi:10.1080/14656566.2018.1442826

9. David MZ, Daum RS. Treatment of Staphylococcus aureus infections. In: *Current Topics in Microbiology and Immunology*. Vol 409. Springer Verlag; 2017:325-383. doi:10.1007/82_2017_42

10. Holland TL, Arnold C, Fowler VG. Clinical management of staphylococcus aureus bacteremia: A review. *JAMA - J Am Med Assoc*. 2014;312(13):1330-1341. doi:10.1001/jama.2014.9743

11. Strom BL, Schinnar R, Apter AJ, et al. Absence of Cross-Reactivity between Sulfonamide Antibiotics and Sulfonamide Nonantibiotics. *N Engl J Med*. 2003;349(17):1628-1635. doi:10.1056/NEJMoa022963

12. Montanaro A. Sulfonamide allergy in HIV-uninfected patients. UpToDate. https://www.uptodate.com/contents/sulfonamide-allergy-in-hiv-uninfected-patients. Published April 3, 2020. Accessed June 25, 2021.

13. Khan DA, Knowles SR, Shear NH. Sulfonamide Hypersensitivity: Fact and Fiction. *J Allergy Clin Immunol Pract*. 2019;7(7):2116-2123. doi:10.1016/j.jaip.2019.05.034

14. Gruchalla RS, Sullivan TJ. Detection of human IgE to sulfamethoxazole by skin testing with sulfamethoxazoyl-poly-l-tyrosine. *J Allergy Clin Immunol*. 1991;88(5):784-792. doi:10.1016/0091-6749(91)90186-R

15. Empedrad R, Darter AL, Earl HS, Gruchalla RS. Nonirritating intradermal skin test concentrations for commonly prescribed antibiotics [4]. *J Allergy Clin Immunol*. 2003;112(3):629-630. doi:10.1016/S0091-6749(03)01783-4

16. Özkaya-Bayazit E, Bayazit H, Özarmağan G. Topical provocation in 27 cases of cotrimoxazole-induced fixed drug eruption. *Contact Dermatitis*. 1999;41(4):185-189. doi:10.1111/j.1600-0536.1999.tb06127.x

17. Tornero P, De Barrio M, Baeza ML, Herrero T. Cross-reactivity among p-amino group compounds in sulfonamide fixed drug eruption: Diagnostic value of patch testing. *Contact Dermatitis*. 2004;51(2):57-62. doi:10.1111/j.0105-1873.2004.00274.x

18. Krantz MS, Stone CA, Abreo A, Phillips EJ. Oral challenge with trimethoprim-sulfamethoxazole in patients with "sulfa" antibiotic allergy. *J Allergy Clin Immunol Pract*. 2020;8(2):757-760.e4. doi:10.1016/j.jaip.2019.07.003

19. Pyle RC, Butterfield JH, Volcheck GW, et al. Successful outpatient graded administration of trimethoprim-sulfamethoxazole in patients without HIV and with a history of sulfonamide adverse drug reaction. *J Allergy Clin Immunol Pract*. 2014;2(1):52-58. doi:10.1016/j.jaip.2013.11.002

20. Apter AJ, Kinman JL, Bilker WB, et al. Is there cross-reactivity between penicillins and cephalosporins? *Am J Med*. 2006;119(4). doi:10.1016/j.amjmed.2005.10.052

21. Jaffe HS, Ammann AJ, Abrams DI, Lewis BJ, Golden JA. COMPLICATIONS OF CO-TRIMOXAZOLE IN TREATMENT OF AIDS-ASSOCIATED PNEUMOCYSTIS CARINII PNEUMONIA IN HOMOSEXUAL MEN. *Lancet*. 1983;322(8359):1109-1111. doi:10.1016/S0140-6736(83)90627-X

22. Gordin FM, Simon GL, Wofsy CB, Mills J. Adverse reactions to trimethoprim-sulfamethoxazole in patients with the acquired immunodeficiency syndrome. *Ann Intern Med*. 1984;100(4):495-499. doi:10.7326/0003-4819-100-4-495

23. Eliaszewicz M, Flahault A, Roujeau JC, et al. Prospective evaluation of risk factors of cutaneous drug reactions to sulfonamides in patients with AIDS. *J Am Acad Dermatol*. 2002;47(1):40-46. doi:10.1067/mjd.2002.120468

24. Wong JT, Ripple RE, MacLean JA, Marks DR, Bloch KJ. Vancomycin hypersensitivity: Synergism with narcotics and "desensitization" by a rapid continuous intravenous protocol. *J Allergy Clin Immunol*. 1994;94(2):189-194. doi:10.1016/0091-6749(94)90039-6
