# Supplementary material for: Standards for practical intravenous rapid drug desensitization & delabeling: A WAO committee statement
Source: World Allergy Organ J. 2022 May 31;15(6):100640. doi: 10.1016/j.waojou.2022.100640 (PMC9163606; doi:10.1016/j.waojou.2022.100640)
Supplement: Multimedia component 8 [file mmc8.pdf]

SUPPLEMENTARY TEXT 8

Macrolide Hypersensitivity Reactions

Johnson T. Wong, M.D.

Division of Rheumatology, Allergy and Immunology, Massachusetts General Hospital, Boston, Mass, USA.

This document is not intended to act as a prescriptive guideline for drug challenge or desensitization protocols. The objective of this supplementary text is not to review current evidence but to share personal experience. Local guidelines and guidelines of the corresponding national Allergy Societies should always be adhered to, and protocols should be adapted to the local population, local requirements, and local resources.

Macrolide is a popular class of antibiotics that works by binding the 50S subunit of bacterial ribosomes, leading to inhibition of transpeptidation, translocation, chain elongation, and, bacterial protein synthesis[1–3]. There are 3 major members, the original Erythromycin, the subsequent Clarithromycin, and the popular Azithromycin. Macrolides can be classified according to the number of carbon atoms in their lactone ring: 14 membered (erythromycin, troleandomycin, roxithromycin, dirithromycin, and clarithromycin), 15 membered (azithromycin), and 16 membered (spiramycin, rokitamycin, josamycin, and midecamycin).

Macrolides are widely used to treat respiratory infections, including community acquired pneumonia (CAP), pertussis, atypical mycobacterial infections, and mycoplasma infections. They are important component in the treatment of Helicobacter pylori infections. We have used them as prophylactic antibiotic for refractory chronic sinusitis and immunodeficiency. They are thought to have anti-inflammatory property in addition to their antibiotic property[4,5].

Adverse side effects to macrolides, especially GI discomfort, are common[3]. True hypersensitivity reactions are infrequent and are generally comprised of various forms of dermatologic manifestation such as urticaria and nonspecific drug exanthem[3].

In most cases, alternative antibiotics are available for reactive patient. However, in seven patients, we deemed it was important to establish whether the patient can tolerate a macrolide antibiotic as a macrolide was either the optimal antibiotic for their infection/prophylaxis or we needed additional antibiotic choices for their complex infections. The HSR spectrum to the macrolide antibiotics in these 7 patients is detailed in Table 1. Five of the seven patients had cutaneous reactions (2 immediate and 3 delayed), and 1 each had oral swelling, bronchospasm, delayed joint swelling, and GI bleed alone or

in combinations. However, our sample size is rather small to make any definitive conclusions, but it seems similar to experiences by other authors[6].

The initial drug causing HSR was Erythromycin in 2 patients, Clarithromycin in 3 patients, and Azithromycin in 3 patients with 1 patient had reaction to both Clarithromycin and Azithromycin. We did not perform skin test with the macrolide antibiotic as the accuracy of skin test is still not established though concentration thresholds have been established in the literature (0.01mg/ml for Azithromycin and 0.05mg/ml for Clarithromycin)[7]. The sensitivity of skin tests with macrolides is low; therefore, challenges with the suspected macrolides often are necessary in order to establish a firm diagnosis or to identify safe alternatives[8,9]. Regarding cross-reactivity, macrolide hypersensitivity is unlikely to be a class hypersensitivity. Indeed, in subjects with hypersensitivity to a given macrolide who needed an alternative compound, challenges with alternative macrolides proved to be safe and effective[8–10].

We proceeded to direct challenge with Azithromycin in all seven patients as Azithromycin has the lowest incidence of GI side effects, low drug-interaction, simple regimens, inexpensive, and readily available. This included the 3 patients who had reaction to Azithromycin initially. The time elapsed since the initial reaction ranged from 2-47 years and indeterminate in 2 patients. We have used 2 versions of the challenge protocol: a rapid simple single 250mg dose challenge (observed for 3 additional hrs) and a 500 mg modified protocol. Six of the seven patients tolerated the challenge well. One of the patient, starting about an hour into the challenge, felt waves of slightly woozy and difficulty concentrating, mildly flushed sensation intermittently, and mild GI sickish sensation ("wave of something."). There was no progression over an additional 2 hours. This was likely side effect/anxiety as she tolerated a subsequent course of Azithromycin. Hence all seven patients were able to take Azithromycin subsequently. This illustrated that most patients with HSR to macrolide antibiotics would lose their sensitivity and able to tolerate Azithromycin several years later. No desensitization to the macrolide antibiotics was needed.

TABLES:

Table 1. HSR to macrolide antibiotics (n=7) who needed challenge

HSR Type	Number of patients	Percentage of patients (%)
Immediate	2	29%
Delayed	3	43%
Indeterminate	2	29%
Nasal/ocular	0	0%
Oral/pharyngeal	1	14%
Cardiovascular	0	0%
Pulmonary	1	14%
GI (bleeding)	1	14%
Musculoskeletal/rheumatologic	1	14%
Skin-rapid onset	2	29%
Skin-delay onset	3	43%

Table 2. Azithromycin Oral Challenge Protocols

- Go over interval history
- Consent obtained
- Check baseline PFT and BP

250 mg protocol (rapid) (n=5)

0:00	250 mg tab	
Observed for additional 2-3 hr		

500 mg protocol (extended) (n=2)

0:00	125 mg ½ of 250 mg tab	
0:30	125 mg ½ of 250 mg tab	
1:00	250 mg tab	
Observed for additional 2 hr		

- Recheck BP and PFT if reaction occur
- Treat any adverse reactions deemed necessary.

REFERENCES:

1. Sturgill MG, Rapp RP. Clarithromycin: Review of a new macrolide antibiotic with improved microbiologic spectrum and favorable pharmacokinetic and adverse effect profiles. *Ann Pharmacother*. 1992;26(9):1099-1108. doi:10.1177/106002809202600912

2. Rapp RP, McCraney SA, Goodman NL, Shaddick DJ. New macrolide antibiotics: Usefulness in infections caused by mycobacteria other than Mycobacterium tuberculosis. *Ann Pharmacother*. 1994;28(11):1255-1263. doi:10.1177/106002809402801109

3. Graziani AL. Azithromycin and clarithromycin. UpToDate. https://www.uptodate.com/contents/azithromycin-and-clarithromycin. Published May 11, 2021. Accessed June 24, 2021.

4. Cramer CL, Patterson A, Alchakaki A, Soubani AO. Immunomodulatory indications of azithromycin in respiratory disease: a concise review for the clinician. *Postgrad Med*. 2017;129(5):493-499. doi:10.1080/00325481.2017.1285677

5. Takizawa H, Desaki M, Ohtoshi T, et al. Erythromycin suppresses interleukin 6 expression by human bronchial epithelial cells: A potential mechanism of its anti-inflammatory action. *Biochem Biophys Res Commun*. 1995;210(3):781-786. doi:10.1006/bbrc.1995.1727

6. Sánchez-Borges M, Thong B, Blanca M, et al. Hypersensitivity reactions to non beta-lactam antimicrobial agents, a statement of the WAO special committee on drug allergy. *World Allergy Organ J*. 2013;6(1):1-23. doi:10.1186/1939-4551-6-18

7. Mori F, Barni S, Pucci N, et al. Sensitivity and specificity of skin tests in the diagnosis of clarithromycin allergy. *Ann Allergy, Asthma Immunol*. 2010;104(5):417-419. doi:10.1016/j.anai.2010.03.010

8. Benahmed S, Scaramuzza C, Messaad D, Sahla H, Demoly P. The accuracy of the diagnosis of suspected macrolide antibiotic hypersensitivity: Results of a single-blinded trial. *Allergy Eur J Allergy Clin Immunol*. 2004;59(10):1130-1133. doi:10.1111/j.1398-9995.2004.00638.x

9. Ünal D, Demir S, Gelincik A, et al. Diagnostic Value of Oral Challenge Testing in the Diagnosis of Macrolide Hypersensitivity. *J Allergy Clin Immunol Pract*. 2018;6(2):521-527. doi:10.1016/j.jaip.2017.06.036

10. Macy E, Romano A, Khan D. Practical Management of Antibiotic Hypersensitivity in 2017. *J Allergy Clin Immunol Pract*. 2017;5(3):577-586. doi:10.1016/j.jaip.2017.02.014
