# Supplementary material for: Standards for practical intravenous rapid drug desensitization & delabeling: A WAO committee statement
Source: World Allergy Organ J. 2022 May 31;15(6):100640. doi: 10.1016/j.waojou.2022.100640 (PMC9163606; doi:10.1016/j.waojou.2022.100640)
Supplement: Multimedia component 9 [file mmc9.pdf]

SUPPLEMENTARY TEXT 9

Rapid Desensitization Protocols for HSR to Other Non-Antibiotic and Non-Chemo Drugs (Adopted from Vancomycin and Aspirin Desensitization Protocols)

Johnson T. Wong, M.D.

Division of Rheumatology, Allergy and Immunology, Massachusetts General Hospital, Boston, Mass, USA.

This manuscript is not intended to act as a prescriptive guideline for drug challenge or desensitization protocols. Local guidelines and guidelines of the corresponding national Allergy Societies should always be adhered to, and protocols should be adapted to the local population, local requirements, and local resources. There is an array of protocols for many of these drugs that have been validated in large series of well-characterized patients (i.e., after drug challenge), especially for Aspirin. However, the objective of this supplementary text is not to review current evidence but to share personal experience.

The incremental dose escalation protocols that we established for Vancomycin has been modified for desensitization to many other medications[1]. We will not discuss the protocols for chemo-oncologic drugs as they are covered in detail in their dedicated sections by our coauthor. We are presenting their usage for desensitization for non-antibiotic, non-chemo drugs. The Aspirin/NSAID protocols have been utilized in over 30 patients whereas the other protocols were utilized in 1-5 patients each.

Aspirin Oral Desensitization Protocol

Time (hr:min)	Dose (mg)	Solution (mg/ml) or tablet (81mg/tab)	Volume (ml) or number of tablet	Cumulative Dose (mg)	Reaction
0:00	0.1	0.1	1	0.1	
0:15	0.3	0.1	3	0.4	
0:30	1	1	1	1.4	
0:45	3	1	3	4.4	
1:00	10	1	10	14.4	
1:15	20	1	20	34.4	
1:30	40	1	40	74.4	
1:45	81	tablet	1	155.5	
2:00 (optional)	162	tablet	2	317.5	
2:15 (optional)	324	tablet	4	641.5	

Based on our published experience[2].

1. Dissolve chewable baby aspirin (81mg) in 81 ml of water to make 1mg/ml stock solution.
2. Dilute 1:10 to make 0.1mg/ml solution by taking 3ml of 1mg solution to 27ml of water.
3. Check vital signs prior to protocol and periodically as needed.
4. Perform pulmonary, cardiac, throat, and cutaneous examinations prior to protocol and periodically as needed.

Balsalazide (Colazal) Oral Desensitization Protocol (5 Day)

Day	Time	Concentration (mg/cc)	Volume	Drug amount	Reaction
1	0	0.75mg/cc	1 cc	0.75mg	
	2hr	0.75mg/cc	3 cc	2.25mg	
	4hr	0.75mg/cc	10 cc	7.5mg	
2	0	7.5mg/cc	3 cc	22.5mg	
	2hr	7.5mg/cc	10 cc	75mg	
	4hr	7.5mg/cc	20 cc	150mg	
3	0	75mg/cc	4 cc	300mg	
	2hr	75mg/cc	8 cc	600mg	
	4hr	750mg cap	1 capsule	750mg	
4	AM	750mg cap	1 capsule	750mg	
	Noon	750mg cap	1 capsule	750mg	
	PM	750mg capsule	1 capsule	750mg	
5	AM	750mg capsule	2 capsules	1500mg	
	PM	750mg capsule	2 capsules	1500mg	

- Dissolve 2 of the 750mg capsule and suspend in 20cc of water to made the 75mg/cc suspension
- 3 cc of 75mg/cc suspension add to 27cc of water to made the 7.5mg/cc suspension
- 3 cc of 7.5mg/cc suspension add to 27cc of water to made the 0.75mg/cc suspension
- Obtain informed consent.
- Examine vital signs, oral mucosa, skin, and chest prior to start
- Shake up each suspension well prior to taking out appropriate amount with appropriate size syringe for taking
- Monitor temperature, skin, GI side effect, or other adverse symptoms.
- If GI or skin side effect occurs, then repeat that dose or cut down to previous dose. Lengthen protocol accordingly.

Clopidogrel (Plavix) Oral Desensitization Protocol-8 Hr

Dose	Time (hr)	Concentration (mg/cc)	Volume	Reaction
1	0	0.1	0.3cc	
2	1	0.1	1 cc	
3	2	0.1	3 cc	
4	3	0.1	10 cc	
5	4	1	3 cc	
6	5	1	10 cc	
7	6	1	20 cc	
8	7	1	40 cc (remaining of bottle)	
Day 2 onward		75mg tablet	1 tablet	

- As suspension is not commercially available, pharmacy will need to prepare and label 1mg/cc suspension by crushing/dissolving one 75mg tablet of Plavix (Clopidogrel) into 75cc of water.
- Dilute 2 cc of the 1mg/cc Plavix (Clopidogrel) suspension from pharmacy to 18cc of water to make 0.1mg/cc suspension
- Obtain informed consent.
- Examine vital signs, oral mucosa, skin, and chest prior to start
- Shake up each suspension well prior to taking out appropriate amount with appropriate size syringe for taking
- Monitor temperature, skin, GI side effect, or other adverse symptoms.
- If GI side effect occurs, then repeat that dose or cut down to previous dose. Lengthen protocol accordingly.

Clopidogrel (Plavix) Oral Desensitization Protocol- 5 Day

Day	Time	Concentration (mg/cc)	Volume	Reaction
1	AM	0.1	0.3cc	
	PM	0.1	1 cc	
2	AM	0.1	3 cc	
	PM	0.1	10 cc	
3	AM	1	3 cc	
	PM	1	10 cc	
4	AM	1	20 cc	
	PM	1	40 cc (remaining of bottle)	
5		75mg tablet	1 tablet	
6 onward		75mg tablet	1 tablet	

- As suspension is not commercially available, pharmacy will need to prepare and label 1mg/cc suspension by crushing/dissolving one 75mg tablet of Plavix (Clopidogrel) into 75cc of water.
- Dilute 2 cc of the 1mg/cc Plavix (Clopidogrel) suspension from pharmacy to 18cc of water to make 0.1mg/cc suspension
- Obtain informed consent.
- Examine vital signs, oral mucosa, skin, and chest prior to start
- Shake up each suspension well prior to taking out appropriate amount with appropriate size syringe for taking
- Monitor temperature, skin, GI side effect, or other adverse symptoms.
- If GI side effect occurs, then repeat that dose or cut down to previous dose. Lengthen protocol accordingly.

Clopidogrel (Plavix) Oral Desensitization Protocol-9 Day

Day	Time	Concentration (mg/cc)	Volume	Reaction
1		0.1	0.3cc	
2		0.1	1 cc	
3		0.1	3 cc	
4		0.1	10 cc	
5		1	3 cc	
6		1	10 cc	
7		1	20 cc	
8		1	40 cc (remaining of bottle)	
9		75mg tablet	1 tablet	
10 onward		75mg tablet	1 tablet	

- As suspension is not commercially available, pharmacy will need to prepare and label 1mg/cc suspension by crushing/dissolving one 75mg tablet of Plavix (Clopidogrel) into 75cc of water.
- Dilute 2 cc of the 1mg/cc Plavix (Clopidogrel) suspension from pharmacy to 18cc of water to make 0.1mg/cc suspension
- Obtain informed consent.
- Examine vital signs, oral mucosa, skin, and chest prior to start
- Shake up each suspension well prior to taking out appropriate amount with appropriate size syringe for taking
- Monitor temperature, skin, GI side effect, or other adverse symptoms.
- If GI side effect occurs, then repeat that dose or cut down to previous dose. Lengthen protocol accordingly.

Ethacrynic acid (Edecrin) Oral Desensitization/Challenge Protocol

Time	Concentration	Amount	Cumulative Dose
0:00	5mg/ml	0.4cc	2.0mg
0:30		0.8cc	6.0mg
1:00		1.6	19mg
1:30		2.2	25mg

- Crush and dissolve 25mg tab in 5cc of water = 5mg/ml
- Go over interval history
- Consent obtained
- Check baseline PFT and BP
- Administer Edecrin per above protocol (make adjustments depending on final dose)
- Observe for additional 1-2 hrs.
- Recheck BP and PFT if reaction occur
- Treat any adverse reactions deemed necessary.

Hydroxychloroquine (Plaquenil) Oral Desensitization Protocol

Day	Time	Concentration (mg/cc)	Volume	Reaction
1	AM	(0.2mg/cc)	1 cc	
	Noon	(0.2mg/cc)	3 cc	
	PM	(0.2mg/cc)	10 cc	
2	AM	(2mg/cc)	3 cc	
	Noon	(2mg/cc)	10 cc	
	PM	(2mg/cc)	30 cc	
3	AM	Tablet (200mg)	¼ tablet	
	Noon	Tablet	½ tablet	
	PM	Tablet	1	
4	AM	200mg tablet	1	
	PM	200mg tablet	1	

- Grind one 200mg tablet of Plaquenil and suspend in 100cc sterile water to make a 2mg/cc suspension.
- Take 10cc of the 2mg/cc suspension and add to 90cc of sterile water to make a 0.2mg/cc suspension.
- Obtain informed consent.
- **Shake up each suspension well prior to taking out appropriate amount with appropriate size syringe for taking**

Lamictal Oral Desensitization Protocol

Day	Time	Concentration (mg/cc)	Volume	Reaction
1	0hr	(0.025mg/cc)	1 cc	
	1hr	(0.025mg/cc)	3 cc	
	2hr	(0.25mg/cc)	1 cc	
	3hr	(0.25mg/cc)	3 cc	
2	0hr	5mg tab	½ tab	
	1hr	5 mg tab	1 tab	
	2hr	5mg tab	2 tab	
	3hr	5mg tab	2 tab	
3-9<br>(home)		25mg tablet	1 tab	
10-16<br>(day 10 at our office, rest at home)		25mg tablet	2 tab	
17-23<br>(day 17 at our office, rest at home)		25mg tablet	3 tab	
24 on<br>(day 24 at our office, rest at home)		25mg tablet	4 tab	

- Dissolve 5mg chewable tablet of Lamictal and suspend in 20cc sterile water to make a 0.25mg/cc suspension.
- Take 10cc of the .25mg/cc suspension and add to 90cc of sterile water to make a 0.025mg/cc suspension.
- Obtain informed consent.
- **Shake up each suspension well prior to taking out appropriate amount with appropriate size syringe for taking**

Methadone Oral Desensitization Protocol (5 Day)

**Day**	**Time**	**Concentration (mg/cc)**	**Volume**	**Reaction**
1	AM	0.02mg/cc = 1/100	1 cc	
	PM	0.02mg/cc = 1/100	3 cc	
2	AM	0.02mg/cc = 1/100	10 cc	
	PM	0.2mg/cc = 1/10	3 cc	
3	AM	0.2mg/cc = 1/10	10 cc	
	PM	0.2mg/cc = 1/10	20 cc	
4	AM	2mg/cc=full strength	2.5cc	
	PM	2mg/cc=full strength	2.5 cc	
5	AM	5mg tablet	1	
	PM	5mg tablet	1	

- Dilute 1cc of methadone suspension 2 mg/cc from pharmacy to 100cc to make 0.02mg/cc (1/100) suspension
- 10 cc of methadone suspension 2 mg/cc add to 90 cc of water to make the 0.2 mg/cc (1/10) suspension
- Obtain informed consent.
- Examine vital signs, oral mucosa, skin, and chest prior to start
- Shake up each suspension well prior to taking out appropriate amount with appropriate size syringe for taking
- Monitor temperature, skin, GI side effect, or other adverse symptoms.
- If itching side effect occurs, then repeat that dose or cut down to previous dose. Lengthen protocol accordingly.

Methadone Oral Desensitization Protocol (15 Day)

Day	Time	Concentration (mg/cc)	Volume	Reaction
1&2	AM	0.02mg/cc = 1/100	1 cc	
	PM	0.02mg/cc = 1/100	1 cc	
3&4	AM	0.02mg/cc = 1/100	3 cc	
	PM	0.02mg/cc = 1/100	3 cc	
5&6	AM	0.02mg/cc = 1/100	10 cc	
	PM	0.02mg/cc = 1/100	10 cc	
7&8	AM	0.2mg/cc = 1/10	3 cc	
	PM	0.2mg/cc = 1/10	3 cc	
9&10	AM	0.2mg/cc = 1/10	10 cc	
	PM	0.2mg/cc = 1/10	10 cc	
11&12	AM	0.2mg/cc = 1/10	20 cc	
	PM	0.2mg/cc = 1/10	20 cc	
13&14	AM	2mg/cc=full strength	2.5cc	
	PM	2mg/cc=full strength	2.5cc	
15 on	AM	5mg tablet	1	

- Dilute 1cc of Methadone suspension 2mg/cc from pharmacy to 100cc to make 0.02mg/cc (1/100) suspension
- 10 cc of methadone suspension 2 mg/cc add to 90 cc of water to make the 0.2 mg/cc (1/10) suspension
- Obtain informed consent.
- Examine vital signs, oral mucosa, skin, and chest prior to start
- Shake up each suspension well prior to taking out appropriate amount with appropriate size syringe for taking
- Monitor temperature, skin, GI side effect, or other adverse symptoms.
- If itching side effect occurs, then repeat that dose or cut down to previous dose. Lengthen protocol accordingly.

Metronidazole Oral Desensitization/Challenge Protocol (3 Hr)

Time (hr:min)	Dose (mg)	Solution (mg/ml) or tablet (250mg/tab)	Volume (ml) or number of tablet	Cumulative Dose (mg)	Reaction
0:00	0.1	0.1	1	0.1	
0:20	0.3	0.1	3	0.4	
0:40	1	1	1	1.4	
1:00	3	1	3	4.4	
1:20	10	10	1	14.4	
1:40	30	10	3	44.4	
2:00	60	10	6	104	
2:20	120	10	12	224	
2:40	250	tablet	1	474	

1. Dissolve content of Metronidazole 375mg capsule in 37.5 ml of water to make 10mg/ml stock solution/suspension. Alternatively crush 250mg tab and dissolve in 25ml of water to make 10mg/ml solution/ suspension.
2. Dilute 1:10 to make 1mg/ml solution by taking 3ml of 10mg solution/suspension (shake well) to 27ml of water.
3. Dilute 1:10 to make 0.1mg/ml solution by taking 3ml of 1mg solution (shake well) to 27ml of water.
4. Check vital signs prior to protocol and periodically as needed.
5. Perform pulmonary, cardiac, throat, and cutaneous examinations prior to protocol and periodically as needed.

Addendum:

- **Montelukast (Singulair) 10mg po given prior to desensitization**
- **Fexofenadine (Allegra) 180mg or Cetirizine (Zyrtec) 10mg po given prior to desensitization.**

Sulfadiazine Oral Desensitization Protocol (9 Day)

**Day**	**Time**	**Concentration (mg/cc)**	**Volume**	**Drug amount**	**Reaction**
1	AM	0.025mg/cc	1 cc	0.05mg	
	PM	0.025mg/cc	3 cc	0.15mg	
2	AM	0.025mg/cc	10 cc	0.5mg	
	PM	0.25mg/cc	3 cc	1.5mg	
3	AM	0.25mg/cc	10 cc	5mg	
	PM	0.25mg/cc	20 cc	10mg	
4	AM	2.5mg/cc	4 cc	20mg	
	PM	2.5mg/cc	8 cc	40mg	
5	AM	2.5mg/cc	15cc	75mg	
	Noon	2.5mg/cc	30cc	150mg	
	6PM	2.5mg/cc	60cc	300mg	
6	AM	250mg capsule	1 capsule	500mg	
	Noon	250mg capsule	1 capsule	500mg	
	6PM	250mg capsule	1 capsule	500mg	
7	AM	500mg capsule	1 capsule	500mg	
	Noon	500mg capsule	1 capsule	500mg	
	6PM	500mg capsule	1 capsule	500mg	
	Bed-time	500mg capsule	1 capsule	500mg	
8	AM	500mg capsule	2 capsules	1000mg	
	Noon	500mg capsule	1 capsule	500mg	
	6PM	500mg capsule	2 capsules	1000mg	
	Bed-time	500mg capsule	1 capsule	500mg	
9	AM	500mg capsule	2 capsules	1000mg	

	Noon	500mg capsule	2 capsules	1000mg	
	6PM	500mg capsule	2 capsules	1000mg	
	Bed-time	500mg capsule	2 capsules	1000mg	

- Crush 500mg capsule and suspend in 100cc of water to made the 5mg/cc suspension
- 10cc of 5mg/cc suspension add to 90cc of water to made the 0.5mg/cc suspension
- 10cc of 0.5mg/cc suspension add to 90cc of water to made the 0.05mg/cc suspension
- Obtain informed consent.
- Examine vital signs, oral mucosa, skin, and chest prior to start
- Shake up each suspension well prior to taking out appropriate amount with appropriate size syringe for taking
- Monitor temperature, skin, GI side effect, or other adverse symptoms.
- If GI side effect occurs, then repeat that dose or cut down to previous dose. Lengthen protocol accordingly.

Zocor Oral Desensitization/Challenge Protocol (8 Day)

Day	Volume (cc)	Dose (mg)
1	0.5	0.5
2	1	1
3	2	2
4	4	4
5	8	8
6	1 tablet	10
7	2 tablet	20
8	4 tablet	40

- 10mg tablet broken into fine powder and resuspended in 10cc sterile water to make 1mg/cc suspension. This will be used for the first 5 days. The suspension should be shaken up each day prior to taking.
- Patient may then switch over to 40mg tablet.
- Repeat dose or decrease 1-2 steps if problem occurs.

REFERENCES:

1. Wong JT, Ripple RE, MacLean JA, Marks DR, Bloch KJ. Vancomycin hypersensitivity: Synergism with narcotics and "desensitization" by a rapid continuous intravenous protocol. *J Allergy Clin Immunol*. 1994;94(2):189-194. doi:10.1016/0091-6749(94)90039-6

2. Wong JT, Nagy CS, Krinzman SJ, MacLean JA, Bloch KJ. Rapid oral challenge-desensitization for patients with aspirin-related urticaria-angioedema. *J Allergy Clin Immunol*. 2000;105(5):997-1001. doi:10.1067/mai.2000.104571
