# Supplementary material for: Standards for practical intravenous rapid drug desensitization & delabeling: A WAO committee statement
Source: World Allergy Organ J. 2022 May 31;15(6):100640. doi: 10.1016/j.waojou.2022.100640 (PMC9163606; doi:10.1016/j.waojou.2022.100640)
Supplement: Multimedia component 10 [file mmc10.pdf]

SUPPLEMENTARY TEXT 10

Intravenous Rapid Drug Desensitization in Corticosteroid Allergy

Dr María Antonieta Guzmán Meléndez

Servicio de Inmunología y Alergias, Hospital Clínico Universidad de Chile, Santiago, Chile

Javier Cuesta-Herranz MD, PhD

Fundación IIS-Fundación Jiménez Díaz, Retic ARADyAL (RD16/0006/0013), Madrid (Spain).

Corticosteroids are widely used in allergic and inflammatory conditions (1). They have a variety of uses, for example, they are used as a premedication for drug hypersensitivity reactions (DHRs) to contrast media, they might have a role in the management of the late-phase of anaphylaxis, and they have immunosuppression and antiproliferative effects, which explains why they are widely used for the treatment of allergic diseases (such as asthma), autoimmune conditions, or tumors and transplants.

Immediate DHRs (I-DHRs) to corticosteroids have a relatively low prevalence in the range of 0.1% to 0.3%, whereas non-immediate DHRs (NI-DHRs) show prevalences of up to 5% mainly explained by contact dermatitis caused by topical corticosteroids.

It is unclear whether I-DHRs are caused by sensitization to the corticosteroid moiety or by the haptenization of metabolites. Some patients show positive specific immunoglobulin E (IgE) or positive skin tests (STs) to corticosteroids, and there are reports of positive histamine release basophil activation tests, which suggests that an IgE-mediated mechanism could be involved (3). However, some patients might have negative results to these tests, thus suggesting a non-IgE-mediated or "pseudoallergic" DHR (4).

Some patients have a history of previous exposure to the same culprit corticosteroid, where others might have only a history of exposure to different corticosteroids (5-7), or no previous exposure whatsoever (8). Cross-reactivity has been identified within the same corticosteroid groups as well as between groups. Cross-reactivity seems to be less frequent with triamcinolone, amcinonide, halcinonide, fluocinonide, and desonide (9). Other studies suggest a greater prevalence of contact dermatitis with non-fluorinated corticosteroids (hydrocortisone, hydrocortisone-17-butyrate, and budesonide), adding that the lateral chain C17 of hydrocortisone could potentially be involved in the process of protein binding and would be the antigenic site (10).

I-DHRs to corticosteroids may feature itch, urticaria, rash, angioedema, difficulty in breathing (whether nasally, pharyngeal, or bronchial), hypotension, and anaphylactic shock (11). There are several administration routes available, namely, intravenous, intraarticular, intramuscular, intralesional, epidural, oral, inhaled, and topical. Severe anaphylaxis caused by DHRs to corticosteroids is usually linked to the intravenous route, followed by the intraarticular and intramuscular routes (12).

A review by Patel, et al. (12) covering 11 years, from January 2004 to December 2014, found that most I-DHRs are caused by methylprednisolone (49 cases), prednisolone (24 cases), triamcinolone (17 cases), hydrocortisone (12 cases), betamethasone (6 cases), dexamethasone (5 cases), and prednisone (5 cases). Two I-DHRs were linked to the use of inhaled corticosteroids (mometasone in one case, and fluticasone, in combination with salmeterol, in another).

DHRs with certain formulations of corticosteroids can be caused by the added excipients (13). Succinate esters are used to facilitate the solubility of corticosteroids, given their hydrophobic nature. Other excipients are carboxymethylcellulose (an emulsifying and thickening agent), polyethyleneglycol (a surfactant, solvent and dispersing agent), and lactose (derived from milk, hence can potentially be contaminated by cow's milk proteins).

There are only seldom cases of intravenous rapid drug desensitization (RDD) to corticosteroids in the literature. This procedure is used when there is no alternative treatment and the culprit corticosteroid is needed.

(i) Angel-Pereira, et al. published a case report of a 34 years old gentleman with a multiple sclerosis relapse (14). Remarkably, this is the first published case including a confirmatory drug provocation test before attempting desensitization.

In this case report, the patient required urgent administration of methylprednisolone sodium hemisuccinate (MSH) to control his neurologic symptoms. He had five planned 1 gram intravenous doses (administered in 250 ml of a saline solution in a daily one-hour infusion). Fifteen minutes after starting the first administration he came up in widespread urticaria, which was controlled by stopping the infusion. He was urgently referred to the allergy department and skin testing (skin prick testing with all of the following corticosteroids, and intradermal testing only with those cortecosteroids with intravenous formulations available) was performed with MSH (Solumoderin, Pfizer S.L, Madrid, Spain), unesterified methylprednisolone, betamethasone, dexamethasone, triamcinolone, prednisolone, prednisone, and hydrocortisone. Skin testing with all these drugs came back negative. A drug provocation test (DPT) was performed with MSH and a similar urticarial I-DHR was confirmed. The patient's neurologists considered that MSH was the first-choice treatment in this gentleman, and thus

RDD to MSH was programmed. Of note, the succinate formulation of methylprednisolone increases its solubility in water for intravenous use. The patient was successfully desensitized using a 12-step protocol, which lasted 5.66 hours. The initial dose was 0.02 mg and was gradually increased to reach a cumulative dose of 1,000 mg. Three different solution bags were used, with increasing concentrations of the drug, 0.04 mg/ml, 0.4 mg/ml and 3.969 mg/ml. Each solution bag was administered in four sequential steps with progressively higher doses per step for a total of 12 steps.

Notably, this is the first time a diagnostic DPT was performed before an RDD to corticosteroids. Furthermore, there was a systematic approach to offering safe alternatives to the patient. DPT was performed with 1 g of methylprednisolone orally (Urbason, Sanofi-Aventis S.P.A, Barcelona, Spain), and 8 mg of dexamethasone sodium phosphate intravenously (KERN PHARMA S.L, Barcelona, Spain). Both DPTs were negative. To further study the causal link to sodium succinate, a DPT was completed with 20 grams of succinate gelatin solution in 500 ml saline (Gelafundina, B. Braun. Melsungen, Germany) infused in one hour. No reactions were observed. DPT confirmed tolerance to other unlikely alternatives and even the suspected excipient.

Mechanistic reasoning not based on evidence (i.e. attributing the allergy to the methylprednisolone moiety or the excipient with no confirmatory tests) could have made other groups opt for unnecessarily avoiding all drugs containing sodium succinate. However, this group recommended making use of the DPT, whenever possible, before irreversibly and unnecessarily making tentative recommendations of drug avoidance.

(ii) Lee-Wong et al. (15) reported the case of a 62 years old lady who presented with widespread urticaria after the intraarticular injection of an unknown corticosteroid. Interestingly, she had previously experienced an anaphylactoid DHR to a contrast medium. At the time of the report, she had comorbidities (final stage renal impairment, cardiac and coronary disease, high blood pressure, and chronic cholecystitis) that made a cardiac catheterism with contrast medium necessary. Premedication with corticosteroids was considered necessary. Therefore, the patient underwent skin testing with three corticosteroids (hydrocortisone, methylprednisolone and dexamethasone), and all these tests came back positive. Her doctors opted for administering hydrocortisone (reasoning that it was the drug with a shorter half-life of 80-118 minutes, and skin testing was less intensively positive).

The RDD was successful. The authors used a protocol with 18-steps of progressively higher doses of intravenous hydrocortisone at 15-minutes intervals (with an extra 5 minutes on each step for the infusion of the drug), which lasted for 4.5 hours. The initial dose was 0.001 mg and the target cumulative dose was 256 mg. On the third dose, the patient experienced mild itch and erythema, but she received 50 mg of diphenhydramine and was able to finish the procedure uneventfully.

After finalising the protocol, the patient received full doses of intravenous hydrocortisone 200 mg every 4 hours (in 3-5 minute boluses from a hydrocortisone dilution of 50 mg/ml). For an intermittent administration, the drug had to be used in a diluted form. In this patient, there was no DPT before the RDD. There are no data on sensitization to the excipients of the corticosteroid formulation.

(iii) Subsequently, Guvenir et al. published the first case report of an RDD to corticosteroids in a pediatric patient (16). This was the case of a 6 years old boy admitted as an inpatient for Schoenlein-Henoch purpura, who was planned for 1 gram boluses (infused over an hour) of methylprednisolone sodium succinate (MSS). On the second day of therapy, he experienced facial angioedema, shortness of breath, low blood pressure, nausea, and vomiting within 20 minutes of starting the infusion. The drug infusion was stopped and the patient received intramuscular adrenaline and hydroxyzine. No skin testing or DPT were performed with MSS, but the patient required this drug and no other appropriate alternatives were found, so the patient was programmed for RDD with MSS following a modification of the protocol published by Angel-Pereira et al. (14). The patient did not present with any further adverse reactions during the RDD procedure or immediately after with the following doses of the drug (either intravenous or orally).

1) Liu D, Ahmet A, Ward L, Krishnamoorthy P, Mandelcorn ED, Leigh R, et al. A practical guide to the monitoring and management of the complications of systemic corticosteroid therapy. Allergy Asthma Clin Immunol 2013; 9: 30-54

2) Baeck M, Marot L, Nicolas JF, Pilette C, Tennstedt D, Goossens A. Allergic hypersensitivity to topical and systemic corticosteroids: a review. Allergy 2009; 64: 978-94

3) Said B, Leray V, Nicolas JF, Rozieres A, Berard F. Methylprednisolone-induced anaphylaxis: diagnosis by skin testand basophil activation test. Allergy 2010; 65: 531-532

4) Dajani BM, Sliman NA, Shubair KS, Hamzeh YS. Bronchospasm caused by intravenous hydrocortisone sodium succinate (Solu-Cortef) in aspirin-sensitive asthmatics. J Allergy Clin Immunol 1981; 68: 201-204

5) Butani L. Corticosteroid-induced hypersensitivity reactions. An Allergy Asthma Immunol 2002; 89 (5): 439-45

6) Karsh J, Yang WH. An anaphylactic reaction to intra-articular triamcinolone: a case report and review of the literature. An Allergy Asthma Immunol 2003; 90: 254-258

7) Mace S, Vadas P, Pruzanski W. Anaphylactic shock induced by intraarticular injection of methylprednisolone acetate. J Rheumatol 1997; 24: 1191-4

8) Pryse-Phillips WE, Chandra RK, Rose B. Anaphylactoid reaction to methylprednisolone pulsed therapy for multiple sclerosis. Neurology 1984; 34: 1119-21

9) Lepoittevin JP, Drieghe J, Dooms-Goossens A. Studies in patients with corticosteroids contact allergy. Understanding cross-reactivity among different steroids. Arch Dermatol 1995; 131: 31-37

10) Wilkinson SM, English JS. Hydrocortisone sensitivity. An investigation into the nature of the allergen. Contact Dermatitis 1991; 25: 178-181

11) Vatti RR, Ali F, Teuber S. Hypersensitivity reactions to corticosteroids. Clin Rev Allergy Immunol 2014; 47: 26-37

12) Patel A, Bahna S. Immediate hypersensitivity reactions to corticosteroids. Ann Allergy Asthma Immunol 2015; 115: 178-182

13) Li PH, Wagner A, Thomas I, Watts T, Rutkowski R, Rutkowski K. Steroid Allergy: Clinical Features and the Importance of Excipient Testing in a Diagnostic Algorithm. J Allergy Clin Immunol Pract 2018; 6: 1655-61

14) Angel-Pereira D, Berges-Gimeno M, Madrigal-Burgaleta R, Ureña-Tavera M, Zamora-Verduga M, Alvarez-Cuesta E. Successful rapid desensitization to methylprednisolone sodium hemisuccinate: A case report. J Allergy Clin Immunol Pract 2014: 2; 346-348

15) Lee-Wong M, McClelland S, Chong K, Fernandez E. A case of hydrocortisone desensitization in a patient with radiocontrast-induced anaphylactoid reaction and corticosteroid allergy. Allergy Asthma Proc 2006, 27: 265-268

16) Guvenir H, Dibek E, Aydin F. Successful Methylprednisolone Desensitization in a Pediatric Patient. Pediatr Allergy Immunol 2016; 28 (3): 305-6
