# Supplementary material for: Standards for practical intravenous rapid drug desensitization & delabeling: A WAO committee statement
Source: World Allergy Organ J. 2022 May 31;15(6):100640. doi: 10.1016/j.waojou.2022.100640 (PMC9163606; doi:10.1016/j.waojou.2022.100640)
Supplement: Multimedia component 11 [file mmc11.pdf]

SUPPLEMENTARY TEXT 11

Desensitization in Intravenous Iron Hypersensitivity

Javier Cuesta-Herranz MD, PhD

Fundación IIS-Fundación Jiménez Díaz, Retic ARADyAL (RD16/0006/0013), Madrid (Spain).

Dr María Antonieta Guzmán Meléndez

Servicio de Inmunología y Alergias, Hospital Clínico Universidad de Chile, Santiago, Chile

Current intravenous iron formulations include high or low molecular weight iron dextran (IDX), ferric gluconate, iron isomaltoside (ISM), iron sucrose/saccharate (IS), ferumoxytol and ferric carboxymaltose (FCM). All share a similar, albeit for each compound unique, structure, containing a core of iron-hydroxide gel encased in a carbohydrate shell which serves to stabilize the core and regulate the speed of iron release. It has been reported that ferric carboxymaltose is the most effective intravenous iron formulation, followed by iron sucrose (1). In addition, ferric carboxymaltose tends to be better tolerated (1).

Hypersensitivity reactions to IV iron are extremely rare (2). Two main immunological responses have been described for iron hypersensitivity reactions (HSRs): IgE-mediated allergy and complement activation related pseudo-allergy (CARPA). In the case of IgE-mediated reactions, skin tests (skin prick tests [SPT] and intradermal tests [IDT]) are often positive, while in CARPA reactions, skin tests are usually negative.

In cases of adverse reactions to IV iron but nevertheless patients need to receive IV iron treatment, the recommendation is to perform an evaluation by the allergist to confirm the diagnosis and to determine if another iron formulation might be used. Stojanovic et al. (3) reported that recommencement of the index infusion or subsequent re-challenge appears to be safe in appropriate patient groups after an adverse reaction to intravenous iron. Recomencement of the initial infusion using a slow infusion protocol appears to be a safe approach following Fishbane reactions (transient flushing and truncal myalgias), or mild hypersensitivity reactions. It may also be reasonable to consider recommencement of an infusion after moderate reactions if these are transient and affect a single system. Nonetheless, there are cases in which the best alternative is desensitization. Different protocols of desensitization to iron IV have been described.

The classical protocol was reported by Altman et al. (4), in 1988. They administered intravenous methylprednisolone 50 mg at 13, 7, and 1 hours and diphenhydramine 50 mg, and ephedrine 25 mg intramuscularly at 1 hour before iron dextran administration. Twenty mL of Promit (Pharmacia, Hillerod, Denmark) were given immediately before the iron dextran. Finally, a rapid, parenteral iron dextran desensitization protocol was used, infusing 50 mL volumes of iron dextran diluted in 5% dextrose every 30 minutes, starting at a concentration of 10-7 and increasing tenfold until full strength was achieved. At the end of the protocol, the patient could receive the iron dextran dose without adverse reactions.

After that, Monaghan et al. (5) used a modified desensitization protocol reported by Altman et al, providing 2 g of elemental iron over 4 days.

In 2000, Hickam et al. (6), reported a successful protocol utilizing a pretreatment protocol described previously by Monaghan et al. and a similar graded challenge protocol with rapid dose escalation successfully utilized by Altman et al. with iron dextran.

Montandon et al. (7) reported a case series demonstrating the safety of ferric carboxymaltose desensitization. An eleven step desensitization protocol for ferric carboxymaltose was used.

Cardona et al. (8), in 2016, reported the successful treatment with IV iron dextran following a modified Altman protocol. The target dose was approximately 2 g in 3 days. The first 7 doses were administered continuously for 15 minutes each. The eight dose was administered over 30 minutes (total time 4 hours and 45 minutes). Finally, the total administered dose was 188 mg in 944 mL of normal saline.

Chapman et al. (9), in 2017 used an iron sucrose 10-step protocol with increasing concentrations, at 15 minutes intervals. The procedure started with an intravenous iron sucrose supply of 0.1 mg, and the complete cumulative dose was 100 mg. Oral courses of cetirizine (10 mg every 12 h) and montelukast (10 mg every 12 h) were continued daily for the duration of the hospitalization. Daily administration of iron continued in equal doses until completion of the estimated deficit for each patient. A total supply of 1400 mg of intravenous iron sucrose was required to complete the treatment in each of the two patients. The daily supply of 200 mg was administered intravenously in the following manner: 20 mg was injected in two doses over the first 30 min while the patient was monitored for a reaction. In the absence of further reaction, the remaining 180 mg of the daily dose was administered over a period of 4 h, with time intervals between each daily dose of 24 to 26 hours.

The last reported desensitization protocol was published by Di Girolamo et al, in 2020 (9). They reported the treatment of 4 patients with sodium ferric gluconate (12.5 mg/mL) over 4 days. Each daily sodium ferric gluconate dose was, in turn, fractionated into 4 administrations given every 30 min. Patients received a total dose over the last day of 62.5 mg and a total accumulated dose of 119.375 mg over the four days.

In summary, we can conclude that desensitization of intravenous iron formulations is an effective and safe procedure that prevents treatment discontinuation and hence allows therapeutic target achievement in both immunological contexts: IgE-mediated allergy and complement activation related pseudo-allergy (CARPA). Iron product proprieties and patient requirements are crucial in selecting the most appropriate IV iron formulation and desensitization protocol.

REFERENCES:

1) Aksan A, Işık H, Radeke H, Dignass A, et al. Systematic review with network meta-analysis: comparative efficacy and tolerability of different intravenous iron formulations for the treatment of iron deficiency anaemia in patients with inflammatory bowel disease. Aliment Pharmacol Ther. 2017;45:1303-18.

2) Rampton D, Folkersen J, Fishbane S, et al. Hypersensitivity reactions to intravenous iron: guidance for risk minimization and management. Haematologica. 2014; 99:1671-6.

3) Stojanovic S, Graudins LV, Aung AK, et al. Safety of intravenous iron following infusion reactions. J Allergy Clin Immunol: In practice. 2021; 9:1660-6.

4) Altman LC, Petersen PE. Successful Prevention of an Anaphylactoid Reaction to Iron Dextran. Ann Inter Med. 1988: 346.

5) Monaghan MS, Glasco G, St John G, et al. Safe administration of iron dextran to a patient who reacted to the test dose. South Med J [Internet]. 1994 Oct [cited 2016 Jun 7];87(10):1010–1012. Available from: http://www.ncbi.nlm.nih.gov/pubmed/7939912.

6)Hickman MA, Bernstein IL, Palascak JE. Successful administration of iron dextran in a patient who experienced a life-threatening reaction to intravenous iron dextran. Ann Allergy Asthma Immunol. 2000;84:262-3.

7) Cardona R, Sánchez J, Ramírez R. Life-threatening reaction to iron dextran: Protocol for induction tolerance. J Allergy Clin Immunol. 2015; 26:48-72

8) Montandon SV, Fajt ML, Petrov AA. A Safe and Novel Desensitization Protocol with Ferric Carboxymaltose to Treat Iron Deficiency Anemia. Curr Drug Saf. 2016; 11(2): 145–8.

9) Chapman E, Leal D, Alvarez L, Duarte M, García E. Two case reports of desensitization in patients with hypersensitivity to iron. World Allergy Organ J. 2017 Oct; 10(1): 38.

10) Di Girolamo A., AlbanesiM., Loconte F, Di Bona D, Caiaffa MF, Macchia L. Desensitization in Iron Product Allergy. Acta Haematol 2020; 17:1-4
