# Supplementary material for: Standards for practical intravenous rapid drug desensitization & delabeling: A WAO committee statement
Source: World Allergy Organ J. 2022 May 31;15(6):100640. doi: 10.1016/j.waojou.2022.100640 (PMC9163606; doi:10.1016/j.waojou.2022.100640)
Supplement: Multimedia component 12 [file mmc12.pdf]

SUPPLEMENTARY TEXT 12

DESENSITIZATION IN HEPARIN HYPERSENSITIVITY

Javier Cuesta-Herranz MD, PhD

Fundación IIS-Fundación Jiménez Díaz, Retic ARADyAL (RD16/0006/0013), Madrid (Spain).

Dr María Antonieta Guzmán Meléndez

Servicio de Inmunología y Alergias, Hospital Clínico Universidad de Chile, Santiago (Chile).

Unfractionated heparin (UFH), low-molecular-weight heparins (LMWHs), fondaparinux and danaparoid are indirect parenteral anticoagulants used in daily clinical practice. Characteristics of the patient and the anticoagulant will define the best drug to be used in each case.

Heparin formulations can elicit allergic reactions, but anaphylactic reactions are rare. The incidence has declined after the introduction of the highly purified or synthetic heparin. In this way, allergic reaction to fondaparinux is almost 20 times lower than that of other heparins (1). Nonetheless, unfractionated heparin (UFH) is routinely used to prevent thrombosis within the cardiopulmonary bypass circuit for its rapid onset of action after intravenous administration, its relative ease of monitoring, and its prompt reversal with protamine sulfate.

In case of an allergic reaction to anticoagulant drugs, the patient has to be evaluated by the allergist and complete the study with skin tests (prick testing, intradermal testing, patch testing) and challenge tests when needed with the anticoagulant involved in the reaction but also to other anticoagulants to confirm or exclude cross-reactivity. In the absence of cross-reactivity, a course of action would be to consider alternative anticoagulant strategies in the management of patients with confirmed hypersensitivity to heparin and a high risk of recurrence.

Different cross-reactive patterns to anticoagulants have been described, as documented in previously reported cases of anaphylaxis to them (2-3). There are patients with immediate hypersensitivity to UFH that tolerated LMWHs, whereas patients with delayed hypersensitivity to LMWHs tolerated UFH (4). However, the opposite is also true as patients with immediate UFH allergy, have been reported to react to LMWHs (3), and patients with delayed-type reaction to LMWHs, have reacted to to subcutaneous heparin or heparinoid (5-6).

When we need to use heparin in a heparin allergic patient, a procedure of desensitization may be the best option. Four heparin desensitization protocols had been reported in the literature (7-10). There is low- or very-low-quality evidence because they are based on a small number of clinical cases. A

premedication schedule was used in all of them. The first protocol was reported by Patriarca et al. in 1994 (7). They used a four-day heparin desensitization protocol starting with SC heparin injections increasing doses over two days, followed by IV heparin injections, increasing doses over two days. Doses of heparin were escalated in two- of three-fold increments (see Table 1). Al-Eryani et al. (8) report a three-day heparin desensitization protocol initiated by a continuous infusion of heparin 4.2 U/hour x 24 hours, followed by infusions of 42 U/hour x 24 hours, and of 208 U/hour x 24 hours.(see Table 2). Parekh et al. (9) reported a modified six-day heparin desensitization protocol based on that previously published by El-Aryani et al. This protocol starts with a lower heparin dose of 0.42 U/hour x 24 hours, followed by infusions of 4.2 U/hour x 24 hours, 42 U/hour x 24 hours, and 210 U/hour x 24 hours to a goal of 500 U/hour x 24 hours (see Table 3). Finally, Dave et al. (10) reported a successful heparin desensitization protocol in which heparin doses were continuously infused and escalated in half-log increments over 12-hour periods to goal infusion of 1000 U/hour of heparin.

Kabut et al. (11) reported a case of anaphylaxis-like symptoms due to enoxaparin whose skin/provocation tests showed cross-reactivity to other LMWHs and UFH. Fondaparinux and desensitization with UFH were found to be safe alternatives for immediate heparin allergy.

TABLE 1: Patriarca et al. Heparin Desensitization

50 UI SC	Day 1
After 40 min, 250 UI SC	Day 1
After 40 min, 500 UI SC	Day 1
500 UI SC	Day 2
After 40 min, 1500 UI SC	Day 2
After 40 min, 3000 UI SC	Day 2
500 UI IV	Day 3
After 40 min, 1500 UI IV	Day 3
After 40 min, 3000 UI IV	Day 3
5000 UI IV	Day 4

SC = subcutaneous; IV = intravenous.

TABLE 2: Al-Eryani et al. Heparin Desensitization

4.2 U/h IV	Day 1
42.0 U/h IV	Day 2
208.0 U/h IV	Day 3
5000.0 U bid SC	Until the day of surgery

SC = subcutaneous; IV = intravenous

TABLE 3: Parekh et al. Heparin Desensitization

0.42 U/hr IV	Day 1
4.20 U/h IV	Day 2
42.00 U/h IV	Day 3
210.00 U/h IV	Day 4
500.00 U/h IV	Day 5

IV = intravenous.

REFERENCES:

1) Schindewolf M, Scheuermann J, Kroll H, et al. Low allergenic potential with fondaparinux: results of a prospective investigation. Mayo Clin Proc. 2010;85:913-9.

2) Harr T, Scherer K, Tsakiris DA, Bircher AJ. Immediate type hypersensitivity to low molecular weight heparins and tolerance of unfractionated heparin and fondaparinux. Allergy. 2006;61:787-8.

3) Berkun Y, Haviv YS, Schwartz LB, Shalit M. Heparin-induced recurrent anaphylaxis. Clin Exp Allergy. 2004;34:1916-8.

4) Bircher AJ, Harr T, Hohenstein L, Tsakiris DA. Hypersensitivity reactions to anticoagulant drugs: diagnosis and management options. Allergy. 2006;61:1432–40.

5) Grims RH, Weger W, Reiter H, Arbab E, Kränke B, Aberer W. Delayed-type hypersensitivity to low molecular weight heparins and heparinoids: cross-reactivity does not depend on molecular weight. Br J Dermatol. 2007;157:514-7.

6). Grassegger A, Fritsch P, Reider N. Delayed-type hypersensitivity and cross-reactivity to heparins and danaparoid: a prospective study. Dermatol Surg. 2001;27:47-52.

7) Patriarca G, Rossi M, Schiavino D, Schinco G, Fais G, Varano C, Schiavello R: Rush desensitization in heparin hypersensitivity: A case report. Allergy 1994;49(4):292-294.

8) al-Eryani AY, al-Momen AK, Fayed DR, Allam AK: Successful heparin desensitization after heparin-induced anaphylactic shock. Thromb Res 1995;79:523-6.

9) Parekh K, Burkhart HM, Hatab A, Ross A, Muller BA: Heparin allergy: Successful desensitization for cardiopulmonary bypass. J Thorac Cardiovasc Surg 2005;130(5):1455-6.

10) Dave S, Park M. Successful Heparin Desensitization: A Case Report and Review of the Literature. J Card Surg 2008; 23:394-7.

11) Kavut AB, Koca E. Successful desensitization with un-fractionated heparin in a patient with heparin allergy and tolerance to fondaparinux. Asian Pac J Allergy Immunol 2012; 30:162-6.
