# Supplementary material for: Standards for practical intravenous rapid drug desensitization & delabeling: A WAO committee statement
Source: World Allergy Organ J. 2022 May 31;15(6):100640. doi: 10.1016/j.waojou.2022.100640 (PMC9163606; doi:10.1016/j.waojou.2022.100640)
Supplement: Multimedia component 13 [file mmc13.pdf]

SUPPLEMENTARY TEXT 13

Desensitization in Contrast Media Hypersensitivity

Javier Cuesta-Herranz MD, PhD

Fundación IIS-Fundación Jiménez Díaz, Retic ARADyAL (RD16/0006/0013), Madrid (Spain).

Dr María Antonieta Guzmán Meléndez

Servicio de Inmunología y Alergias, Hospital Clínico Universidad de Chile, Santiago (Chile).

Immediate hypersensitivity reactions to iodinated intravenous (IV) radiographic contrast material (RCM) are rare, although they may be life-threatening. The introduction of modern radioiodine contrast agents has decreased the risk of adverse reactions substantially. However, the risk of severe adverse reactions still remains measurable, with related deaths occurring in up to 1 per 100,000 administrations (1-2).

Adverse reactions to RCM could be prevented by performing an allergy workup based mainly on skin tests in order to identify the responsible compound and find safe alternatives (2-3), especially taking into account the cross-reactivity patterns found in some studies (4-5). In the last decade, indeed, several studies demonstrated the usefulness of performing skin testing with a large panel of RCM, including the suspected one, in patients with hypersensitivity reactions and using a negative skin tested compound afterwards, without systematic use of premedication (4-7).

The role of premedication is controversial (8). Concerning premedication, it should be stated that there is no consensus on its usefulness. In fact, it is not recommended by ESUR guidelines (9), due to a lack of proven effectiveness, whilst premedication is considered helpful for preventing severe reactions in the United States of America (3, 10). Regarding T-cell-mediated hypersensitivity reactions, prophylactic protocols have been used in the sporadic cases of subjects with such hypersensitivity to all RCM (11).

Some patients may either be sensitized to contrast media or will continue experiencing severe adverse reactions despite premedication. In these patients, desensitization can potentially be an option.

Desensitization is a validated preventative measure for anaphylaxis or anaphylactoid reactions. Several studies offer data on the efficacy of RCM desensitization for medical emergencies, such as cardiac catheterization.

All reviewed protocols used premedication based on corticosteroids, antihistamines with or without ephedrine, ranitidine or antileukotrines. Agardh et al, (12) in 1983, performed a desensitization protocol over 1 to 4 days. Initially a dose of 1 ml of the contrast medium in a dilution of 1: 100 was injected subcutaneously, followed by intravenous injection of the same dose. The intravenous dose

and concentration were gradually increased, the rate depending on the severity of the previous reaction and the reactions which occurred during the desensitization period. Finally, about half of the amount expected to be given during the subsequent radiologic examination was administered as a single intravenous injection. There are obvious disadvantages when using such long protocols, so it is not surprising that they are rarely used.

Ghandi et al. (13), used a rapid desensitization protocol based on a previous protocol adapted from Hong et al. (14). It was a 13 step protocol with Visipaque. They prepared several dilutions and then the patient was given escalating doses of Visipaque every 10 minutes, starting with 0.160 mg (dilution 1:10,000). Uppal et al. reported a successful case report using the protocol adopted from the above-mention study. After that, Sanan et al. (15) in 2019 proposed a desensitization protocol identical to Uppal et al. (16), but providing extra doses to the end of the protocol to accommodate cardiac catheterization delays. Al-Ahmad et al. (17) recently succesfully adapted the Brighman and Women's Hospital (Boston, USA) rapid desensitization protocol (which has been used in chemotherapy, biologics, antibiotics, and some miscellaneous drugs) to RCM desensitization (17, 18).

REFERENCES:

1.- Sodagari F, Mozaffary A, Wood CG 3rd, et al. Reactions to Both Nonionic Iodinated and Gadolinium-Based Contrast Media: Incidence and Clinical Characteristics. AJR Am J Roentgenol. 2018;210(4):715-719.

2.- Costantino MT, Romanini L, Gaeta F, et al. SIRM-SIAAIC consensus, an Italian document on management of patients at risk of hypersensitivity reactions to contrast media. Clin Mol Allergy. 2020;18:13.

3.- Sánchez-Borges M, Aberer W, Brockow K, et al. Controversies in Drug Allergy: Radiographic Contrast Media. J Allergy Clin Immunol Pract. 2019 Jan;7(1):61-65.

4.- Lerondeau B, Trechot P, Waton J, et al. Analysis of cross-reactivity among radiocontrast media in 97 hypersensitivity reactions. J Allergy Clin Immunol. 2016 Feb;137(2):633-5.

5.- Schrijvers R, Breynaert C, Ahmedali Y, Bourrain JL, Demoly P, Chiriac AM. Skin Testing for Suspected Iodinated Contrast Media Hypersensitivity. J Allergy Clin Immunol Pract. 2018 Jul-Aug;6(4):1246-54.

6.- Clement O, Dewachter P, Mouton-Faivre C, et al. Immediate Hypersensitivity to Contrast Agents: The French 5-year CIRTACI Study. EClinicalMedicine. 2018 Jul 28;1:51-61.

7.- Trautmann A, Brockow K, Behle V, Stoevesandt J. Radiocontrast Media Hypersensitivity: Skin Testing Differentiates Allergy From Nonallergic Reactions and Identifies a Safe Alternative as Proven by Intravenous Provocation. J Allergy Clin Immunol Pract. 2019 Sep-Oct;7(7):2218-24.

8.- Torres MJ, Trautmann A, Bohm I, et al. Practice parameters for diagnosing and managing iodinated contrast media hypersensitivity. Allergy. 2021;76(5):1325-39.

9.- ESUR, Guidelines on Contrast Media. www.esur-cm.org, 10.0 edition, 2018.

10.- American College of Radiology. ACR Manual on Contrast Media Version 10. Reston, VA: American College of Radiology; 2015.

11.- Romano A, Artesani MC, Andriolo M, Viola M, Pettinato R, Vecchioli-Scaldazza A. Effective prophylactic protocol in delayed hypersensitivity to contrast media: report of a case involving lymphocyte transformation studies with different compounds. Radiology. 2002 Nov;225(2):466-70.

12.- Agardh, C.-D., Arner, B., Ekholm, S., & Boijsen, E. (1983). Desensitisation as a Means of Preventing Untoward Reactions to Ionic Contrast Media. Acta Radiologica. Diagnosis, 24(3), 235–9.

13.- Gandhi S, Litt D, Chandy M, et al. Successful rapid intravenous desensitization for radioiodine contrast allergy in a patient re- quiring urgent coronary angiography. J Allergy Clin Immunol Pract 2014; 2: 101-2.

14.- Hong SJ, Bloch KJ, Wong JT. Rapid IV challenge/desensitiza- tion using iso-osmolar radiocontrast medium (RCM) in two pa- tients at high risk for anaphylactoid reactions. J Allergy Clin Immunol 2002; 109: S150.

15.- Sanan N, Rowane M, Hostoffer R. Radiologic Contrast Media Desensitization for Delayed Cardiac Catheterization. Allergy & Rhinology 2019; 10: 1–3.

16.- Uppal S, Decicco AE, Intini A, Josephson RA. Rapid desensitization to overcome contrast allergy prior to urgent coronary angiography. Int Heart J. 2018;59(3):622–5.

17.- Al-Ahmad M, Bouza TR. Successful desensitization to radiocontrast media in two high-risk cardiac patients. Ann Saudi Med 2017; 37:333-5.

18.- Sloane D, Govindarajulu U, Harrow-Mortelliti J, et al. Safety, Costs, and Efficacy of Rapid Drug Desensitizations to Chemotherapy and Monoclonal Antibodies. J Allergy Clin Immunol Pract. 2016;4(3):497-504.
