# Supplementary material for: Standards for practical intravenous rapid drug desensitization & delabeling: A WAO committee statement
Source: World Allergy Organ J. 2022 May 31;15(6):100640. doi: 10.1016/j.waojou.2022.100640 (PMC9163606; doi:10.1016/j.waojou.2022.100640)
Supplement: Multimedia component 14 [file mmc14.pdf]

SUPPLEMENTARY TEXT 14

DESENSITIZATION IN INSULIN HYPERSENSITIVITY

Javier Cuesta-Herranz MD, PhD

Fundación IIS-Fundación Jiménez Díaz, Retic ARADyAL (RD16/0006/0013), Madrid (Spain).

Dr María Antonieta Guzmán Meléndez

Servicio de Inmunología y Alergias, Hospital Clínico Universidad de Chile, Santiago (Chile).

Insulin is a peptide hormone produced by beta cells of the pancreatic islets. It is also a key component in the management of diabetes mellitus. Currently, the most widely used insulin types are human insulin (NPH, regular insulin) and insulin analogues (ie, Aspart, Lispro, Detemir, Glargine).
Allergy to insulin has been reported, although the frequency has significantly decreased with the introduction of insulin analogues (1). To diagnose insulin allergy, one must both confirm allergy to insulin and exclude allergy to additives in the formulation (ie, protamine or cresol) (2). Due to the possibility of cross-reactivity, It is important to know if patients are allergic to one or several kinds of insulins, in order to find out if there is an alternative treatment.

Immediate systemic reactions to insulin that persist despite changing the insulin preparation may need desensitization for further administration. Multiple protocols for insulin desensitization have been described in the literature, most commonly using small, increasing doses of SC insulin (3-6) or continuous subcutaneous insulin infusion (7-9), and less commonly, IV insulin (10) with successful outcomes. There has been described a great variability in the starting dose (1,000,000-fold to 1000-fold diluted from 100 IU/mL), as well as in the schedule of dose increase, from 10-fold to 2-fold, every 15 to 30 minutes to reach the target dose (11). These protocols also included pretreatment regimens adding antihistamines and corticosteroids. We should remember that during the procedure it is essential to control blood glucose levels on a regular interval and coordination with the endocrinology department is recommended. Additional protocols have reported successful insulin desensitization in patients with diabetic ketoacidosis (12-13).

REFERENCES

1) Jacquier J, Chik CL, Senior PA. (2013) A practical, clinical approach to the assessment and management of suspected insulin allergy. Diabetic Medicine 30: 977-85.

2) Ghazavi ML, Johnston GA. Insulin allergy. Clin Dermatol 2011;29:300–5.

3) Yuan T, Zhao W, Wang L, Dong Y, Li N. Continuous subcutaneous insulin infusion as an effective method of desensitization therapy for diabetic patients with insulin allergy: a 4-year single-centre experience. Clin Ther. 2016;38:2489–94.

4) Wu P, Ji C, Wang M, Zou S, Ge W. Desensitization of allergy to human insulin and its analogs by administering insulin aspart and insulin glargine. Ann Endocrinol. 2013;74:56–8.

5) Hasselman C, Pecquet C, Bismuth E et al. Continuous subcutaneous insulin infusion allows tolerance induction and diabetes treatment in a type 1 diabetic child with insulin allergy. Diabetes Metab. 2013;39:174–7.

6) Moyes V, Driver R, Croom A, Mirakian R, Chowdhury TA. Insulin allergy in a patient with type 2 diabetes successfully treated with continuous subcutaneous insulin infusion. Diabet Med. 2006;23:204–6.

7) Eguiluz-Gracia I, Rodriguez-Alvarez M, Cimarra-Alvarez M, Sanabria-Perez MC, Martinez-Cocera C. Desensitization for Insulin Allergy: A Useful Treatment Also for Local Forms. J Investig Allergol Clin Immunol 2021;22:215-235.

8) Pratt EJ, Miles P, Kerr D. Localized insulin allergy treated with continuous subcutaneous insulin. Diabet Med. 2001;18:515–6.

9) Sola-Gazagnes A, Pecquet C, Radermecker R et al. Successful treatment of insulin allergy in a type 1 diabetic patient by means of constant subcutaneous pump infusion of insulin. Diabetes Care. 2003;26:2961–2.

10) Asai M, Yoshida M, Miura Y. Immunologic tolerance to intravenously injected insulin. N Engl J Med. 2006;354:307–9.

11) Hoffman AG, Schram SE, Ercan-Fang NG, et al. Type I allergy to insulin: case report and review of localized and systemic reactions to insulin. Dermatitis 2008;19:52–8.

12) Nguyen AD, Luong CQ, Chu HC et al. Successful management of severe diabetic ketoacidosis in a type 2 diabetes patient with insulin allergy. BMC Endocr Disord 2019; 19: 121.

13) Shuster S; Borici-Mazi R, Awad S, Houlden RL. Rapid desensitization with intravenous insulin in a patient with diabetic ketoacidosis and insulin allergy. AACE Clinical Case Rep. 2020;6:e147-e150.
