# Supplementary material for: Standards for practical intravenous rapid drug desensitization & delabeling: A WAO committee statement
Source: World Allergy Organ J. 2022 May 31;15(6):100640. doi: 10.1016/j.waojou.2022.100640 (PMC9163606; doi:10.1016/j.waojou.2022.100640)
Supplement: Multimedia component 15 [file mmc15.pdf]

SUPPLEMENTARY TEXT 15

DESENSITIZATION IN FLUORESCEIN HYPERSENSITIVITY

Javier Cuesta-Herranz MD, PhD

Fundación IIS-Fundación Jiménez Díaz, Retic ARADyAL (RD16/0006/0013), Madrid (Spain).

Dr María Antonieta Guzmán Meléndez

Servicio de Inmunología y Alergias, Hospital Clínico Universidad de Chile, Santiago (Chile).

Hypersensitivity reactions to fluorescein have been reported, although they are not frequent (1). If a patient suffers a hypersensitivity reaction to fluorescein, and if future procedures with fluorescein are of main importance, the evaluation at an allergy clinic should be considered. According to current knowledge, there is no evidence of cross-reactivity between fluorescein and Indocyanine Green, and in case of a suspected hypersensitivity reaction to fluorescein, the switch to Indocyanine Green or any other comparable procedure should be considered as the main option. If an IgE-mediated mechanism is confirmed, with positive skin test results, and if there are no satisfactory alternatives, a fluorescein desensitization protocol could be considered (1).

There have been reported two fluorescein desensitization protocols. On the one hand, Nucera et al. (2) describe a patient who was successfully desensitized to fluorescein given every 30 minutes over 2 days. On the third day, the patient underwent fluorescein angiography without any adverse reaction. On the other hand, Knowles et al. (3) reported a successful intravenous desensitization to fluorescein dye in an outpatient clinic over 3 hours. The fluorescein angiogram was done 1 hour after completing the desensitization, and the patient developed minor tingling but without any objective findings or changes in her vital signs. Diphenhydramine was administered, and the itching resolved. The patient completed her procedure without sequelae.

REFERENCES:

1) Jorge Meira J, Marques ML, Falcão-Reis F, Gomes ER, Carneiro A. Immediate Reactions to Fluorescein and Indocyanine Green in Retinal Angiography: Review of Literature and Proposal for Patient's Evaluation. Clinical Ophthalmology 2020:14:171-7.

2) Nucera E, Schiavino D, Merendino E, et al. Successful fluorescein desensitization. Allergy 2003;58:458.

3) Knowles SR, Weber EA, Berbrayer CS. Allergic reaction to fluorescein dye: successful one-day desensitization. Can J Ophthalmol 2007;42:329–330.
