# Supplementary material for: Standards for practical intravenous rapid drug desensitization & delabeling: A WAO committee statement
Source: World Allergy Organ J. 2022 May 31;15(6):100640. doi: 10.1016/j.waojou.2022.100640 (PMC9163606; doi:10.1016/j.waojou.2022.100640)
Supplement: Multimedia component 16 [file mmc16.pdf]

SUPPLEMENTARY TEXT 16

Rapid Drug Desensitization in Vitamin Allergy

Dr María Antonieta Guzmán Meléndez

Servicio de Inmunología y Alergias, Hospital Clínico Universidad de Chile, Santiago (Chile).

Javier Cuesta-Herranz MD, PhD

Fundación IIS-Fundación Jiménez Díaz, Retic ARADyAL (RD16/0006/0013), Madrid (Spain).

Leucovorin is a racemic form of calcium folinate (folinic acid, derived from folic acid), and it is used in combination with antineoplastic agents as an adjuvant in chemotherapy regimens such as FOLFOX (leucovorin, 5-fluorouracil, oxaliplatin) or FOLFIRI (leucovorin, 5-fluorouracil, irinotecan). Leucovorin is also used in combination with methotrexate to antagonize its toxic effects.

Folic acid and vitamin B12 are considered essential dietary elements, and their deficiency can trigger alterations in DNA replication, which can be easily evidenced in tissues with a high rate of cellular replacement, such as the hematopoietic system.

Ureña-Tavera et al. emphasized the relevance of excluding a hypersensitivity reaction to leucovorin in patients reacting to FOLFOX or FOLFIRI chemotherapy regimens, as leucovorin is a possible culprit of these reactions, even if the concomitant chemotherapy drugs are mistakingly thought to be the obvious causal agent (1). This group reviewed hypersensitivity reactions among patients receiving chemotherapy regimens including leucovorin, between January 2013 and January 2014, at the Desensitization Program, Ramon y Cajal University Hospital, Madrid, Spain. They found 44 patients (35 on FOLFOX and 9 on FOLFIRI) with hypersensitivity reactions. Five out of these 44 patients had experienced a reaction to leucovorin. In four of these cases, leucovorin was suspended after discussion with the referring oncologist. One 80 years old lady with colorectal cancer stage IV was thought to benefit from staying on leucovorin, and thus rapid drug desensitization to leucovorin was performed successfully. This patient was well-characterized by means of a thorough allergy workup including oxaliplatin skin testing, irinotecan skin testing, leucovorin skin testing, L-isomer calcium folinate skin testing, which were all negative. Drug provocation testing (DPT) performed with leucovorin was positive (facial erythema, chills, and chest pain) and desensitization was undertaken.

This is the first report of intravenous rapid drug desensitization to leucovorin in a patient that was confirmed by DPT to be allergic. In this desensitization, the protocol used was the Brigham and Women's Hospital protocol (2).

Later on, Powell et al. reported the case of a 51 years old lady with colorectal cancer who experienced urticaria and angioedema during her first FOLFOX infusion (3). In this case, two intravenous desensitizations were performed on consecutive days, both using a 12-step protocol. The first day, with oxaliplatin, and the second day with leucovorin, both using premedication. During the desensitization to leucovorin, the patient experienced urticaria during a dose-escalation. The procedure was stopped and then successfully restarted. The patient was able to continue receiving her FOLFOX regimens uneventfully.

This publication hypothesized that reactions to leucovorin occurring on the first infusion of chemotherapy regimen containing this drug could be caused by previous sensitization to naturally occurring folates in the diet. However, Ureña-Tavera et al. observed that all their patients tolerated these products after the allergic reaction to leucovorin. Unfortunately, neither publication could obtain data on tolerance to oral folic acid supplements.

On another note, several reports described immediate hypersensitivity reactions to B-complex vitamins or their excipients when used intramuscularly (4-8).

In 1944, Mitrani (9) published the case of a 15-year-old lady who experienced a cutaneous eruption after the first injection of thiamine hydrochloride (vitamin B1). She had a background of arthralgia in both legs and bronchial asthma. The patient was studied with intradermal testing for thiamine hydrochloride and the excipient chlorobutanol. These skin tests were strongly positive for thiamine alone. Subcutaneous desensitization was undertaken using dilutions of thiamine in saline of 1:5,000, 1:1,000, 1:100 and 1:10. The initial dose was 0.1 ml of the 1:5,000 dilution, and she was administered daily increments until the target dose of thiamine was reached (see reference for protocol). She then proceeded to receive 100 mg daily of subcutaneous thiamine for 10 days uneventfully. Three months later, intradermal testing was repeated for thiamine and chlorobutanol, and these were negative.

Vitamin B12 is a complex organic compound included in the cobalamin family, which includes four major forms: cyanocobalamins, cobamamide (also known as adenosylcobalamin), hydroxycobalamin, and methylcobalamin. Some patients might experience reactions to one of these compounds and yet tolerate others (8).

Several intramuscular or subcutaneous desensitization protocols have been published for cyanocobalamin (6, 10-12). These protocols last for several days or even weeks, except for the one-day protocol published by Branco-Ferreira et al. (6).

Alves-Correira et al. (13) reported the case of a 64 years old gentleman with allergic rhinitis and asthma and a vitamin B12 deficiency secondary to a malabsortion in the context of a Barrett metaplasia. He

had received periodic 2-monthly injections of cyanocobalamin (Permadoze 1 mg/ml) and cobamamide (Jaba B12 10 mg/2 ml) for 5 years. Two hours after the last dose of cyanocobalamin, the patient experienced angioedema on the face and hands and widespread urticaria, which was controlled with oral levocetirizine 10 mg and deflazacort 30 mg. He underwent skin prick testing and intradermal testing sequentially with cyanocobalamine and cobamamide, which were positive for both drugs on intradermal 1:10. Notably, the patient experienced facial erythema and periorbital angioedema after the intradermal tests with cobamamide, which needed ebastine 20 mg and deflazacort 30 mg orally. Skin testing with the solvent agents was negative. These tests were confirmed negative on two healthy volunteers.

A rush subcutaneous desensitization was planned based on modified protocols by Caballero et al. (11) and Kartal et al. (12). They used dilutions of a commercial formulation of cyanocobalamine (Labesfal 1 mg/ml), three increasing concentrations, 10 mcg/ml, 100 mcg/ml (dose every 15 minutes), and 1,000 mcg/ml (dose every 30 minutes), to reach a target cumulative dose of 1,010 mcg, over a period of 2 hours and 30 minutes, using 9 subcutaneous injections total.

The patient continued receiving cyanocobalamin 1 mg intramuscularly 2-monthly uneventfully. Six months after desensitization, intradermal tests with cyanocobalamin were repeated and were negative.

Subcutaneous desensitization protocols are a less painful alternative to intramuscular desensitization. A protocol published by Costa et al. (10) was intramuscular. Unfortunately, there are no studies comparing efficacy and safety on both routes. The intravenous route cannot be used with commercial preparations for intramuscular use.

REFERENCES:

1) Ureña-Tavera A, Zamora-Verduga M, Madrigal-Burgaleta R, Angel-Pereira D, Berges-Gimeno MP, Alvarez-Cuesta Emilio. Hypersensitivity reactions to racemic calcium folinate (leucovorin) during FOLFOX and FOLFIRI chemotherapy administrations. J Allergy Clin Immunol 2015; 135 (4): 1066-67

2) Liu A, Fanning L, Chong H, Fernandez J, Sloane D, Sancho-Serra M. Desensitizations regimens for drug allergy: state of the art in the 21st century. Clin Exp Allergy 2011; 41: 1679-89

3) Powell S, Woelich S, Caruthers C, Dykewicz M. Successful IV desensitization to racemic leucovorin, an underappreciated cause of hypersensitivity reactions from cancer chemotherapy. Ann Allergy Asthma Immunol 2016; 117: S25

4) Hodving G. Anaphylactic reaction after injection of vitamin B12. Br Med J 1968; 3: 102

5) Lagerholm B, Lodin A, Gentele H. Hypersensitivity to phenylcarbinol preservative in vitamin B12 for injection. Acta Allergol 1958; 12: 295-8

6) Branco-Ferreira M, Clode MH, Pereira-Barbosa MA, Palma-Carlos AG. Anaphylactic reaction to hydroxycobalamin. Allergy 1997; 52: 118-9

7) Turvey SE, Cronin B, Arnold AD, Twarog FJ, Dioun AF. Adverse reactions to vitamin B12 injections due to benzyl alcohol sensitivity: successful treatment with intranasal cyanocobalamin. Allergy 2004; 59: 1023-4

8) Heyworth-Smith D, Hogan PG. Allergy to hydroxycobalamin with tolerance of cyanocobalamin. Med J Aust 2002; 177: 162-3

9) Mitrani M. Vitamin B1 hypersensitivity with desensitization: report of a case. J Immunol 1944; 15 (2): 150-53

10) Costa AC, Branco-Ferreira M, Spinola-Santos A, Pedro E, Palma-Carlos A, Pereira-Barbosa M. Hipersensibilidade a vitamina B12-A possibilidade de dessensibilizacao. Rev Port Imunoalergologia 2005; 13: 177-85

11) Caballero MR, Lukawska J, Lee TH, Dugue P. Allergy to vitamin B12: Two cases of successful desensitization with cyanocobalamin. Allergy 2007; 62: 1341-2

12) Kartal O, Gulec M, Demirel F, Yesillik S, Caliskaner Z, Sener O. Vitamin B12 allergy and successful desensitization with cyanocobalamin: a case report. Allergol Immunopathol (Madr.) 2012; 40: 324-5

13) Alves-Correia M, Gaspar A, Borrego LM, Mota I, Morais-Almeida M. Desensitization to Cyanocobalamin: Rush Protocol. J Invest Allergol Clin Immunol 2017; 27 (3): 196-97
