# Supplementary material for: Standards for practical intravenous rapid drug desensitization & delabeling: A WAO committee statement
Source: World Allergy Organ J. 2022 May 31;15(6):100640. doi: 10.1016/j.waojou.2022.100640 (PMC9163606; doi:10.1016/j.waojou.2022.100640)
Supplement: Multimedia component 17 [file mmc17.pdf]

SUPPLEMENTARY TEXT 17

Rapid Drug Desensitization in Diuretics Allergy

Dr María Antonieta Guzmán Meléndez

Servicio de Inmunología y Alergias, Hospital Clínico Universidad de Chile, Santiago (Chile).

Javier Cuesta-Herranz MD, PhD

Fundación IIS-Fundación Jiménez Díaz, Retic ARADyAL (RD16/0006/0013), Madrid (Spain).

Oral desensitization with loop diuretics has been reported on patients experiencing non-type I hypersensitivity reactions to them (1-2). Furosemide is a loop diuretic that is widely used in congestive heart failure and has only very rarely been associated with hypersensitivity reactions (< 5%), in contrast to other members of the sulfonamide family (3-4).

Shteinberg et al. (5) reported the case of a 57-years-old lady with a severe dilated cardiomyopathy and decompensated heart failure and a history of a reaction to furosemide. The clinical history was rather unclear, however, she reportedly had an oral challenge with furosemide 40 mg that triggered facial angioedema and rash 6 hours after.

Intradermal skin tests with 1% furosemide showed negative results, and an intravenous rapid desensitization was programmed using a 10-step protocol with progressively increasing doses every 20-30 minutes. The initial dose was 1 mcg (1 mcg/ml) and the final dose was 40 mg (2 ml). The patient did not experience any adverse events during the procedure and she was able to continue furosemide orally uneventfully.

Notably, the loop diuretic furosemide is widely used worldwide and the description of an intravenous rapid desensitization protocol is certainly important for patients with severe heart failure and a history of allergy to furosemide who require this drug in an emergency.

REFERENCES:

1) Slatore CG, Tilles SA. Sulfonamide hypersensitivity. Immunol Allergy Clin North Am 2004; 24: 477-90

2) Hemstreet BA, Page RL. Sulfonamide allergies and outcomes related to use of potentially cross-reactive drugs in hospitalized patients. Pharmacotherapy 2006; 26 (4): 551-7

3) Juang P, Page RL, Zolty R. A successful rapid desensitization protocol in a loop diuretic allergic patient. J Card Fail 2005; 11 (6): 481

4) Earl G, Davenport J, Narula J. Furosemide challenge in patients with heart failure and adverse reactions to sulfa-containing diuretics. Ann Intern Med 2003; 138 (4): 358-9

5) Shteinberg M, Karkabi B, Cohen S. Desensitization therapy in a patient with furosemide allergy. Brief report. Eur J Int Med 2007, 18: 69-70
